# Acyl‐Directed *ortho*‐Borylation of Anilines and C7 Borylation of Indoles using just BBr_3_

**DOI:** 10.1002/anie.201909786

**Published:** 2019-09-12

**Authors:** Saqib A. Iqbal, Jessica Cid, Richard J. Procter, Marina Uzelac, Kang Yuan, Michael J. Ingleson

**Affiliations:** ^1^ School of Chemistry University of Edinburgh Edinburgh EH9 3FJ UK; ^2^ Dept. of Chemistry University of Manchester Manchester M13 9PL UK

**Keywords:** boranes, borenium, borylation, directing groups, electrophilic aromatic substitution

## Abstract

Indoles are privileged heterocycles found in many biologically active pharmaceuticals and natural products. However, the selective functionalization of the benzenoid moiety in indoles in preference to the more reactive pyrrolic unit is a significant challenge. Herein we report that N‐acyl directing groups enable the C7‐selective C−H borylation of indoles using just BBr_3_. This transformation shows some functional‐group tolerance and notably proceeds with C6 substituted indoles. The directing group can be readily removed in situ and the products isolated as the pinacol boronate esters. Acyl‐directed electrophilic borylation can be extended to carbazoles and anilines with excellent *ortho* selectivity. 4‐amino‐indoles are amenable to this process, with acyl group installation and directed electrophilic C−H borylation enabling selective formation of C5‐BPin‐indoles.

## Graphical abstract

**B‐directed**: Acyl‐directed electrophilic C−H borylation provides access to novel C5 and C7 borylated indoles using just BBr_3_ as the borylating agent via the formation of six‐membered boracycles.

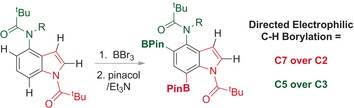

C−H borylation is a powerful methodology to form synthetically versatile C−B bonds.^1^ Numerous methods have been developed, with iridium‐catalysed C−H borylation one of the most notable.^1^ This method functionalises the pharmaceutically important heteroarene indole at the C2‐position.^2^ Alternative indole C−H borylation methods include electrophilic borylation (dominated by electronic effects)^3^ and C−H lithiation/borylation (controlled by C−H acidity).^4^ However, these also functionalise the pyrrole unit (at C3 and C2, respectively, Scheme 1 top left). Indole C−H borylation that occurs selectively on the less reactive benzenoid unit is desirable, including for accessing C5 and C7‐functionalised indoles which are motifs found in many biologically active natural products and pharmaceuticals (e.g. chloropeptin I, teleocidins, hippadine, tiplaxtinin).^5^ To date the selective C5−H/C7−H borylation of indoles in the presence of C2−H/C3−H requires prefunctionalised indoles (e.g. halide at C5/C7) or functionalisation of the more reactive C2−H/C3−H site prior to C5−H/C7−H borylation and then unmasking of the C2−H/C3−H.^6^ To the best of our knowledge, one example of directed iridium‐catalysed C−H borylation^7^ provides the only exception to these requirements (Scheme 1, middle left).^8^ This process while notable uses ruthenium and iridium catalysts and substrates containing C6 substituents are not viable (6,7‐disubstituted indoles are also bioactive motifs for example, indole isosteres of combrestatins).^5^, 6c, 9 Therefore a simple, precious metal free route for the C−H borylation of indoles that is selective for: (i) C7 (over C2), including for C6 substituted indoles, and (ii) C5 (over C3), would be highly notable particularly if using a readily removed directing group.

**Scheme 1:**
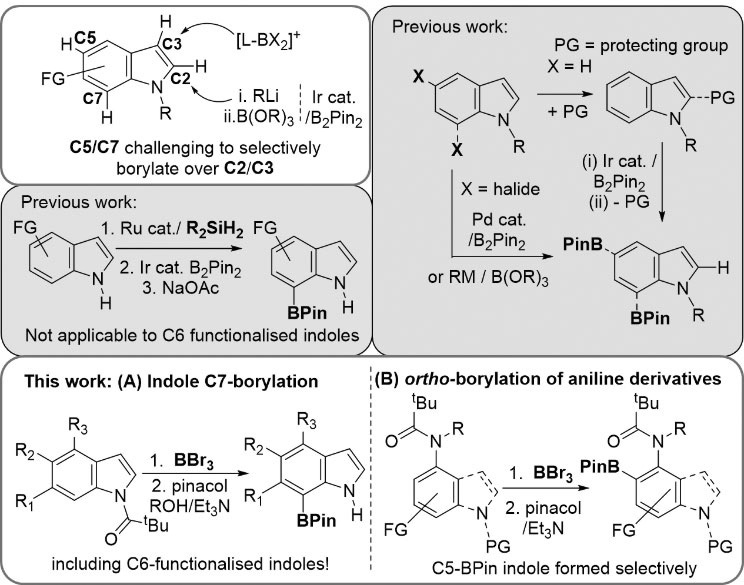
Select previous work on the borylation of indoles, specifically borylation reactions occurring at the C5 and C7 positions. Bottom inset, this work on acyl‐directed electrophilic C−H borylation at C5 and C7 using BBr_3_.

C−H borylation using BX_3_ (X=Cl or Br) is an attractive method to form organoboranes,3a, 3b, 10, 11 and directed borylation using BX_3_ has proved to be a powerful route to form B−C bonds for organic materials applications.^12^ Directed electrophilic C−H borylation is dominated by directing R_2_N‐ or N‐heterocycle groups with borylation generally forming six membered boracycles preferentially over other ring sizes.^13^ The extension of C−H borylation using BX_3_ to the C5/C7 positions of indoles would be highly attractive. However, this requires conditions that disfavour electrophilic C3−H borylation (which is relatively facile) and a directing group that: (i) is compatible with BX_3_; (ii) enables selective borylation at the desired position; (iii) is readily deprotected post C−H borylation. Transition metal‐catalysed C7−H indole functionalisation often uses bulky phosphinyl directing groups installed at N1 which are challenging to remove (requiring refluxing with LiAlH_4_),^5^, 6a, 14 however, in limited cases *N*‐acyl directing groups also have been used^5^, ^15^ and these are more readily removed. Herein we demonstrate that *N*‐acyl directing groups are compatible with BBr_3_ and lead to C7−H borylation of indoles generating useful C7‐BPin products on work up (Scheme 1, bottom). Notably, borylation is compatible with C6 substituted indoles in contrast to the iridium‐catalysed process. Furthermore, acyl directing groups also enable *ortho* C−H borylation of anilines using BBr_3_, including of 4‐amino indoles which affords C5‐BPin indoles.

To guide our selection of appropriate acyl directing groups initially we probed the thermodynamic outcome from indole borylation at C2 and C7 computationally. Notably, the C7 borylated isomer is calculated to be thermodynamically favoured over the C2 (Scheme 2) isomer in all cases, this is attributed to (i) the differing degrees of steric clash between R and the C7−H and C2−H hydrogens (as previously noted);^16^ (ii) the differing bond angles in 5 and 6‐membered boracycles, with the former leading to compressed O‐B‐C angles relative to the latter (which approaches the ideal for tetrahedral boron, Scheme 2). C7‐borylation is also calculated to be the kinetic outcome (for R=^t^Bu) based on borylation proceeding via acyl→BBr_3_ formation, [acyl→BBr_2_]^+^ formation and then S_E_Ar (see SI).

**Scheme 2:**
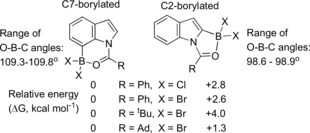
Relative energy of C2 and C7 borylated isomers calculated at the M06‐2X/6‐311G(d,p) level with a polarizable continuum model of DCM.

Based on these calculations the borylation of 1‐benzoyl‐indole, **1 a**, and 1‐pivaloyl‐indole, **2 a**, was targeted. To disfavour borenium cation formation and indole C3 borylation conditions were required avoiding coordinating exogenous base.3b, 17 For example, using reagents which lead to [(amine)BX_2_]^+^ cations (e.g. BBr_3_/ 2,6‐lutidine)^11^ led to the borylation of **2 a** at C3 selectively (see SI) with no C2 or C7 borylation observed (Scheme 3). Therefore, BCl_3_ and BBr_3_ in the absence of base were utilised.

**Scheme 3:**
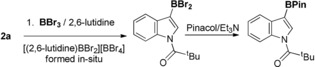
Borylation of **2 a** under conditions that generate [(amine)BX_2_]^+^ borenium cations.

While BCl_3_ resulted in no borylation of **1 a** and **2 a**, with BBr_3_ C−B bond formation proceeded with both these indoles, forming products with *δ*_11B_≈0 ppm (distinct to amide‐BBr_3_ adducts for which *δ*_11B_ is ca. −10 ppm). Subsequent addition of pinacol/Et_3_N led to formation of the pinacol boronate esters **3 a**–**5 a** (Scheme 4). The disparity between BCl_3_/ BBr_3_ also has been observed in N‐heterocycle directed borylation and the origin of this has been examined previously.^18^ The regioselectivity of borylation using BBr_3_ was assessed by NMR spectroscopy in situ and post pinacol protection. This revealed that borylation of **1 a** led to C7 and C2 borylation products (with **3 a** and **4 a** formed in a 4:1 ratio). Borylation of **2 a** with BBr_3_ led to more selective C7 borylation, with compound **5 a‐BBr_2_** the major borylated product observed in situ (in ca. 85–90 % conversion, see SI). **5 a‐BBr_2_** and **6 a‐BBr_2_** are more soluble (than benzoyl congeners) enabling in situ reaction monitoring. Notably, while minor amounts of **6 a‐BBr_2_** were observed in situ no **6 a** was observed after pinacol protection. To confirm regioselectivity ZnPh_2_ was added to the reaction mixture from **2 a**/BBr_3_ to form predominantly **7** (right, Scheme 4) which has a *δ*_11B_ of 8.6 ppm indicating a four‐coordinate boron centre (in contrast **5 a** has a broad *δ*_11B_ at 26 ppm consistent with a weaker PinB‐O_pivaloyl_ interaction). **7** was isolated in 42 % yield and subsequently crystallised with X‐ray diffraction studies confirming the formulation as the C7‐borylated regioisomer. The solid state structure of **7** revealed a B−O distance of 1.610(2) Å and a O‐B‐C angle of 104.3(1)° that deviates from that calculated for **5 a‐BBr_2_** presumably due to the different steric demand of BPh_2_ vs. BBr_2_. The complete absence of C3‐borylation is consistent with the requirement for boranes more electrophilic than BBr_3_ (e.g. borenium salts) to effect intermolecular indole C3 borylation.3b, 17

**Scheme 4:**
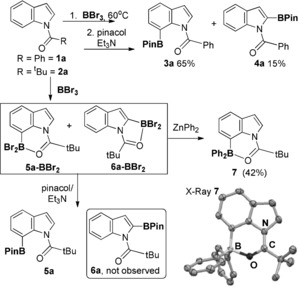
Borylation of **1 a** and **2 a** with BBr_3_ and subsequent protection with pinacol/Et_3_N or ZnPh_2_. Right, solid state structure of **7** (hydrogens omitted and ellipsoids at the 50 % probability level).^24^

The substrate scope was explored next and notably C6 substituted N‐pivaloyl‐indoles were amenable to C−H borylation using BBr_3_ in moderate to good yields (e.g., **5 c** and **5 d**) (Table 1). The 6‐methoxy derivative **2 e** was also a viable substrate, however, it underwent competitive ether cleavage with BBr_3_ producing two C7‐borylated products (**5 e** and **5 f**) in varying amounts depending on the amount of BBr_3_ used. Conditions for one‐pot C−H borylation, pinacol protection and pivaloyl deprotection simply required the addition of methanol after BPin formation and heating to 60 °C. The removal of the pivaloyl group occurs without any observable C−B cleavage. This enables three steps to be achieved in one‐pot with no solvent switches with **8 a** formed in 71 % isolated yield. These conditions were applicable to indoles substituted at C2, C3, C4, C5 and C6 (**8 g**–**8 l**), and containing electron withdrawing and donating groups. The reaction was performed on a 3 mmol scale to provide 0.82 g of **8 g** in 86 % yield. However, 5‐SMe, 5‐NO_2_ and 4‐CN substituted indoles did not furnish isolable C‐BPin products, while attempts with a bulkier group at C6, 6‐(*p*‐tolyl)‐N‐pivaloyl‐ indole, led to C2 borylation dominating (35:65 C7:C2). Compounds **8 x** are useful in Suzuki–Miyaura cross couplings, allylations and halogenations,^8^ and we note that **8 a** readily undergoes oxidation with H_2_O_2_/NaOH to form 7‐hydroxy‐indole.

**Table 1:** Substrate scope of pivaloyl‐directed C7‐borylation. 
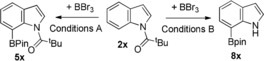

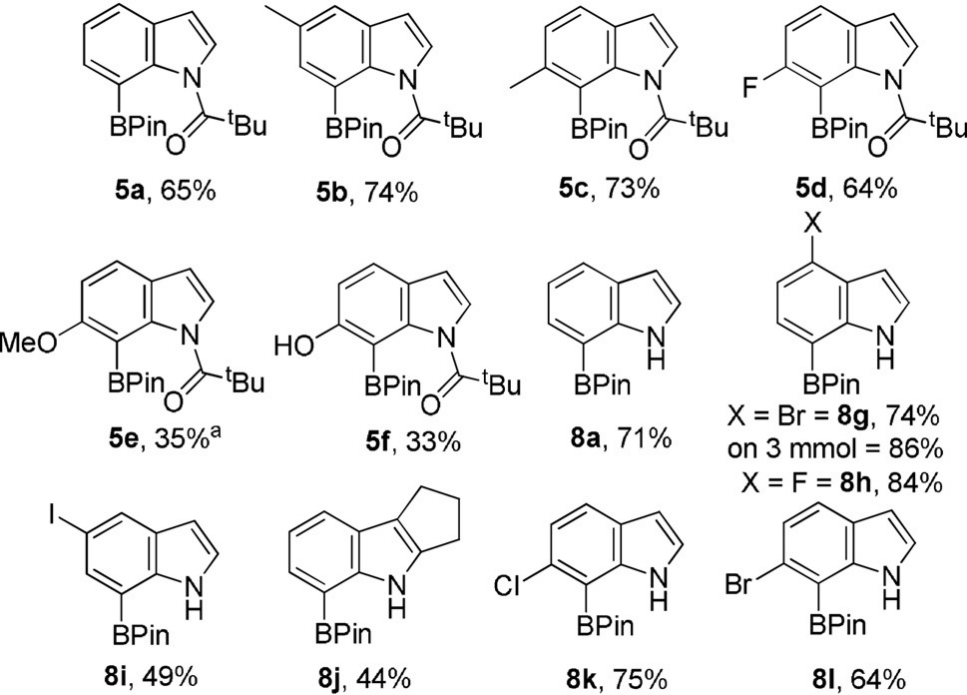

Conditions A=1. 2.2 equiv BBr_3_ in DCM. 2. +pinacol/Et_3_N. Conditions B=2.2 equiv BBr_3_ in DCM, 2. +pinacol/Et_3_N 3. +MeOH, 60 °C. Yields are of isolated products post column chromatography. [a]=using 1 equiv BBr_3_.

During substrate screening minor C2−BBr_2_ borylation (forming **6 x‐BBr_2_**) often was observed. Attempts to form the C7−BBr_2_ products (**5 x‐BBr_2_**) selectively by heating (in sealed tubes so HBr does not leave the system) failed to change the C2:C7 ratio suggesting that C−H borylation of these indoles is irreversible under these conditions. However, it was observed that the ratio of C2:C7 BBr_2_ products was different to that of the C2:C7 BPin products (with C7‐BPin increasing). Furthermore, in a number of cases the amount of **5 x**/**8 x** isolated was greater than that possible based on the observed **5 x‐BBr_2_**:**6 x‐BBr_2_** ratio (precluding C2‐selective protodeborylation during pinacol addition as the only origin of ratio changes). For example, substrate **2 k** borylates to form a **5 k‐BBr_2_**:**6 k‐BBr_2_** ratio of ca. 55:45 (by ^1^H NMR spectroscopy), however, post work up **8 k** was isolated in 75 % yield. This indicates that addition of pinacol enables C2−B protodeborylation and C7−H borylation. As the BBr_2_ products are stable to isomerisation in the presence of HBr this suggests that it is a C−B(OR)Br or C−B(OR)_2_ species that is undergoing protodeborylation and leading to more selective C7−H borylation.^19^ While the species undergoing C2→C7 isomerisation on pinacol addition is unknown Lewis/Brønsted acid initiated isomerisation of (RO)_2_B‐Aryl has been previously observed.^17^

To expand the utility of acyl‐directed electrophilic borylation other N‐heterocyclic frameworks were explored. However, *N*‐pivaloyl‐carbazole did not undergo C−H borylation using BBr_3_ (even on heating). This is attributed to steric crowding between the two proximal C−H units (at C1 and C8, Scheme 5, top left) and the pivaloyl ^t^Bu group that presumably results in large B‐O‐C‐N dihedral angles in the pivaloyl analogue of **10**. Benzoyl contains a smaller R group (phenyl relative to ^t^Bu), therefore *N*‐benzoyl carbazole, **9**, was combined with BBr_3_. This did not lead to C−H borylation at room temperature, instead the Lewis adduct, **10**, was formed which was poorly soluble in DCM facilitating isolation and characterisation (including by X‐ray diffraction, Scheme 5).

**Scheme 5:**
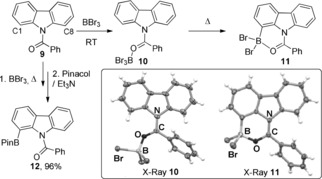
The directed borylation of *N*‐benzoyl carbazole using BBr_3_. Inset, the solid state structure of **10** and **11**, ellipsoids at the 50 % probability level.^24^

Heating combinations of **9**/ BBr_3_ led to high yielding C−H borylation at the C1 position. The C−H borylated product, **11**, could be isolated (and structurally characterised by X‐ray diffraction studies) or protected at boron in situ to furnish the pinacol boronate ester **12** in excellent yield (96 %). For **10** and **11**, the C=O (1.284(3) and 1.296(7) Å) and O−B distances (1.485(3) and 1.504(8) Å) reveal minimal difference, while the O‐B‐C angle in **11** (109.9(5)°) is comparable to that calculated for **5 a‐BBr_2_** and is close to ideal for four coordinate boron centres. Notably the B−O distance in **11** is significantly shorter than in **7** indicative of the greater Lewis acidity of the BBr_2_ moiety relative to BPh_2_.

We next explored the *ortho* borylation of anilines (Scheme 6). In previous work, borenium mediated electrophilic borylation of anilines proceeded at the *para* position.^17^ *Ortho* borylated anilines are accessible e.g., by directed lithiation of carbamate functionalised anilines,^20^ however, this approach has functional group limitations (e.g., C−Br). Both *N*‐pivaloyl and *N*‐benzoyl anilines were found to undergo selective *ortho* borylation using BBr_3_, with no *para*‐borylation observed.

**Scheme 6:**
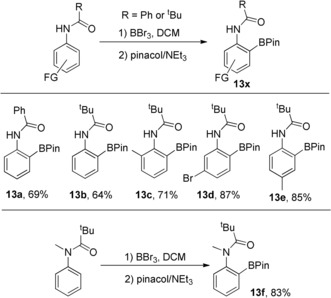
Directed *ortho* electrophilic borylation of N−H and N−Me anilines. Pivaloyl‐directed C−H borylation proceeds at 20 °C (over the course of 3–16 h), whereas as the benzoyl congener requires heating at 60 °C for 16 h.

This methodology was applicable to *o‐, m*‐ and *p*‐substituted anilines, forming **13 c**–**e** in good yield, including for a bromo containing derivative (**13 d**). Directed borylation with BBr_3_ also can be applied to tertiary amides with the *N*‐Me derivative, **13 f**, formed in good yield (83 %). Smith, Chattopadhyay and co‐workers have recently developed directed iridium‐catalysed *ortho*‐borylation of anilines using B_2_eg_2_ (eg=ethylene glycolate).^21^ This report is notable, but while excellent for N−H systems it is low yielding with *N*‐Me substituted anilines (<25 %),^21^ in contrast to the high yielding formation of **13 f** using just commercially available DCM solutions of BBr_3_.

*N*‐Bn‐indol‐4‐yl‐2,2‐dimethylpropanamide, **14**, next was investigated with it hypothesised that borylation would occur at C5 instead of C3 (the preferred site for S_E_Ar in indoles) due to the preference for the formation of six membered boracycles over seven.13c Functionalisation of the C5−H of indoles is important for accessing pharmaceuticals such as C4‐amino‐C5‐functionalised indoles (e.g. Branebrutinib).^5^, ^22^ The thermodynamics of C5 vs. C3 borylation again was probed by DFT calculations which showed the C5 isomers **15A** to be more stable than the C3 isomers **15B** (inset, Scheme 7) for both halide and pinacol substituents. C5 borylation of **14** was achieved in high selectivity with the pinacol boronate ester **16** formed in moderate yield (77 % in situ and 40 % post purification). Attempts to monitor the borylation of **14** at the BBr_2_ stage were prevented by this intermediate being poorly soluble. Finally, the ability to perform a C5/C7 double C−H borylation using BBr_3_ was demonstrated using **17** (made in one step from 4‐amino‐indole). This formed **18** selectively post pinacol protection. Notably, in situ NMR spectra prior to pinacol addition show that the C3, C7 diborylated compound, **19**, was formed as the major product and this does not isomerise on standing. However, addition of pinacol induces isomerisation of the C3−B moiety to form the thermodynamically favoured C5‐BPin unit and yield the desired C5/C7 product in good conversion (72 %).

**Scheme 7:**
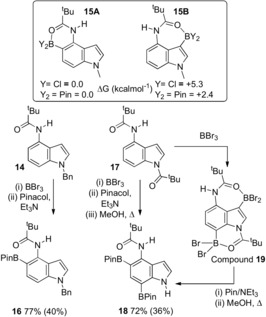
Top, relative energy of C3 and C5 borylated isomers at the M06‐2X/6‐311G(d,p) level, PCM (DCM). Bottom, borylation of **14** and **17**. In situ yields versus an internal standard, isolated yields are provided in parentheses.^23^.

In summary, *N*‐pivaloyl is an effective and readily removed directing group enabling C7 borylation of indoles and *ortho* borylation of anilines simply using commercial solutions of BBr_3_. The process is complementary to borylation with [(amine)BBr_2_]^+^ and to iridium‐catalyzed directed borylation as C6‐substituted indoles are tolerated using BBr_3_, while it has complementary functional group tolerance to directed lithiation methods. Notably, in a number of cases pinacol induced isomerisation of the initial borylated regioisomer is essential to access the desired products containing C5−B and C7−B units. Due to the simplicity of this process and the many heterocycles containing N−H groups we believe acyl‐directed borylation with BBr_3_ will be applicable to many other systems.

## Conflict of interest

The authors declare no conflict of interest.

## Supporting information

As a service to our authors and readers, this journal provides supporting information supplied by the authors. Such materials are peer reviewed and may be re‐organized for online delivery, but are not copy‐edited or typeset. Technical support issues arising from supporting information (other than missing files) should be addressed to the authors.

Supplementary
