# Supplementary material for: Acyl‐Directed ortho‐Borylation of Anilines and C7 Borylation of Indoles using just BBr3
Source: Angew Chem Int Ed Engl. 2019 Sep 12;58(43):15381–5. doi: 10.1002/anie.201909786 (PMC6856876; doi:10.1002/anie.201909786)
Supplement: Supplementary file 1 — Supplementary [file ANIE-58-15381-s001.pdf]

Angewandte Chemie
Eine Zeitschrift der Gesellschaft Deutscher Chemiker

Supporting Information

Acyl-Directed *ortho*-Borylation of Anilines and C7 Borylation of Indoles using just BBr_3

*Saqib A. Iqbal, Jessica Cid, Richard J. Procter, Marina Uzelac, Kang Yuan, and Michael J. Ingleson**

anie_201909786_sm_miscellaneous_information.pdf

Supporting information

Table of contents

General experimental....2
Preparation of (1H-indol-1-yl)(phenyl)methanone....3
Acyl-Directed Borylation of Benzoyl-Protected Indole....3
Synthesis of N-pivaloylindole substrates: GP 1....5
Preparation of 1-(7-(diphenylboraneyl)-1H-indol-1-yl)-2,2-dimethylpropan-1-one (7)....9
Pivaloyl directed C7-Borylation of Indoles: GP 2....11
One-pot borylation and de-acylation: GP 3....13
BPin transformations....16
Preparation of (9H-carbazol-9-yl)(phenyl)methanone....17
Acyl directed Borylation of Bz-Protected Carbazole....17
Acyl Directed Borylation of Benzoyl-Protected Aniline....19
Synthesis of N-pivaloylanilines: GP 4....19
Acyl-Directed Borylation of Pivaloyl-Protected Anilines: GP 5....20
Synthesis of C5 borylation substrate 14....23
C5 borylation....24
Synthesis of C5/ C7 double-borylation substrate 17....25
C5/C7 double borylation....25
References....27
Comparison of ^{1}H NMR spectra: C3 vs C7 borylated indole....28
C5 borylation: 2D NMR....29
BBr_2 boracycles: In-situ NMR spectra....31
Pinacol induced isomerisation....36
NMR spectra....39
X-Ray crystallographic data....112
Computational data....114

General experimental

All reactions were performed under an inert atmosphere using standard Schlenk techniques unless otherwise stated.

All chemicals were purchased from commercial sources and used without further purification unless stated otherwise. BBr_3 solutions were transferred to Schlenks fitted with J. Youngs valves prior to use. Dry solvents were obtained from an Inert PureSolv MD5 SPS machine or dried over CaH_2 and stored over 3 Å molecular sieves.

Bruker 300, Bruker 400 and Bruker 500 MHz NMR spectrometers were used to obtain $^{13}C\{^{1}H\}$, ^{1}H, ^{11}B and ^{19}F NMR spectra. $CDCl_3$ or CD_2Cl_2 was used as the solvent in all cases and the residual $CHCl_3$ or CH_2Cl_2 was used as reference (7.26 ppm and 5.30 ppm respectively) for $^{13}C\{^{1}H\}$ and ^{1}H NMR spectra. NMR Spectroscopy was undertaken at room temperature (~20 °C), spin-spin J coupling constants are reported in hertz (Hz) and the chemical shifts δ are reported in ppm. The multiplicity of the signals is given as s, d, t, q, dd, dt, m, for singlet, doublet, triplet, quartet, doublet of doublets, doublet of triplets and multiplet respectively.

C-B bonded and C-(N)3 ^{13}C resonances were not detected in the $^{13}C\{^{1}H\}$ NMR spectra due to broad resonances / low intensity due to quadrupolar effects.

Fourier-transform infrared (FT-IR) spectroscopy was conducted on a Perkin-Elmer Spectrum 65 FT-IR spectrometer.

Column chromatography was performed on 40-63 µm silica gel manually or using a CombiFlash NextGen 300+ Autocolumn system.

Mass spectrometry was performed by the mass spectrometry services at either the University of Manchester or the University of Edinburgh using electrospray or APCI ionisation modes

Elemental analysis was conducted at London Metropolitan University.

(1H-indol-1-yl)(phenyl)methanone (1a)

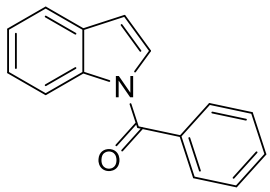

Indole (8.5 mmol, 1g), Bu_4NBr (0.12 mmol, 40 mg) and powdered NaOH (25 mmol, 1g) were dissolved in DCM (30 mL). Then benzoyl chloride (17.6 mmol, 2.04 mL) was added dropwise to the stirring solution. After 2h at r.t., the reaction mixture was dried in-vacuo and purified by column chromatography on silica gel (hexane/EtOAc=20/1) to afford the product as a white solid (1.39 g, 73 %).
Analytical data are in accordance with the literature.[1]

Acyl-Directed Borylation of Benzoyl-Protected Indole

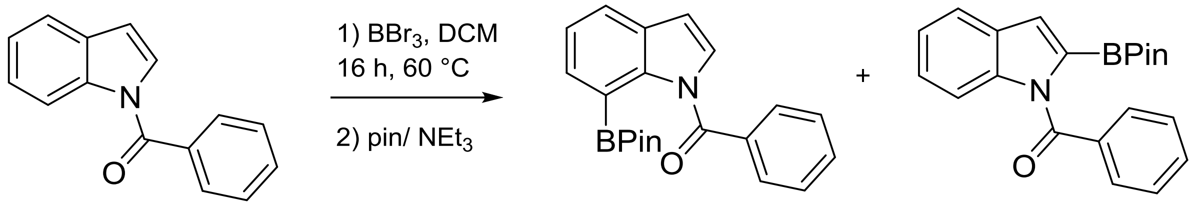

BBr_3 1M in DCM (1.05 mmol, 1.05 mL) was added to a stirred solution of (1H-indol-1-yl)(phenyl)methanone (0.5 mmol, 110.7 mg) in DCM (3 mL). After the addition, the reaction mixture was stirred for 16 hours at 60 °C. On completion of borylation, pinacol (1.5 mmol, 177.3 mg) and NEt_3 (7.5 mmol, 1.05 mL) were added to the reaction mixture and stirred for 1 hour. Volatiles were removed under vacuum and the crude product was purified by column chromatography on silica gel (hexane/EtOAc=20/1) to yield the C7-product (113.2 mg, 65 %) and C2-product (26.3 mg, 15 %).

phenyl(7-(4,4,5,5-tetramethyl-1,3,2-dioxaborolan-2-yl)-1H-indol-1-yl-)methanone (3a)

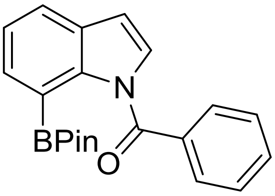

^{1}H NMR (400 MHz, CD_2Cl_2) δ 7.88 – 7.82 (m, 2H), 7.66 (td, *J* = 7.4, 6.9, 1.3 Hz, 2H), 7.57 (ddt, *J* = 8.3, 6.6, 1.3 Hz, 2H), 7.51 (dd, *J* = 7.2, 1.3 Hz, 1H), 7.34 (t, *J* = 7.5 Hz, 1H), 7.27 (d, *J* = 3.8 Hz, 1H), 6.64 (d, *J* = 3.8 Hz, 1H), 1.38 (s, 12H). **^{13}C{^{1}H} NMR** (101 MHz, CD_2Cl_2) δ 169.4, 138.4, 134.5, 132.8, 130.7, 129.9, 129.8, 129.2, 128.3, 109.3, 84.2, 25.7. **^{11}B NMR** (128 MHz, CD_2Cl_2) δ 29.42. **[Acc. Mass]** Calculated $[M+H]^+$: 348.1766 $gmol^{-1}$, Observed $[M+H]^+$: 348.1751 $gmol^{-1}$.

phenyl(2-(4,4,5,5-tetramethyl-1,3,2-dioxaborolan-2-yl)-1H-indol-1-yl)methanone (4a)

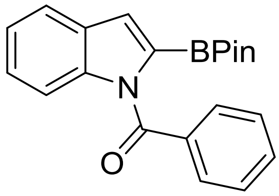

^{1}H NMR (400 MHz, CD_2Cl_2) δ 7.79 (d, *J* = 8.5 Hz, 1H), 7.73 – 7.68 (m, 2H), 7.67 – 7.63 (m, 1H), 7.63 – 7.57 (m, 1H), 7.52 – 7.45 (m, 2H), 7.33 – 7.22 (m, 2H), 7.14 (s, 1H), 1.04 (s, 12H). **^{13}C{H} NMR** (101 MHz, CD_2Cl_2) δ 170.4, 139.9, 136.9, 133.1, 130.7, 130.5, 129.2, 125.9, 123.6, 121.9, 120.1, 115.1, 84.6, 24.8. **^{11}B NMR** (128 MHz, CD_2Cl_2) δ 28.34. **[Acc. Mass]** Calculated $[M+H]^+$: 348.1766 $gmol^{-1}$, Observed $[M+H]^+$: 348.1766 $gmol^{-1}$.

Synthesis of N-pivaloylindole substrates: General procedure 1

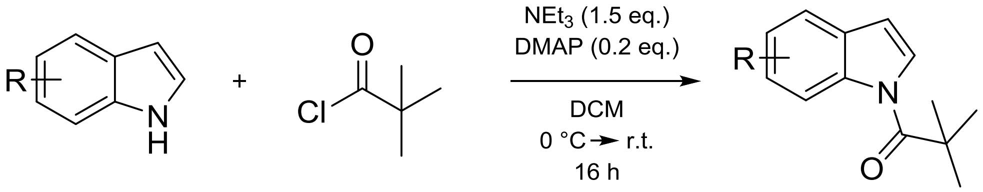

Dry triethylamine (1.5 eq.) was added to a solution of R-indole (1 eq.) and 4-N,N-dimethylaminopyridine (DMAP) (0.2 eq.) in dry DCM. The mixture was cooled to 0 °C and trimethylacetyl chloride (1.2 eq.) was added dropwise. The reaction was stirred at this temperature for 10 minutes before the ice bath was removed and the solution warmed to room temperature. The reaction mixture was stirred for 16 h. The DCM was removed on a rotary evaporator and the remaining solid was partitioned between diethyl ether and NH_4Cl (aq.), the layers were separated and the organic layer was washed once with brine. The aqueous layer was extracted three times with diethyl ether. The combined organic layers were dried over magnesium sulfate. Chromatography on silica gel (PET: EtOAc) yielded the pure product.

1-(1H-indol-1-yl)-2,2-dimethylpropan-1-one (2a)

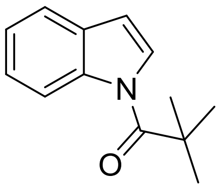

Compound **2a** was prepared following general procedure **1** using indole (1.170 g, 10 mmol), trimethylacetyl chloride (1.45 mL, 12 mmol), DMAP (0.122 g, 1 mmol), triethylamine (1.52 mL, 15 mmol) in DCM (18 mL) to give the pure product (1.891 g, 94%) as a white solid. **^{1}H NMR** (400 MHz, $CDCl_3$) δ 8.53 (d, J = 8.3 Hz, 1H), 7.74 (d, J = 3.9 Hz, 1H), 7.59 – 7.54 (m, 1H), 7.39 – 7.32 (m, 1H), 7.30 – 7.23 (m, 1H), 6.63 (d, J = 3.8 Hz, 1H), 1.53 (s, 9H). **^{13}C{H} NMR** (101 MHz, $CDCl_3$) δ 177.2, 136.9, 129.5, 125.8, 125.3, 123.7, 120.6, 117.5, 108.4, 41.4, 28.9. Analytical data are in accordance with the literature.[2]

2,2-dimethyl-1-(5-methyl-1H-indol-1-yl)propan-1-one (2b)

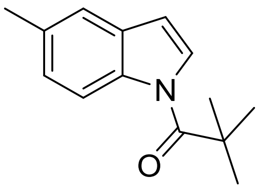

Compound **2b** was prepared following general procedure **1** using 5-methylindole (0.394 g, 3 mmol), trimethylacetyl chloride (0.44 mL, 3.6 mmol), DMAP (0.037 g, 0.3 mmol), triethylamine (0.63 mL, 4.5 mmol) in DCM (6 mL) to give the pure product (0.549 g, 85%) as a white solid. **^{1}H NMR** (400 MHz, $CDCl_3$) δ 8.38 (d, J = 8.5 Hz, 1H), 7.69 (d, J = 3.8 Hz, 1H), 7.34 (s, 1H), 7.16 (dd, J = 8.6, 1.5 Hz, 1H), 6.54 (d, J = 3.8 Hz, 1H), 2.44 (s, 3H), 1.51 (s, 9H). **^{13}C{H} NMR** (101 MHz, $CDCl_3$) δ 177.1, 135.1, 133.2, 129.8, 126.6, 125.8, 120.5, 117.1, 108.2, 41.3, 28.9, 21.4. **[Acc. Mass]** Calculated $[M+Na]^+$: 238.1202 $gmol^{-1}$, Observed $[M+Na]^+$: 238.1202 $gmol^{-1}$. **IR** (neat): ν_{max} = 1677 (vs) cm^{-1} (C=O)

2,2-dimethyl-1-(6-methyl-1H-indol-1-yl)propan-1-one (2c)

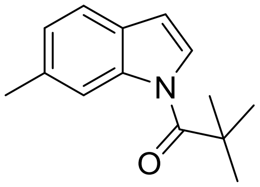

Compound **2c** was prepared following general procedure **1** using 6-methylindole (0.656 g, 5 mmol), trimethylacetyl chloride (0.74 mL, 6 mmol), DMAP (0.061 g, 0.5 mmol), triethylamine (1.05 mL, 7.5 mmol) in DCM (10 mL) to give the pure product (0.761 g, 71%) as a white solid. **^{1}H NMR** (400 MHz, $CDCl_3$) δ 8.39 – 8.36 (m, 1H), 7.66 (d, *J* = 3.9 Hz, 1H), 7.43 (d, *J* = 7.9 Hz, 1H), 7.12 – 7.08 (m, 1H), 6.57 (dd, *J* = 3.8, 0.6 Hz, 1H), 2.48 (s, 3H), 1.52 (s, 9H). **^{13}C{H} NMR** (101 MHz, $CDCl_3$) δ 177.4, 137.3, 135.3, 127.2, 125.2, 125.1, 120.2, 117.7, 108.3, 41.4, 28.8, 22.1. Analytical data are in accordance with the literature.[2]

1-(6-fluoro-1H-indol-1-yl)-2,2-dimethylpropan-1-one (2d)

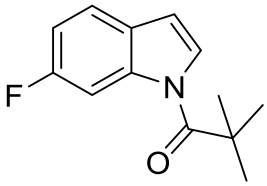

Compound **2d** was prepared following general procedure **1** using 6-fluoroindole (0.405 g, 3 mmol), trimethylacetyl chloride (0.44 mL, 3.6 mmol), DMAP (0.037 g, 0.3 mmol), triethylamine (0.63 mL, 4.5 mmol) in DCM (6 mL) to give the pure product (0.652 g, 99%) as a clear oil. **^{1}H NMR** (400 MHz, $CDCl_3$) δ 8.27 (dd, *J* = 10.9, 2.4 Hz, 1H), 7.72 (d, *J* = 3.9 Hz, 1H), 7.46 (dd, *J* = 8.5, 5.5 Hz, 1H), 7.02 (td, *J* = 8.9, 2.4 Hz, 1H), 6.59 (dd, *J* = 3.9, 0.6 Hz, 1H), 1.52 (s, 9H). **^{13}C{H} NMR** (101 MHz, $CDCl_3$) δ 177.2, 161.5 (d, J = 240.0 Hz), 137.0, 126.1 (d, J = 4.0 Hz), 125.7 (d, J = 1.4 Hz), 121.0 (d, J = 10.0 Hz), 111.9 (d, J = 24.3 Hz), 108.1, 104.9 (d, J = 29.1 Hz), 41.4, 28.7. Analytical data are in accordance with the literature.[3]

1-(6-methoxy-1H-indol-1-yl)-2,2-dimethylpropan-1-one (2e)

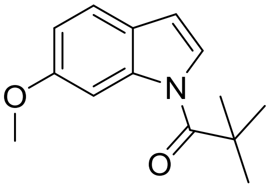

Compound **2e** was prepared following general procedure **1** using 6-methoxyindole (0.736 g, 5 mmol), trimethylacetyl chloride (0.74 mL, 6 mmol), DMAP (0.061 g, 0.5 mmol), triethylamine (1.00 mL, 7.5 mmol) in DCM (10 mL) to give the pure product (0.918 g, 80%) as a white solid. **^{1}H NMR** (400 MHz, $CDCl_3$) δ 8.15 (d, *J* = 2.3 Hz, 1H), 7.63 (d, *J* = 3.9 Hz, 1H), 7.41 (d, *J* = 8.5 Hz, 1H), 6.91 (dd, *J* = 8.5, 2.4 Hz, 1H), 6.58 – 6.51 (m, 1H), 3.88 (s, 3H), 1.52 (s, 9H). **^{13}C{H} NMR** (101 MHz, $CDCl_3$) δ 177.6, 158.6, 137.9, 124.6, 123.2, 120.9, 113.4, 108.3, 101.4, 55.8, 41.4, 28.8. Analytical data are in accordance with the literature.[4]

1-(4-bromo-1H-indol-1-yl)-2,2-dimethylpropan-1-one (2g)

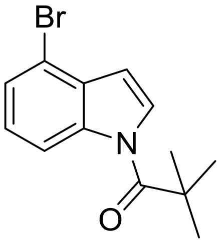

Compound **2g** was prepared following general procedure **1** using 4-bromoindole (0.627 g, 5 mmol), trimethylacetyl chloride (0.74 mL, 6 mmol), DMAP (0.061 g, 0.5 mmol), triethylamine (1.05 mL, 7.5 mmol) in DCM (10 mL) to give the pure product (1.209 g, 86%) as an off-white solid. **^{1}H NMR** (400 MHz, $CDCl_3$) δ 8.46 (d, *J* = 8.4 Hz, 1H), 7.79 (d, *J* = 3.9 Hz, 1H), 7.43 (d, *J* = 7.8 Hz, 1H), 7.21 (t, *J* = 8.1 Hz, 1H), 6.71 (d, *J* = 3.9 Hz, 1H), 1.52 (s, 9H). **^{13}C{H} NMR** (101 MHz, $CDCl_3$) δ 177.3, 137.3, 130.2, 126.6, 126.4, 126.2, 116.5, 114.6, 108.1, 41.5, 28.8. Analytical data are in accordance with the literature.[2]

1-(4-fluoro-1H-indol-1-yl)-2,2-dimethylpropan-1-one (2h)

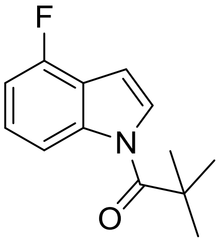

Compound **2h** was prepared following general procedure **1** using 4-fluoroindole (0.676 g, 5 mmol), trimethylacetyl chloride (0.735 mL, 6 mmol), DMAP (0.061 g, 0.5 mmol), triethylamine (1.05 mL, 7.5 mmol) in DCM (10 mL) to give the pure product (0.912 g, 83%) as a clear oil (92% purity-8% Pivaloyl chloride). **^{1}H NMR** (400 MHz, $CDCl_3$) δ 8.29 (d, *J* = 8.4 Hz, 1H), 7.72 (d, *J* = 3.9 Hz, 1H), 7.35 – 7.15 (m, 1H), 7.04 – 6.87 (m, 1H), 6.78 – 6.67 (m, 1H), 1.53 (s, 9H). **^{13}C{H} NMR** (101 MHz, $CDCl_3$) δ 177.3, 155.8 (d, *J* = 247.4 Hz), 139.0 (d, *J* = 9.0 Hz), 126.0 (d, *J* = 7.3 Hz), 125.7, 118.4 (d, *J* = 21.9 Hz), 113.5 (d, *J* = 3.8 Hz), 109.0 (d, *J* = 18.4 Hz), 103.8, 41.5, 28.8. **[Acc. Mass]** Calculated $[M+Na]^+$: 242.0952 $gmol^{-1}$, Observed $[M+Na]^+$: 242.0968 $gmol^{-1}$.

1-(5-iodo-1H-indol-1-yl)-2,2-dimethylpropan-1-one (2i)

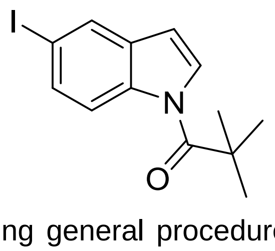

Compound **2i** was prepared following general procedure **1** using 5-iodolindole (0.729 g, 3 mmol), trimethylacetyl chloride (0.440 mL, 3.6 mmol), DMAP (0.036 g, 0.3 mmol), triethylamine (0.63mL, 4.5 mmol) in DCM (6 mL) to give the pure product (0.918 g, 94%) as a white solid. **^{1}H NMR** (500 MHz, $CDCl_3$) δ 8.27 (d, *J* = 8.8 Hz, 1H), 7.90 (d, *J* = 1.7 Hz, 1H), 7.70 (d, *J* = 3.8 Hz, 1H), 7.61 (dd, *J* = 8.8, 1.8 Hz, 1H), 6.54 (d, *J* = 3.8 Hz, 1H), 1.51 (s, 9H). **^{13}C{H} NMR** (126 MHz, $CDCl_3$) δ 177.2, 136.2, 133.7, 131.9, 129.5, 126.5, 119.3, 107.3, 87.7, 41.5, 28.8. **[Acc. Mass]** Calculated $[M+Na]^+$: 350.0012 $gmol^{-1}$, Observed $[M+Na]^+$: 350.0008 $gmol^{-1}$.

1-(2,3-dihydrocyclopenta[b]indol-4(1H)-yl)-2,2-dimethylpropan-1-one (2j)

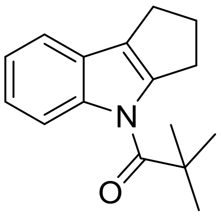

Compound **2j** was prepared following general procedure **1** using 1,2,3,4-tetrahydrocyclopent[b] indole (0.786 g, 5 mmol), trimethylacetyl chloride (0.74 mL, 6 mmol), DMAP (0.061 g, 0.5 mmol), triethylamine (1.05 mL, 7.5 mmol) in DCM (10 mL) to give the pure product (1.035 g, 86%) as a white solid. **^{1}H NMR** (500 MHz, $CDCl_3$) δ 8.01 (d, *J* = 8.0 Hz, 1H), 7.38 (d, *J* = 7.3 Hz, 1H), 7.21 (p, *J* = 7.2 Hz, 2H), 3.13 (t, *J* = 6.6 Hz, 2H), 2.78 (t, *J* = 6.5 Hz, 2H), 2.52 (p, *J* = 7.0 Hz, 2H), 1.46 (s, 9H). **^{13}C{H} NMR** (126 MHz, $CDCl_3$) δ 180.0, 142.5, 142.4, 127.1, 126.4, 123.4, 122.6, 118.7, 116.5, 41.6, 32.3, 28.2, 28.1, 23.9. **[Acc. Mass]** Calculated $[M+H]^+$: 242.1539 $gmol^{-1}$, Observed $[M+H]^+$: 242.1529 $gmol^{-1}$.

1-(6-chloro-1H-indol-1-yl)-2,2-dimethylpropan-1-one (2k)

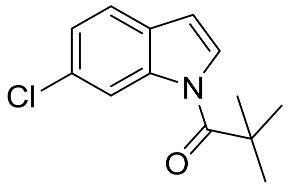

Compound **2k** was prepared following general procedure **1** using 6-chloroindole (0.758 g, 5 mmol), trimethylacetyl chloride (0.74 mL, 6 mmol), DMAP (0.061 g, 0.5 mmol), triethylamine (1.05 mL, 7.5 mmol) in DCM (10 mL) to give the pure product (0.972 g, 82%) as a white solid. **^{1}H NMR** (400 MHz, $CDCl_3$) δ 8.58 (d, *J* = 1.9 Hz, 1H), 7.72 (d, *J* = 3.9 Hz, 1H), 7.45 (d, *J* = 8.3 Hz, 1H), 7.26 – 7.22 (m, 1H), 6.59 (dd, *J* = 3.8, 0.6 Hz, 1H), 1.52 (s, 9H). **^{13}C{H} NMR** (101 MHz, $CDCl_3$) δ 177.2, 137.2, 131.2, 128.0, 126.3, 124.2, 121.2, 117.8, 108.1, 41.4, 28.8. Analytical data are in accordance with the literature.[4]

1-(6-bromo-1H-indol-1-yl)-2,2-dimethylpropan-1-one (2l)

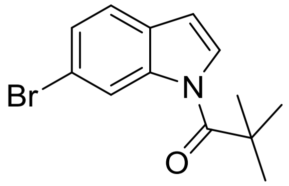

Compound **2l** was prepared following general procedure **1** using 6-Bromoindole (0.980 g, 5 mmol), trimethylacetyl chloride (0.735 mL, 6 mmol), DMAP (0.061 g, 0.5 mmol), triethylamine (1.05 mL, 7.5 mmol) in DCM (10 mL) to give the pure product (0.984 g, 70%) as a white solid. **^{1}H NMR** (400 MHz, $CDCl_3$) δ 8.77 – 8.72 (m, 1H), 7.71 (d, *J* = 3.9 Hz, 1H), 7.46 – 7.34 (m, 2H), 6.58 (dd, *J* = 3.8, 0.6 Hz, 1H), 1.51 (s, 9H). **^{13}C{H} NMR** (101 MHz, $CDCl_3$) δ 177.2, 137.5, 128.3, 126.9, 126.3, 121.6, 120.6, 119.1, 108.1, 41.4, 28.7. **[Acc. Mass]** Calculated $[M-H]^+$: 278.0175 $gmol^{-1}$, Observed $[M-H]^+$: 278.0164 $gmol^{-1}$.

6-(p-tolyl)-1H-indole

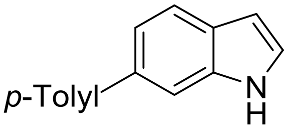

$Pd(PPh_3)_4$ (0.924 g, 0.8 mmol) was added into an ampule. A degassed solution of 6-bromoindole (1.568 g, 8 mmol) in DME (40 mL) was added to the ampule and the mixture was stirred for 15 minutes. A degassed solution of *p*-tolylboronic acid (1.088 g, 8 mmol) in ethanol (5 mL) was added followed by addition of a degassed solution of K_2CO_3 (1.658 g, 12 mmol) in water (4 mL). The mixture was heated to 78 °C for 16 h. The reaction mixture was extracted with EtOAc and washed with Brine. The organic fractions were dried over magnesium sulfate and purified by column chromatography on silica gel (EtOAc: Hexane) to give the pure product (0.526 g, 32%) as an orange solid. **^{1}H NMR** (400 MHz, $CDCl_3$) δ 8.18 (s, 1H), 7.69 (d, *J* = 8.2 Hz, 1H), 7.61 – 7.52 (m, 3H), 7.38 (dd, *J* = 8.2, 1.5 Hz, 1H), 7.29 – 7.21 (m, 3H), 6.58 (m, 1H), 2.41 (s, 3H). **^{13}C{H} NMR** (101 MHz, $CDCl_3$) δ 139.6, 136.6, 136.4, 135.7, 129.6, 127.4, 127.2, 124.8, 121.0, 119.9, 109.4, 102.7, 21.2. Analytical data are in accordance with the literature.[5]

2,2-dimethyl-1-(6-(p-tolyl)-1H-indol-1-yl)propan-1-one

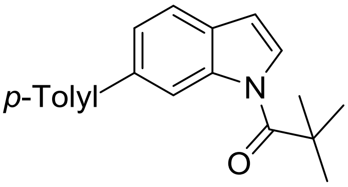

Compound **16** was prepared following general procedure **1** using 6-*p*-tolylindole (0.488 g, 2.35 mmol), trimethylacetyl chloride (0.35 mL, 2.83 mmol), DMAP (0.029 g, 0.235 mmol), triethylamine (0.50 mL, 3.53 mmol) in DCM (4 mL) to give the pure product (0.972 g, 70%) as a white solid. **^{1}H NMR** (400 MHz, $CDCl_3$) δ 8.86 – 8.77 (m, 1H), 7.75 (d, *J* = 3.8 Hz, 1H), 7.59 (d, *J* = 8.4 Hz, 3H), 7.52 (dd, *J* = 8.1, 1.6 Hz, 1H), 7.25 (d, *J* = 7.9 Hz, 2H), 6.63 (dd, *J* = 3.8, 0.6 Hz, 1H), 2.40 (s, 3H), 1.54 (s, 9H). **^{13}C{H} NMR** (101 MHz, $CDCl_3$) δ 177.4, 139.0, 138.7, 137.6, 136.8, 129.5, 128.6, 127.5, 126.2, 123.1, 120.7, 116.0, 108.2, 41.5, 28.9, 21.2. **[Acc. Mass]** Calculated $[M+Na]^+$: 314.1527 gmol^{-1}, Observed $[M+Na]^+$: 314.1515 gmol^{-1}. *Note the borylation of this compound resulted in a mixture of C2/ C7 compounds. Separation of these was not possible.*

1-(7-(diphenylboraneyl)-1H-indol-1-yl)-2,2-dimethylpropan-1-one (7)

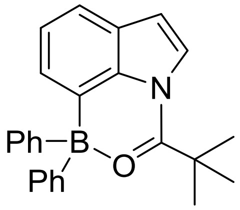

To a J-Youngs NMR tube was added compound **2a** (0.031 g, 0.15 mmol). To the tube was added DCM (0.45 mL) followed by BBr_3 1M (0.33 mL, 0.33 mmol) and the reaction mixture was stirred at 60 °C for 16 h. DCM, hexanes and (remaining) BBr_3 was removed under vacuum and Ph_2Zn (0.077 g, 0.35 mmol) was added as a solid followed by DCM (0.4 mL). The reaction mixture was stirred for 10 minutes at room temperature. The crude product was purified by column chromatography on alumina (PET: EtOAc) to yield the pure product (0.023 g, 42%). The product was dissolved in a minimum amount of DCM and layered with pentane to produce white crystals suitable for x-ray studies. **^{1}H NMR** (400 MHz, $CDCl_3$) δ 7.44 (d, *J* = 3.9 Hz, 1H), 7.41 (dd, *J* = 7.1, 1.1 Hz, 1H), 7.32 (dd, *J* = 8.0, 1.3 Hz, 4H), 7.29 (d, *J* = 7.6 Hz, 1H), 7.22 (dd, *J* = 7.6, 1.2 Hz, 1H), 7.16 (t, *J* = 7.3 Hz, 4H), 7.10 – 7.04 (m, 2H), 6.71 (d, *J* = 3.9 Hz, 1H),

1.51 (s, 9H). **^{13}C{H} NMR** (101 MHz, $CDCl_3$) δ 178.3, 136.3, 132.2, 129.9, 127.6, 127.3, 125.7, 122.9, 118.0, 116.3, 40.7, 28.6. **^{11}B NMR** (128 MHz, $CDCl_3$) δ 8.59. **[Acc. Mass]** Calculated $[M+H]^+$: 366.2024 gmol^{-1}, Observed $[M+H]^+$: 366.2030 gmol^{-1}.

Pivaloyl directed C7-Borylation of Indoles: General procedure 2

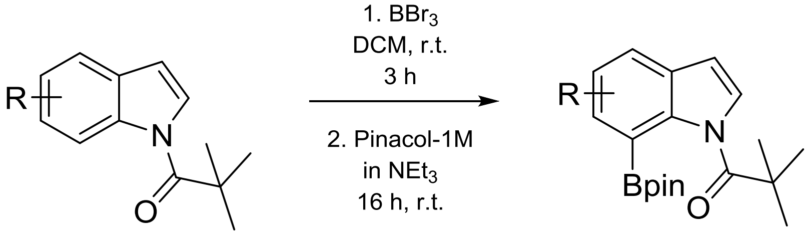

BBr_3 (1M solution, 2.2 eq,, 0.33 mmol) was added to a stirred solution of substituted indole (1 eq., 0.15 mmol) in dry DCM. The reaction mixture was stirred for 3 h at room temperature. pinacol-1M in dry NEt_3 (3.5 eq., 0.53 mmol) was then added and the reaction mixture was stirred for 16 h at room temperature. The crude reaction mixture was purified by chromatography on silica gel (PET: EtOAc) to yield the pure C7-Bpin product.

2,2-dimethyl-1-(7-(4,4,5,5-tetramethyl-1,3,2-dioxaborolan-2-yl)-1H-indol-1-yl)propan-1-one (5a)

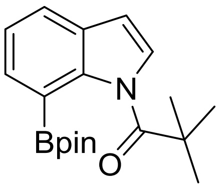

Compound **5a** was prepared following **General procedure 2** using a solution of BBr_3 1M in hexanes and **2a** (0.030 g, 0.15 mmol) to yield the product (0.032 g, 65%) as a pale-yellow oil. **^{1}H NMR** (400 MHz, $CDCl_3$) δ 7.65 (d, *J* = 3.9 Hz, 1H), 7.51 (dd, *J* = 7.7, 1.2 Hz, 1H), 7.46 – 7.42 (m, 1H), 7.31 – 7.25 (m, 1H), 6.62 (d, *J* = 3.9 Hz, 1H), 1.52 (s, 9H), 1.45 (s, 12H) **^{13}C{H} NMR** (101 MHz, $CDCl_3$) δ 177.4, 138.2, 129.7, 128.2, 124.7, 124.2, 121.1, 110.3, 83.1, 40.8, 28.8, 25.7. **^{11}B NMR** (128 MHz, $CDCl_3$) δ 25.99. **[Acc. Mass]** Calculated $[M+H]^+$: 328.2079 gmol^{-1}, Observed $[M+H]^+$: 328.2076 gmol^{-1}.

2,2-dimethyl-1-(5-methyl-7-(4,4,5,5-tetramethyl-1,3,2-dioxaborolan-2-yl)-1H-indol-1-yl)-propan-1-one (5b)

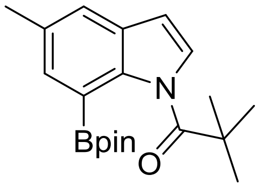

Compound **5b** was prepared following **General procedure 2** using a solution of BBr_3 1M in hexanes and **2b** (0.032 g, 0.15 mmol) to yield the product (0.038 g, 74%) as a pale-yellow oil. **^{1}H NMR** (400 MHz, $CDCl_3$) δ 7.60 (d, *J* = 3.9 Hz, 1H), 7.29 (s, 1H), 7.24 (s, 1H), 6.54 (d, *J* = 3.9 Hz, 1H), 2.42 (s, 3H), 1.50 (s, 9H), 1.45 (s, 12H). **^{13}C{H} NMR** (101 MHz, $CDCl_3$) δ 177.2, 136.4, 133.5, 130.8, 128.5, 124.7, 121.2, 110.0, 83.1, 40.7, 28.8, 25.7, 21.5. **^{11}B NMR** (128 MHz, $CDCl_3$) δ 26.21. **[Acc. Mass]** Calculated $[M+H]^+$: 342.2235 gmol^{-1}, Observed $[M+H]^+$: 342.2229 gmol^{-1}. **IR** (neat): ν_{max} = 1669 (vs) cm^{-1} (C=O)

2,2-dimethyl-1-(6-methyl-7-(4,4,5,5-tetramethyl-1,3,2-dioxaborolan-2-yl)-1H-indol-1-yl)propan-1-one (5c)

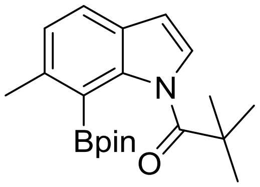

Compound **5c** was prepared following **General procedure 2** using a solution of BBr_3 1M in heptanes and **2c** (0.032 g, 0.15 mmol) to yield the product (0.037 g, 73%) as a clear oil. ^{1}**H NMR** (400 MHz, $CDCl_3$) δ 7.56 (d, *J* = 4.0 Hz, 1H), 7.36 (d, *J* = 7.8 Hz, 1H), 7.08 (d, *J* = 8.5 Hz, 1H), 6.56 (d, *J* = 3.9 Hz, 1H), 2.58 (s, 3H), 1.50 (s, 9H), 1.48 (s, 12H). ^{13}**C{H} NMR** (101 MHz, $CDCl_3$) δ 177.2, 139.9, 139.1, 126.6, 126.3, 123.9, 120.7, 110.4, 83.0, 40.6, 28.8, 26.9, 22.1. ^{11}**B NMR** (128 MHz, $CDCl_3$) δ 25.02. **[Acc. Mass]** Calculated $[M+H]^+$: 342.2239 $gmol^{-1}$, Observed $[M+H]^+$: 342.2243 $gmol^{-1}$.

1-(6-fluoro-7-(4,4,5,5-tetramethyl-1,3,2-dioxaborolan-2-yl)-1H-indol-1-yl)-2,2-dimethylpropan-1-one (5d)

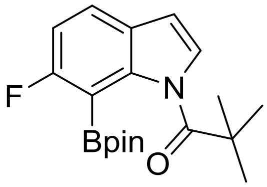

Compound **5d** was prepared following **General procedure 2** using a solution of BBr_3 1 M in heptanes and **2d** (0.033 g, 0.15 mmol) to yield the product (0.032 g, 64%) as a pale yellow oil. ^{1}**H NMR** (400 MHz, $CDCl_3$) δ 7.62 (d, *J* = 3.9 Hz, 1H), 7.43 (dd, *J* = 8.5, 5.6 Hz, 1H), 6.99 (t, 1H), 6.59 (d, *J* = 3.9 Hz, 1H), 1.50 (s, 9H), 1.46 (s, 12H). ^{13}**C{H} NMR** (101 MHz, $CDCl_3$) δ 177.4, 164.8 (d, *J* = 237.9 Hz), 138.4 (d, *J* = 14.3 Hz), 124.9 (d, *J* = 4.0 Hz), 124.7 (d, *J* = 1.5 Hz), 121.9 (d, *J* = 10.6 Hz), 112.3 (d, *J* = 27.2 Hz), 109.9, 83.7, 40.8, 28.7, 25.9. ^{11}**B NMR** (128 MHz, $CDCl_3$) δ 25.26. ^{19}**F NMR** (376 MHz, $CDCl_3$) δ -108.89 – -108.94 (m). **[Acc. Mass]** Calculated $[M+H]^+$: 346.1984 $gmol^{-1}$, Observed $[M+H]^+$: 346.1982 $gmol^{-1}$.

1-(6-methoxy-7-(4,4,5,5-tetramethyl-1,3,2-dioxaborolan-2-yl)-1H-indol-1-yl)-2,2-dimethylpropan-1-one (5e)

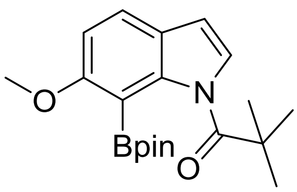

Compound **5e** was prepared following an adaptation of **General procedure 2** using a solution of BBr_3 1M in heptanes (0.12 mL, 0.12 mmol), Pinacol 1M in NEt_3 (0.15 mL, 0.15 mmol) and **2e** (0.023 g, 0.10 mmol) to yield the product (0.013 g, 35%) as an off-white solid. ^{1}**H NMR** (400 MHz, $CDCl_3$) δ 7.54 (d, *J* = 3.9 Hz, 1H), 7.41 (d, *J* = 8.5 Hz, 1H), 6.88 (d, *J* = 8.4 Hz, 1H), 6.53 (d, *J* = 3.9 Hz, 1H), 3.85 (s, 3H), 1.49 (s, 9H), 1.46 (s, 112H). ^{13}**C{H} NMR** (101 MHz, $CDCl_3$) δ 176.9, 162.5, 139.2, 123.9, 123.1, 121.5, 109.8, 109.4, 83.2, 57.2, 40.7, 28.7, 26.0.^{11}**B NMR** (128 MHz, $CDCl_3$) δ 26.50. **[Acc. Mass]** Calculated $[M+H]^+$: 358.2184 $gmol^{-1}$, Observed $[M+H]^+$: 358.2178 $gmol^{-1}$.

1-(6-hydroxy-7-(4,4,5,5-tetramethyl-1,3,2-dioxaborolan-2-yl)-1H-indol-1-yl)-2,2-dimethylpropan-1-one (5f)

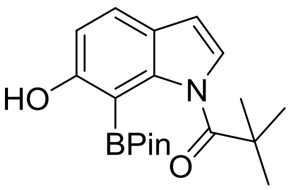

Compound **5f** was prepared following **General procedure 2** using a solution of BBr_3 1M in heptanes and **2e** (0.034 g, 0.15 mmol) to yield the product (0.016 g, 33%) as an off-white solid. ^{1}**H NMR** (400 MHz, $CDCl_3$) δ 7.49 (d, *J* = 3.9 Hz, 1H), 7.37 (d, *J* = 8.4 Hz, 1H), 6.85 (d, *J* = 8.4 Hz, 1H), 6.79 (s, 1H), 6.56 (d, *J* = 3.9 Hz, 1H), 1.51 (s, 9H), 1.43 (s, 12H). ^{13}**C{H} NMR** (126 MHz, $CDCl_3$) δ 177.9, 159.2, 139.6, 123.6, 122.8, 121.8, 113.4, 111.1, 83.3, 40.8, 28.7, 25.9. ^{11}**B NMR** (128 MHz, $CDCl_3$) δ 23.13. **[Acc. Mass]** Calculated $[M+H]^+$: 344.2031 $gmol^{-1}$, Observed $[M+H]^+$: 344.2025 $gmol^{-1}$.

One-pot borylation and de-acylation: General procedure 3

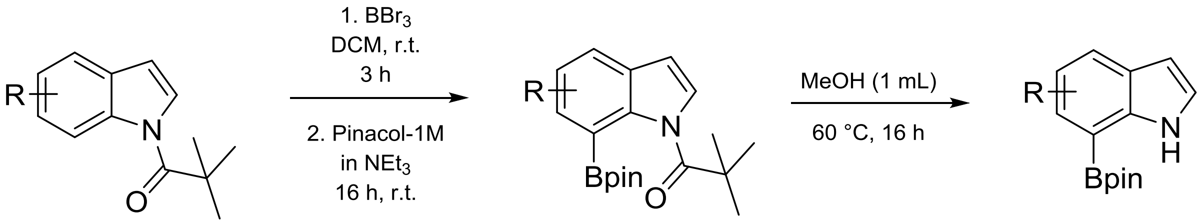

BBr_3 1M (2.2 eq. 0.33 mmol) was added to a stirred solution of substituted Indole (1 eq., 0.15 mmol) in dry DCM. The reaction mixture was stirred for 3 h at room temperature. pinacol-1M in dry NEt_3 (3.5 eq., 0.53 mmol) was then added and the reaction mixture was stirred for 16 h at room temperature. 'Wet' MeOH (1 mL) was added and the reaction mixture was heated to 60 ℃ and stirred for a further 16 h. The crude reaction mixture was purified by chromatography on silica gel (PET: EtOAc) to yield the pure **de-acylated** C7-Bpin product.

7-(4,4,5,5-tetramethyl-1,3,2-dioxaborolan-2-yl)-1H-indole (8a)

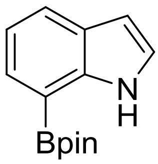

Compound **8a** was prepared following **General procedure 3** using a solution of BBr_3 1M in heptanes and **2a** (0.030 g, 0.15 mmol) to yield the product (0.026 g, 71%) as a white solid.^{1}**H NMR** (400 MHz, $CDCl_3$) δ 9.25 (s, 1H), 7.78 (d, *J* = 7.9 Hz, 1H), 7.66 (d, *J* = 6.9 Hz, 1H), 7.30 – 7.23 (m, 1H), 7.14 (t, *J* = 7.5 Hz, 1H), 6.59 – 6.52 (m, 1H), 1.41 (s, 12H). ^{13}**C{H} NMR** (101 MHz, $CDCl_3$) δ 141.1, 129.4, 126.9, 124.4, 124.2, 119.4, 102.1, 84.0, 25.2. ^{11}**B NMR** (128 MHz, $CDCl_3$) δ 31.13. **[Acc. Mass]** Calculated $[M+H]^+$: 244.1503 $gmol^{-1}$, Observed $[M+H]^+$: 244.1503 $gmol^{-1}$. Analytical data are in accordance with the literature.[6]

4-bromo-7-(4,4,5,5-tetramethyl-1,3,2-dioxaborolan-2-yl)-1H-indole (8g)

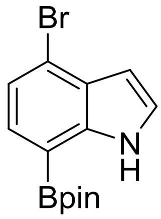

Compound **8g** was prepared following **General procedure 3** using a solution of BBr_3 1M in heptanes and **2g** (0.042 g, 0.15 mmol) to yield the product (0.036 g, 74%) as a white solid. **^{1}H NMR** (400 MHz, $CDCl_3$) δ 9.35 (s, 1H), 7.49 (d, *J* = 7.6 Hz, 1H), 7.34 – 7.28 (m, 2H), 6.62 – 6.58 (m, 1H), 1.40 (s, 12H). **^{13}C{H} NMR** (101 MHz, $CDCl_3$) δ 141.3, 130. 1, 127.9, 124.8, 122.6, 119.2, 102.5, 84.2, 25.1. **^{11}B NMR** (128 MHz, $CDCl_3$) δ 31.06. **[Acc. Mass]** Calculated $[M+H]^+$: 322.0608 $gmol^{-1}$, Observed $[M+H]^+$: 322.0609 $gmol^{-1}$.

This reaction was shown to work on a larger scale. Using **2g** (0.840 g, 3 mmol) gave **8g** (0.825 g, 86%). Note: In this case the addition of pinacol was done dropwise at 0 °C and an additional 14 mL of DCM was added at this stage to facilitate efficient stirring.

4-fluoro-7-(4,4,5,5-tetramethyl-1,3,2-dioxaborolan-2-yl)-1H-indole (8h)

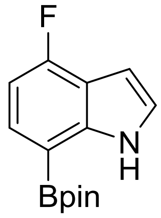

Compound **8h** was prepared following **General procedure 3** using a solution of BBr_3 1M in heptanes and **2h** (0.033 g, 0.15 mmol) to yield the product (0.033 g, 84%) as a white solid. **^{1}H NMR** (400 MHz, $CDCl_3$) δ 9.33 (s, 1H), 7.61 (dd, *J* = 7.9, 5.6 Hz, 1H), 7.25 – 7.22 (m, 1H), 6.81 (dd, *J* = 10.5, 7.8 Hz, 1H), 6.64 (dd, *J* = 3.3, 2.3 Hz, 1H), 1.40 (s, 12H). **^{13}C{H} NMR** (101 MHz, $CDCl_3$) δ 159.3 (d, *J* = 252.2 Hz), 144.3 (d, *J* = 12.3 Hz), 130.8 (d, *J* = 8.0 Hz), 124.1, 116.0 (d, *J* = 21.5 Hz), 104.8 (d, *J* = 18.7 Hz), 98.3, 84.0, 25.1. **^{11}B NMR** (128 MHz, $CDCl_3$) δ 30.85. **^{19}F NMR** (376 MHz, $CDCl_3$) δ -116.25 – -116.30 (m). **[Acc. Mass]** Calculated $[M+H]^+$: 262.1412 $gmol^{-1}$, Observed $[M+H]^+$: 262.1433 $gmol^{-1}$.

5-iodo-7-(4,4,5,5-tetramethyl-1,3,2-dioxaborolan-2-yl)-1H-indole (8i)

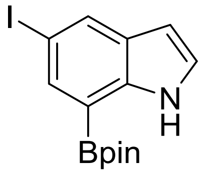

Compound **8i** was prepared following **General procedure 3** using a solution of BBr_3 1M in heptanes and **2i** (0.049 g, 0.15 mmol) to yield the product (0.027 g, 49%) as a white solid. **^{1}H NMR** (400 MHz, $CDCl_3$) δ 9.21 (s, 1H), 8.09 (dd, *J* = 1.8, 0.7 Hz, 1H), 7.89 (d, *J* = 1.7 Hz, 1H), 7.23 – 7.19 (m, 1H), 6.47 (dd, *J* = 3.2, 2.2 Hz, 1H), 1.39 (s, 12H). **^{13}C{H} NMR** (101 MHz, $CDCl_3$) δ 140.1, 137.0, 132.8, 129.7, 125.1, 101.5, 84.3, 83.5, 25.1. **^{11}B NMR** (128 MHz, $CDCl_3$) δ 30.46. **[Acc. Mass]** Calculated $[M+H]^+$: 370.0472 $gmol^{-1}$, Observed $[M+H]^+$: 370.0464 $gmol^{-1}$. Analytical data are in accordance with the literature.[6]

5-(4,4,5,5-tetramethyl-1,3,2-dioxaborolan-2-yl)-1,2,3,4-tetrahydrocyclopenta[b]indole (8j)

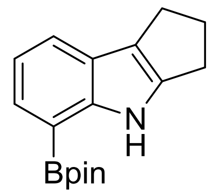

Compound **8j** was prepared following **General procedure 3** using a solution of BBr_3 1M in heptanes and **2j** (0.037 g, 0.15 mmol) to yield the product (0.019 g, 44%) as a white solid. **^{1}H NMR** (400 MHz, $CDCl_3$) δ 8.94 (s, 1H), 7.61 – 7.49 (m, 2H), 7.09 (dd, *J* = 7.8, 7.2 Hz, 1H), 2.97 – 2.89 (m, 2H), 2.89 – 2.79 (m, 2H), 2.55 (p, *J* = 7.1 Hz, 2H), 1.41 (s, 12H). **^{13}C{H} NMR** (101 MHz, $CDCl_3$) δ 146.5, 143.9, 127.7, 123.8, 122.1, 119.2, 119.0, 83.9, 28.9, 26.2, 25.1, 24.6. **^{11}B NMR** (128 MHz, $CDCl_3$) δ 31.24. **[Acc. Mass]** Calculated $[M+H]^+$: 284.1819 $gmol^{-1}$, Observed $[M+H]^+$: 284.1790 $gmol^{-1}$. Analytical data are in accordance with the literature.[6]

6-chloro-7-(4,4,5,5-tetramethyl-1,3,2-dioxaborolan-2-yl)-1H-indole (8k)

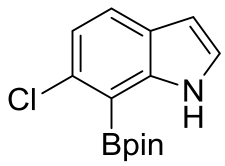

Compound **8k** was prepared following **General procedure 3** using a solution of BBr_3 1M in heptanes and **2k** (0.035 g, 0.15 mmol) to yield the product (0.031 g, 75%) as a white solid. **^{1}H NMR** (400 MHz, $CDCl_3$) δ 9.55 (s, 1H), 7.63 (d, *J* = 8.4 Hz, 1H), 7.26 (dd, *J* = 5.6, 2.3 Hz, 1H), 7.14 (d, *J* = 8.4 Hz, 1H), 6.52 (dd, *J* = 3.2, 2.2 Hz, 1H), 1.45 (s, 12H). **^{13}C{H} NMR** (101 MHz, $CDCl_3$) δ 142.2, 135.3, 125.6, 124.8, 124.6, 121.9, 102.2, 84.0, 25.1. **^{11}B NMR** (128 MHz, $CDCl_3$) δ 30.87. **[Acc. Mass]** Calculated $[M+H]^+$: 278.1114 $gmol^{-1}$, Observed $[M+H]^+$: 278.1113 $gmol^{-1}$.

6-bromo-7-(4,4,5,5-tetramethyl-1,3,2-dioxaborolan-2-yl)-1H-indole (8l)

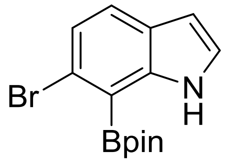

Compound **8l** was prepared following **General procedure 3** using a solution of BBr_3 1M in heptanes and **2l** (0.042 g, 0.15 mmol) to yield the product (0.031 g, 64%) as a white solid. **^{1}H NMR** (400 MHz, $CDCl_3$) δ 9.54 (s, 1H), 7.55 (d, *J* = 8.3 Hz, 1H), 7.32 (d, *J* = 8.3 Hz, 1H), 7.23 (dd, *J* = 3.2, 2.4 Hz, 1H), 6.51 (dd, *J* = 3.2, 2.2 Hz, 1H), 1.43 (s, 12H). **^{13}C{H} NMR** (101 MHz, $CDCl_3$) δ 142.7, 125.9, 125.1, 124.8, 124.7, 123.5, 102.2, 84.1, 25.2. **^{11}B NMR** (128 MHz, $CDCl_3$) δ 30.89. **[Acc. Mass]** Calculated $[M+Na]^+$: 344.0431 $gmol^{-1}$, Observed $[M+Na]^+$: 344.0452 $gmol^{-1}$.

BPin transformations

7-Hydroxyindole

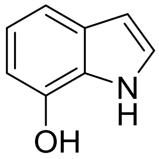

To a solution of compound **8a** (0.0240 g, 0.1 mmol) in THF (1.5 mL) was added $NaOH_{(aq)}$ (0.011 g, 0.53 M). To this was slowly added H_2O_2 (30% aqueous solution, 0.05 mL). The reaction mixture was stirred at room temperature for 3 h. The reaction mixture was quenched by addition of a 3:1 ratio of NH_4OH: NH_4Cl (8 mL). The product was extracted with EtOAc and washed with Brine, dried over Magnesium sulfate and purified by column chromatography on silica-gel (Hexane: EtOAc) to give the pure product (0.007 g, 53%) as a white solid. **^{1}H NMR** (500 MHz, $CDCl_3$) δ 8.37 (s, 1H), 7.26 (d, *J* = 8.0 Hz, 1H), 7.23 – 7.19 (m, 1H), 6.95 (t, *J* = 7.8 Hz, 1H), 6.58 (d, *J* = 7.5 Hz, 1H), 6.55 (dd, *J* = 3.0, 2.2 Hz, 1H), 4.97 (s, 1H). **^{13}C{H} NMR** (126 MHz, $CDCl_3$) δ 141.6, 130.3, 125.9, 124.3, 120.2, 113.9, 106.5, 103.1. Analytical data are in accordance with the literature.[7]

7-Chloroindole

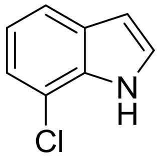

To a stirred solution of compound **8a** (0.046 g, 0.19 mmol) in MeOH (2.4 mL) was added Cu(II)Cl_2 (0.114 g, 0.67 mmol) in H_2O (2.4 mL). The solution was stirred at 80 ℃ for 4.5 h. The reaction mixture was diluted with EtOAc. The organic layer was separated and the aqueous layer extracted with EtOAc. The combined organic fractions were dried over Magnesium sulfate and the product purified by column chromatography on silica-gel (Hexane: EtOAc) to give the pure product (0.012 g, 42%) as a white solid. **^{1}H NMR** (500 MHz, $CDCl_3$) δ 8.37 (s, 1H), 7.55 (d, *J* = 7.9 Hz, 1H), 7.27 (d, *J* = 2.9 Hz, 1H), 7.21 (d, *J* = 7.6 Hz, 1H), 7.06 (t, *J* = 7.8 Hz, 1H), 6.61 (dd, *J* = 3.1, 2.2 Hz, 1H). **^{13}C{H} NMR** (126 MHz, $CDCl_3$) δ 133.3, 129.4, 124.9, 121.5, 120.7, 119.5, 116.7, 103.9. Analytical data are in accordance with the literature.[6]

(9H-carbazol-9-yl)(phenyl)methanone (9)

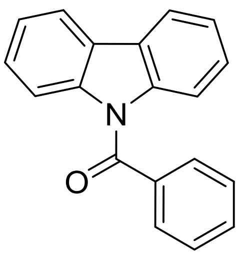

NaH (60% dispersion in mineral oil, 480 mg, 12 mmol) was added to a stirred solution of carbazole (1 g, 6 mmol) in dry THF (40 mL) at 0 °C. The reaction mixture was stirred at room temperature for 30 min. Subsequently, the resulting mixture was added to a solution of benzoyl chloride (1.4 mL, 12 mmol) in THF (20 mL) at 0 °C. After stirring for 10 min at 0 °C and 2 h at room temperature, the reaction mixture was quenched and extracted with DCM three times. The combined organic phases were collected and dried over $MgSO_4$. After filtration and evaporation of the solvents under reduced pressure, the crude product was purified by column chromatography on silica gel (petroleum ether/EtOAc=20/1) to yield the product as a white solid (1.20 g, 74%).
Analytical data are in accordance with the literature.[8]

Acyl directed Borylation of Bz-Protected Carbazole: Adduct (10)

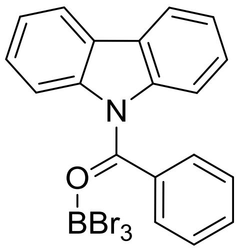

BBr_3 1M in DCM (0.42 mmol, 0.42 mL) was added to a stirred solution of (9H-carbazol-9-yl)(phenyl)methanone (0.2 mmol, 54.3 mg) in DCM (1 mL). After the addition, the reaction mixture was stirred for 30 min during which a bright yellow precipitate formed. This solid was isolated by filtration and washed three times with pentane (70 mg, 67 %). Redissolving this solid in DCM and layering with hexane led to single crystals suitable for X-ray diffraction studies. **^{1}H NMR** (400 MHz CD_2Cl_2) δ 8.99 (v. br, 1H), 7.97 (d, *J* = 7.8 Hz, 2H), 7.92 (d, *J* = 7.7 Hz, 2H), 7.87 (t, *J* = 7.7 Hz, 1H), 7.70 (t, *J* = 7.8 Hz, 2H), 7.54 (br s, 2H), 7.37 (v. br, 2H), 5.94 (v. br, 1H). **^{1}H NMR** (500 MHz, CD_2Cl_2, -25°C) δ 9.06 (d, *J* = 7.4 Hz, 1H), 8.13 – 7.84 (m, 5H), 7.82 – 7.60 (m, 4H), 7.55 – 7.39 (m, 1H), 7.05 (d, *J* = 8.0 Hz, 1H), 5.70 (d, *J* = 7.7 Hz, 1H). **^{13}C{^{1}H} NMR** (101 MHz, CD_2Cl_2) δ 172.8, 138.4, 135.0, 130.4, 129.9, 129.2 (br), 121.2, 119.6 (v. br). **^{11}B NMR** (128 MHz, CD_2Cl_2) δ -10.24.

Boracycle (11) was prepared according to the following procedure

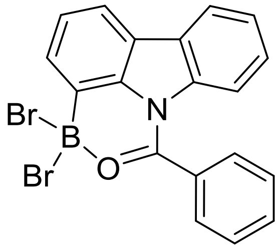

BBr_3 1M in DCM (0.42 mmol, 0.42 mL) was added to a stirred solution of **(9H-carbazol-9-yl)(phenyl)methanone** (0.2 mmol, 54.3 mg) in DCM (1mL). After the addition, the reaction mixture was heated for 4 hours at 60°C after which a pale yellow precipitate formed. This solid

was isolated by filtration and washed three time with pentane (72 mg, 82 %). X-ray quality crystals were obtained by redissolving this solid in DCM and layering with hexane. **^{1}H NMR** (400 MHz, CD_2Cl_2) δ 7.99 (d, *J* = 7.7 Hz, 1H), 7.93 – 7.84 (m, 4H), 7.78 – 7.67 (m, 4H), 7.52 (t, *J* = 7.6 Hz, 1H), 7.24 (dd, *J* = 8.7, 7.4 Hz, 1H), 6.95 (d, *J* = 8.3 Hz, 1H). **^{13}C{^{1}H} NMR** (101 MHz, CD_2Cl_2) δ 165.5, 137.0, 136.2, 135.4, 131.8, 130.9, 130.8, 130.2, 129.8, 129.4, 128.5, 128.1, 125.1, 122.5, 120.7, 117.6. **^{11}B NMR** (128 MHz, CD_2Cl_2) δ 0.56. Elemental analysis calcd for $C_{19}H_{12}BBr_2NO$: C 51.76, H 2.74, N 3.18, found: C 51.84, H 2.85, N 3.10.

phenyl(1-(4,4,5,5-tetramethyl-1,3,2-dioxaborolan-2-yl)-9H-carbazol-9-yl)methanone (12)

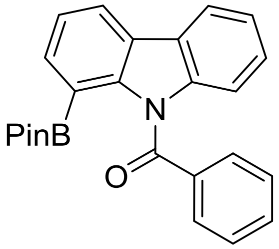

BBr_3 1M in DCM (1.05 mmol, 1.05 mL) was added to a stirred solution of (9H-carbazol-9-yl)(phenyl)methanone (0.5 mmol, 135.7 mg) in DCM (3mL). After the addition, the reaction mixture was stirred for 4 hours at 60°C. Then, pinacol (1.5 mmol, 177.3 mg) and NEt_3 (7.5 mmol, 1.05 mL) were added to the reaction mixture and stirred for 1 hour. Volatiles were removed under vacuum and the crude product was purified by column chromatography on silica gel (hexane/EtOAc=9/1) to yield the product as a white solid (190.7 mg, 96 %). **^{1}H NMR** (400 MHz, CD_2Cl_2) δ 8.14 (d, *J* = 7.7 Hz, 1H), 8.04 (d, *J* = 7.8 Hz, 1H), 7.93 (d, *J* = 8.4 Hz, 2H), 7.78 – 7.69 (m, 2H), 7.60 (dd, *J* = 8.5, 7.1 Hz, 2H), 7.49 – 7.42 (m, 1H), 7.30 (t, *J* = 7.5 Hz, 1H), 7.18 (dd, *J* = 8.5, 7.3 Hz, 1H), 6.75 (d, *J* = 8.5 Hz, 1H), 1.27 (s, 12H). **^{13}C{^{1}H} NMR** (101 MHz, CD_2Cl_2) δ 172.2, 143.5, 139.9, 136.2, 133.5, 132.9, 130.4, 129.4, 126.8, 126.6, 126.1, 123.8, 123.3, 122.1, 120.6, 114.6, 84.4, 25.5. **^{11}B NMR** (128 MHz, CD_2Cl_2) δ 29.57. **[Acc. Mass]** Calculated $[M+H]^+$: 398.1922 $gmol^{-1}$, Observed $[M+H]^+$: 398.1913 $gmol^{-1}$.

Acyl Directed Borylation of Benzoyl-Protected Aniline: N-(2-(4,4,5,5-tetramethyl-1,3,2-dioxaborolan-2-yl)phenyl)benzamide (13a)

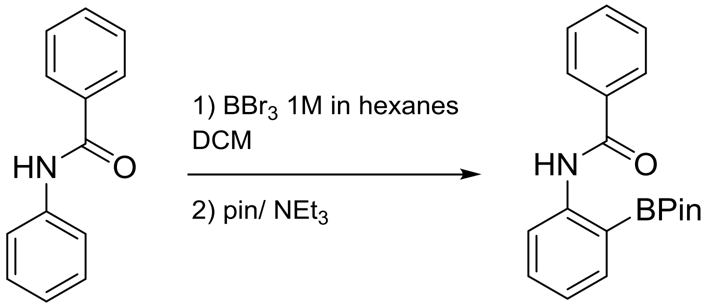

BBr_3 1M in DCM (0.42 mmol, 0.42mL) was added to a stirred solution of benzanilide (0.2 mmol, 39.4 mg) in DCM (3mL). Note, no significant borylation was observed at room temperature after 2 h. The reaction mixture was stirred for 16 hours at 60°C. On completion of borylation, pinacol (0.6 mmol, 71 mg) and NEt_3 (3 mmol, 418 μL) were added to the reaction mixture and stirred for 1 hour. Volatiles were removed under vacuum and the crude product was purified by column chromatography on silica gel (hexane/EtOAc = 4/1) to yield the product as a white solid (44.6 mg, 69 %). **^{1}H NMR** (400 MHz, $CDCl_3$) δ 10.31 (br s, 1H), 8.74 (d, *J* = 8.4 Hz, 1H), 8.04 (d, *J* = 7.4 Hz, 2H), 7.82 (d, *J* = 7.4 Hz, 1H), 7.59 – 7.45 (m, 4H), 7.16 – 7.06 (t, *J* = 7.4 Hz, 1H), 1.41 (s, 12H). **^{13}C{H} NMR** (101 MHz, $CDCl_3$) δ 165.4, 145.1, 136.1, 135.5, 133.2, 131.8, 128.6, 127.4, 123.2, 119.3, 84.7, 25.1. **^{11}B NMR** (128 MHz, $CDCl_3$) δ 30.59. **[Acc. Mass]** Calculated $[M+H]^+$: 324.1766 $gmol^{-1}$, Observed $[M+H]^+$: 324.1753 $gmol^{-1}$

Synthesis of N-pivaloylanilines: General procedure 4

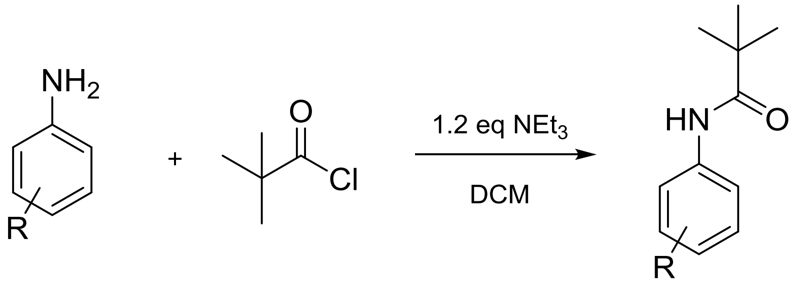

To a stirring solution of the aniline (10 mmol) and Et_3N (1.673 mL, 12 mmol) in DCM (30 mL) was added dropwise pivaloyl chloride (11 mmol) at 0 °C (ice/water bath) for 10 min. Then, the reaction mixture was stirred at room temperature overnight. HCl 1M (20 mL) was added to the crude mixture and the reaction mixture was extracted with DCM (20 mL × 3), washed with brine (20 mL × 3), and dried over anhydrous $MgSO_4$. Then, the extract was concentrated and recrystallized from a hexane/DCM mixture to afford the amide.
Analytical data for the aniline substrates are in accordance with the literature.[9]

Acyl-Directed Borylation of Pivaloyl-Protected Anilines: General procedure 5

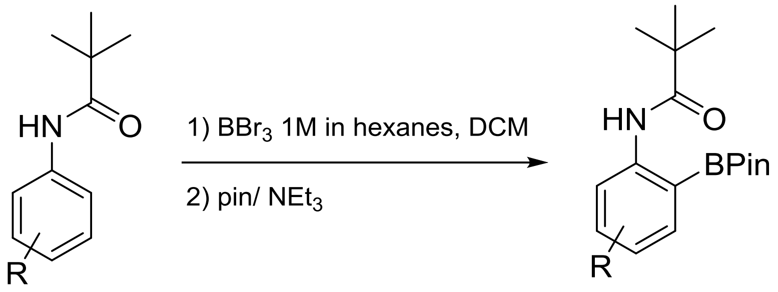

BBr_3 1M in DCM (amounts provided below) was added to a stirred solution of pivaloyl-protected aniline in DCM (3mL). After the addition, the reaction mixture was stirred at room temperature until consumption of the pivaloyl-protected aniline (times given below). On completion of borylation, pinacol (0.6 mmol, 71 mg) and NEt_3 (3 mmol, 418 mL) were added to the reaction mixture and stirred for 1 hour. Volatiles were removed under vacuum and the crude product was purified by column chromatography on silica gel (hexane/EtOAc) to yield the product as a solid.

N-(2-(4,4,5,5-tetramethyl-1,3,2-dioxaborolan-2-yl)phenyl)pivalamide (13b)

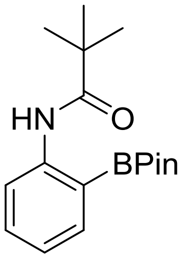

Compound **13b** was prepared following **general procedure 5** using BBr_3 1M in DCM (0.42 mmol, 0.42 mL) and pivaloyl-protected aniline (0.2 mmol, 35.5 mg). The reaction mixture was stirred at room temperature for 16 h. The product was purified by column chromatography on silica gel (hexane/EtOAc=6/1) to yield the product as white solid (39 mg, 64 %). **^{1}H NMR** (400 MHz, CD_2Cl_2) δ 9.51 (br s, 1H), 8.52 (d, *J* = 8.4 Hz, 1H), 7.75 (dd, *J* = 7.4, 1.8 Hz, 1H), 7.43 (ddd, *J* = 8.7, 7.2, 1.8 Hz, 1H), 7.04 (t, *J* = 7.4 Hz, 1H), 1.38 (s, 12H), 1.31 (s, 9H). **^{13}C {^{1}H} NMR** (101 MHz, CD_2Cl_2) δ 177.5, 146.0, 136.7, 133.2, 122.9, 119.5, 85.0, 40.6, 27.9, 25.3. **^{11}B NMR** (128 MHz, CD_2Cl_2) δ 30.45. **[Acc. Mass]** Calculated $[M+H]^+$: 304.2079 $gmol^{-1}$, Observed $[M+H]^+$: 304.2065 $gmol^{-1}$

N-(2-methyl-6-(4,4,5,5-tetramethyl-1,3,2-dioxaborolan-2-yl)phenyl)pivalamide (13c)

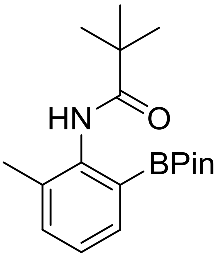

Compound **13c** was prepared following **general procedure** 5 using BBr_3 1M in DCM (1.05 mmol, 1.05mL) and pivaloyl-protected aniline (0.5 mmol, 95.6 mg). The reaction mixture was stirred at room temperature for 16 h. The product was purified by column chromatography on silica gel (hexane/EtOAc=3/1) to yield the product as a white solid (112 mg, 71 %). **^{1}H NMR** (400 MHz, CD_2Cl_2) δ 8.20 (br s, 1H), 7.56 (d, *J* = 7.4 Hz, 1H), 7.27 (d, *J* = 7.4 Hz, 1H), 7.14 (t, *J* = 7.4 Hz, 1H), 2.20 (s, 3H), 1.35 (s, 9H), 1.32 (s, 12H). **^{13}C{^{1}H} NMR** (101 MHz, CD_2Cl_2) δ

177.2, 140.8, 133.4, 132.1, 125.9, 83.7, 39.7, 27.8, 25.6, 18.6. **^{11}B NMR** (128 MHz, CD_2Cl_2) δ 26.06. **[Acc. Mass]** Calculated $[M+H]^+$: 318.2235 $gmol^{-1}$, Observed $[M+H]^+$: 318.2228 $gmol^{-1}$

N-(5-bromo-2-(4,4,5,5-tetramethyl-1,3,2-dioxaborolan-2-yl)phenyl)pivalamide (13d)

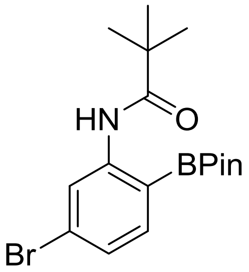

Compound **13d** was prepared following **general procedure 5** using BBr_3 1M in DCM (1.05 mmol, 1.05mL) and pivaloyl-protected aniline (0.5 mmol, 128.1 mg). The reaction mixture was stirred at room temperature for 16 h. The product was purified by column chromatography on silica gel (hexane/EtOAc=9/1) to yield the product as a white solid (166.1 mg, 87 %). **^{1}H NMR** (400 MHz, CD_2Cl_2) δ 9.57 (br s, 1H), 8.82 (d, *J* = 1.9 Hz, 1H), 7.61 (d, *J* = 8.0 Hz, 1H), 7.19 (dd, *J* = 8.0, 1.8 Hz, 1H), 1.38 (s, 12H), 1.31 (s, 9H). **^{13}C{^{1}H} NMR** (101 MHz, CD_2Cl_2) δ 177.6, 146.9, 137.8, 127.8, 126.0, 122.4, 85.3, 40.6, 27.8, 25.2. **^{11}B NMR** (128 MHz, CD_2Cl_2) δ 30.42. **[Acc. Mass]** Calculated $[M+H]^+$: 382.1184 $gmol^{-1}$, Observed $[M+H]^+$: 382.1178 $gmol^{-1}$

N-(4-methyl-2-(4,4,5,5-tetramethyl-1,3,2-dioxaborolan-2-yl)phenyl)pivalamide (13e)

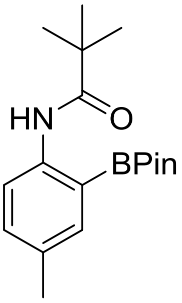

Compound **13e** was prepared following **general procedure 5** using BBr_3 1M in DCM (0.42 mmol, 0.42 mL) and pivaloyl-protected aniline (0.2 mmol, 38.3 mg). The reaction mixture was stirred at room temperature for 3 h. The product was purified by column chromatography on silica gel (hexane/EtOAc=4/1) to yield the product as a white solid (53.8 mg, 85 %)**.** **^{1}H NMR** (400 MHz, CD_2Cl_2) δ 9.44 (s, 1H), 8.40 (d, *J* = 8.5 Hz, 1H), 7.56 (d, *J* = 2.3 Hz, 1H), 7.25 (dd, *J* = 8.3, 2.3 Hz, 1H), 2.30 (s, 3H), 1.38 (s, 12H), 1.31 (s, 9H).**^{13}C{^{1}H} NMR** (101 MHz, CD_2Cl_2) δ 177.2, 143.5, 137.0, 133.7, 132.3, 119.5, 84.9, 40.5, 28.0, 25.3, 20.9. **^{11}B NMR** (128 MHz, CD_2Cl_2) δ 30.39. **[Acc. Mass]** Calculated $[M+H]^+$: 318.2235 $gmol^{-1}$, Observed $[M+H]^+$: 318.2230 $gmol^{-1}$

N-methyl-N-(2-(4,4,5,5-tetramethyl-1,3,2-dioxaborolan-2-yl)phenyl)pivalamide (13f)

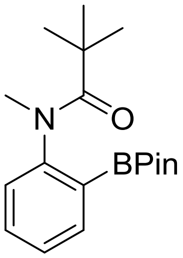

Compound **13**f was prepared following **general procedure 5** using BBr_3 1M in DCM (0.42 mmol, 0.42mL) and pivaloyl-protected aniline (0.2 mmol, 38.3 mg). The reaction mixture was stirred at 60 °C for 6 h. The product was purified by column chromatography on silica gel (hexane/EtOAc=6/1) to yield the product as a colorless solid (52.6 mg, 83 %). **^{1}H NMR** (400 MHz, CD_2Cl_2) δ 7.80 (dd, *J* = 7.4, 1.7 Hz, 1H), 7.45 (td, *J* = 7.6, 1.8 Hz, 1H), 7.32 (t, *J* = 7.4 Hz, 1H), 7.17 (d, *J* = 7.8 Hz, 1H), 3.32 – 2.99 (br s, 3H), 1.30 (s, 12H), 0.99 (br s, 9H). **^{13}C{^{1}H} NMR** (101 MHz, CD_2Cl_2) δ 177.1, 151.1, 136.9, 132.2, 129.7, 127.5, 84.2, 41.6, 41.1, 29.9, 25.2. **^{11}B NMR** (128 MHz, CD_2Cl_2) δ 30.33. **[Acc. Mass]** Calculated $[M+H]^+$: 318.2227 $gmol^{-1}$, Observed $[M+H]^+$: 318.2235 $gmol^{-1}$

Synthesis of C5 borylation starting material

1-benzyl-4-nitro-1H-indole

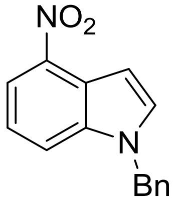

To a suspension of 60% NaH in mineral oil (0.660 g, 16.5 mmol) in DMF (15 mL) was added a solution of 4-nitroindole (2.430 g, 15 mmol) in DMF (15 mL). Benzyl bromide (1.96 mL, 16.5 mmol) was added and the reaction mixture was stirred overnight at room temperature. The reaction was quenched with saturated $NH_4Cl_{(aq)}$, extracted with DCM and dried over Magnesium sulfate. Purification by column chromatography on silica gel (EtOAc: Petroleum ether) gave the pure product (2.573 g, 62%) as a yellow solid. **^{1}H NMR** (500 MHz, $CDCl_3$) δ 8.14 (d, J = 7.9 Hz, 1H), 7.59 (d, J = 8.1 Hz, 1H), 7.40 (d, J = 3.1 Hz, 1H), 7.36 – 7.27 (m, 4H), 7.23 (t, J = 8.0 Hz, 1H), 7.10 (d, J = 6.6 Hz, 2H), 5.41 (s, 2H). **^{13}C{H} NMR** (126 MHz, $CDCl_3$) δ 140.8, 138.5, 136.5, 132.9, 129.2, 128.3, 126.8, 123.1, 120.8, 117.8, 116.7, 102.6, 50.8. This compound was prepared following an adapted procedure.[10]

1-benzyl-1H-indol-4-amine

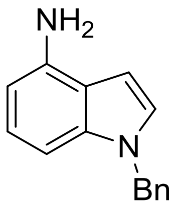

To a solution of **1-benzyl-4-nitro-1H-indole** (2.002 g, 7.935 mmol) in THF (40 mL) was added acetic acid (27 mL) and the mixture was stirred for 15 minutes at room temperature. Zinc (6.800 g, 104.01 mmol) was added followed by the slow addition of concentrated $HCl_{(aq.)}$ at 0 C. The reaction mixture was stirred for 2 h at room temperature. NaOH in MeOH was slowly added to basify the solution. The product was extracted with EtOAc and washed with brine. The organic fractions were dried over magnesium sulfate and purified by column chromatography on silica gel (EtOAc: Hexane) to give the product (0.638 g, 36%) as a dark brown solid. **^{1}H NMR** (500 MHz, $CDCl_3$) δ 7.32 – 7.22 (m, 3H), 7.14 – 7.09 (m, 2H), 7.03 (d, J = 3.2 Hz, 1H), 7.02 – 6.97 (m, 1H), 6.76 (d, J = 8.2 Hz, 1H), 6.47 (dd, J = 3.2, 0.8 Hz, 1H), 6.42 – 6.39 (m, 1H), 5.28 (s, 2H), 3.93 (s, 2H). **^{13}C{H} NMR** (126 MHz, $CDCl_3$) δ 139.7, 137.8, 137.6, 128.9, 127.7, 127.0, 126.6, 123.1, 118.1, 104.2, 101.1, 98.1, 50.4. Analytical data are in accordance with the literature.[10]

N-(1-benzyl-1H-indol-4-yl)pivalamide (14)

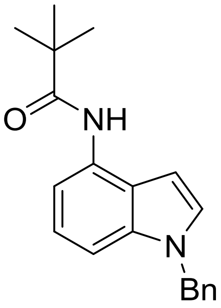

To a solution of 1-benzyl-4-NH_2-1H-indole (0.524 g, 2.360 mmol) in DCM (4.7 mL) was added NEt_3 (0.36 mL, 2.596 mmol). The reaction mixture was stirred at 0 °C for 10 minutes. Pivaloyl chloride (0.30 mL, 2.478 mmol) was added dropwise and the solution was stirred at 0 °C for 10 minutes after which it was allowed to warm to room temperature and stirred overnight. 1M $HCl_{(aq).}$ (10 mL) was added and the product was extracted with DCM. The organic layers were combined and washed with saturated $NaHCO_{3(aq)}$ and dried over magnesium sulfate. Purification by column chromatography on silica gel gave the pure product (0.556 g, 77%) as a grey solid. **^{1}H NMR** (500 MHz, $CDCl_3$) δ 7.83 (d, *J* = 7.7 Hz, 1H), 7.64 (s, 1H), 7.31 – 7.24 (m, 3H), 7.16 (t, *J* = 8.0 Hz, 1H), 7.13 (d, *J* = 3.3 Hz, 1H), 7.09 – 7.05 (m, 3H), 6.43 (dd, *J* = 3.2, 0.9 Hz, 1H), 5.33 (s, 2H), 1.39 (s, 9H). **^{13}C{H} NMR** (126 MHz, $CDCl_3$) δ 176.5, 137.4, 137.2, 130.4, 128.9, 127.91, 127.85, 126.9, 122.8, 120.8, 111.4, 106.5, 97.3, 50.4, 40.0, 27.9. **[Acc. Mass]** Calculated $[M+H]^+$: 307.1805 $gmol^{-1}$, Observed $[M+H]^+$: 307.1806 $gmol^{-1}$.

C5 borylation: 5-(4,4,5,5-tetramethyl-1,3,2-dioxaborolan-2-yl)N-(1-benzyl-1H-indol-4-yl)pivalamide (16)

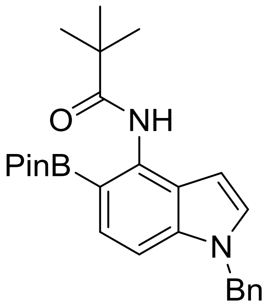

To a solution of N-(1-benzyl-1H-indol-4-yl)pivalamide(20) (0.030 g, 0.1 mmol) in DCM (0.3 mL) was added BBr_3 1M in heptanes (0.2 mL, 0.2 mmol). The mixture was stirred for 3 h at room temperature after which pinacol 1M in NEt_3 (0.35 mL, 0.35 mmol) was added and the mixture was stirred overnight at room temperature. The solvent was removed in *vacuo* (crude NMR yield – 77% with mesitylene as internal standard) and the product purified by column chromatography on silica-gel (Hexane: EtOAc) to give the product (0.017 g, 40%) as an orange oil. Note this compound is sensitive to protodeboronation in wet solvents reforming **14**. **^{1}H NMR** (500 MHz, $CDCl_3$) δ 9.07 (s, 1H), 7.56 (d, *J* = 8.3 Hz, 1H), 7.30 – 7.19 (m, 3H), 7.09 – 7.04 (m, 4H), 6.55 – 6.52 (m, 1H), 5.31 (s, 2H), 1.41 (s, 9H), 1.36 (s, 12H). **^{13}C{H} NMR** (126 MHz, $CDCl_3$) δ 176.5, 140.0, 137.8, 137.6, 129.0, 128.8, 127.7, 127.4, 126.8, 122.6, 106.8, 103.8, 83.7, 50.2, 39.8, 27.8, 25.2. **^{11}B NMR** (160 MHz, $CDCl_3$) δ 30.53. **[Acc. Mass]** Calculated $[M-H]^+$: 431.2502 $gmol^{-1}$, Observed $[M-H]^+$: 431.2502 $gmol^{-1}$.

N-(1-pivaloyl-1H-indol-4-yl)pivalamide (17)

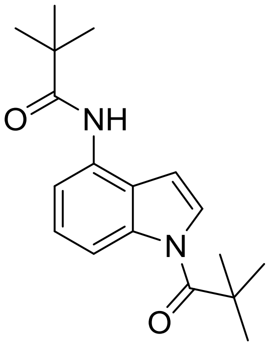

To an ampule containing 4-NH_2-indole (0.661 g, 5 mmol) and 4-DMAP (0.061 g, 0.5 mmol) was added DCM (10 mL) and NEt_3 (1.5 mL). The solution was cooled to 0 °C and pivaloyl chloride (1.23 mL, 10 mmol) was added. The mixture was stirred at this temperature for 10 minutes after which it was warmed to room temperature and stirred overnight. The solvent was removed *in vacuo* and the crude product was dissolved in DCM and was washed with 1M HCl, sat. $NaHCO_3$ (aq.), sat. $NH_4Cl_{(aq)}$ and brine. The organic fraction was dried over Magnesium sulfate and purified by column chromatography on silica gel (Hexane: EtOAc) to give the pure product (0.853 g, 57%) as a white solid. **^{1}H NMR** (500 MHz, $CDCl_3$) δ 8.31 (dt, *J* = 8.5, 0.7 Hz, 1H), 7.73 (d, *J* = 3.9 Hz, 1H), 7.70 (d, *J* = 7.8 Hz, 1H), 7.53 (s, 1H), 7.32 (t, *J* = 8.1 Hz, 1H), 6.51 (dd, *J* = 3.9, 0.7 Hz, 1H), 1.52 (s, 9H), 1.39 (s, 9H). **^{13}C{H} NMR** (126 MHz, $CDCl_3$) δ 177.2, 176.7, 137.6, 129.7, 126.0, 125.4, 122.3, 116.7, 114.4, 104.4, 41.5, 39.9, 28.8, 27.9. **[Acc. Mass]** Calculated $[M+H]^+$: 301.1911 $gmol^{-1}$, Observed $[M+H]^+$: 301.1909 $gmol^{-1}$.

C5/C7 double borylation: N-(5,7-bis(4,4,5,5-tetramethyl-1,3,2-dioxaborolan-2-yl)-1H-indol-4-yl)pivalamide (18)

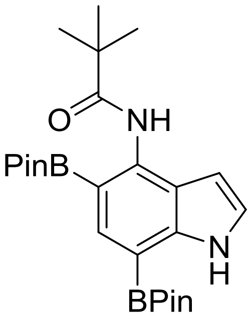

To a solution of compound **17** (0.030 g, 0.1 mmol) in DCM (0.3 mL) was added BBr_3 1M in heptanes (0.32 mL, 0.32 mmol). The reaction mixture was stirred at 60 °C for 16 h. Pinacol 1M in NEt_3 (0.45 mL, 0.45 mmol) was added followed by DCM (0.8 mL) and the reaction mixture was stirred at room temperature for 16 h. 'Wet' MeOH (1 mL) was added and the solution was stirred at 60 °C for a further 16 h. The solvent was removed in *vacuo* (crude NMR yield – 72% with mesitylene internal standard) and was purified by column chromatography on silica gel (Hexane: DCM→Hexane: EtOAc) to give the pure product (0.017g, 36%) as a white solid. **^{1}H NMR** (400 MHz, $CDCl_3$) δ 9.28 (s, 1H), 9.25 (s, 1H), 8.08 (s, 1H), 7.18 (dd, *J* = 3.2, 2.3 Hz, 1H), 6.54 (dd, *J* = 3.3, 2.3 Hz, 1H), 1.41 (s, 9H), 1.38 (s, 12H), 1.36 (s, 12H). **^{11}B NMR** (128 MHz, $CDCl_3$) δ 31.29. **^{13}C{H} NMR** (101 MHz, $CDCl_3$) δ 176.5, 145.1, 140.9, 137.6, 122.8, 120.3, 104.3, 83.8, 83.8, 40.0, 27.8, 25.13, 25.12. **[Acc. Mass]** Calculated $[M+H]^+$: 469.3048 $gmol^{-1}$, Observed $[M+H]^+$: 469.3041 $gmol^{-1}$.

Compound 19: C3/C7 boracycle

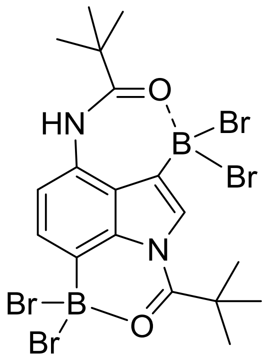

In a J-Youngs NMR tube, to a solution of **17** (0.030 g, 0.1 mmol) in DCM (0.3 mL) was added BBr_3 1M in heptanes (0.32 mL, 0.32 mmol). The reaction mixture was heated at 60 °C for 16 h. The volatiles (DCM, BBr_3) were removed under vacuum and dry $CDCl_3$ was added for NMR analysis. **^{1}H NMR** (500 MHz, $CDCl_3$) δ 9.54 (s, 1H), 8.94 (d, *J* = 8.6 Hz, 1H), 8.13 (d, *J* = 8.6 Hz, 1H), 1.75 (s, 9H), 1.54 (s, 9H). **^{13}C{H} NMR** (126 MHz, $CDCl_3$) δ 207.7, 173.2, 148.4, 145.2, 133.2, 129.1, 123.2, 113.0, 44.5, 40.0, 30.0, 27.6.

References

1. Z. Yan, W. Chen, Y. Gao, S. Mao, Y. Zhang, Y. Wang, *Adv. Synth. Catal*. **2014**, 356, 1085–1092
2. J. Cornella, P. Lu, I. Larrosa., *Org. Lett*. **2009**, 11, 23, 5506-5509
3. Y. Kim, J. Park, S. Chang, *Org. Lett*., **2016**, 18, 8, 1892-1895
4. D. R Stuart, E. Villemure, K. Fagnou., *J. Am. Chem. Soc*. **2007**, 129, 40, 12072-12073
5. Y. Yang, R. Li, Y. Zhao, D. Zhao, Z. Shi, *J. Am. Chem. Soc*., **2016**, 138, 28, 8734-8737
6. D. W. Robbins, T. A. Boebel, J. F. Hartwig, *J. Am. Chem. Soc*., **2010**, 132, 4068-4069
7. A. Ahmed, H. S. Dutta, B. Khan, R. Kant, D. Koley, *Adv. Synth. Catal*., **2018**, 360, 1644–1649
8. S. Cai, H. Shi, J. Li, L. Gu, Y. Ni, Z. Cheng, S. Wang, W. Xiong, L. Li, Z. An, W. Huang, *Adv. Mater*., **2017**, 29, 1701244
9. C. Mei, W. Lu, *J. Org. Chem*, **2018**, 83, 4812-4823
10. M. Tsuchiya, M. Somei, *Chem. Pharm. Bull*., **1981**, 29, 3145-3157

C3 vs C7 borylation

Substrate **2a** was borylated at the C3 position by reacting with BBr_3 (2 eq.) + 2,6-lutidine (2 eq.), a combination which forms a borenium in-situ, in DCM followed by pinacol protection using Et_3N/Pinacol. The ^1H NMR spectrum of the crude BPin product (shown below) is considerably different to the C7 borylated product **5a**. Note the spectra for the C3-BPin indole was obtained as the crude product (due to its sensitivity to protodeboronation). The formulation as the C3 borylated product is further supported by the fact that the C2 BPin isomeric product give a diagnostic singlet around 7.1 ppm for the C3-H resonance thus can be precluded. Inset is the crude ^{11}B NMR spectrum which confirms the presence of a (heteroaryl)BPin species (30.2 ppm) and a $B(OR)_3$ species (22.4 ppm) the latter is due to the reaction of pinacol with excess BBr_3.

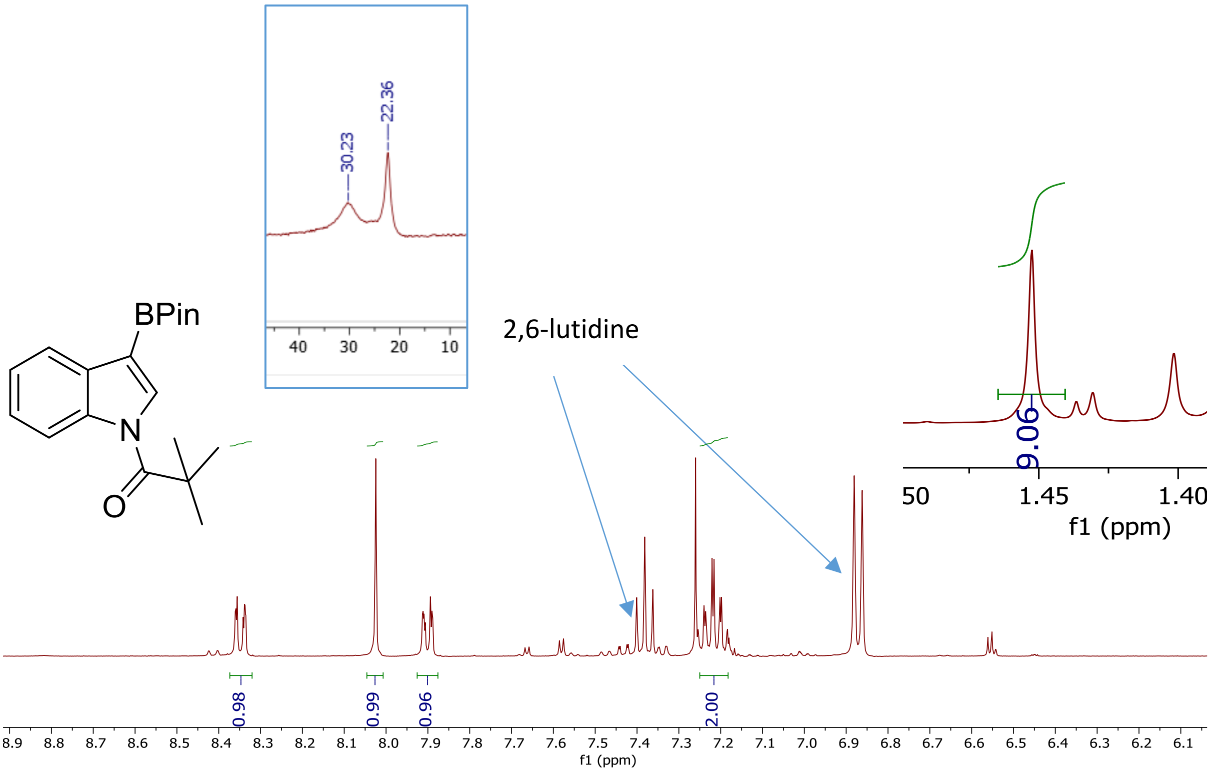

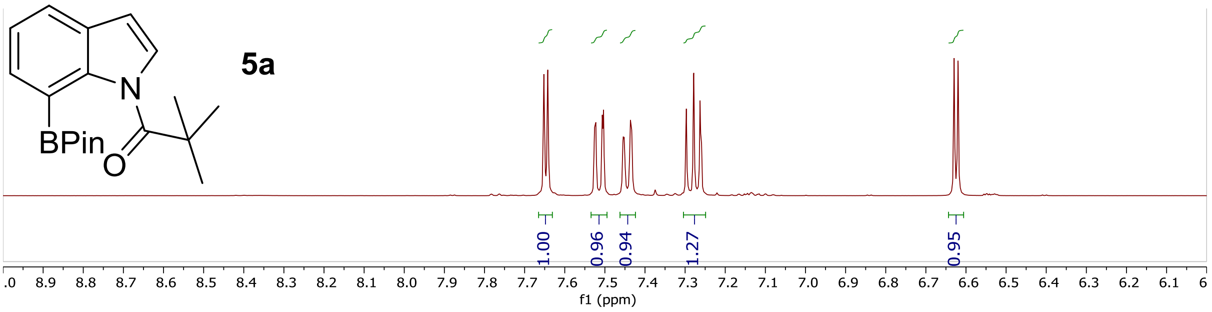

2D NMR data: **5-(4,4,5,5-tetramethyl-1,3,2-dioxaborolan-2-yl)N-(1-benzyl-1H-indol-4-yl)pivalamide (16). <u>^{1}H COSY (top), ^{1}H NOESY (bottom)</u>**. Note: Dotted lines are used to illustrate the interaction between the C2-H and C3-H in the C5 borylated product. This combined with the small $^{3}J_{H-H}$ coupling of 3.2 Hz observed between C2-H and C3-H confirms product identification as the C5 borylated.

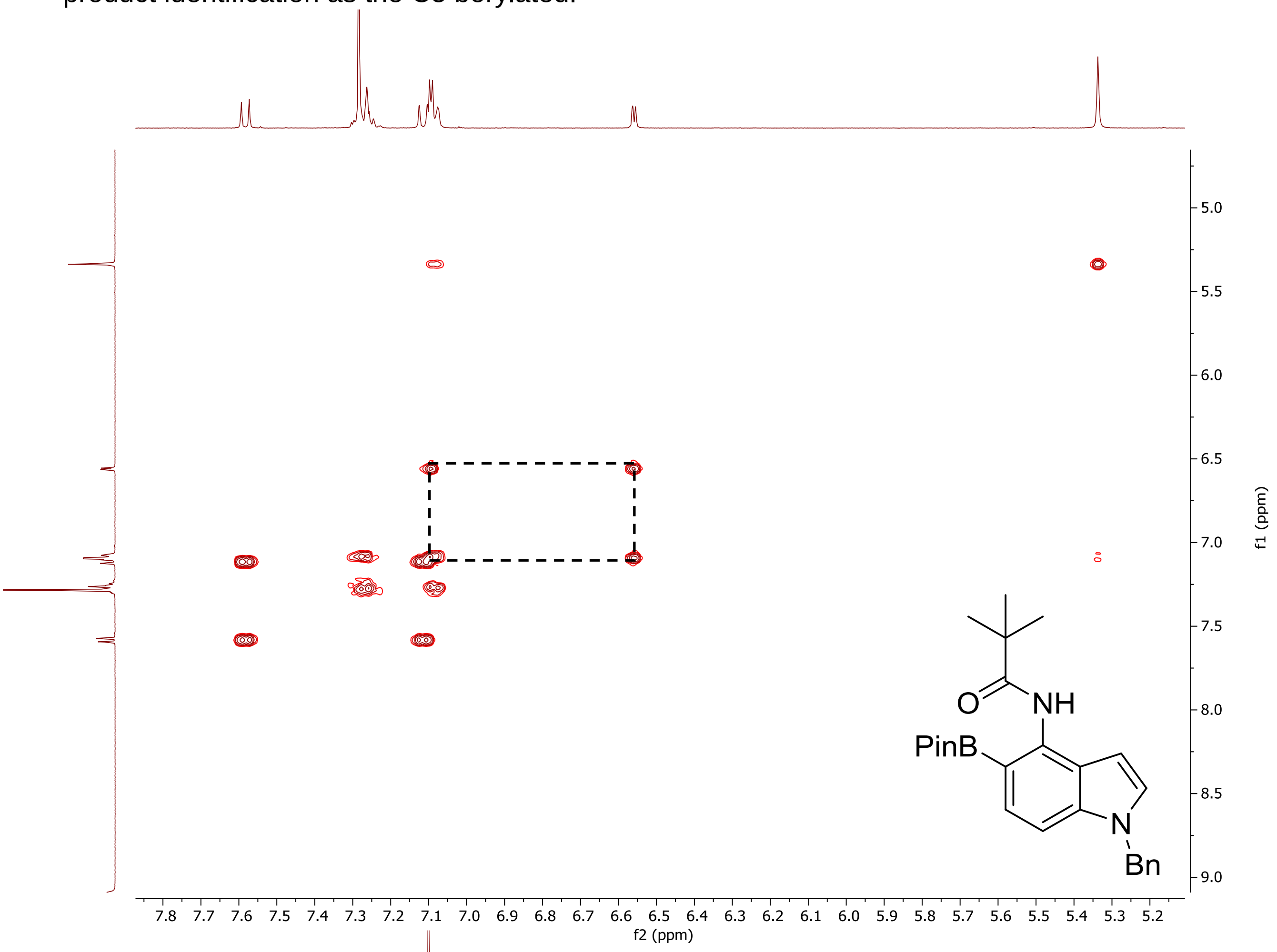

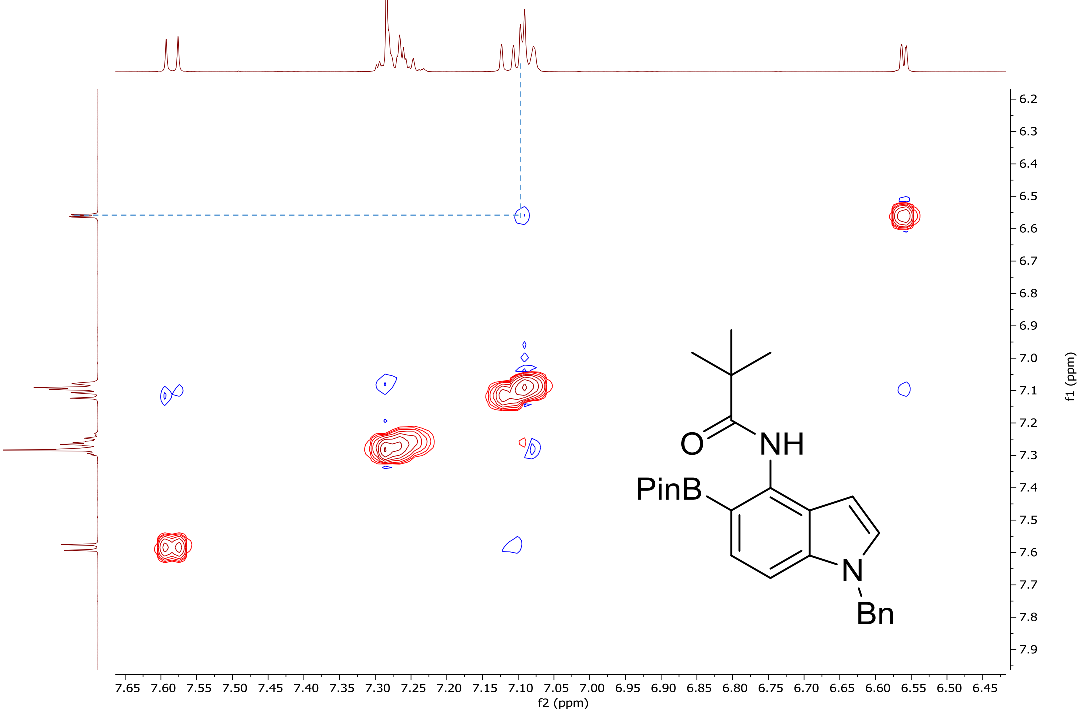

^{13}C HSQC (top), ^{13}C HMBC (bottom)

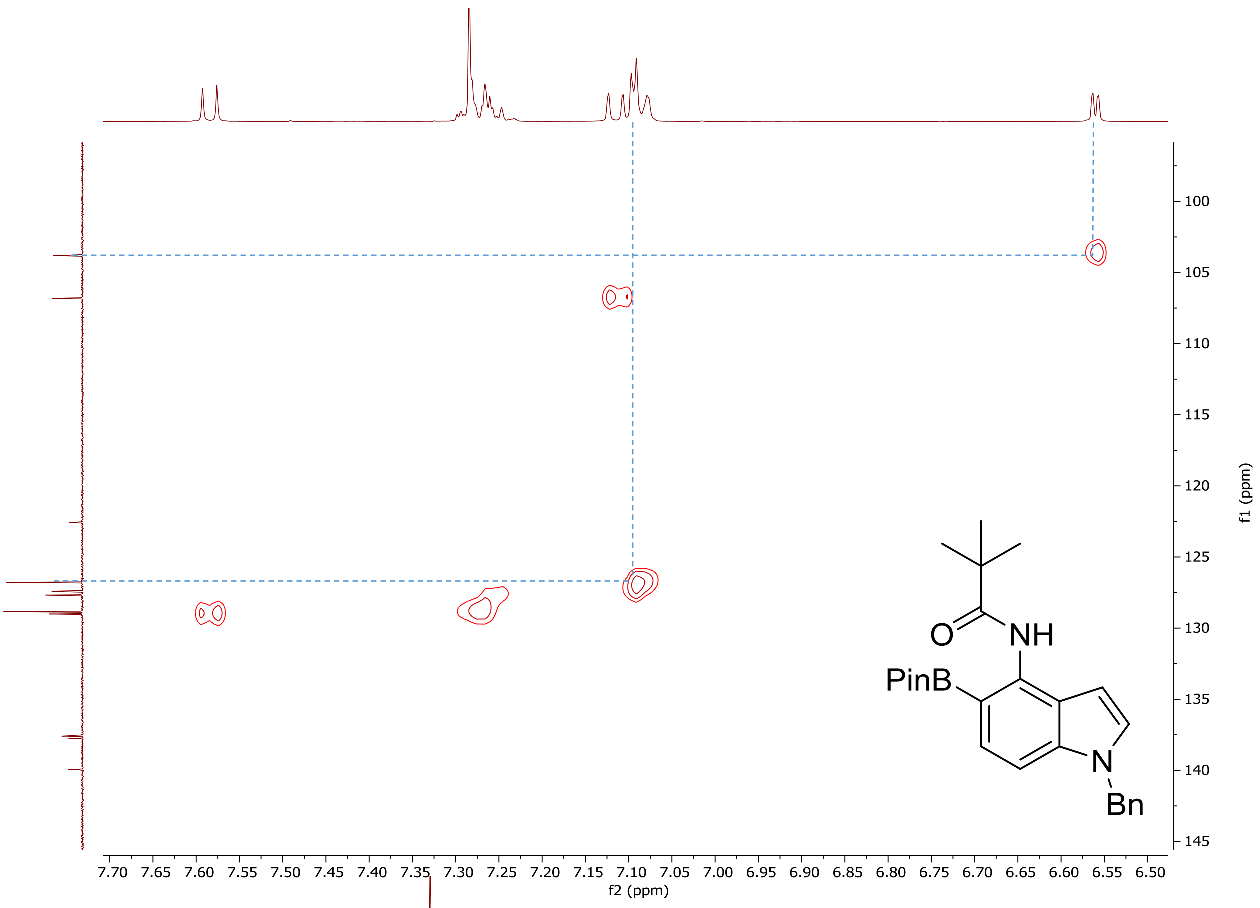

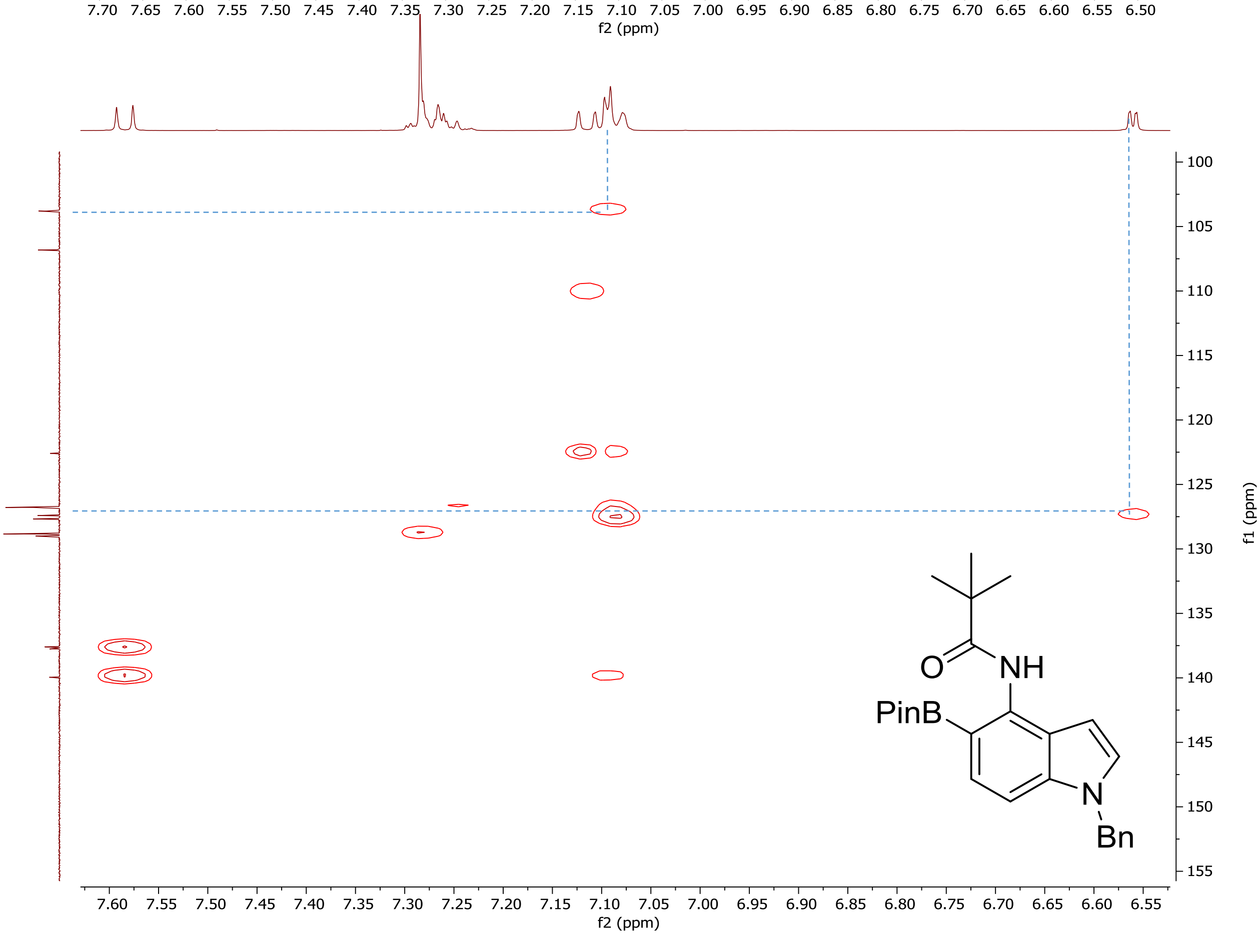

<u>In-situ NMR spectra</u>

The following table and ^{1}H NMR spectra (in DCM) are presented to show the ratio of C7:C2 borylation products in the substrates tested (in-situ with no workup). Note: these ^{1}H NMR spectra depict the intermediate BBr_2 compounds (e.g. **A**/**B**) and not the final pinacol-protected compounds. The diagnostic singlet observed between ca. 6.8-7.1 ppm in the ^{1}H NMR spectrum is for the C2 borylated product (and represents the C3-H resonance in this product)

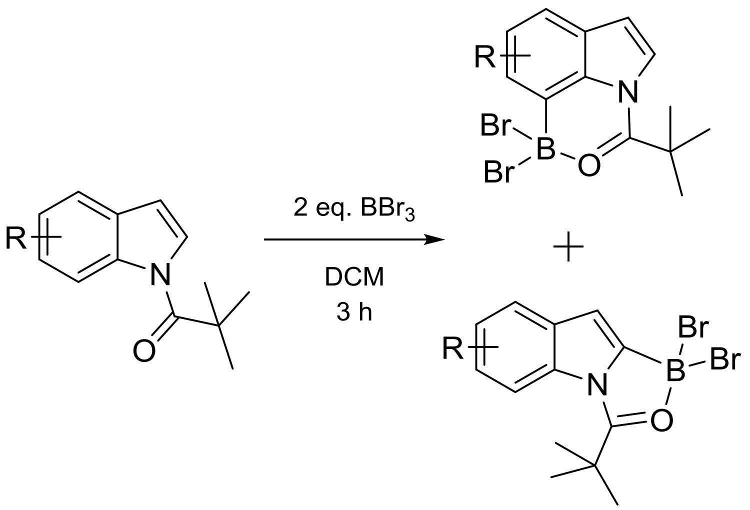

R	Ratio (C7: C2)
H	85:15
5-Me	92:8
6-Me	99:1
4-F	100:0
6-F	71:29
6-OR	100:0
5-I	81:19
6-Cl	56:44
4-Br	100:0
6-Br	57:43
6-tolyl	35:65

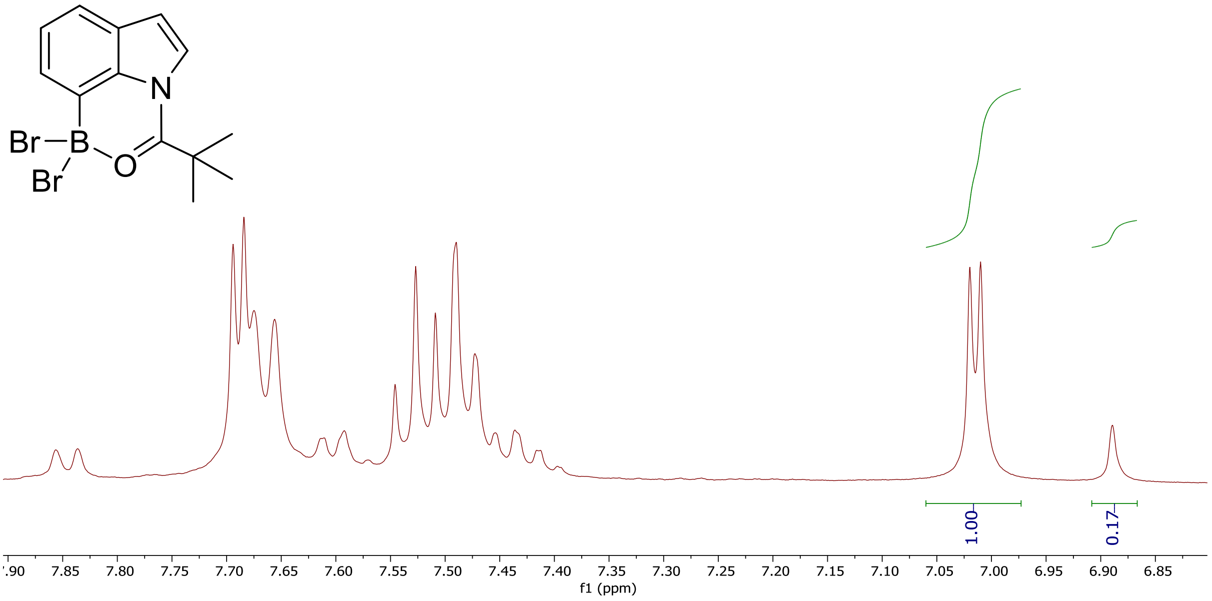

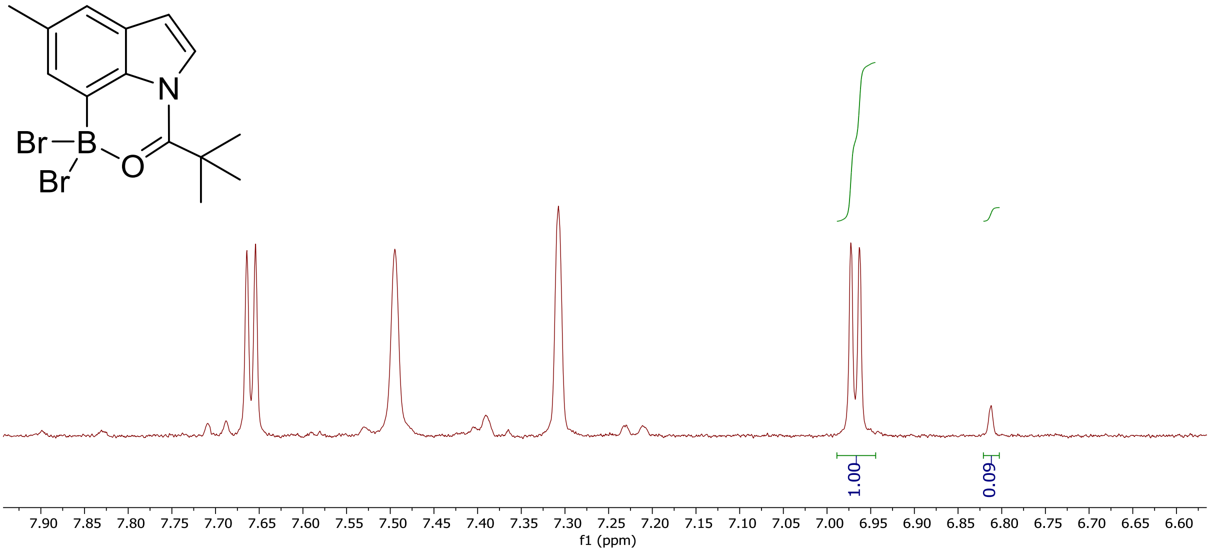

Br
B
Br
O
N
1.00
0.09
7.90 7.85 7.80 7.75 7.70 7.65 7.60 7.55 7.50 7.45 7.40 7.35 7.30 7.25 7.20 7.15 7.10 7.05 7.00 6.95 6.90 6.85 6.80 6.75 6.70 6.65 6.60
f1 (ppm)

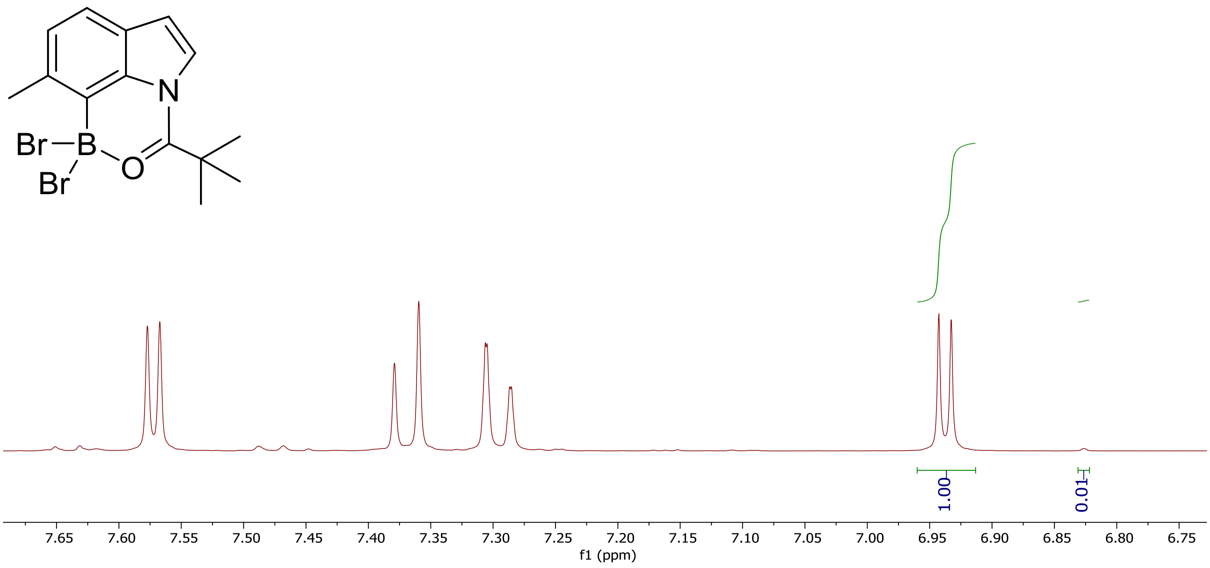

Br
B
Br
O
N
1.00
0.01
7.65 7.60 7.55 7.50 7.45 7.40 7.35 7.30 7.25 7.20 7.15 7.10 7.05 7.00 6.95 6.90 6.85 6.80 6.75
f1 (ppm)

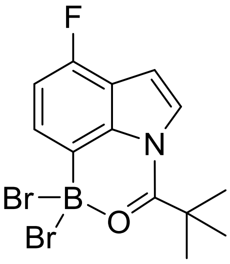

No C2 product
observed

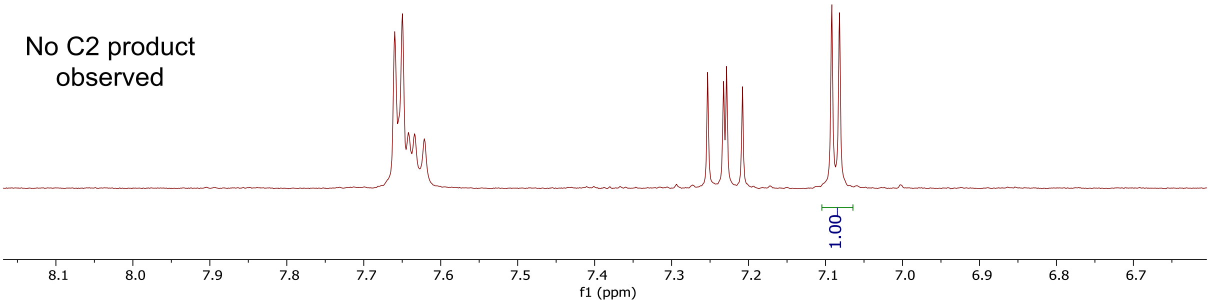

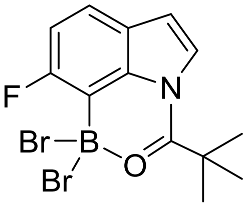

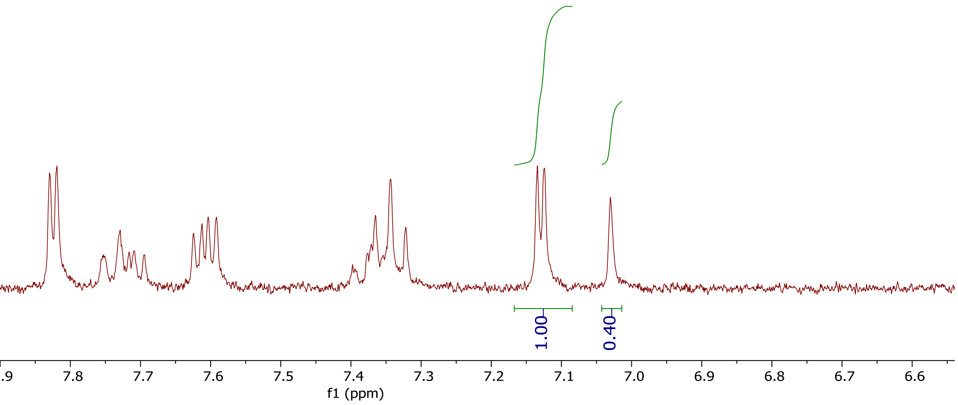

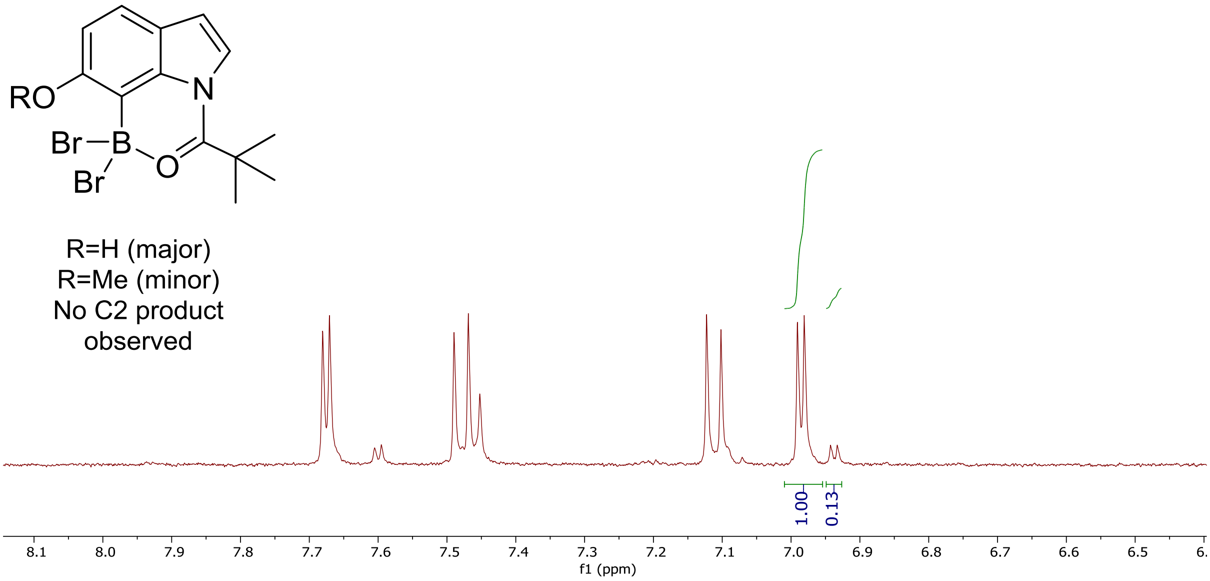
RO
N
Br
B
O
Br
R=H (major)
R=Me (minor)
No C2 product
observed
1.00
0.13
8.1 8.0 7.9 7.8 7.7 7.6 7.5 7.4 7.3 7.2 7.1 7.0 6.9 6.8 6.7 6.6 6.5
f1 (ppm)

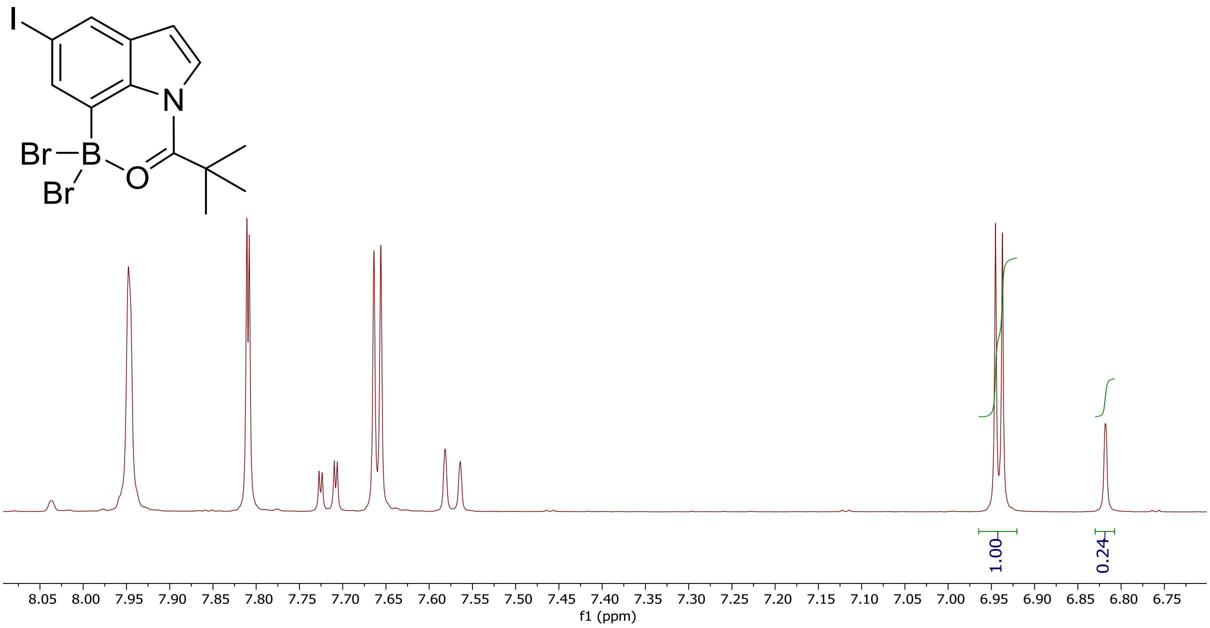
I
N
Br
B
O
Br
1.00
0.24
8.05 8.00 7.95 7.90 7.85 7.80 7.75 7.70 7.65 7.60 7.55 7.50 7.45 7.40 7.35 7.30 7.25 7.20 7.15 7.10 7.05 7.00 6.95 6.90 6.85 6.80 6.75
f1 (ppm)

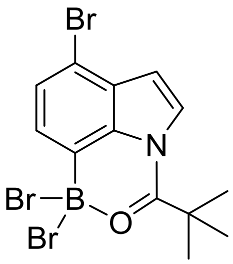

No C2 product observed

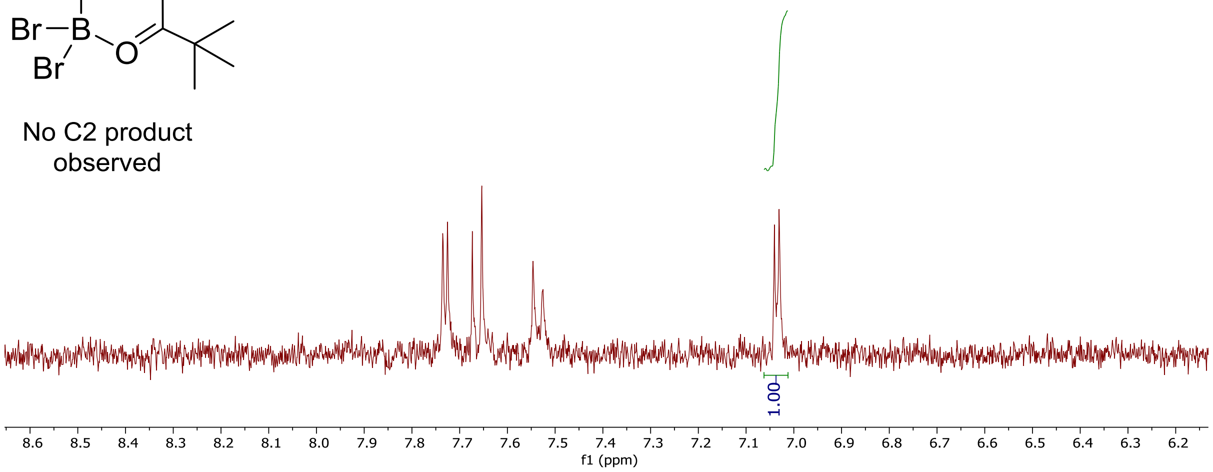

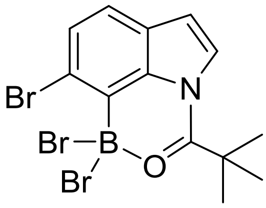

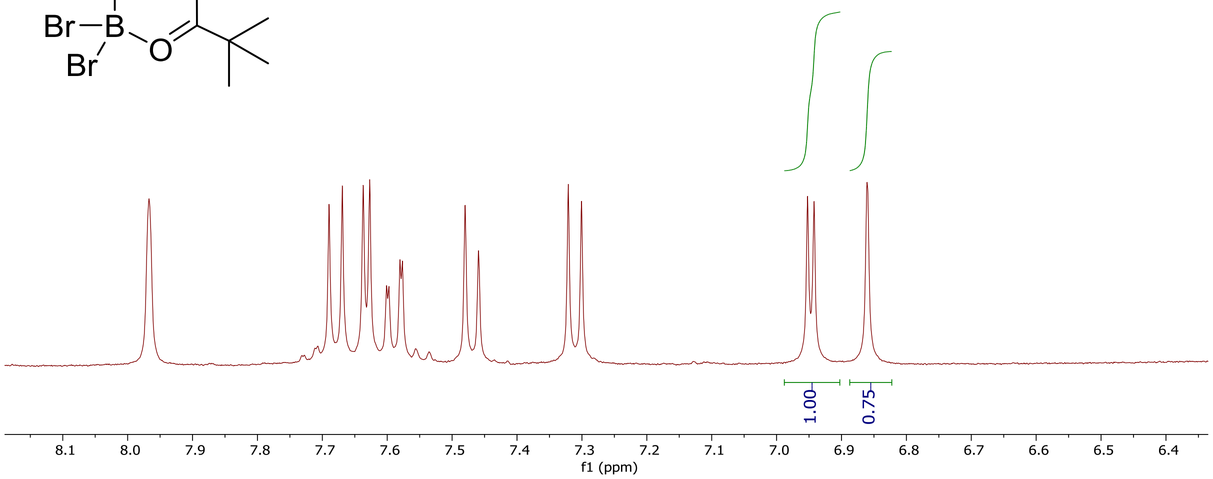

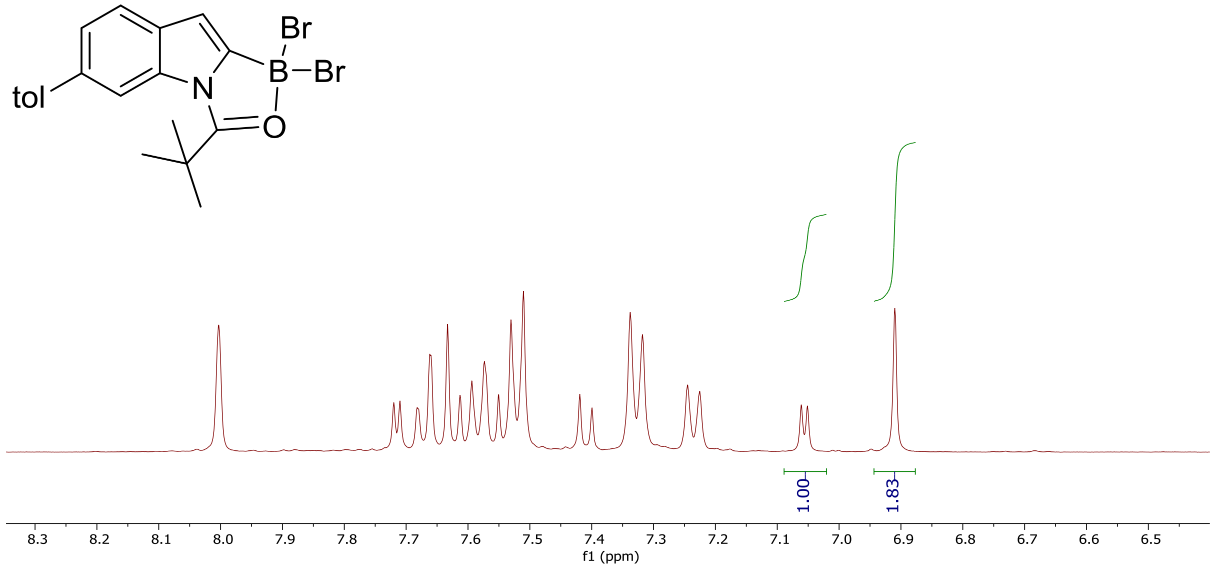

Switching of selectivity after addition of pinacol

The 6-Chloro substrate is shown below as it is the most dramatic shift in the amount of C7-B product, it produced an isolated yield significantly higher than that possible from the in-situ BBr_2 stage ^1H NMR spectra. The mixture of products seen in the in-situ NMR spectrum isomerises to the C7 product upon addition of pinacol as minimal protodeboronation product is formed (little recovered starting material) and the product is isolated in 75% yield

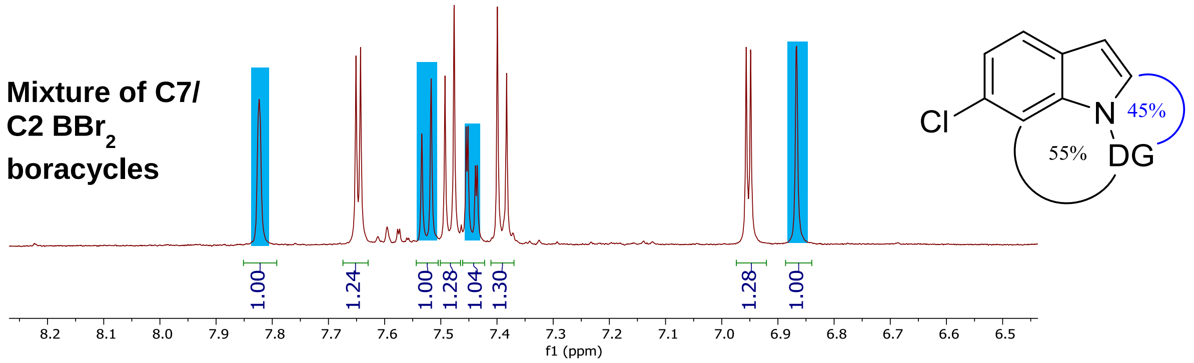

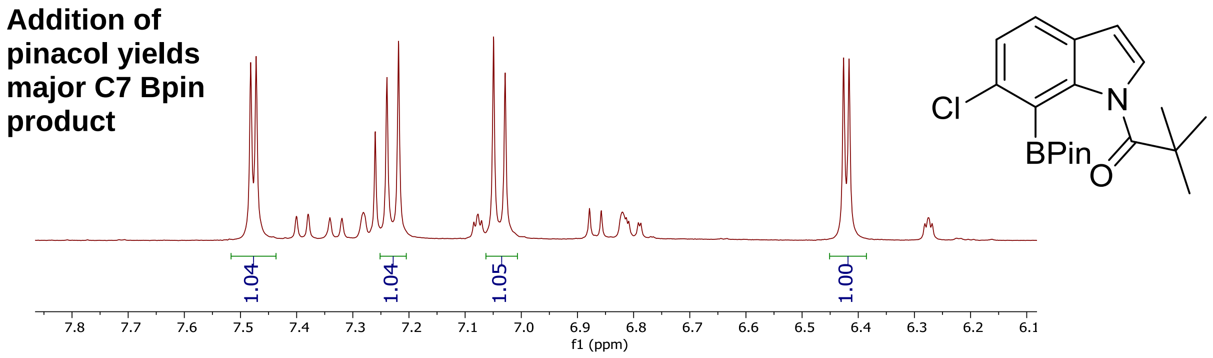

Formation of Compound 19:

The borylation of compound **17** produced two doublet peaks in the aromatic region (at the BBr_2 stage) with a coupling constant of 8.6 Hz – indicative of an ortho benzenoid coupling. It is clear that such coupling can only arise in the case of only one borylation taking place on the benzenoid ring and the other on the indolyl ring. However, upon addition of pinacol, the coupling constant between the two doublets in the bis-BPin product was then 4 Hz (see spectra in NMR data section), indicating a ^3J C2(H)-C3(H) coupling. This suggests an isomerisation process proceeds upon addition of pinacol. *Note: This effect could not be observed by NMR spectroscopy in the borylation of* ***14*** *due to the low solubility of the –BBr_2 boracycle in common organic solvents*.

The below spectra for **17** + 3.2 eq. BBr_3 to produce a C7/ C3 borylated product (compound C). Note: this product isomerised to the desired C5/ C7 BPin product upon addition of pinacol. The spectra were obtained after the volatiles (DCM, Heptane, BBr_3) were removed under vacuum and the product was re-dissolved in dry $CDCl_3$

^{1}H NMR Spectrum ($CDCl_3$) (major impurities are due to $CHCl_3$, DCM and heptanes)

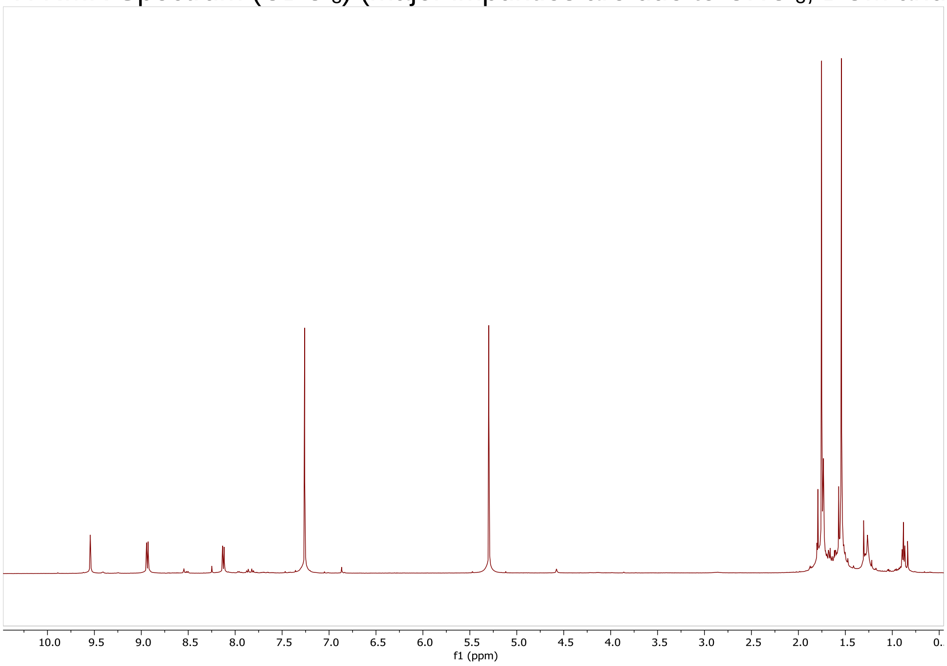

^{13}C{^{1}H} NMR Spectrum of the aromatic region

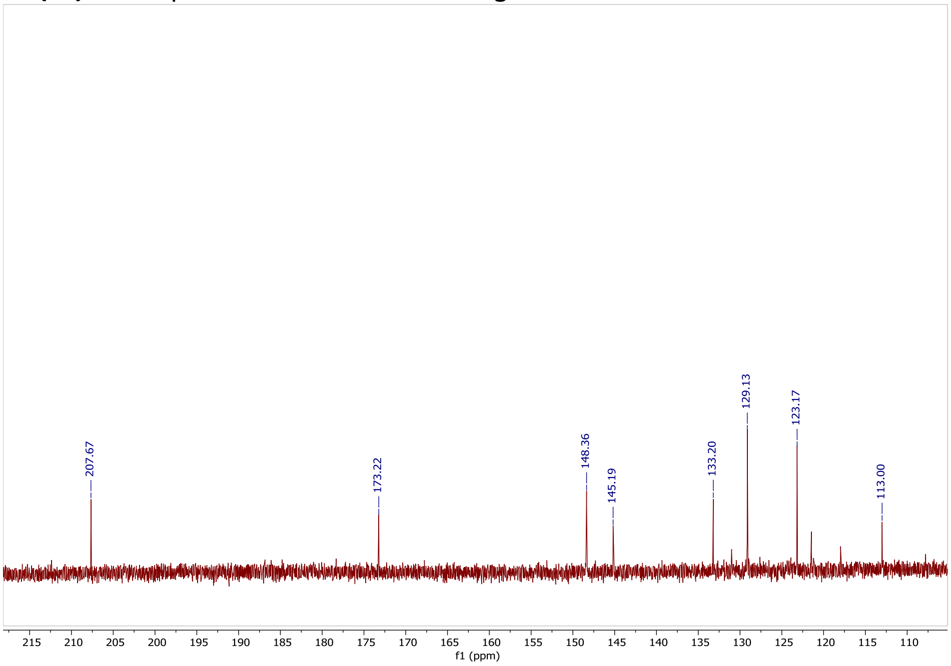

NOESY (top). HSQC (bottom) Dotted lines indicate NOESY interaction between ^{t}Bu (N1-pivaloyl) and the C2-H. In order to obtain clear NOESY spectrum,.

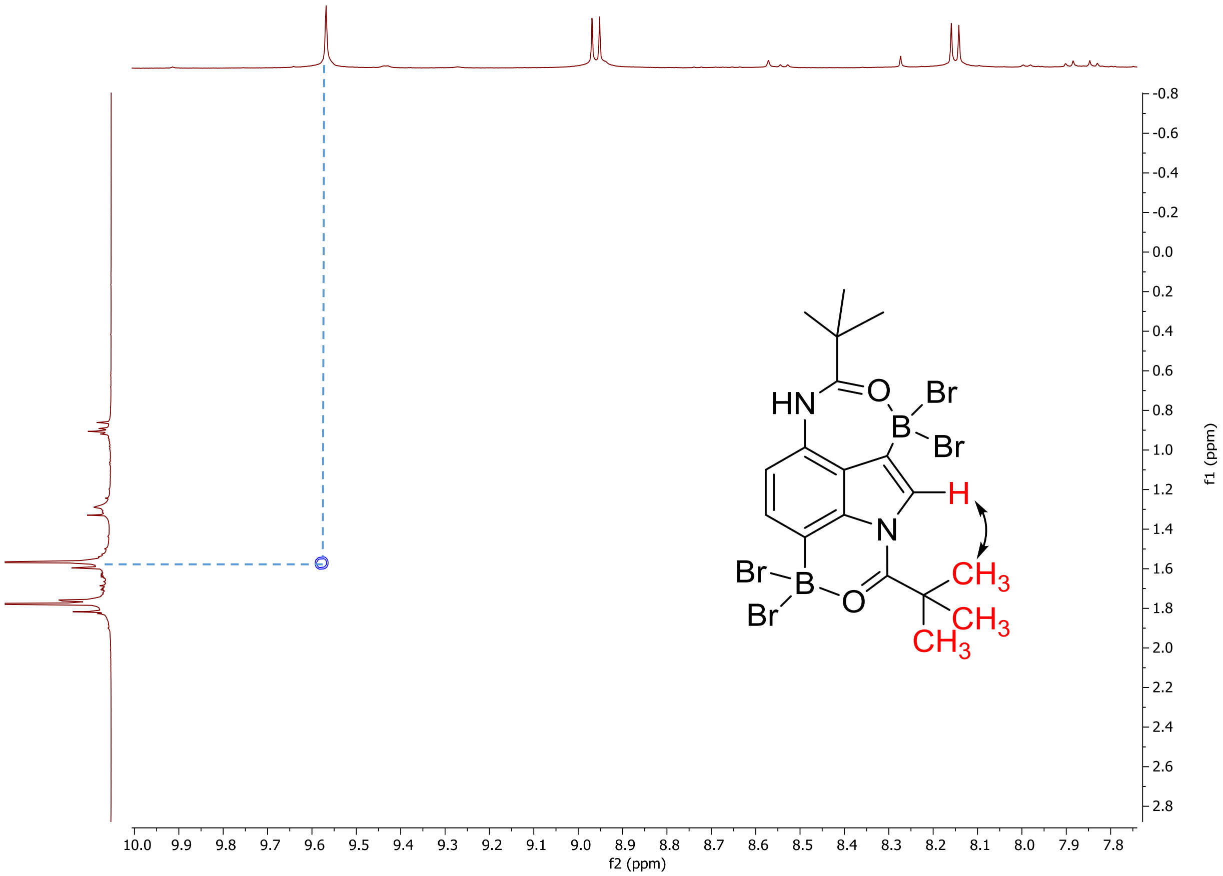

The C2-H is shifted significantly downfield in **C**, however a HSQC spectrum confirmed it is a C-H (and not a N-H, for example).

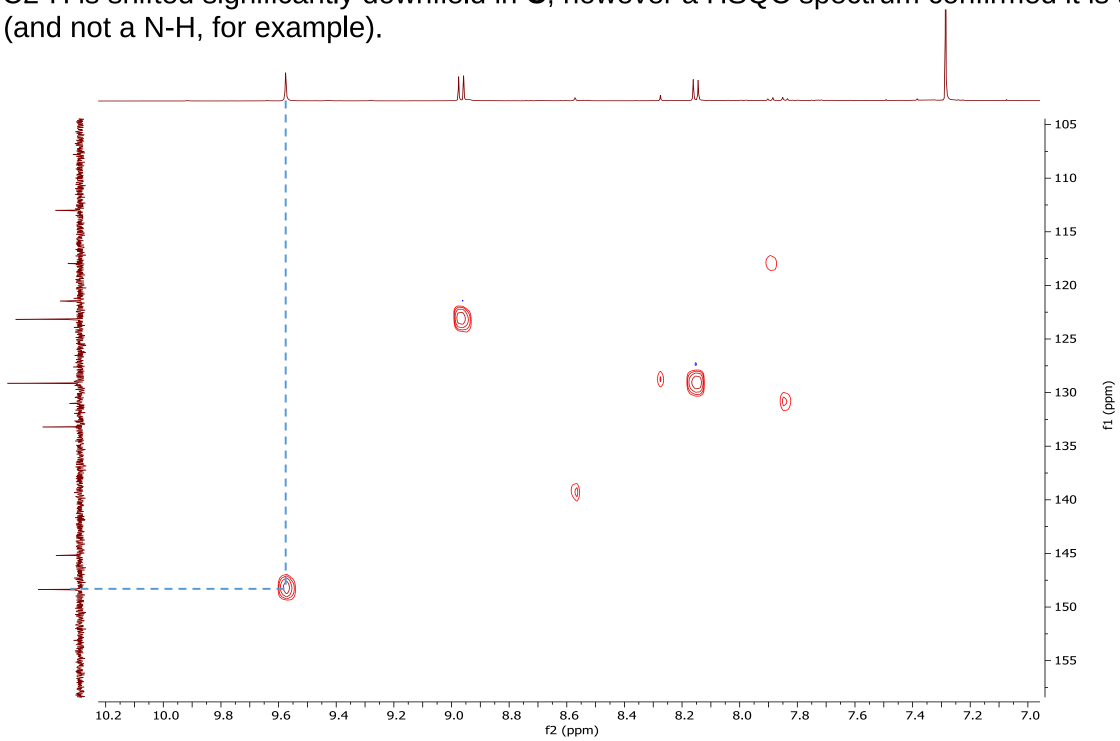

Spectroscopic data

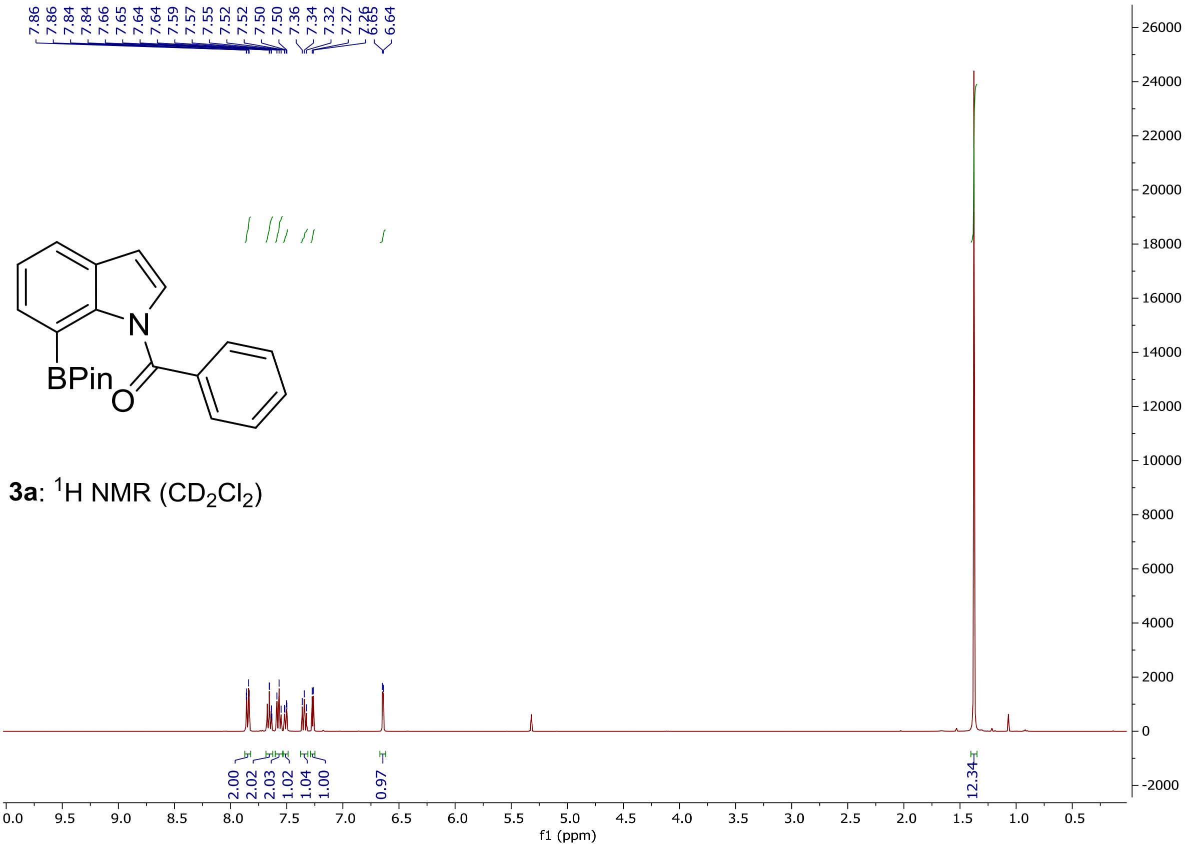

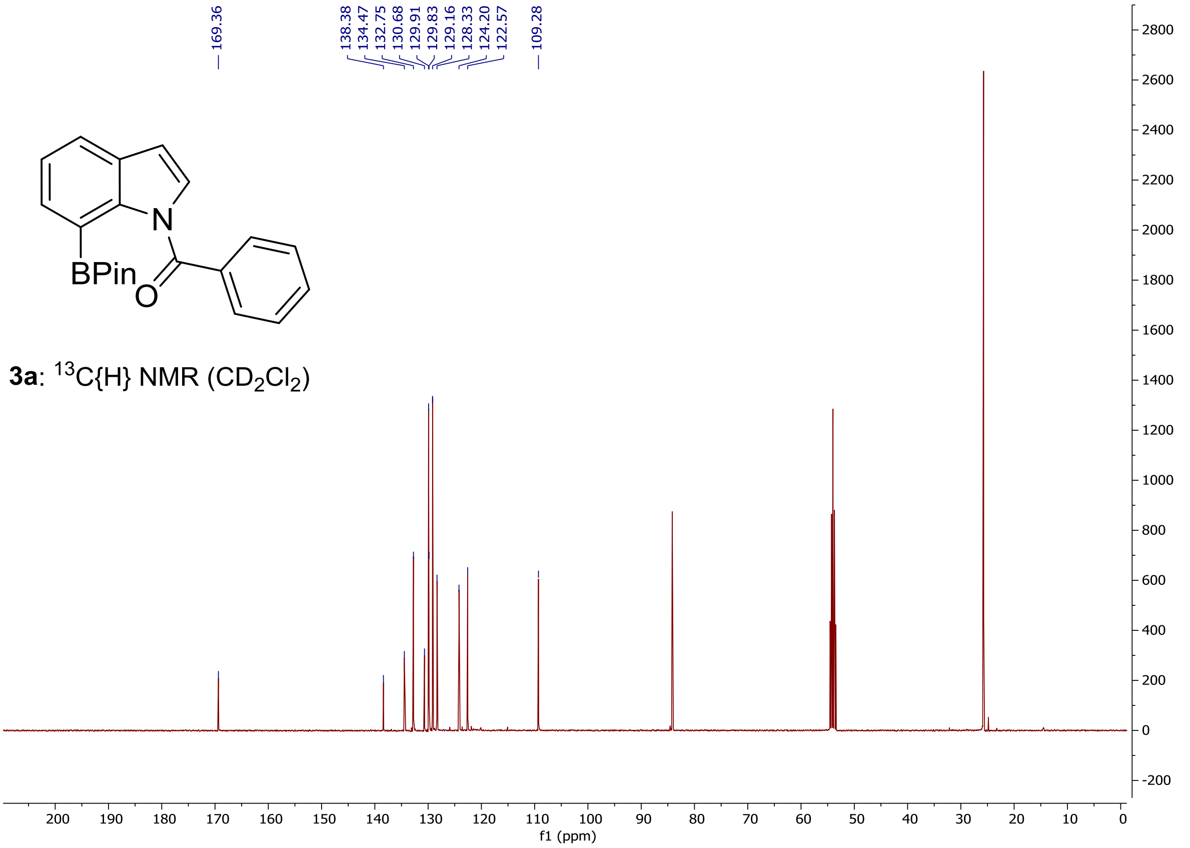

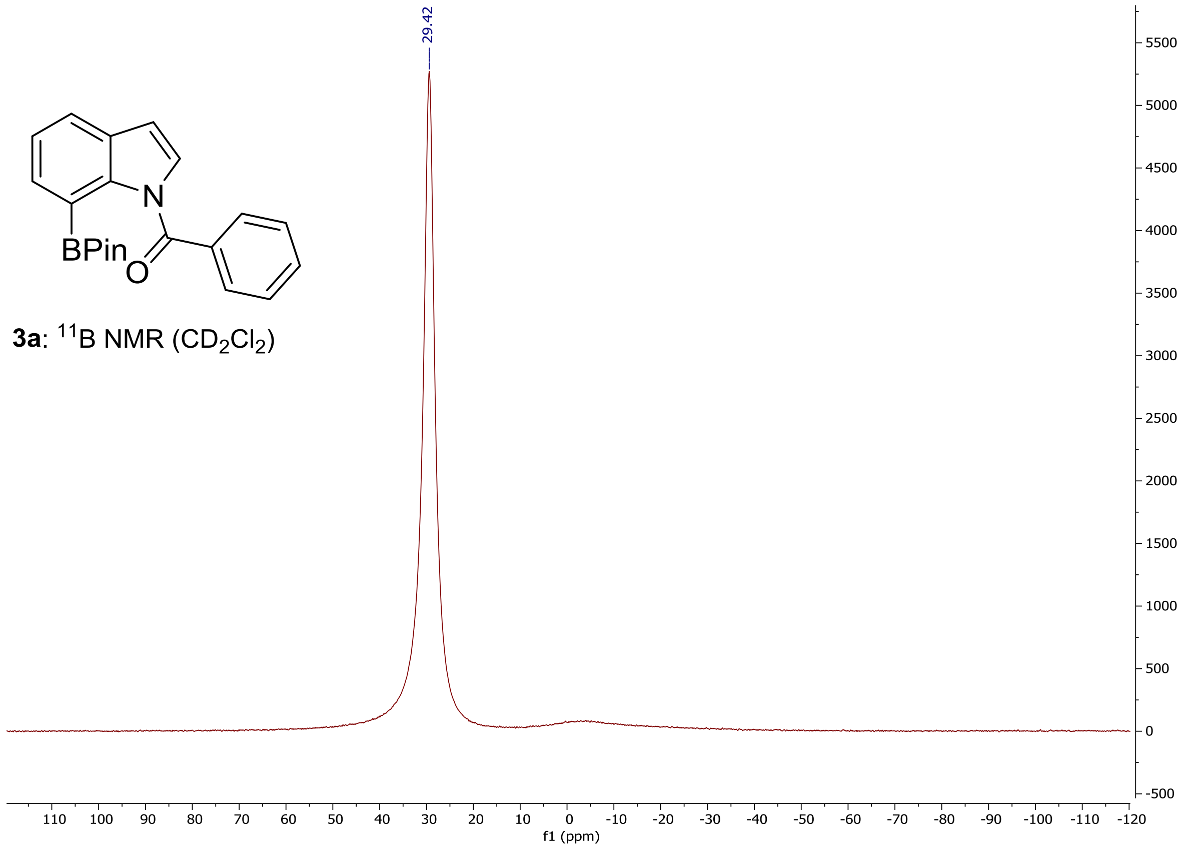

3a: ^{11}B NMR (CD_2Cl_2)

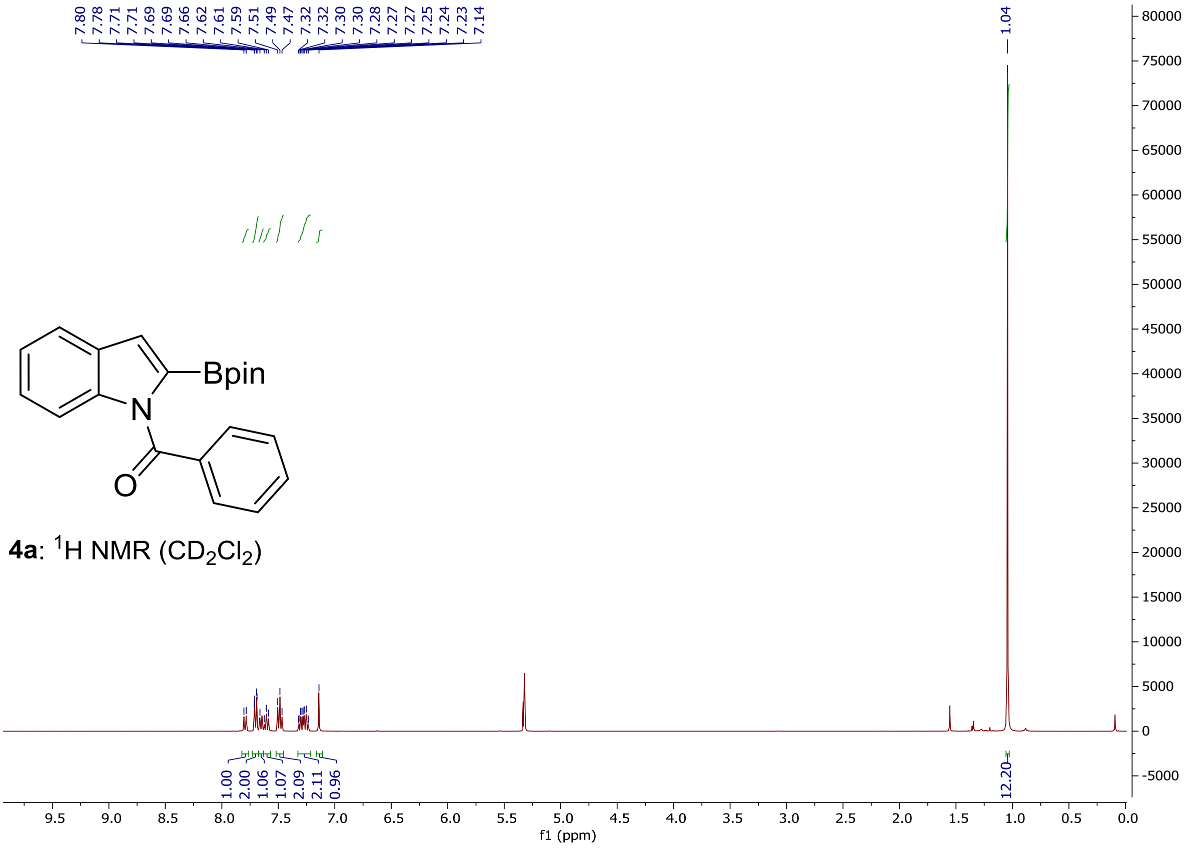

4a: ^{1}H NMR (CD_2Cl_2)

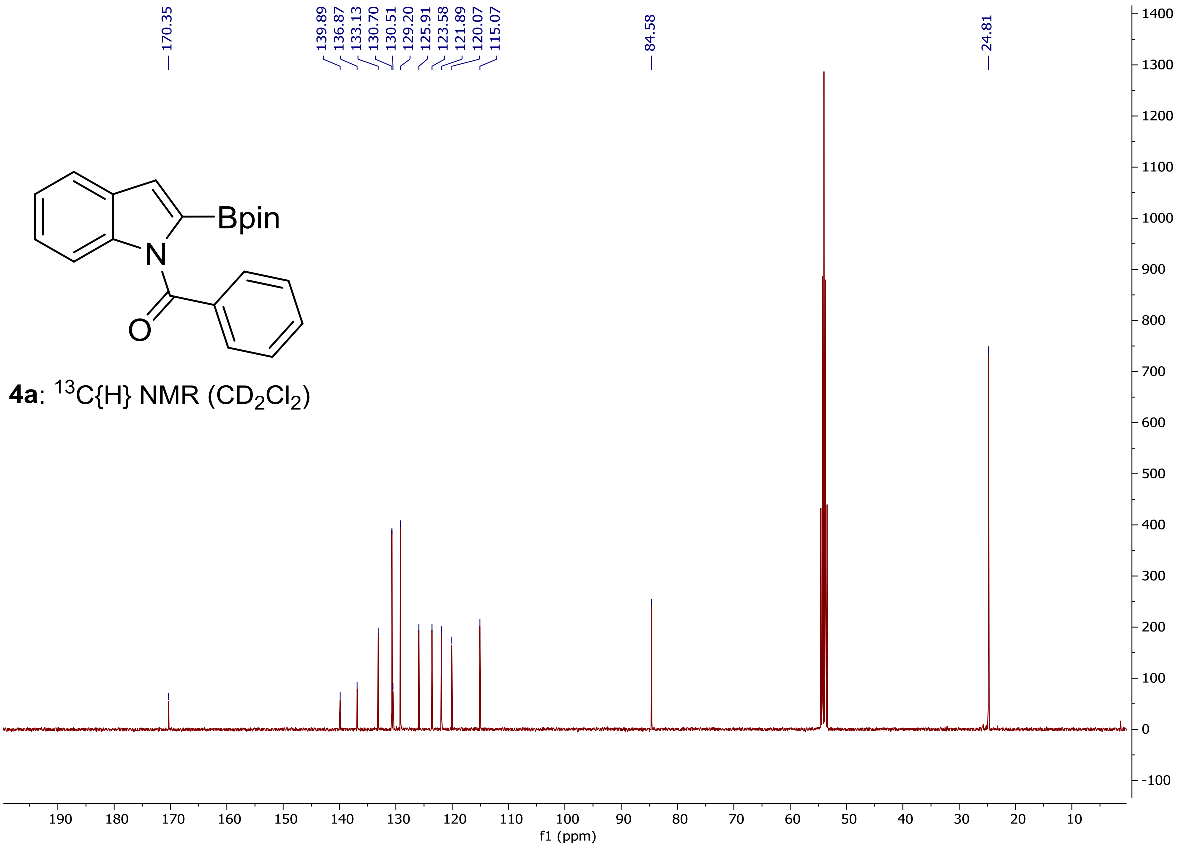

4a: $^{13}C\{H\}$ NMR (CD_2Cl_2)

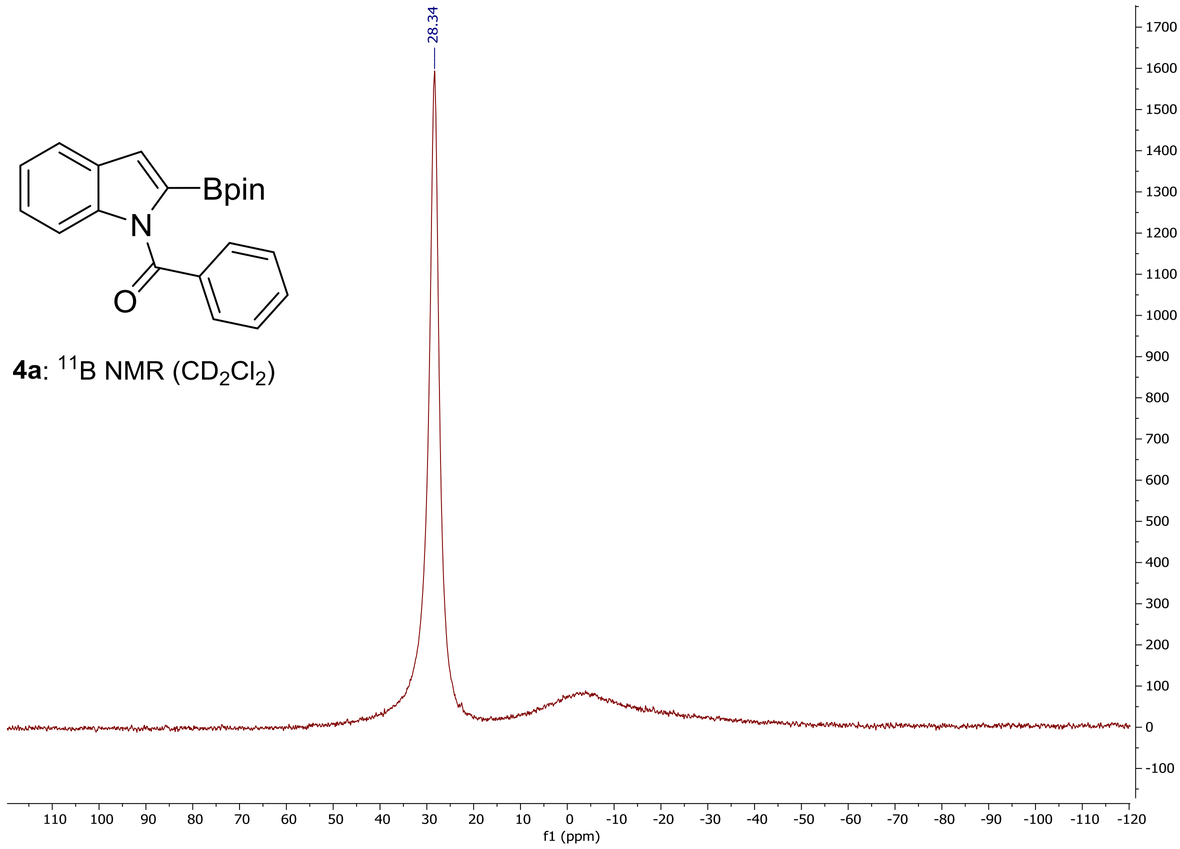

4a: ^{11}B NMR (CD_2Cl_2)

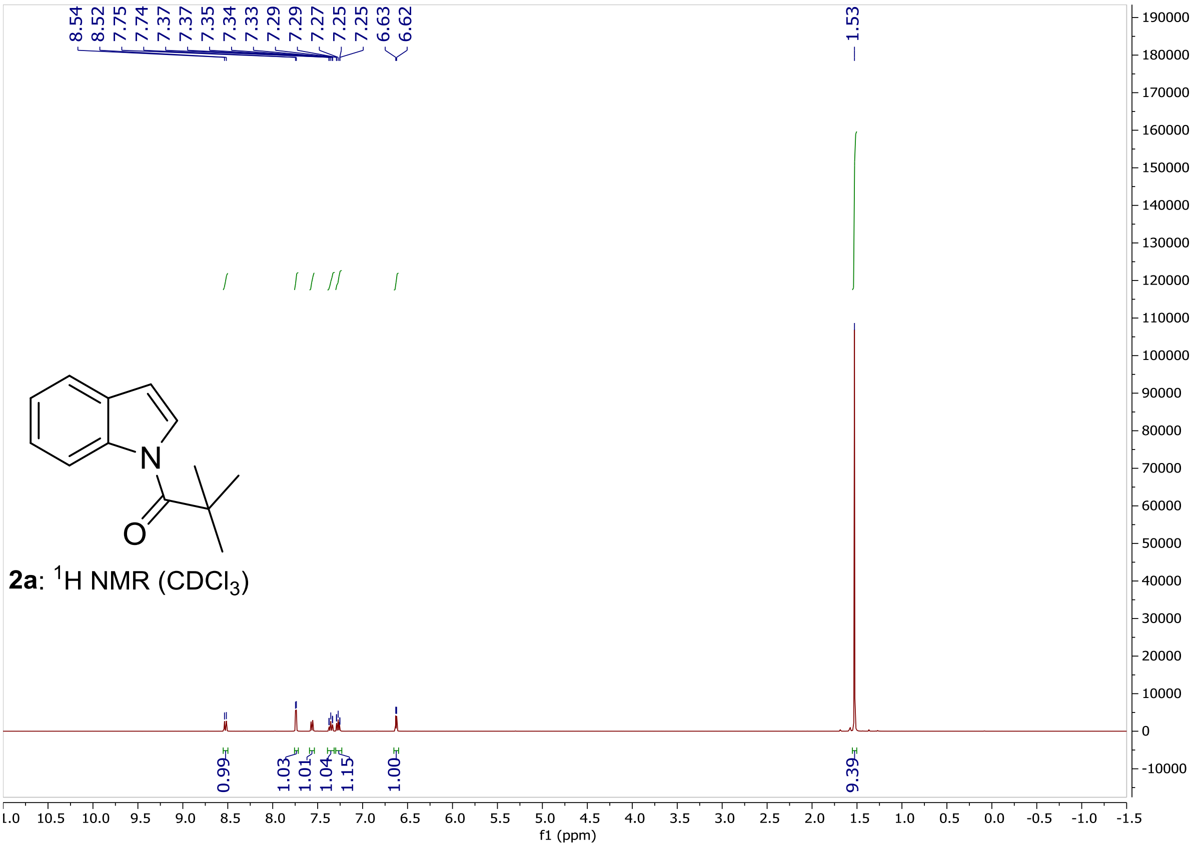
8.54
8.52
7.75
7.74
7.37
7.37
7.35
7.34
7.33
7.29
7.29
7.27
7.25
7.25
6.63
6.62
1.53
2a: ^{1}H NMR ($CDCl_3$)
0.99
1.03
1.01
1.04
1.15
1.00
9.39
f1 (ppm)

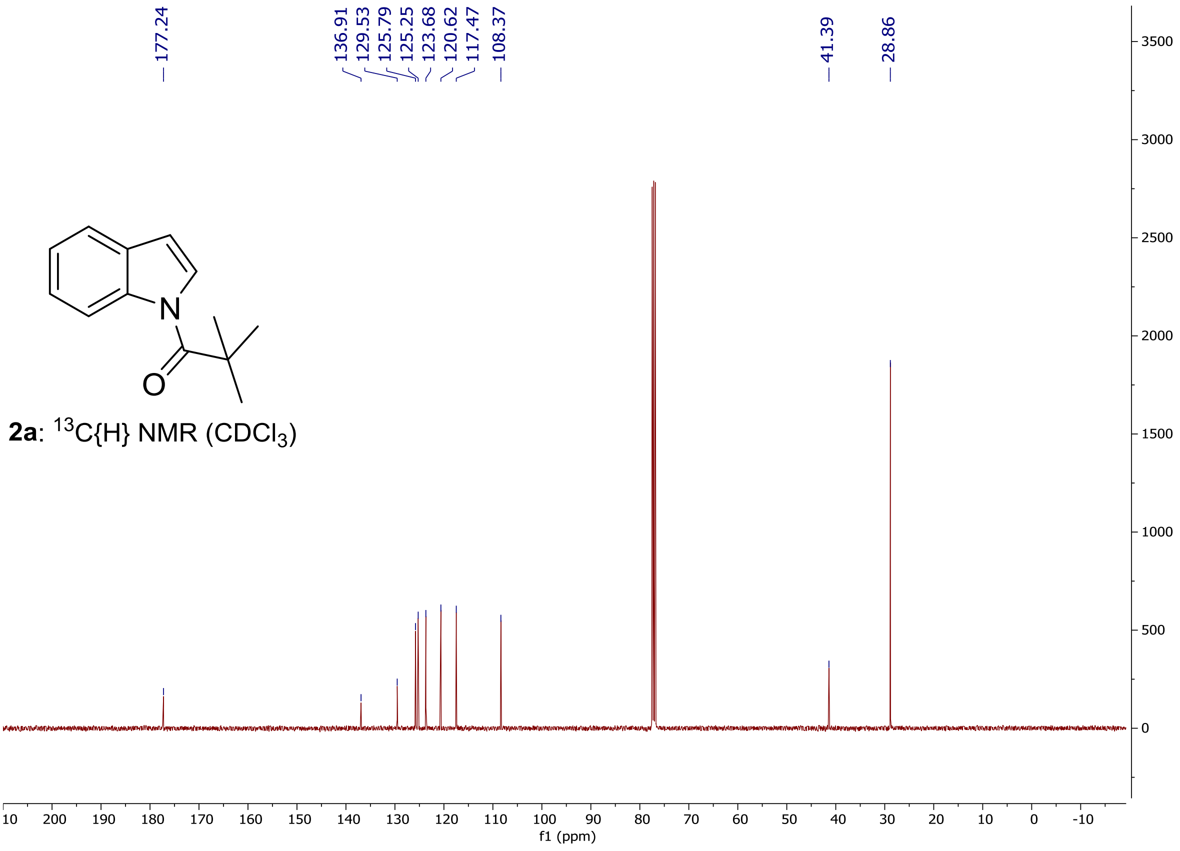
177.24
136.91
129.53
125.79
125.25
123.68
120.62
117.47
108.37
41.39
28.86
2a: ^{13}C{H} NMR ($CDCl_3$)
f1 (ppm)

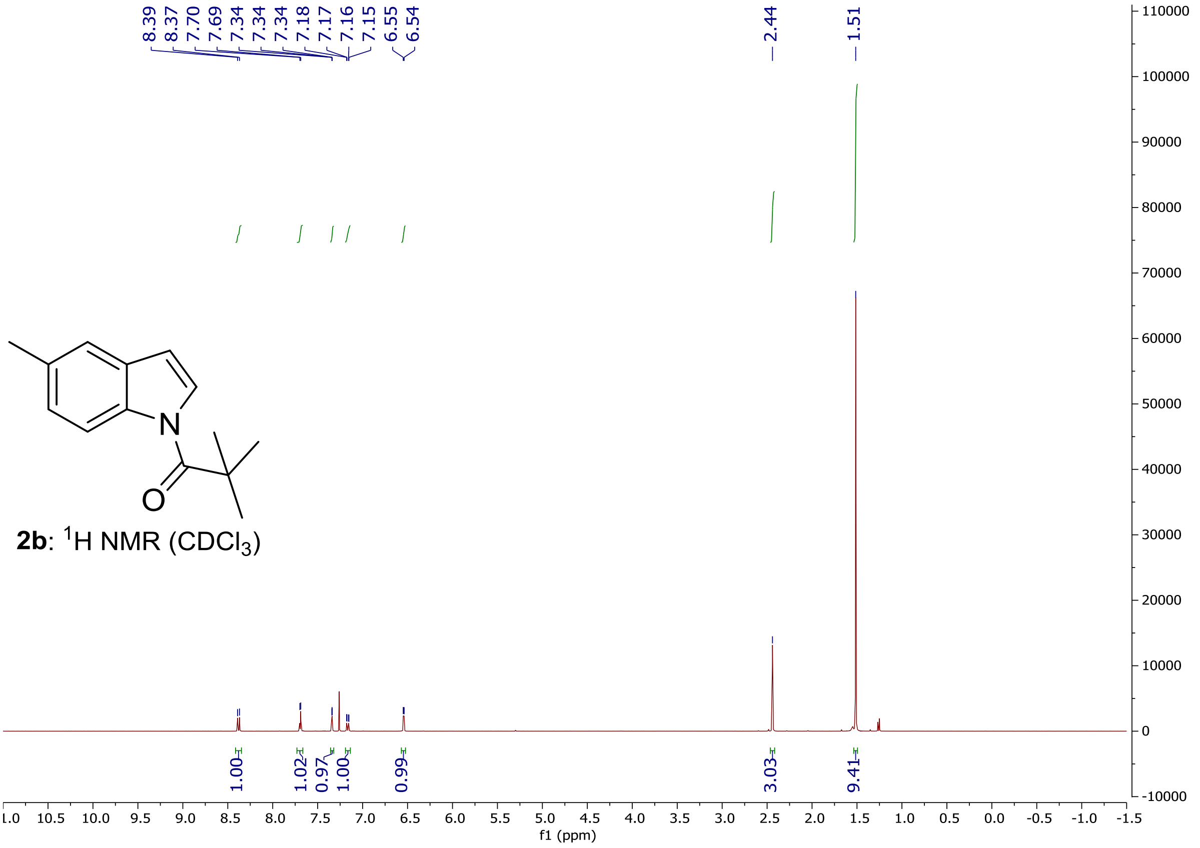
8.39
8.37
7.70
7.69
7.34
7.34
7.34
7.18
7.17
7.16
7.15
6.55
6.54
2.44
1.51
1.00
1.02
0.97
1.00
0.99
3.03
9.41
f1 (ppm)
2b: 1H NMR (CDCl3)

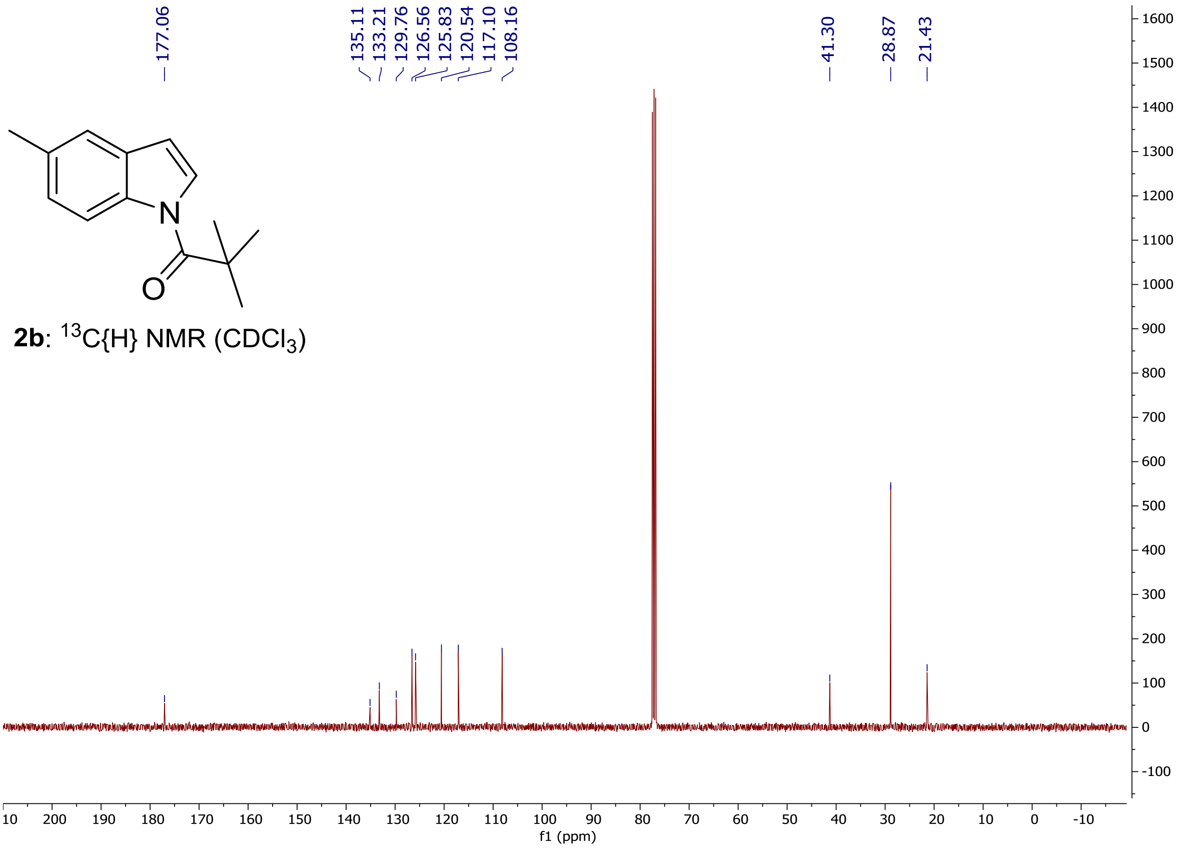
177.06
135.11
133.21
129.76
126.56
125.83
120.54
117.10
108.16
41.30
28.87
21.43
f1 (ppm)
2b: 13C{H} NMR (CDCl3)

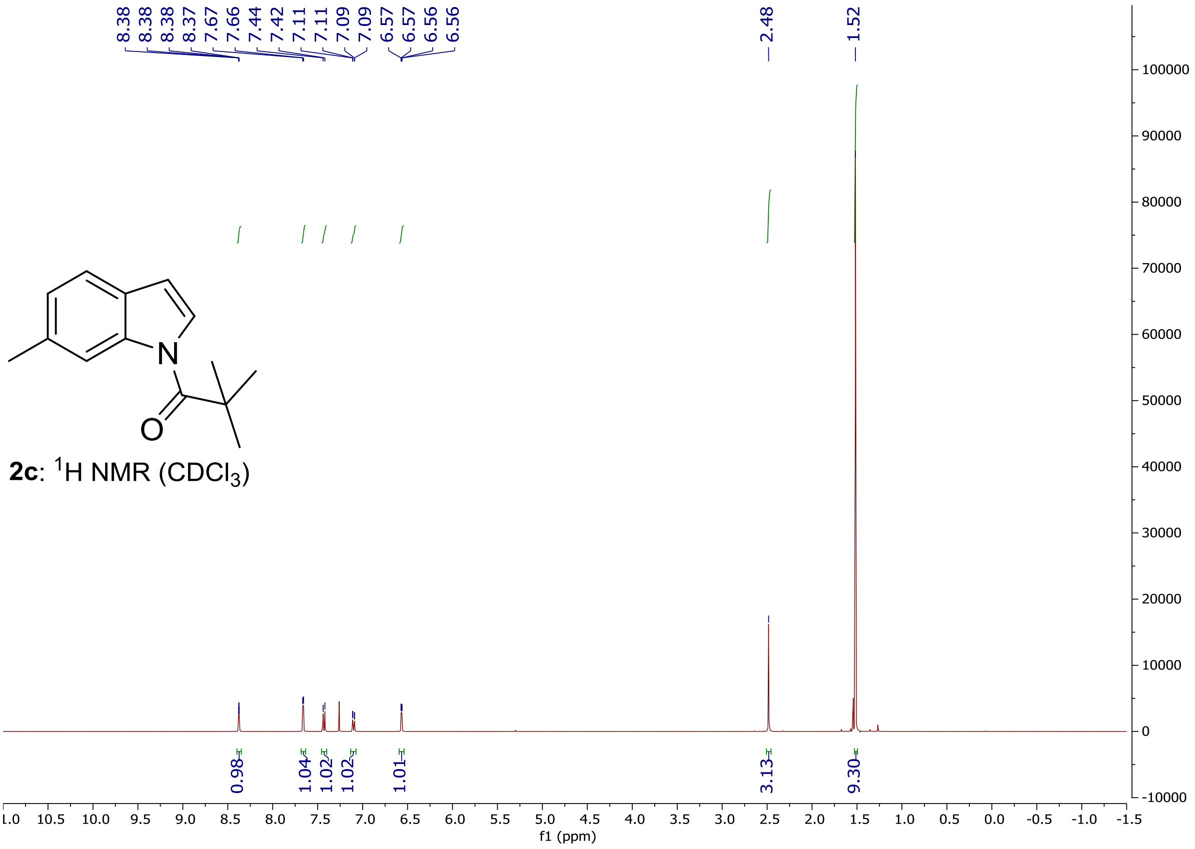
8.38
8.38
8.38
8.37
7.67
7.66
7.44
7.42
7.11
7.11
7.09
7.09
6.57
6.57
6.56
6.56
2.48
1.52
N
O
2c: ¹H NMR (CDCl₃)
0.98
1.04
1.02
1.02
1.01
3.13
9.30
f1 (ppm)

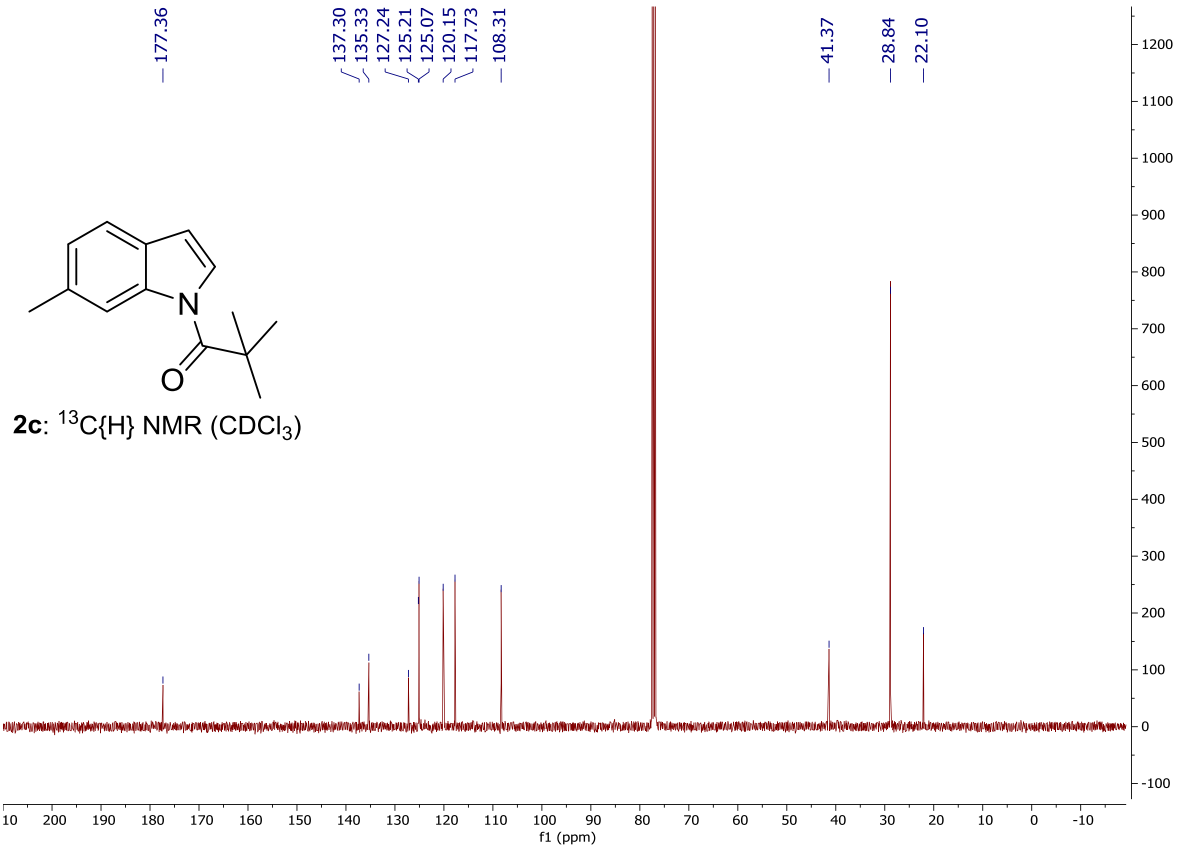
177.36
137.30
135.33
127.24
125.21
125.07
120.15
117.73
108.31
41.37
28.84
22.10
N
O
2c: ¹³C{H} NMR (CDCl₃)
f1 (ppm)

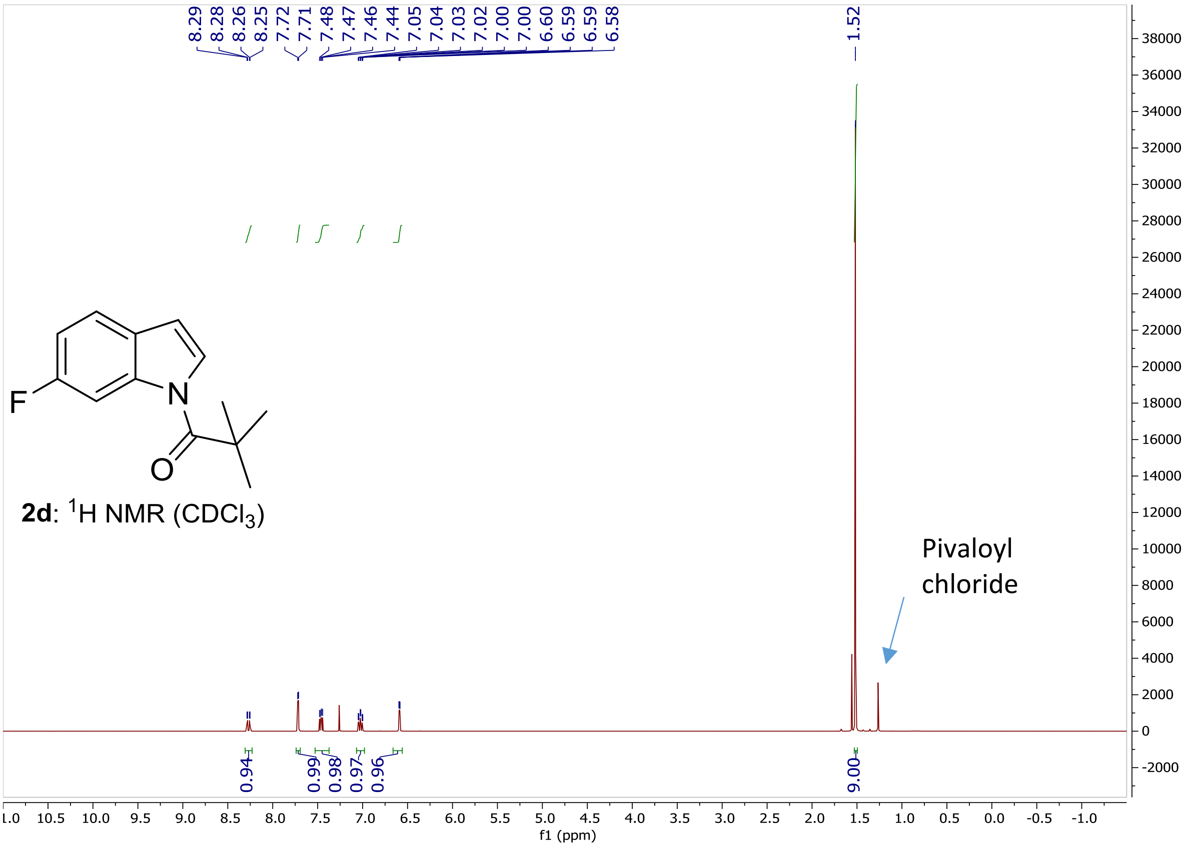

F
N
O
2d: 1H NMR (CDCl3)
Pivaloyl
chloride
8.29
8.28
8.26
8.25
7.72
7.71
7.48
7.47
7.46
7.44
7.05
7.04
7.03
7.02
7.00
7.00
6.60
6.59
6.59
6.58
1.52
0.94
0.99
0.98
0.97
0.96
9.00
f1 (ppm)

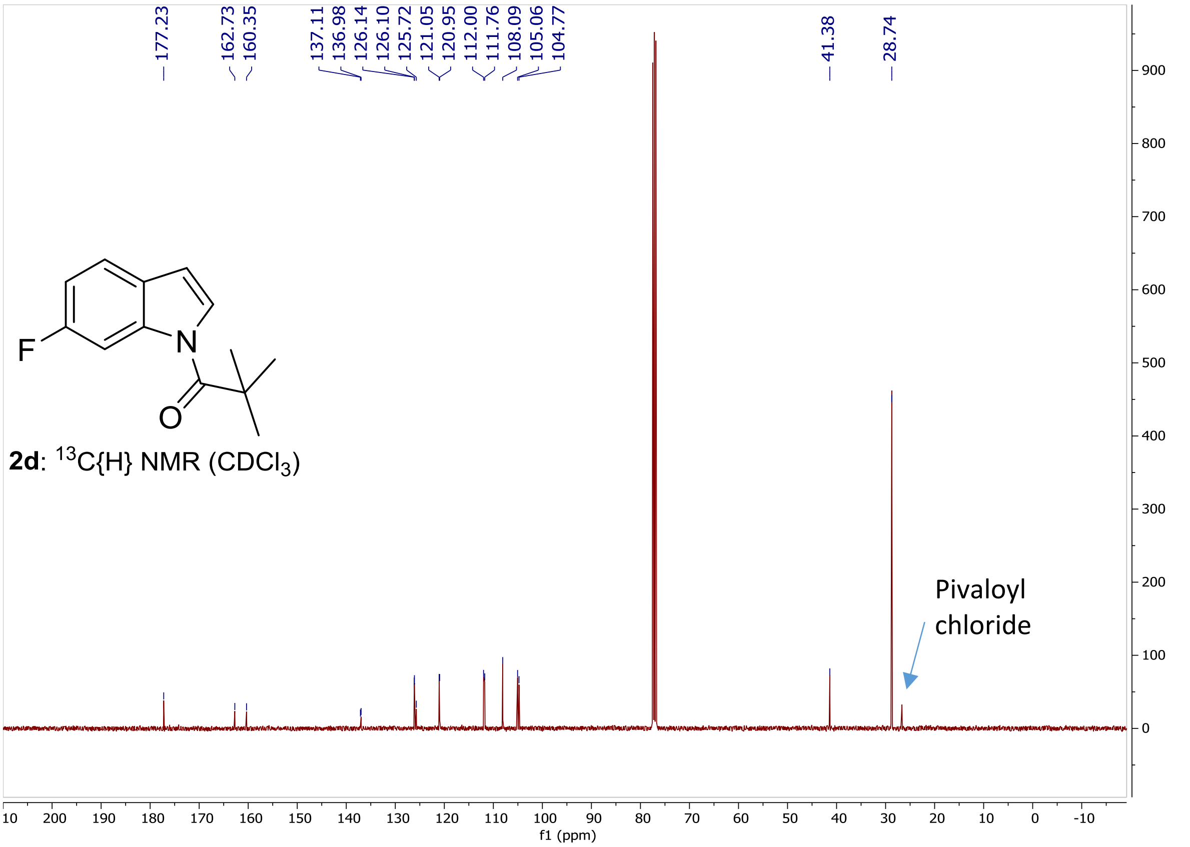

F
N
O
2d: 13C{H} NMR (CDCl3)
Pivaloyl
chloride
177.23
162.73
160.35
137.11
136.98
126.14
126.10
125.72
121.05
120.95
112.00
111.76
108.09
105.06
104.77
41.38
28.74
f1 (ppm)

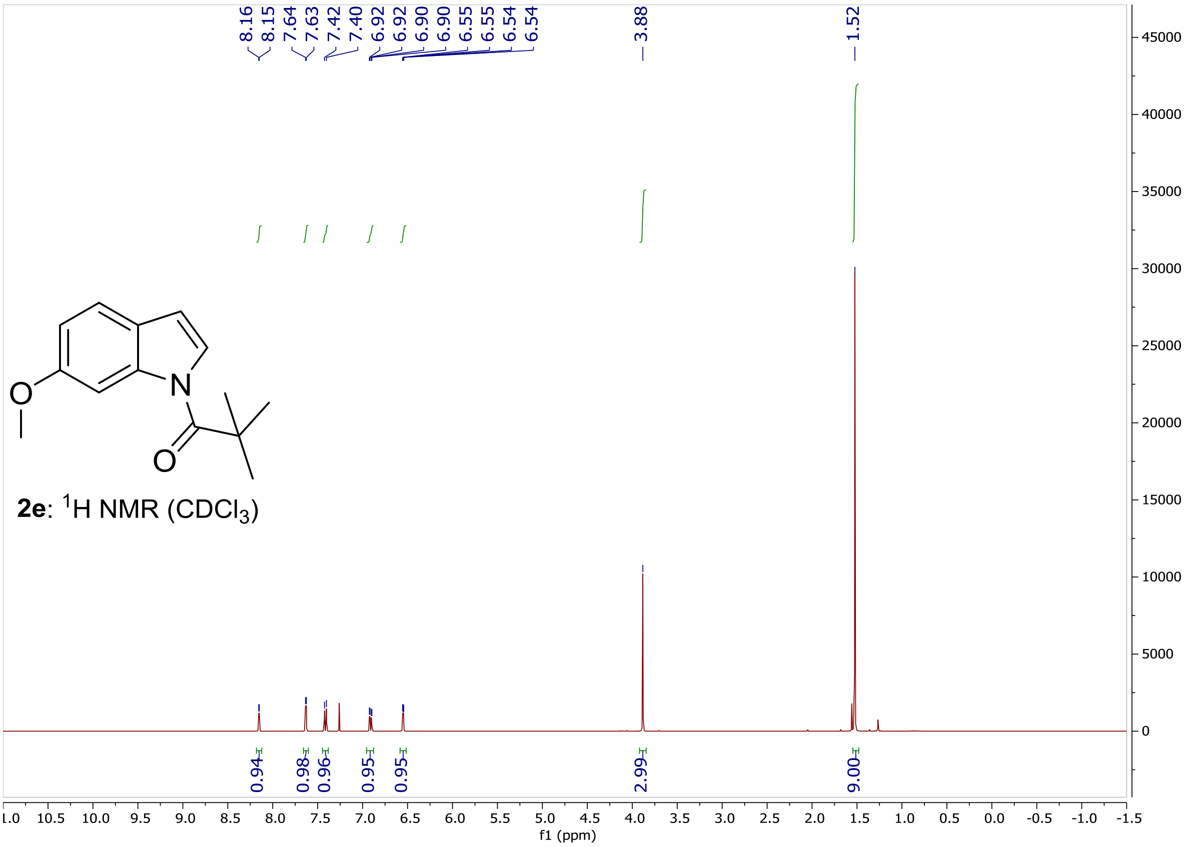
8.16
8.15
7.64
7.63
7.42
7.40
6.92
6.92
6.90
6.90
6.55
6.55
6.54
6.54
3.88
1.52
0.94
0.98
0.96
0.95
0.95
2.99
9.00
2e: ¹H NMR (CDCl3)
f1 (ppm)

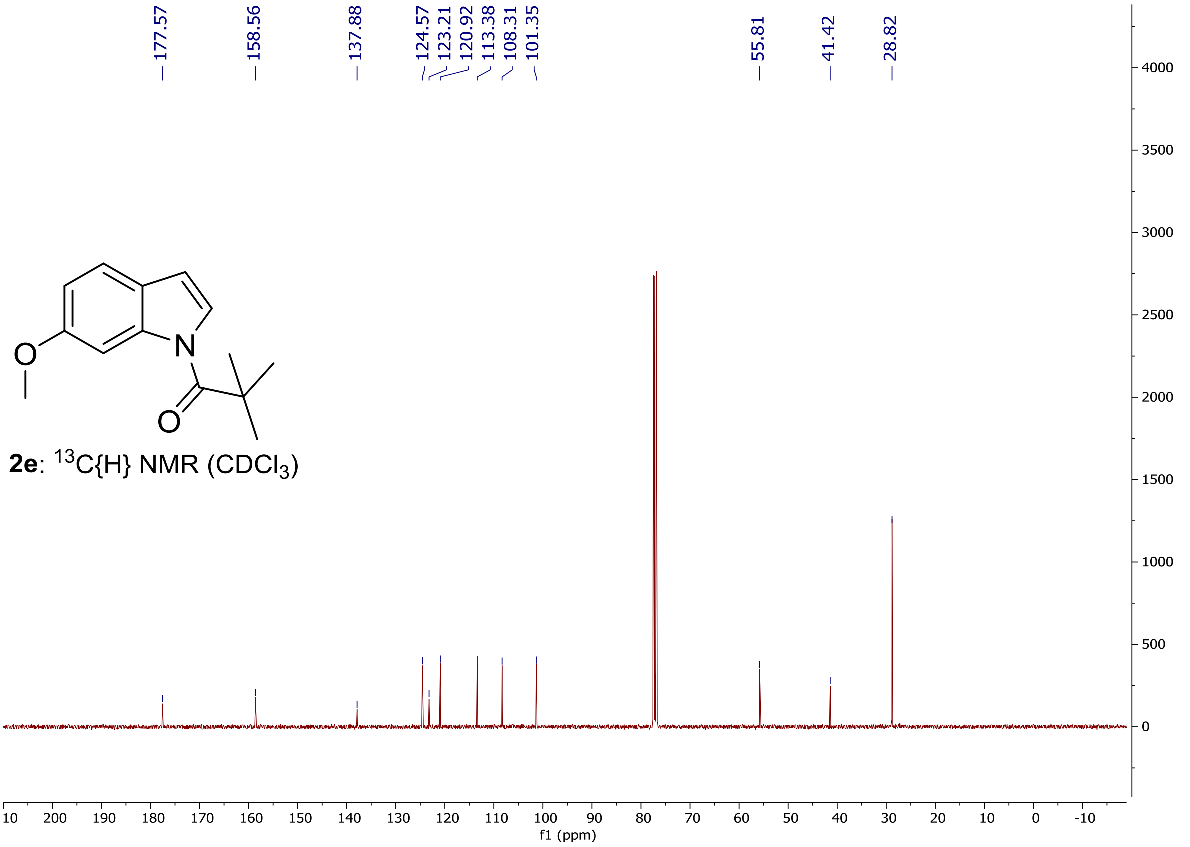
177.57
158.56
137.88
124.57
123.21
120.92
113.38
108.31
101.35
55.81
41.42
28.82
2e: ¹³C{H} NMR (CDCl3)
f1 (ppm)

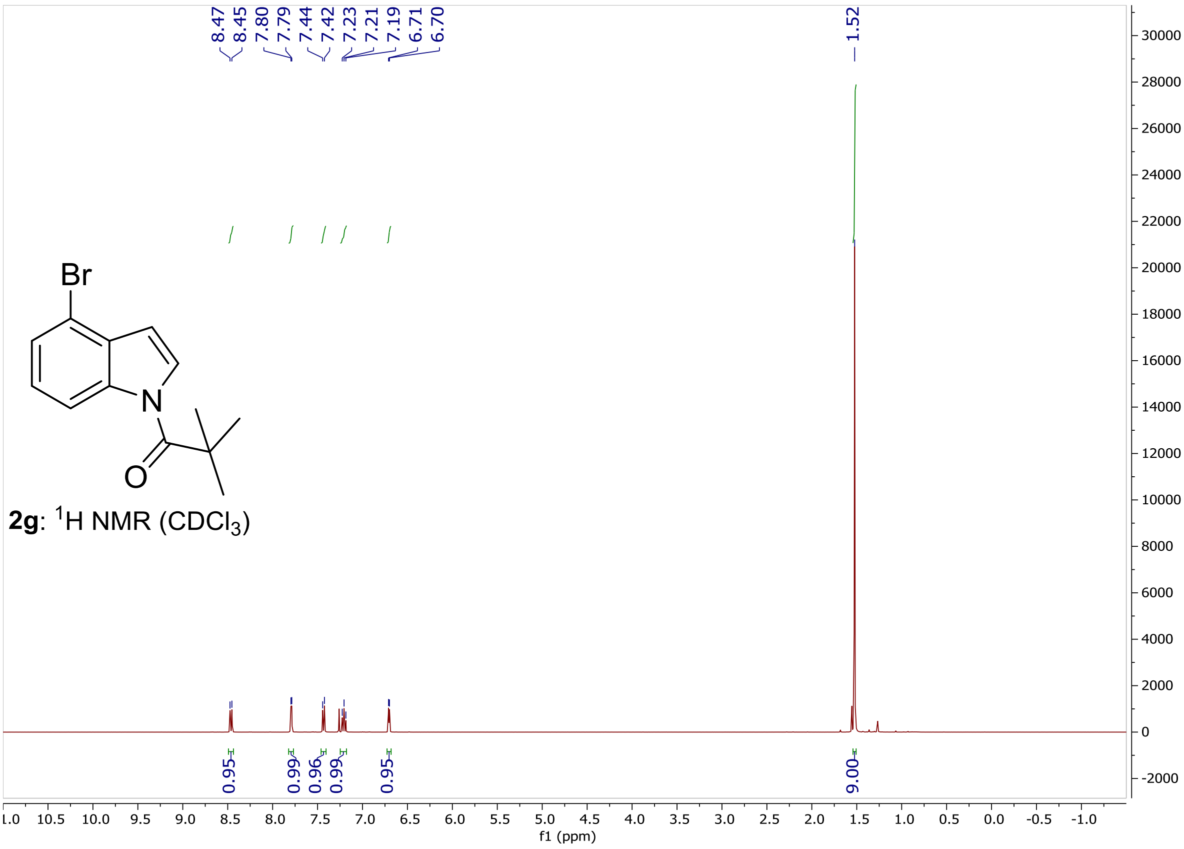
8.47
8.45
7.80
7.79
7.44
7.42
7.23
7.21
7.19
6.71
6.70
1.52
Br
N
O
2g: ^{1}H NMR ($CDCl_3$)
0.95
0.99
0.96
0.99
0.95
9.00
f1 (ppm)

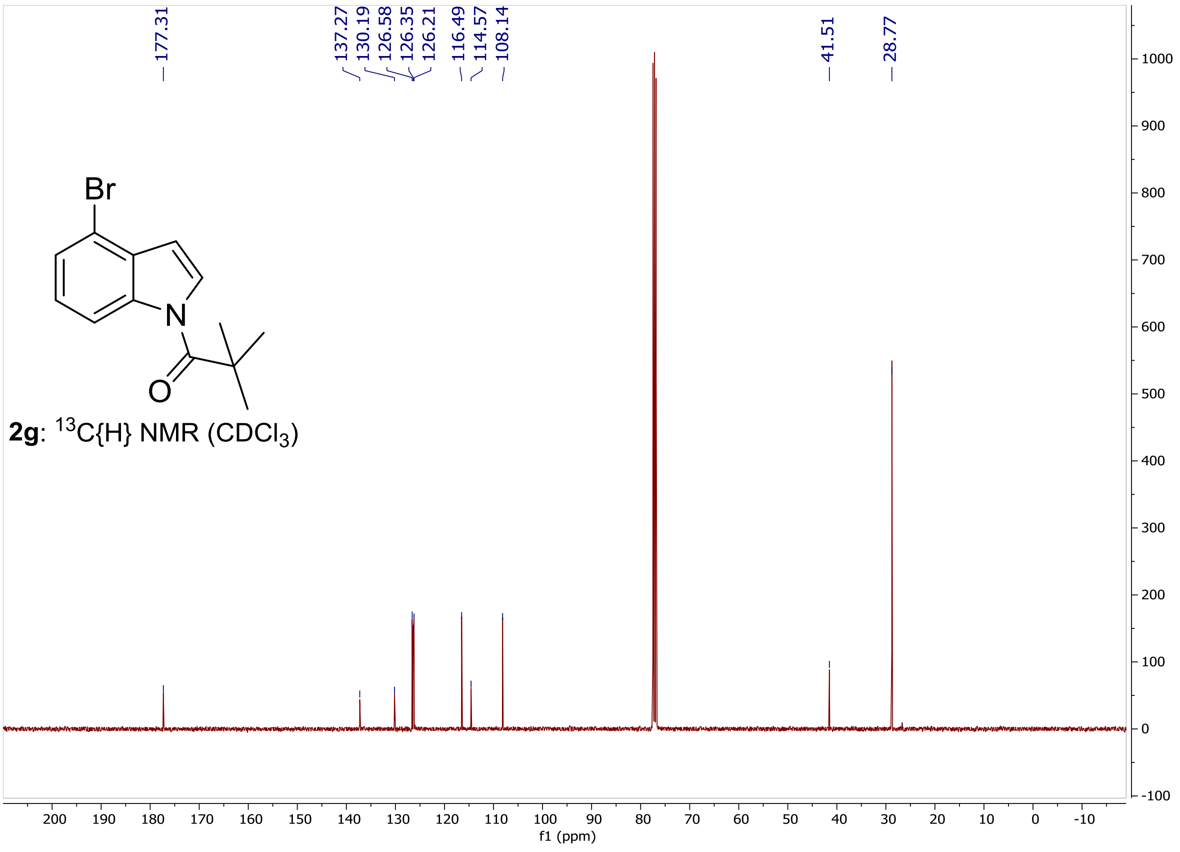
177.31
137.27
130.19
126.58
126.35
126.21
116.49
114.57
108.14
41.51
28.77
Br
N
O
2g: ^{13}C{H} NMR ($CDCl_3$)
f1 (ppm)

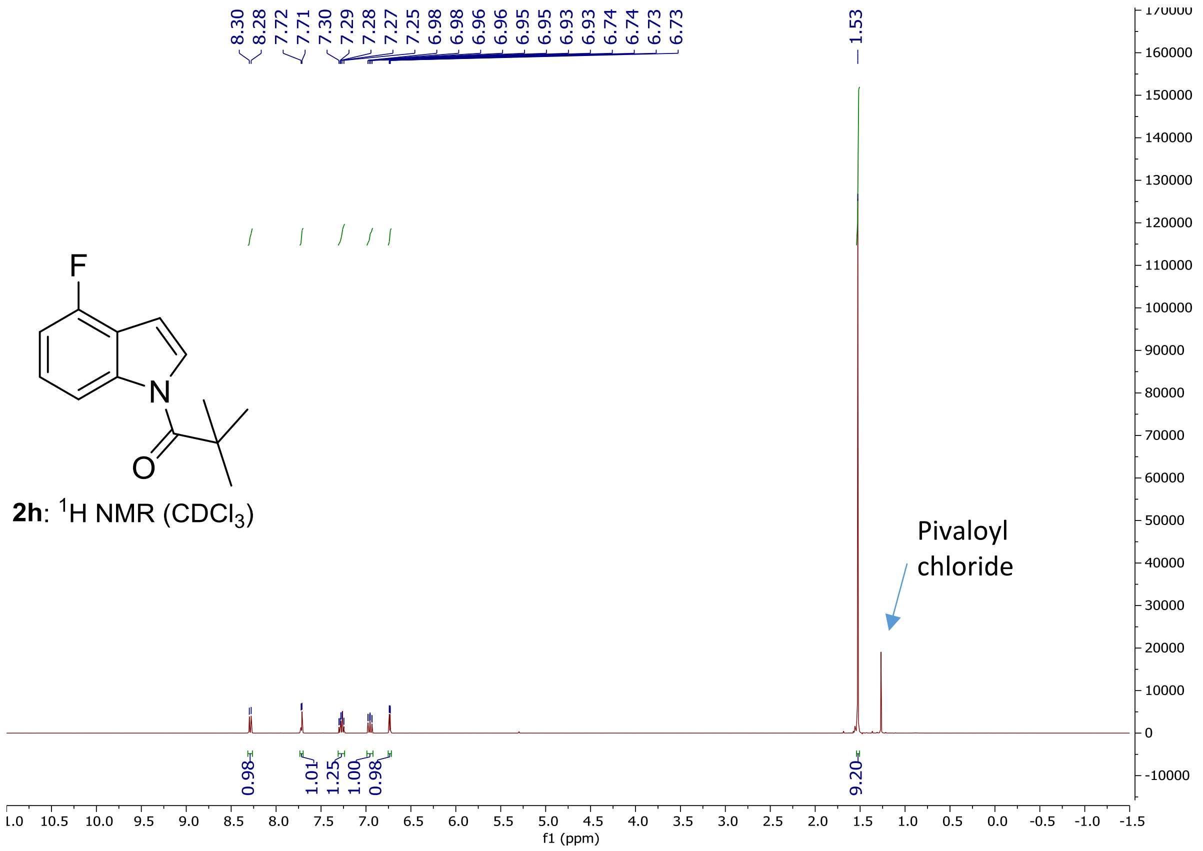

8.30
8.28
7.72
7.71
7.30
7.29
7.28
7.27
7.25
6.98
6.98
6.96
6.96
6.95
6.95
6.93
6.93
6.74
6.74
6.73
6.73
1.53
F
N
O
2h: ^{1}H NMR ($CDCl_3$)
Pivaloyl chloride
0.98
1.01
1.25
1.00
0.98
9.20
f1 (ppm)

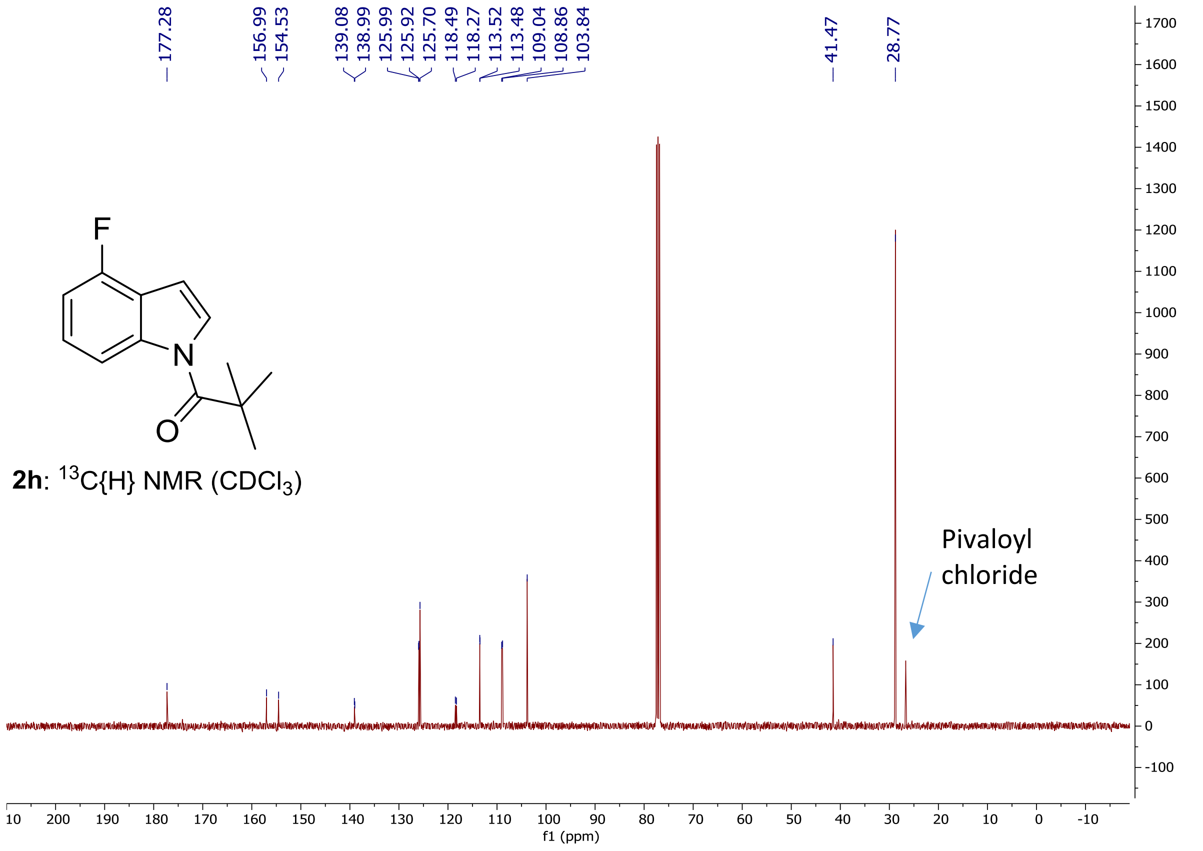

177.28
156.99
154.53
139.08
138.99
125.99
125.92
125.70
118.49
118.27
113.52
113.48
109.04
108.86
103.84
41.47
28.77
F
N
O
2h: ^{13}C{H} NMR ($CDCl_3$)
Pivaloyl chloride
f1 (ppm)

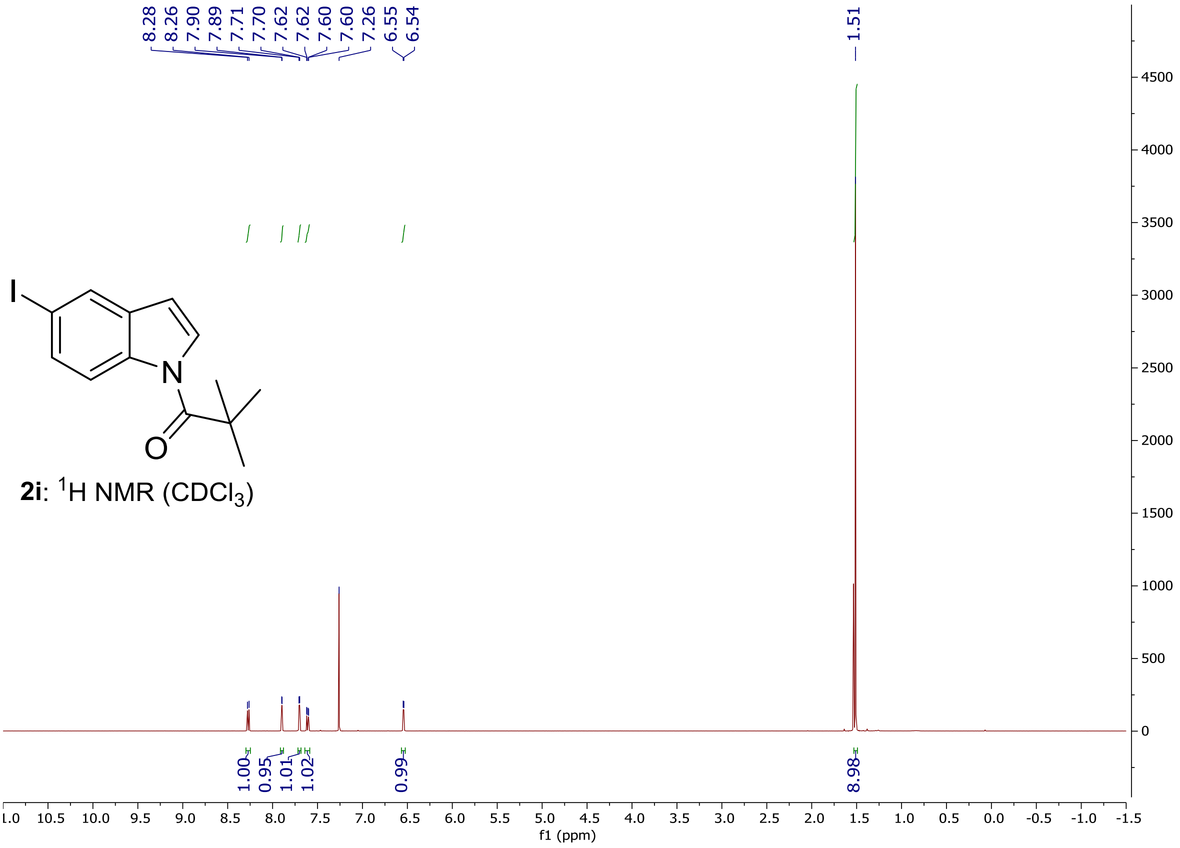
8.28 8.26 7.90 7.89 7.71 7.70 7.62 7.62 7.60 7.60 7.26 6.55 6.54
1.51
2i: ^{1}H NMR (CDCl$_3$)
1.00 0.95 1.01 1.02 0.99 8.98
f1 (ppm)

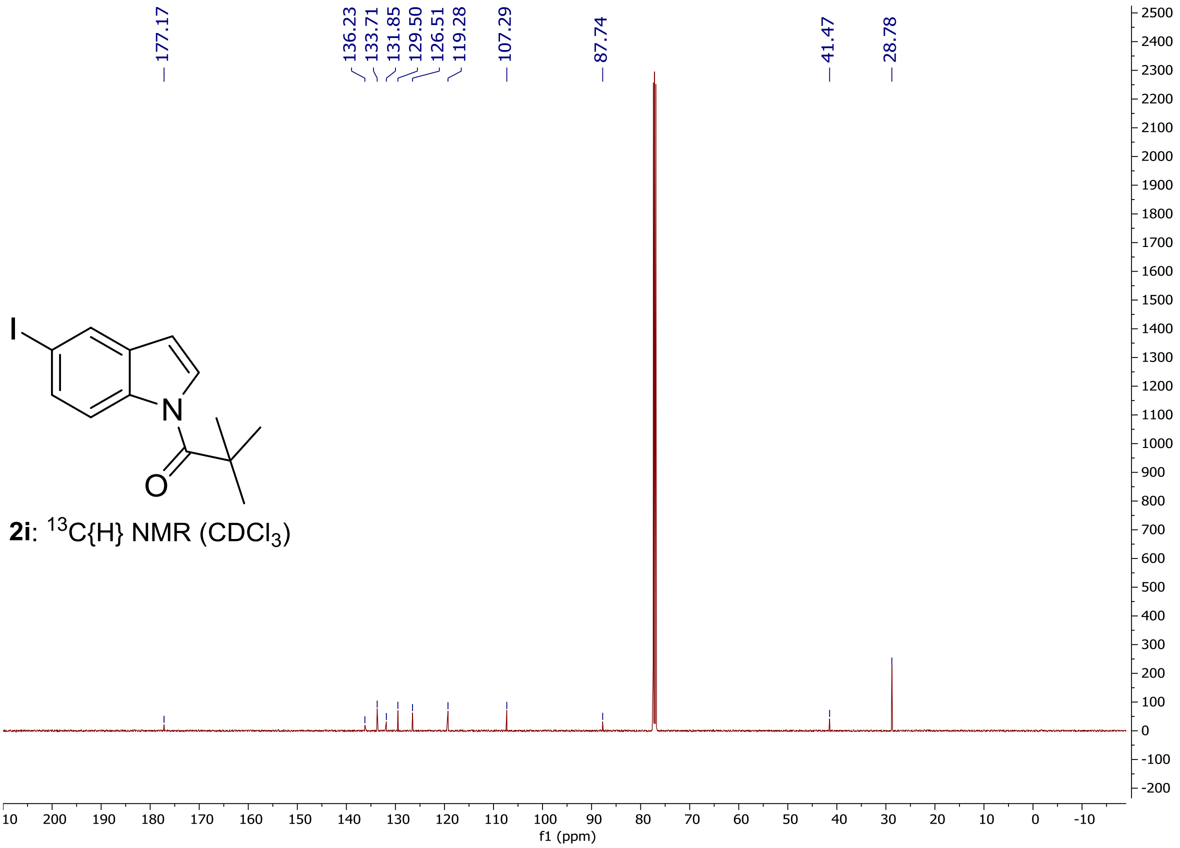
177.17
136.23 133.71 131.85 129.50 126.51 119.28
107.29
87.74
41.47
28.78
2i: ^{13}C{H} NMR (CDCl$_3$)
f1 (ppm)

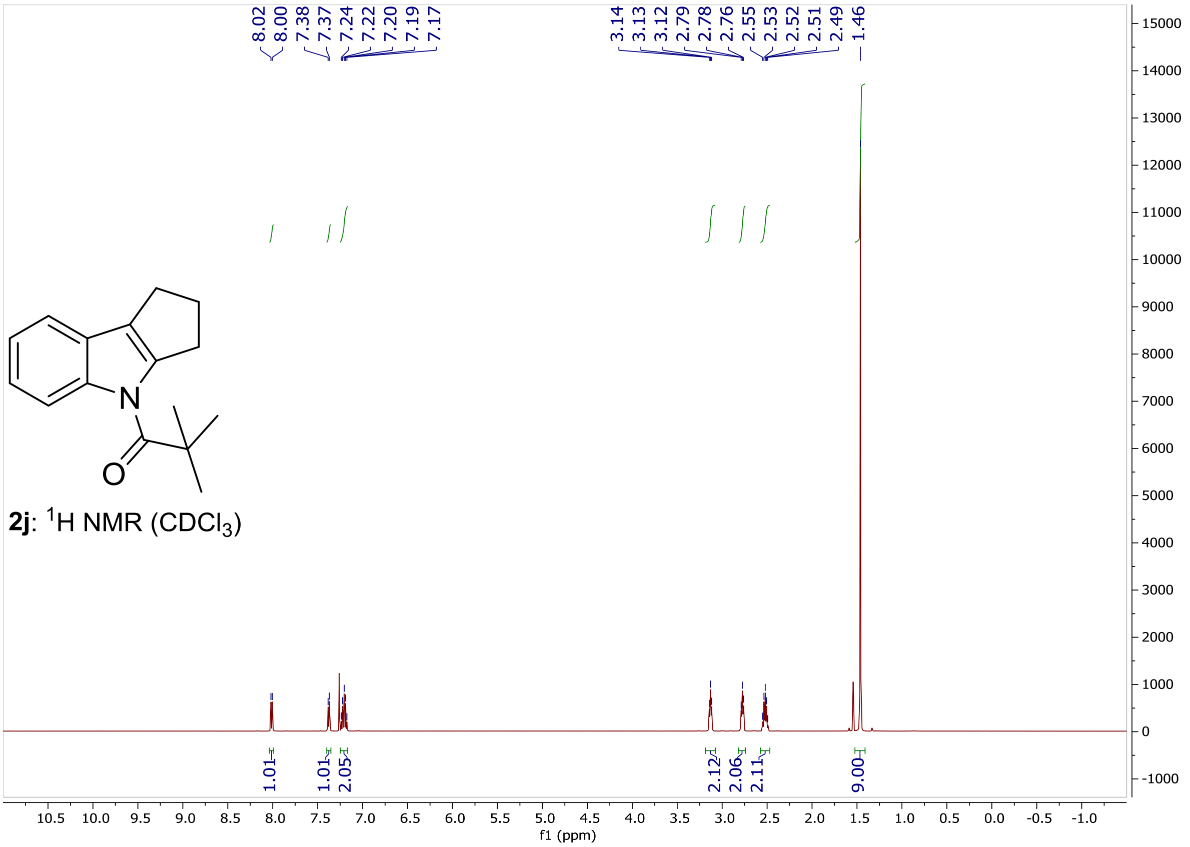
8.02
8.00
7.38
7.37
7.24
7.22
7.20
7.19
7.17
3.14
3.13
3.12
2.79
2.78
2.76
2.55
2.53
2.52
2.51
2.49
1.46
N
O
2j: ^{1}H NMR ($CDCl_3$)
1.01
1.01
2.05
2.12
2.06
2.11
9.00
f1 (ppm)

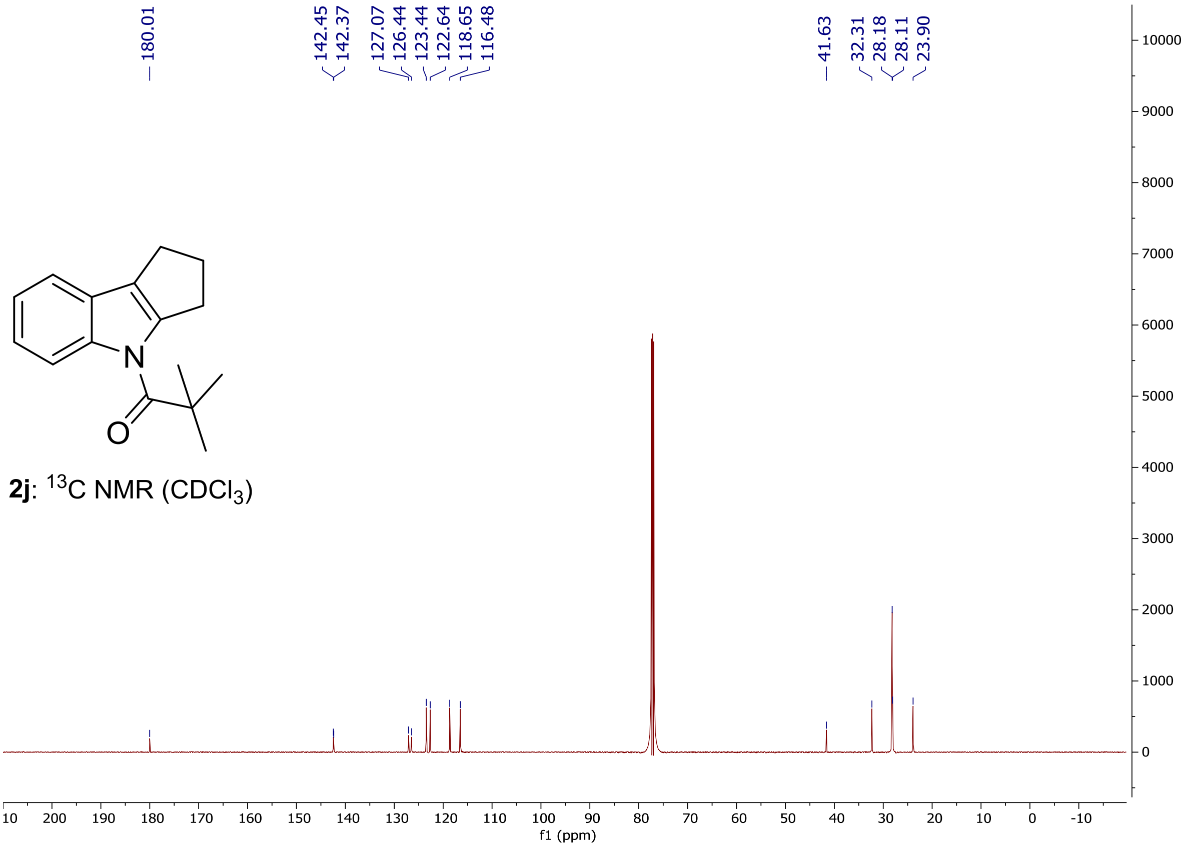
180.01
142.45
142.37
127.07
126.44
123.44
122.64
118.65
116.48
41.63
32.31
28.18
28.11
23.90
N
O
2j: ^{13}C NMR ($CDCl_3$)
f1 (ppm)

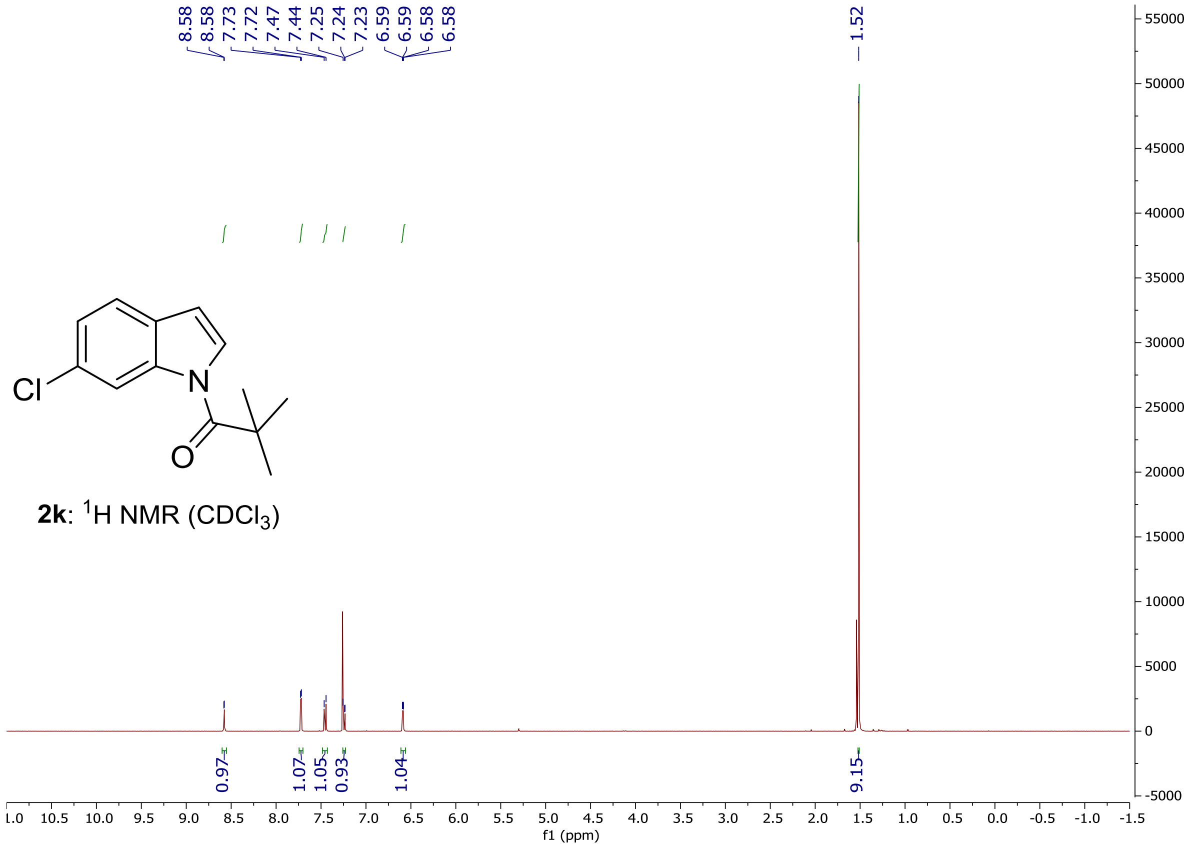

2k: ^{1}H NMR ($CDCl_3$)

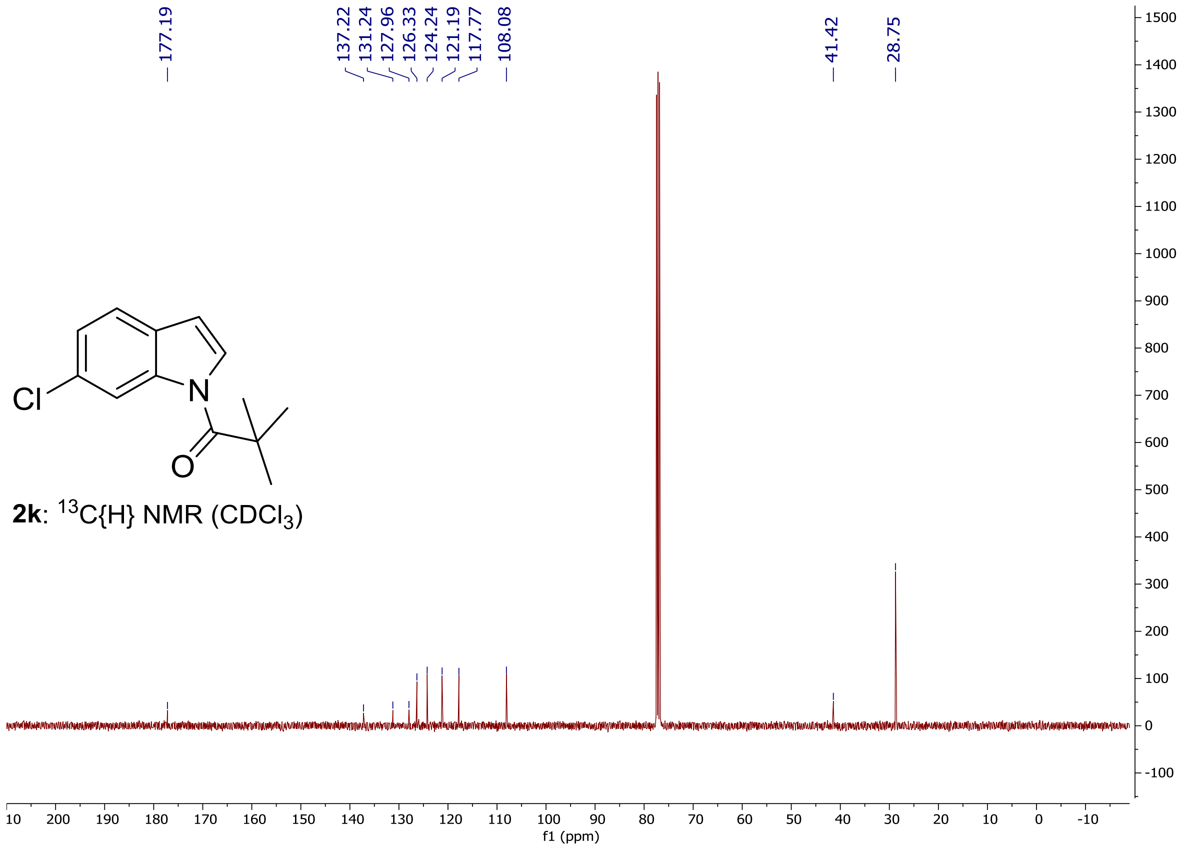

2k: ^{13}C{H} NMR ($CDCl_3$)

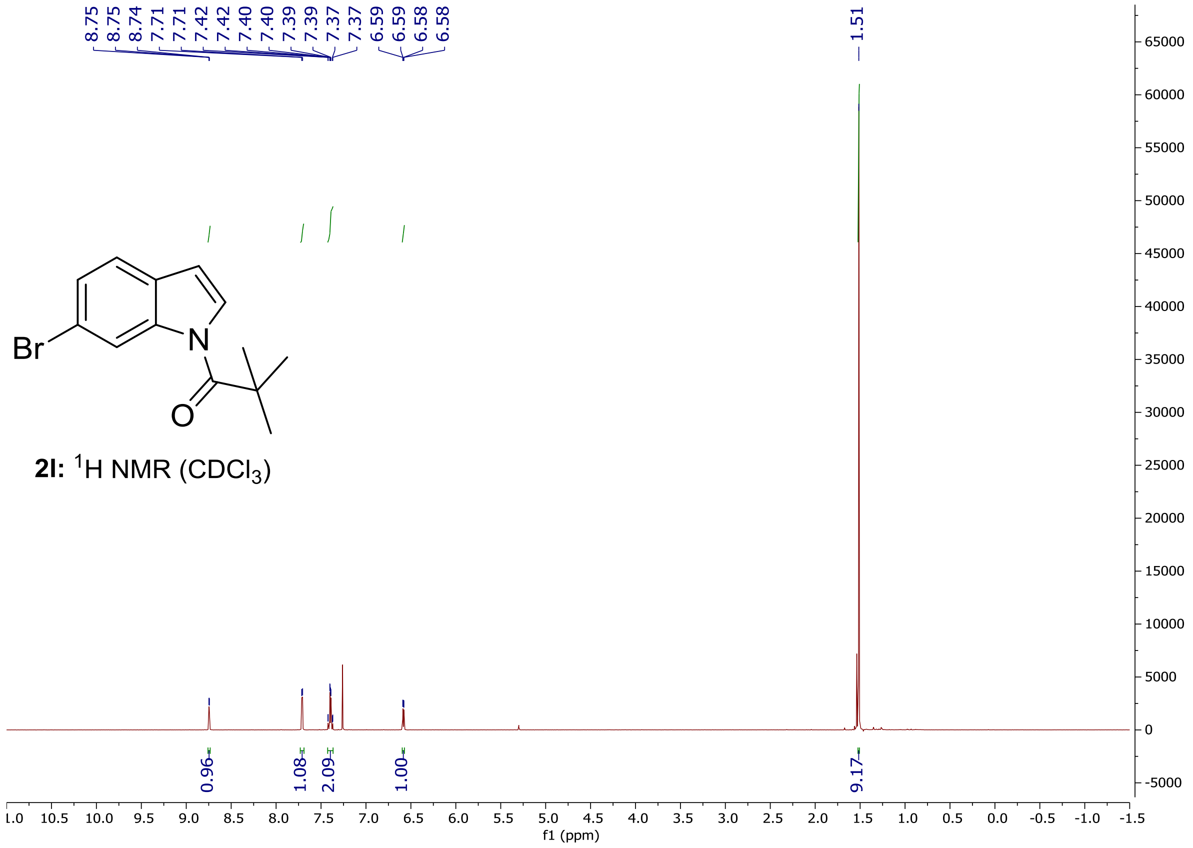
8.75 8.75 8.74 7.71 7.71 7.42 7.42 7.40 7.39 7.39 7.37 7.37 6.59 6.59 6.58 6.58
1.51
Br
N
O
2l: ¹H NMR (CDCl3)
0.96 1.08 2.09 1.00 9.17
f1 (ppm)

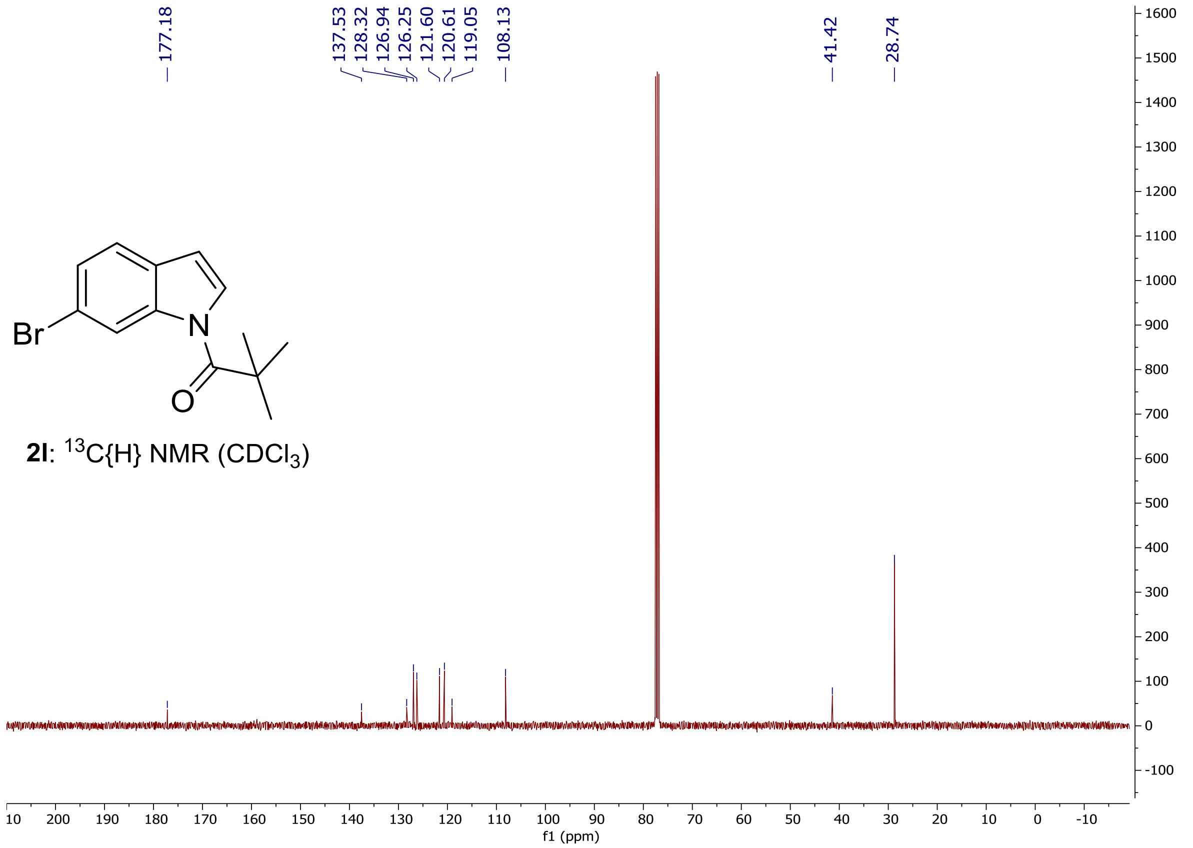
177.18
137.53 128.32 126.94 126.25 121.60 120.61 119.05
108.13
41.42
28.74
Br
N
O
2l: ¹³C{H} NMR (CDCl3)
f1 (ppm)

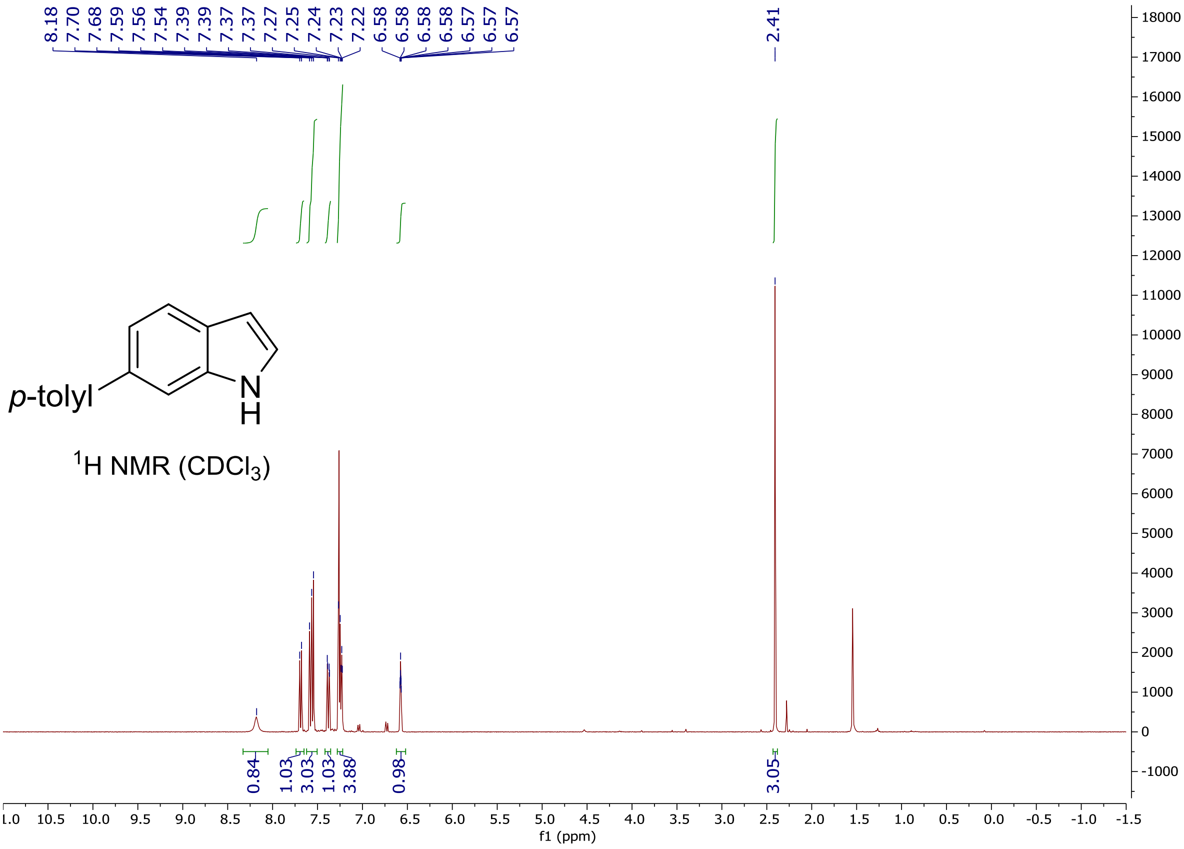
8.18 7.70 7.68 7.59 7.56 7.54 7.39 7.39 7.37 7.37 7.27 7.25 7.24 7.23 7.22 6.58 6.58 6.58 6.58 6.57 6.57 6.57
2.41
p-tolyl
N
H
1H NMR (CDCl3)
0.84 1.03 3.03 1.03 3.88 0.98 3.05
f1 (ppm)

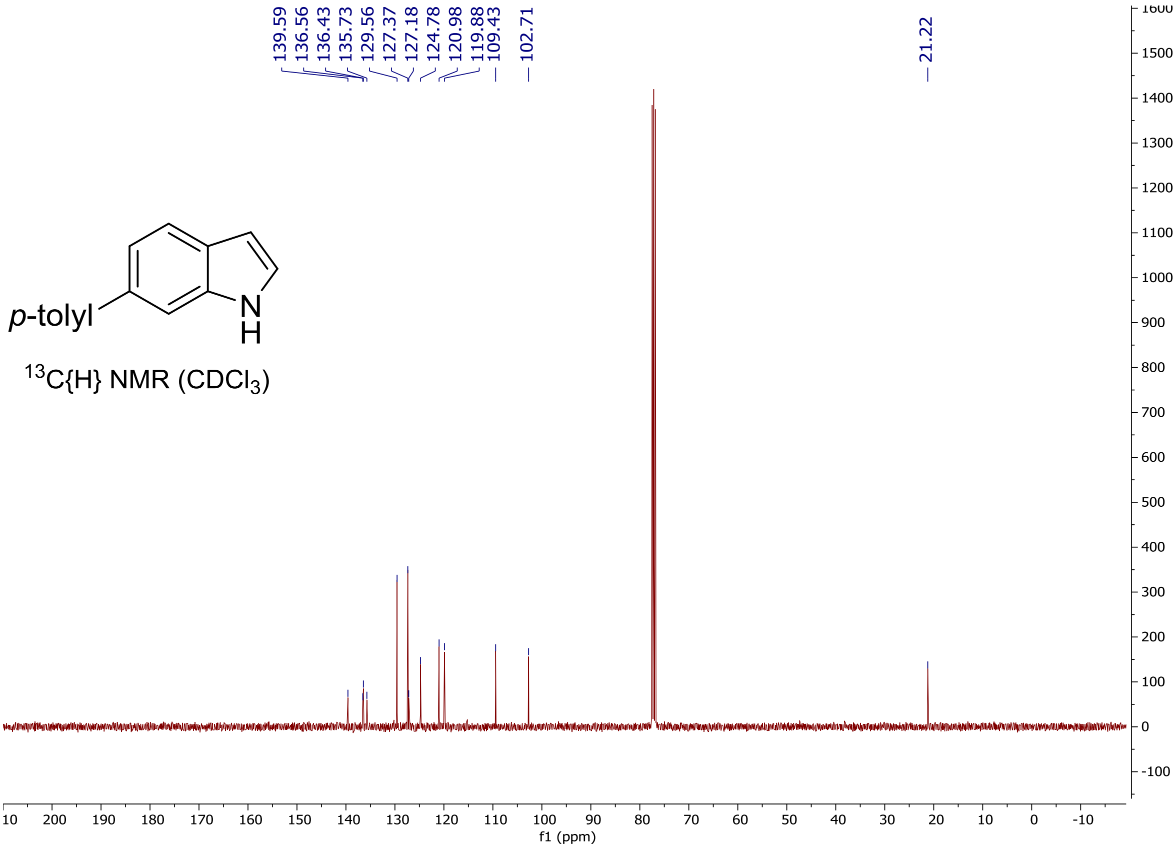
139.59 136.56 136.43 135.73 129.56 127.37 127.18 124.78 120.98 119.88 109.43 102.71
21.22
p-tolyl
N
H
13C{H} NMR (CDCl3)
f1 (ppm)

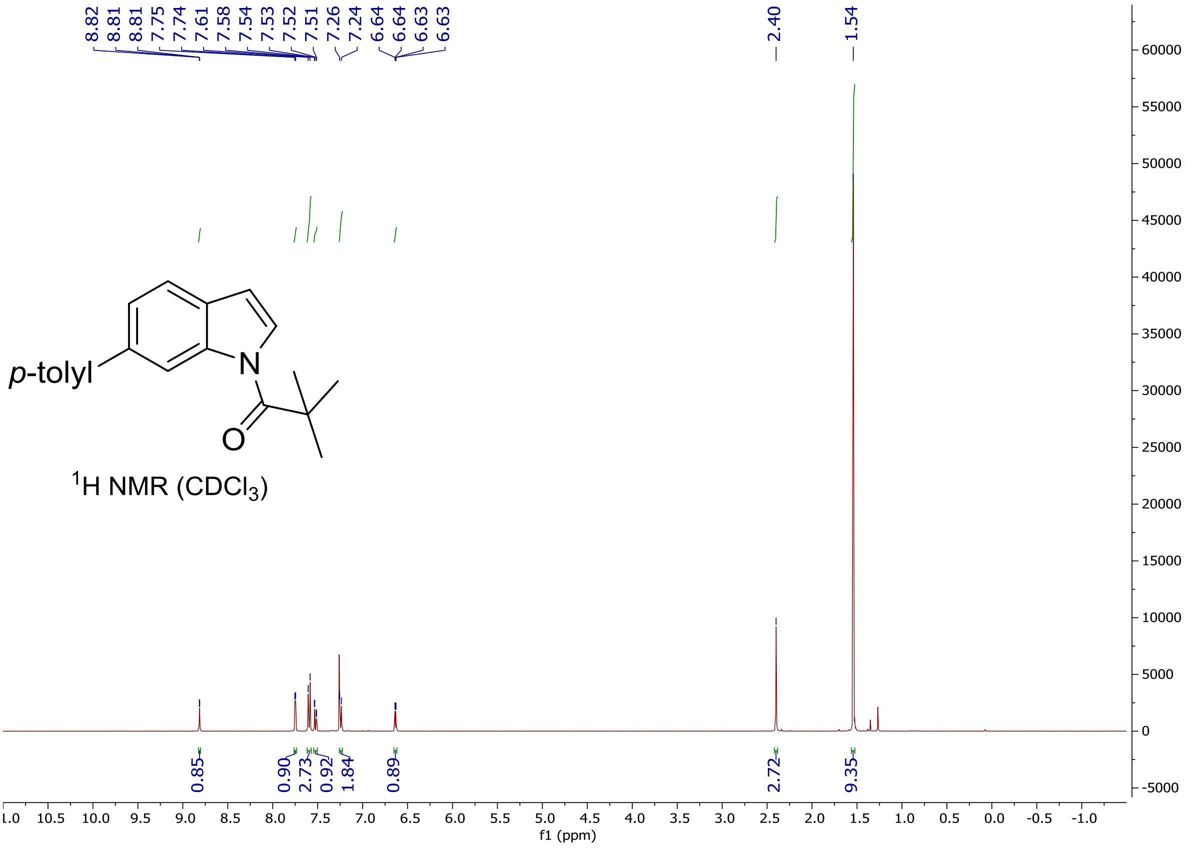

8.82 8.81 8.81 7.75 7.74 7.61 7.58 7.54 7.53 7.52 7.51 7.26 7.24 6.64 6.64 6.63 6.63
2.40
1.54
p-tolyl
N
O
^{1}H NMR (CDCl$_3$)
0.85 0.90 2.73 0.92 1.84 0.89 2.72 9.35
f1 (ppm)

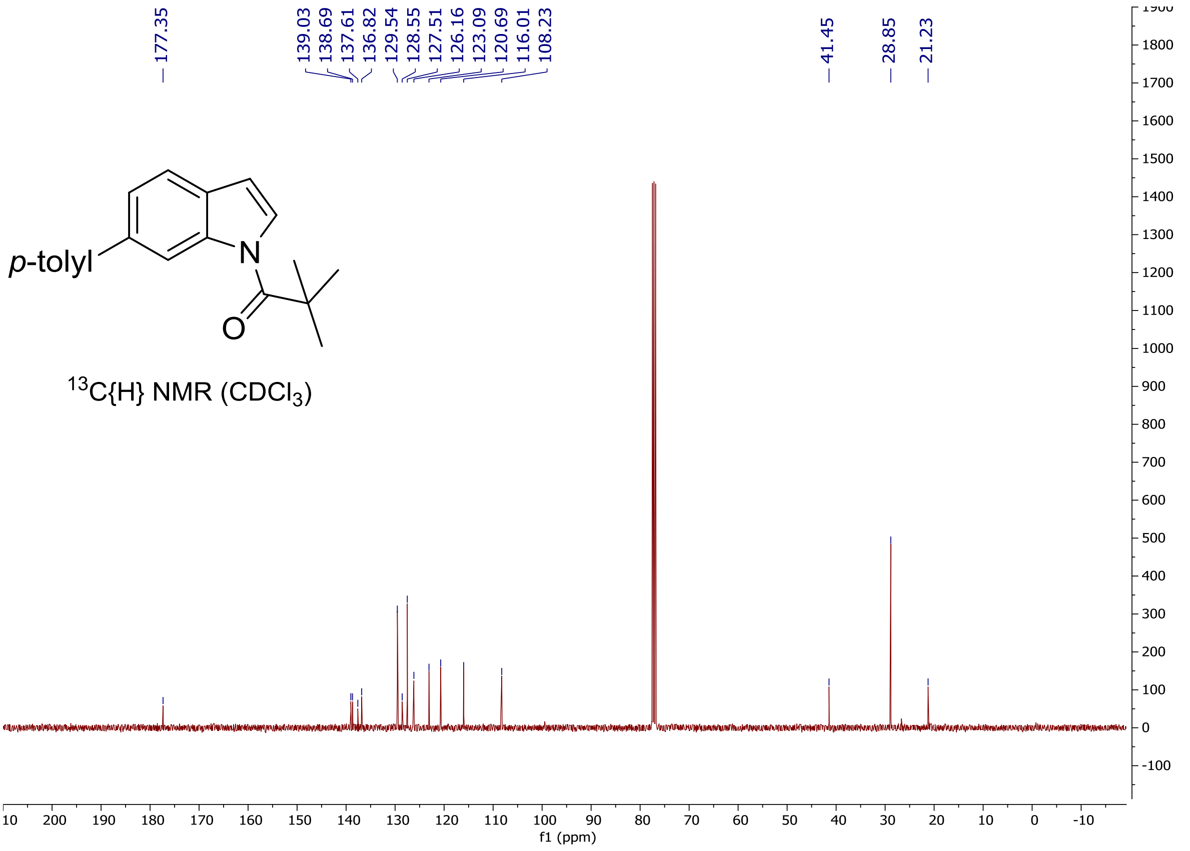

177.35
139.03 138.69 137.61 136.82 129.54 128.55 127.51 126.16 123.09 120.69 116.01 108.23
41.45
28.85
21.23
p-tolyl
N
O
^{13}C{H} NMR (CDCl$_3$)
f1 (ppm)

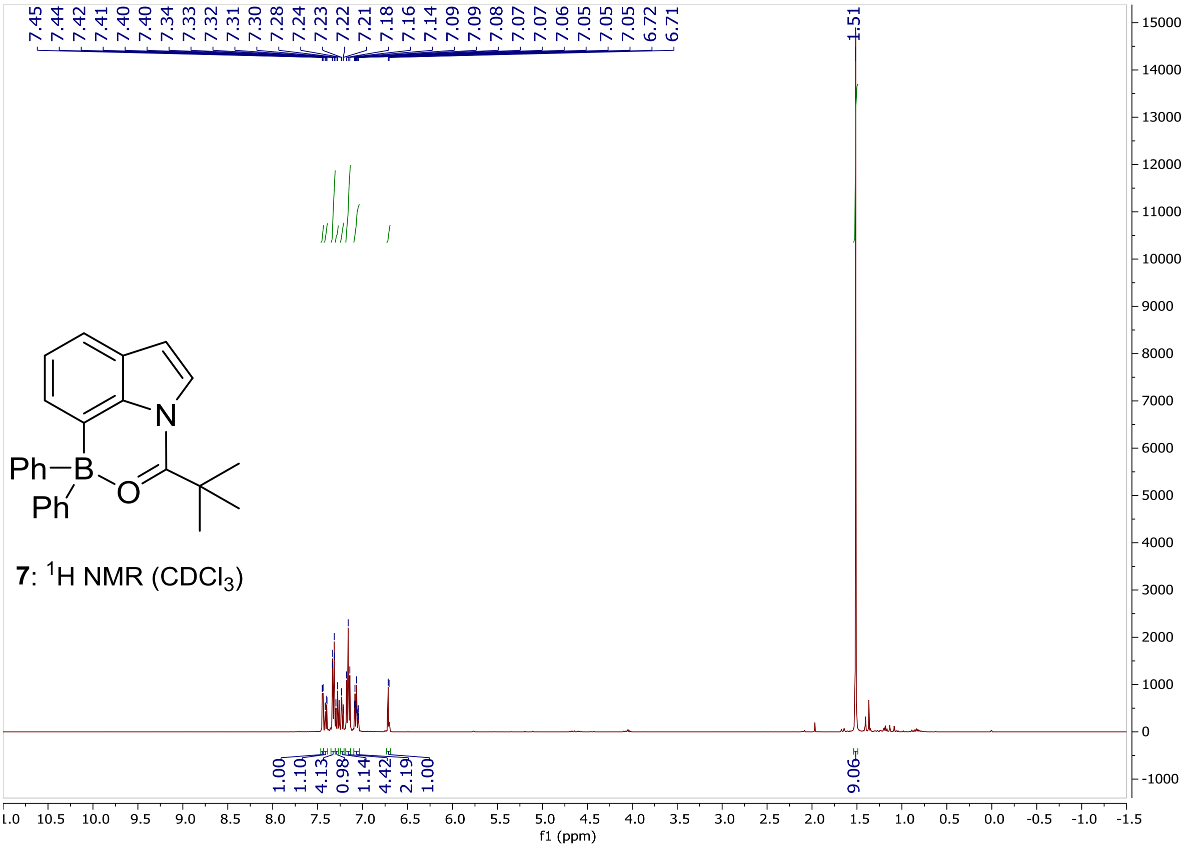
7.45
7.44
7.42
7.41
7.40
7.40
7.34
7.33
7.32
7.31
7.30
7.28
7.24
7.23
7.22
7.21
7.18
7.16
7.14
7.09
7.09
7.08
7.07
7.07
7.06
7.05
7.05
7.05
6.72
6.71
1.51
Ph
B
Ph
O
N
7: 1H NMR (CDCl3)
1.00
1.10
4.13
0.98
1.14
4.42
2.19
1.00
9.06
f1 (ppm)

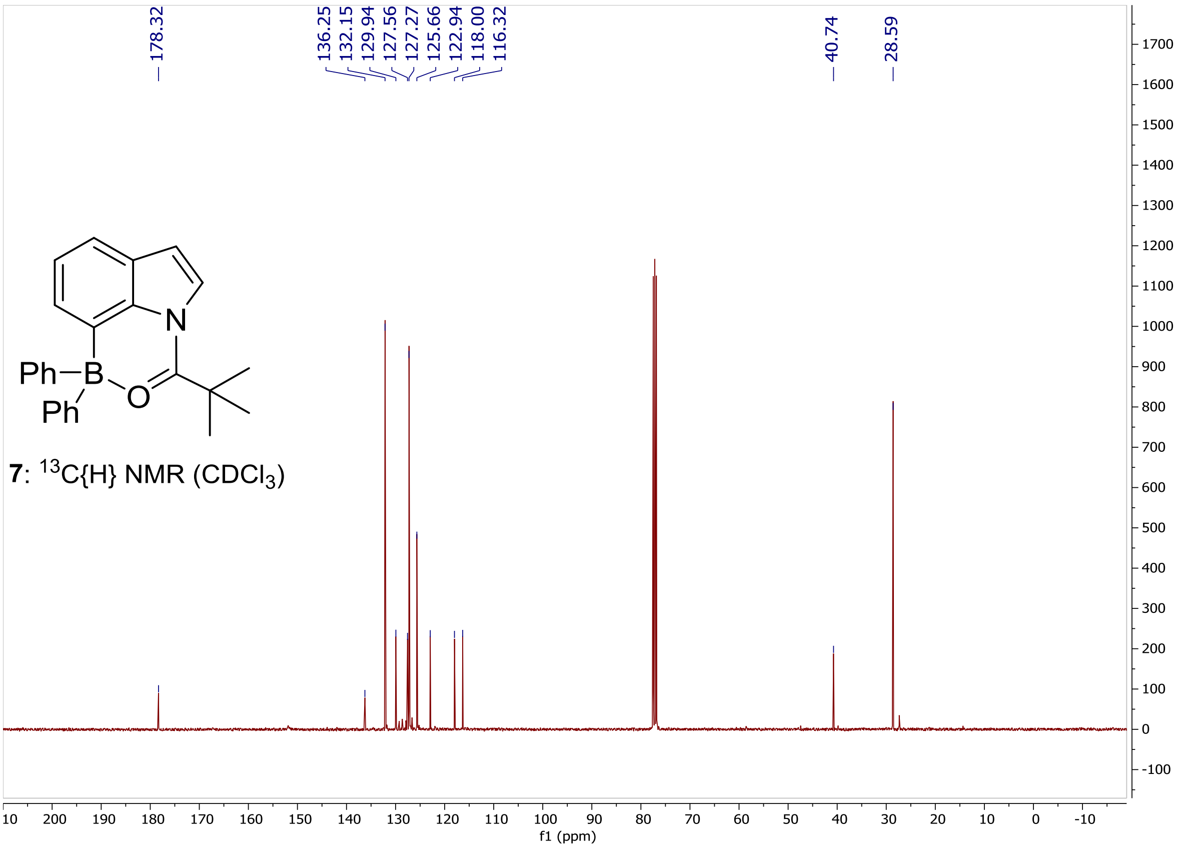
178.32
136.25
132.15
129.94
127.56
127.27
125.66
122.94
118.00
116.32
40.74
28.59
Ph
B
Ph
O
N
7: 13C{H} NMR (CDCl3)
f1 (ppm)

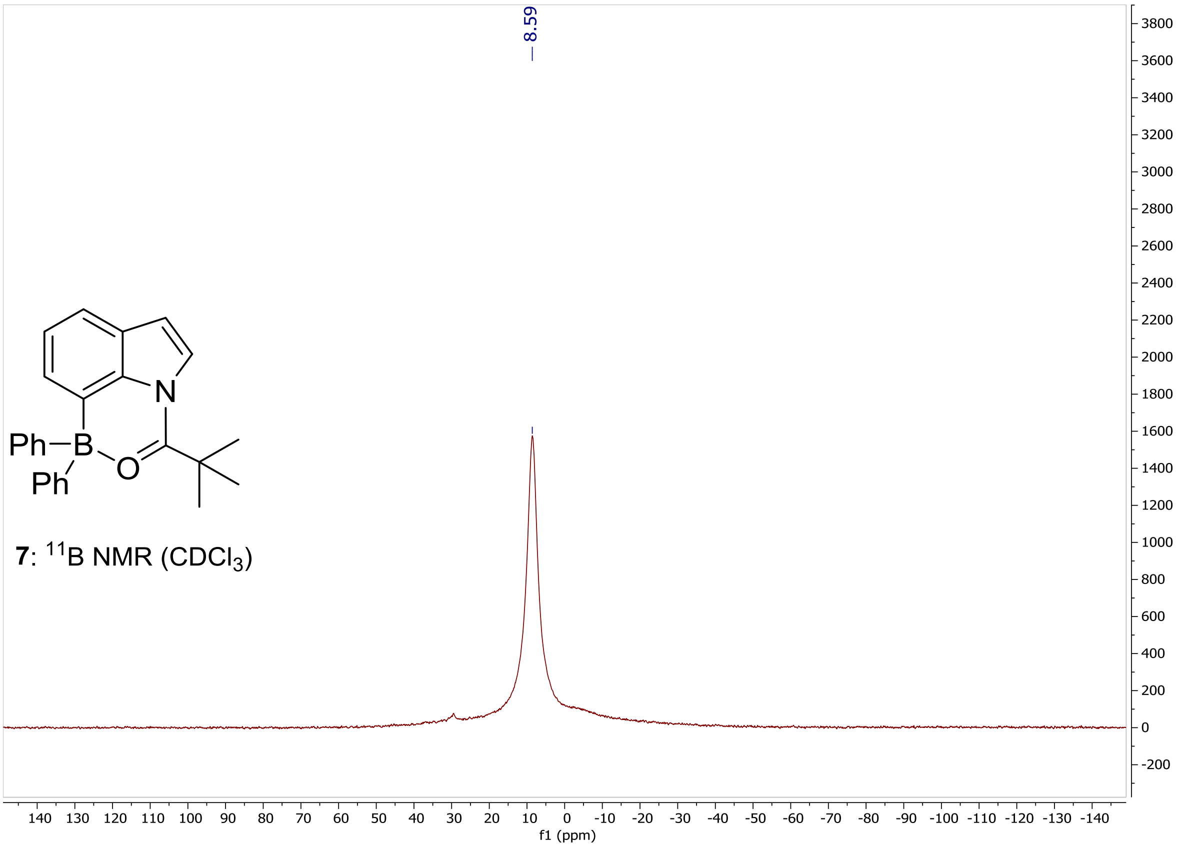
8.59
7: ¹¹B NMR (CDCl3)
Ph
B
Ph
O
N
f1 (ppm)

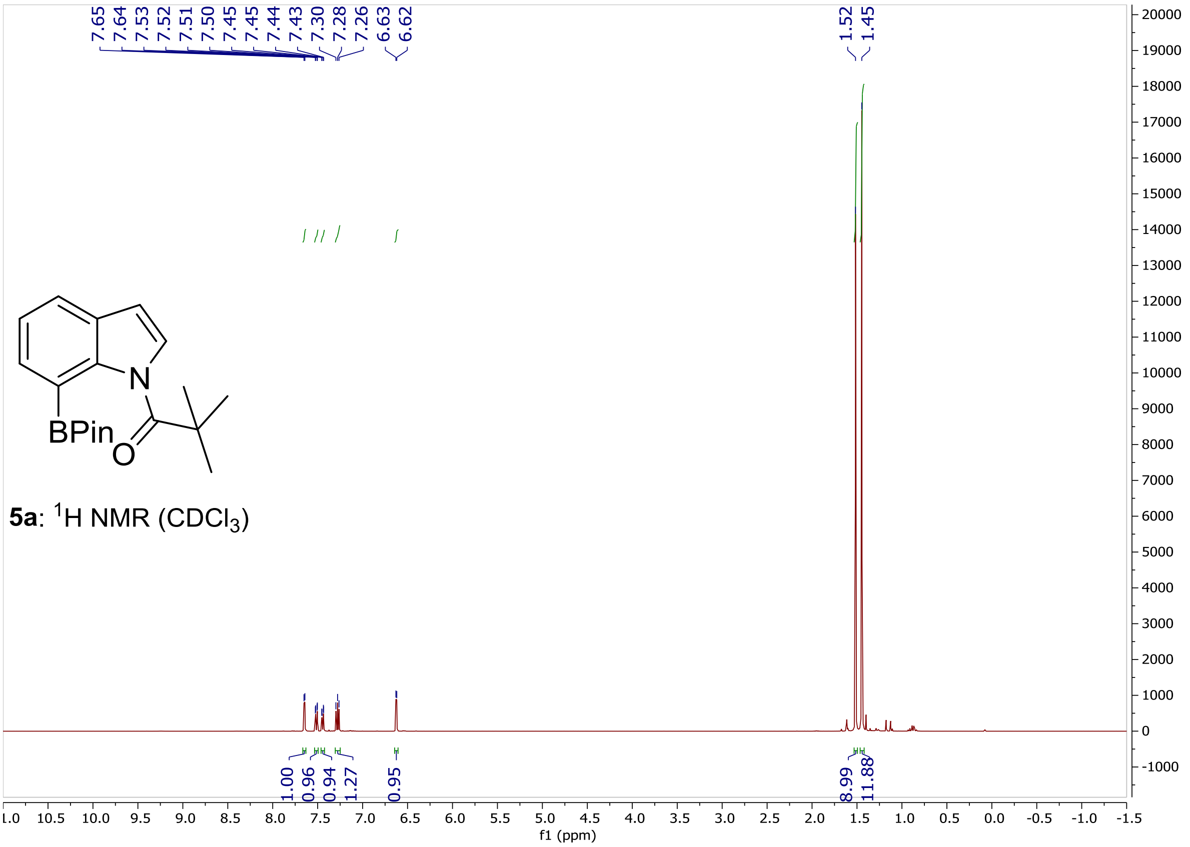
7.65
7.64
7.53
7.52
7.51
7.50
7.45
7.45
7.44
7.43
7.30
7.28
7.26
6.63
6.62
1.52
1.45
1.00
0.96
0.94
1.27
0.95
8.99
11.88
BPin
N
O
5a: 1H NMR (CDCl3)
f1 (ppm)

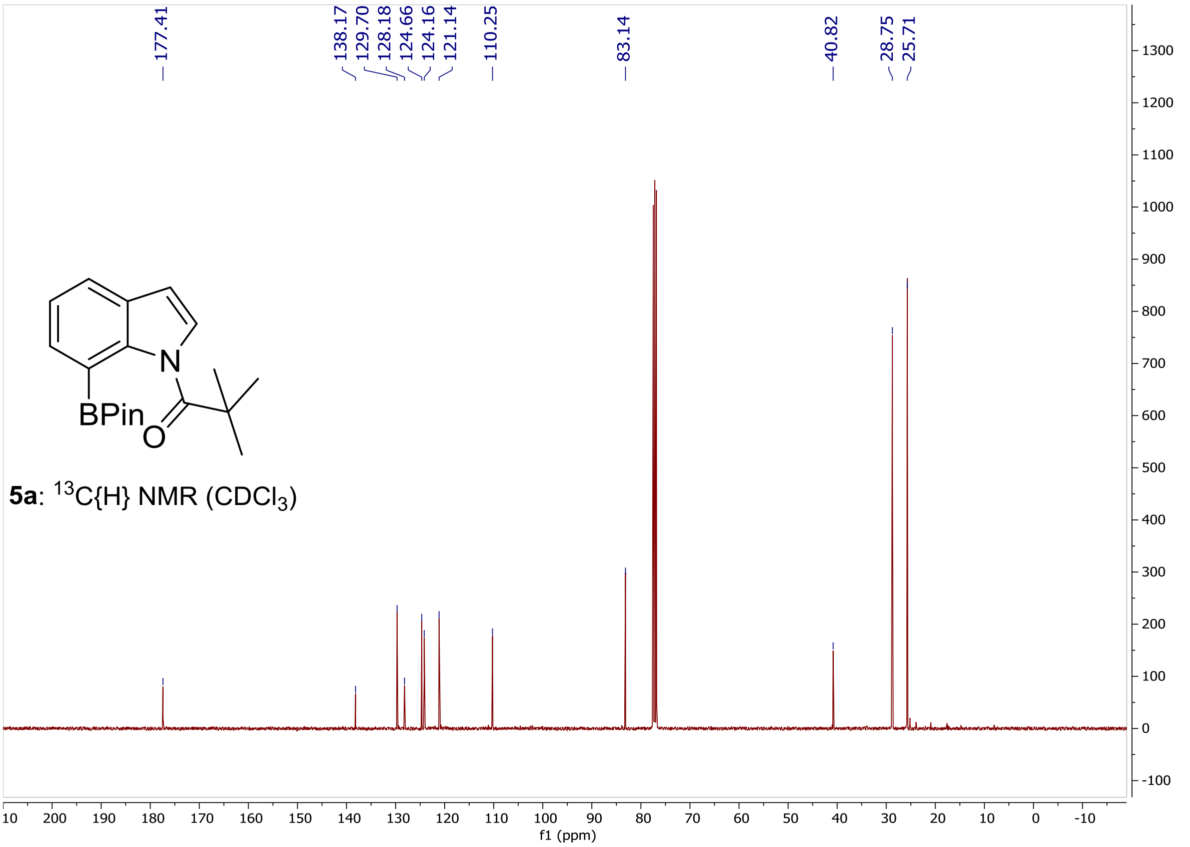
177.41
138.17
129.70
128.18
124.66
124.16
121.14
110.25
83.14
40.82
28.75
25.71
BPin
N
O
5a: 13C{H} NMR (CDCl3)
f1 (ppm)

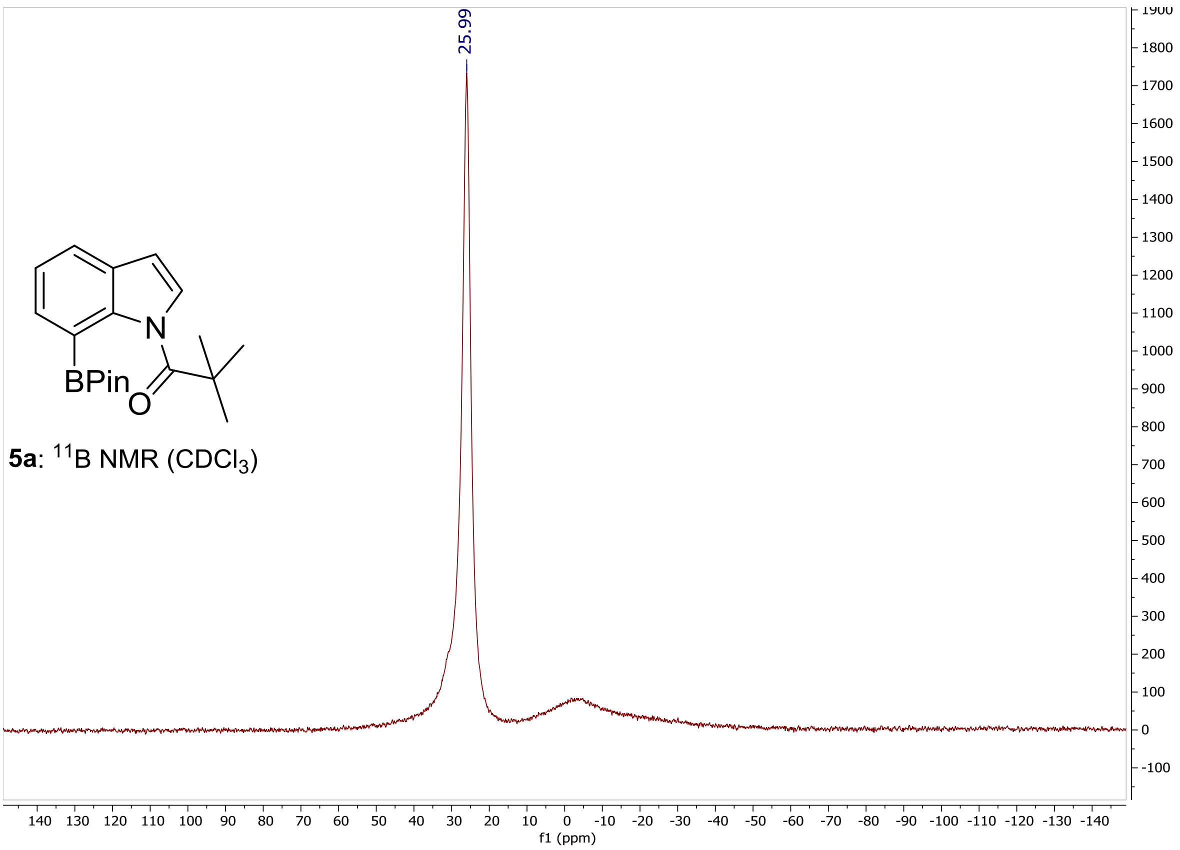
25.99
BPin
N
O
5a: ^{11}B NMR ($CDCl_3$)
f1 (ppm)

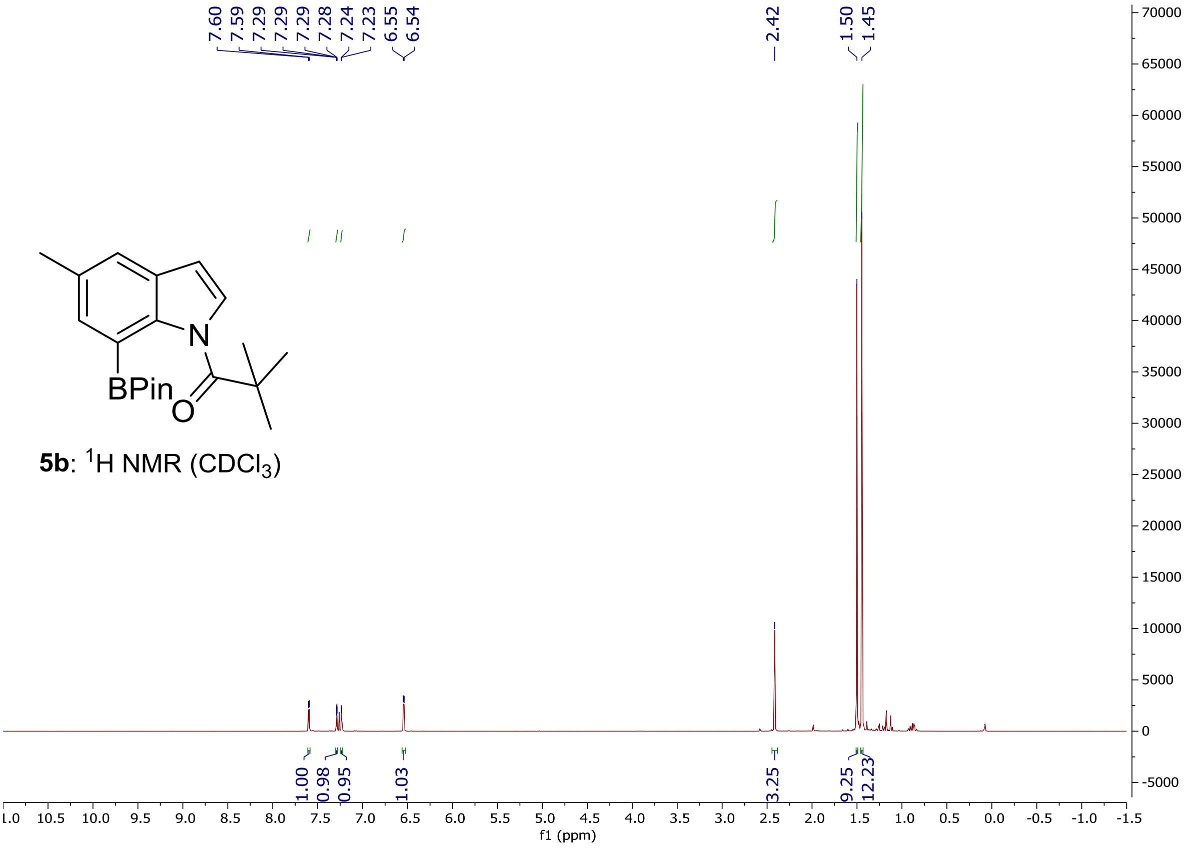
5b: ^{1}H NMR ($CDCl_3$)
BPin
N
O
7.60 7.59 7.29 7.29 7.29 7.28 7.24 7.23 6.55 6.54
2.42
1.50 1.45
1.00 0.98 0.95 1.03 3.25 9.25 12.23
f1 (ppm)

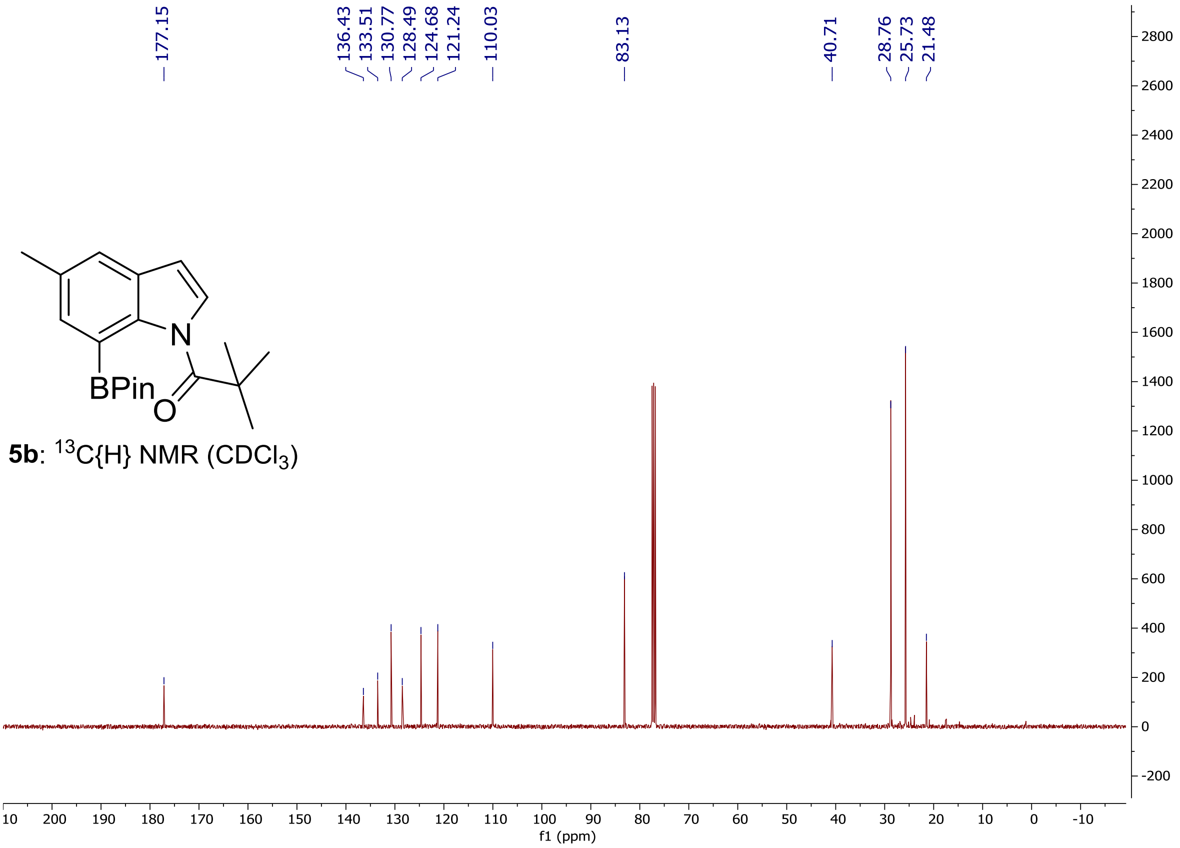
5b: ^{13}C{H} NMR ($CDCl_3$)
BPin
N
O
177.15
136.43 133.51 130.77 128.49 124.68 121.24 110.03
83.13
40.71
28.76 25.73 21.48
f1 (ppm)

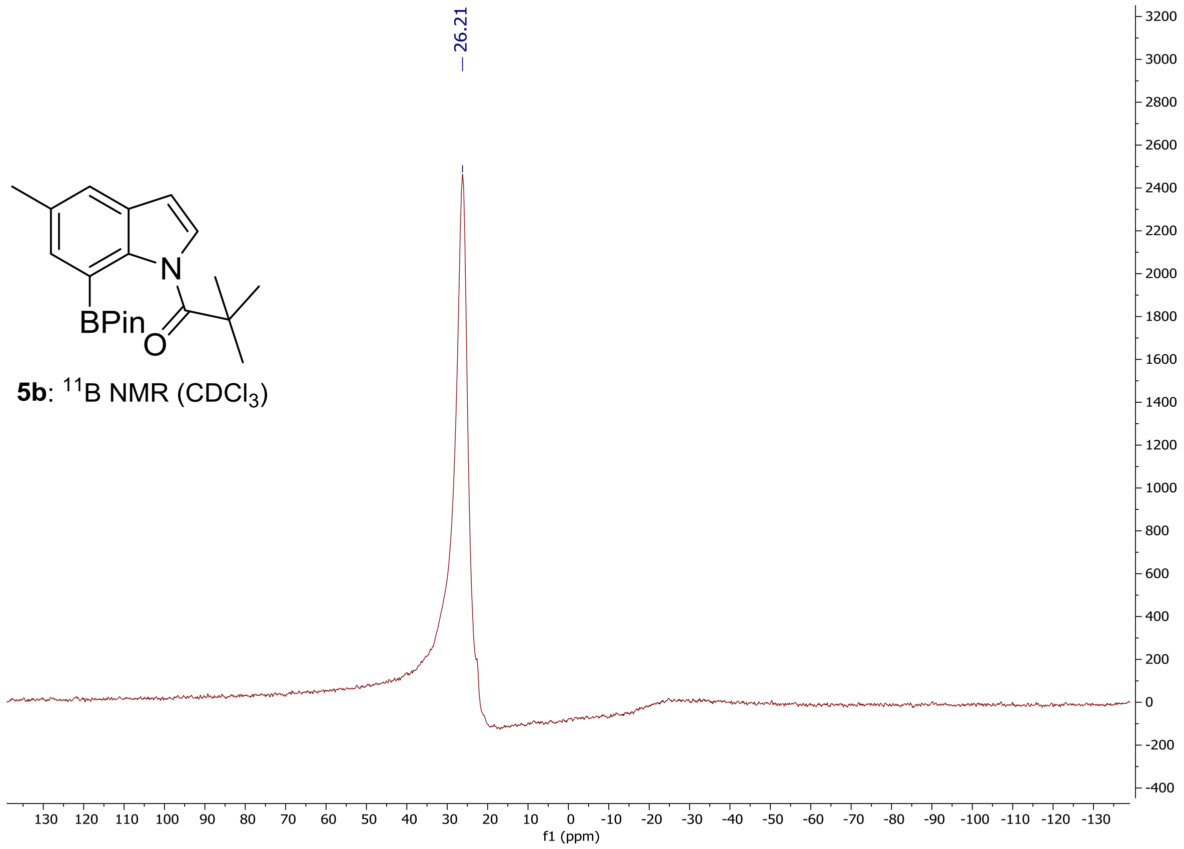
26.21
BPin
N
O
5b: 11B NMR (CDCl3)
f1 (ppm)

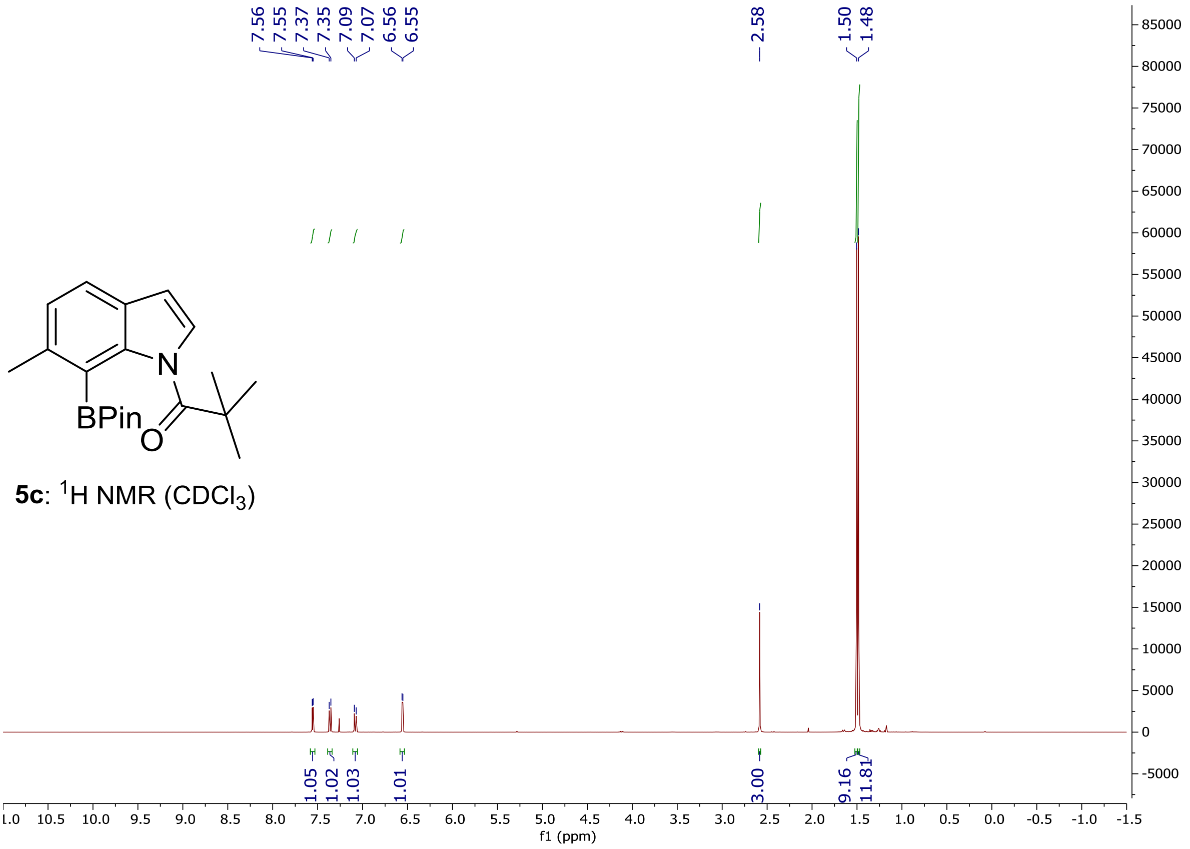
7.56
7.55
7.37
7.35
7.09
7.07
6.56
6.55
2.58
1.50
1.48
N
BPin
O
5c: 1H NMR (CDCl3)
1.05
1.02
1.03
1.01
3.00
9.16
11.81
f1 (ppm)

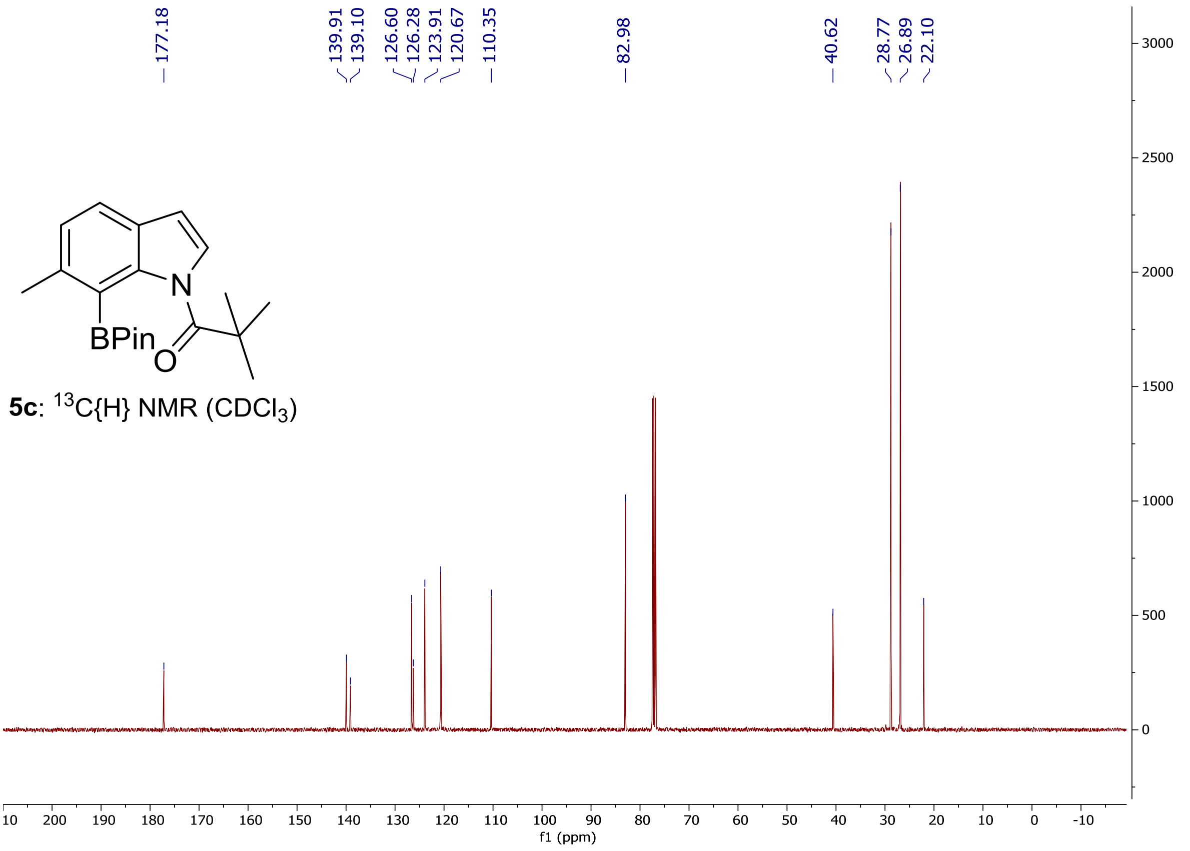
177.18
139.91
139.10
126.60
126.28
123.91
120.67
110.35
82.98
40.62
28.77
26.89
22.10
N
BPin
O
5c: 13C{H} NMR (CDCl3)
f1 (ppm)

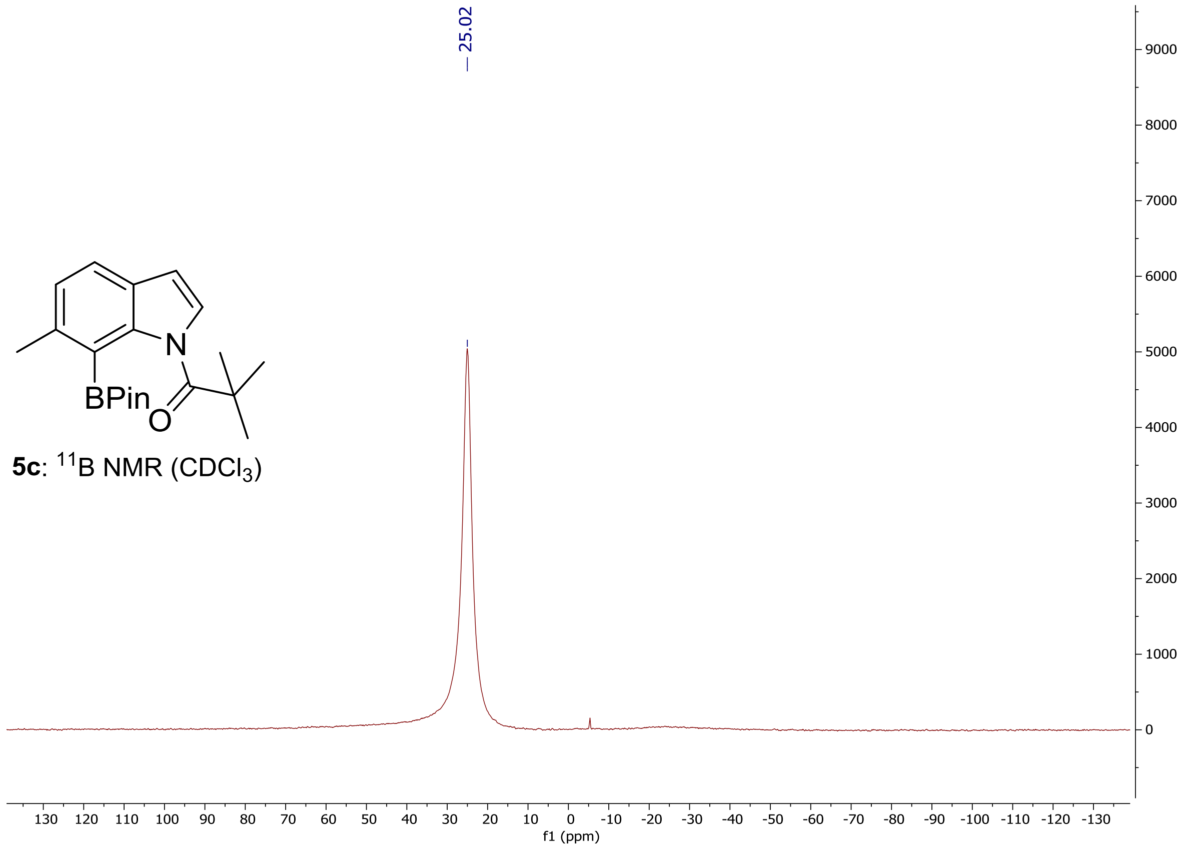

5c: ^{11}B NMR ($CDCl_3$)

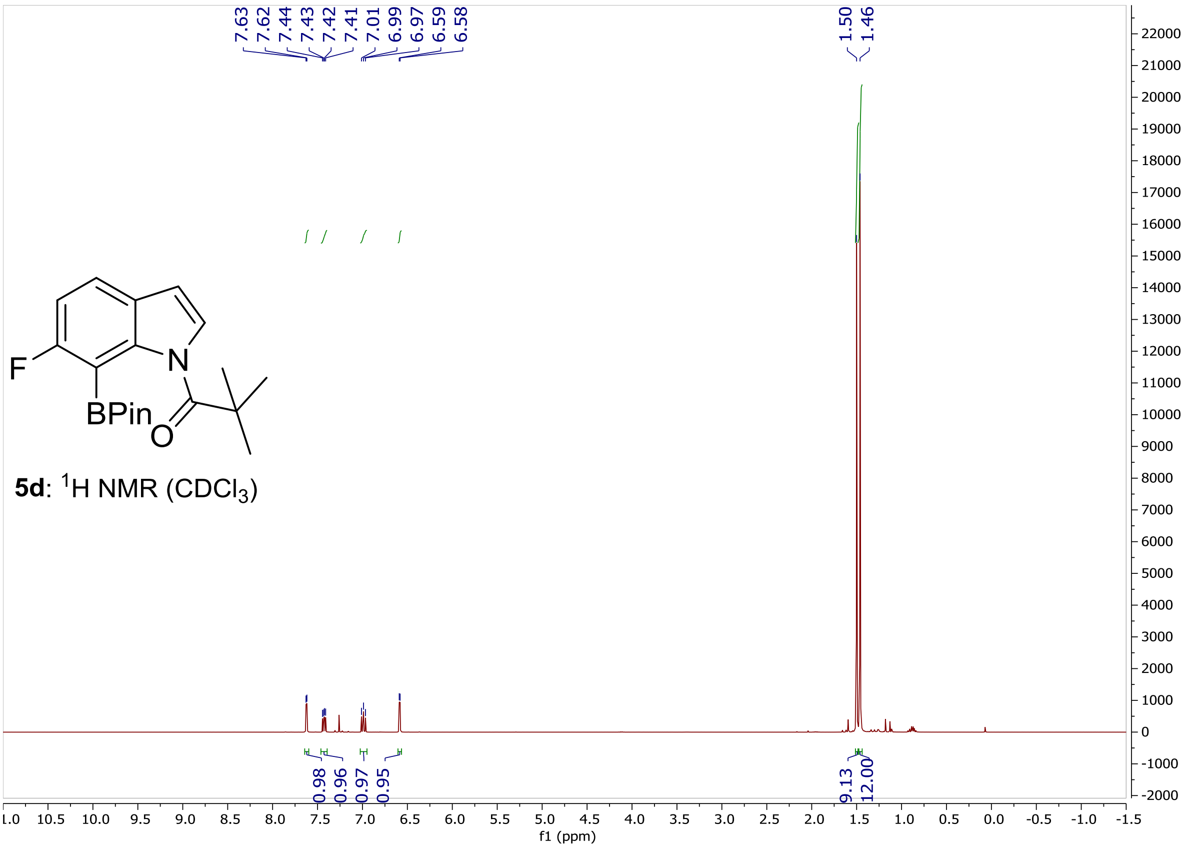

5d: ^{1}H NMR ($CDCl_3$)

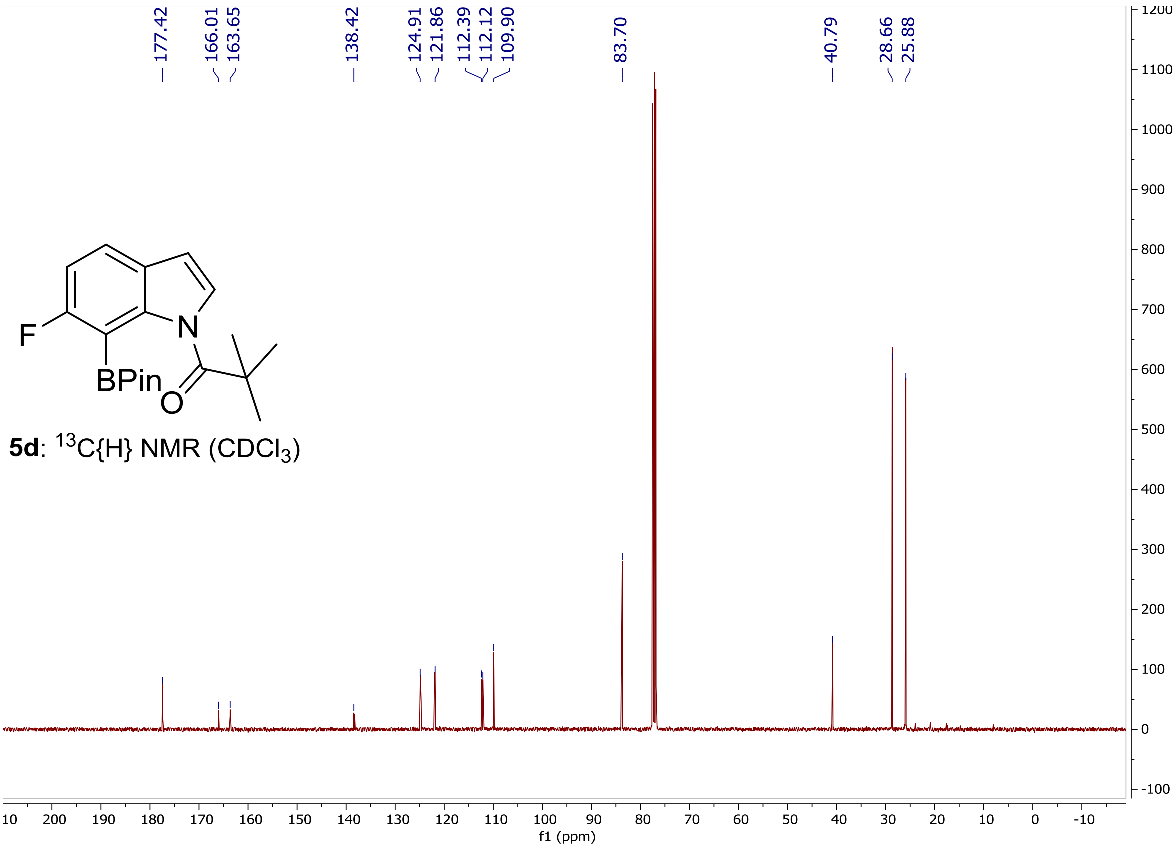

5d: ^{13}C{H} NMR ($CDCl_3$)

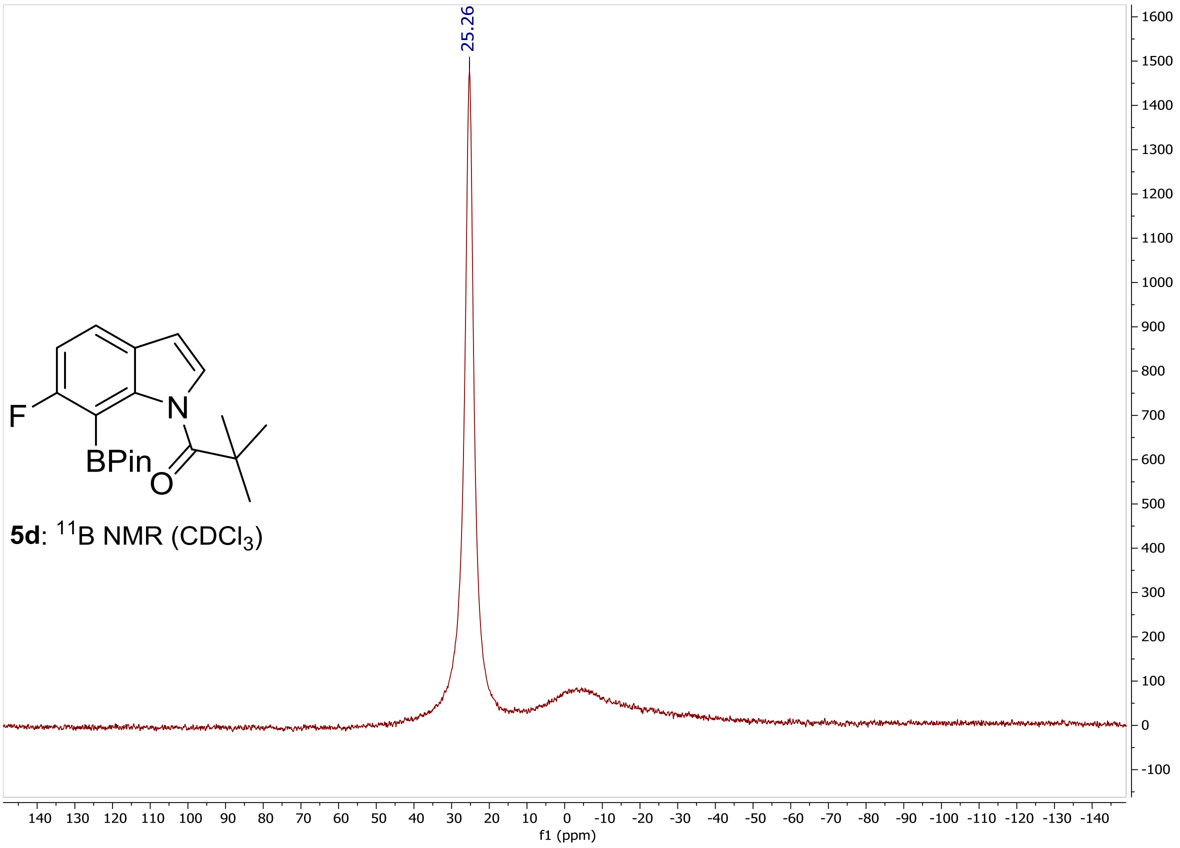

5d: ^{11}B NMR ($CDCl_3$)

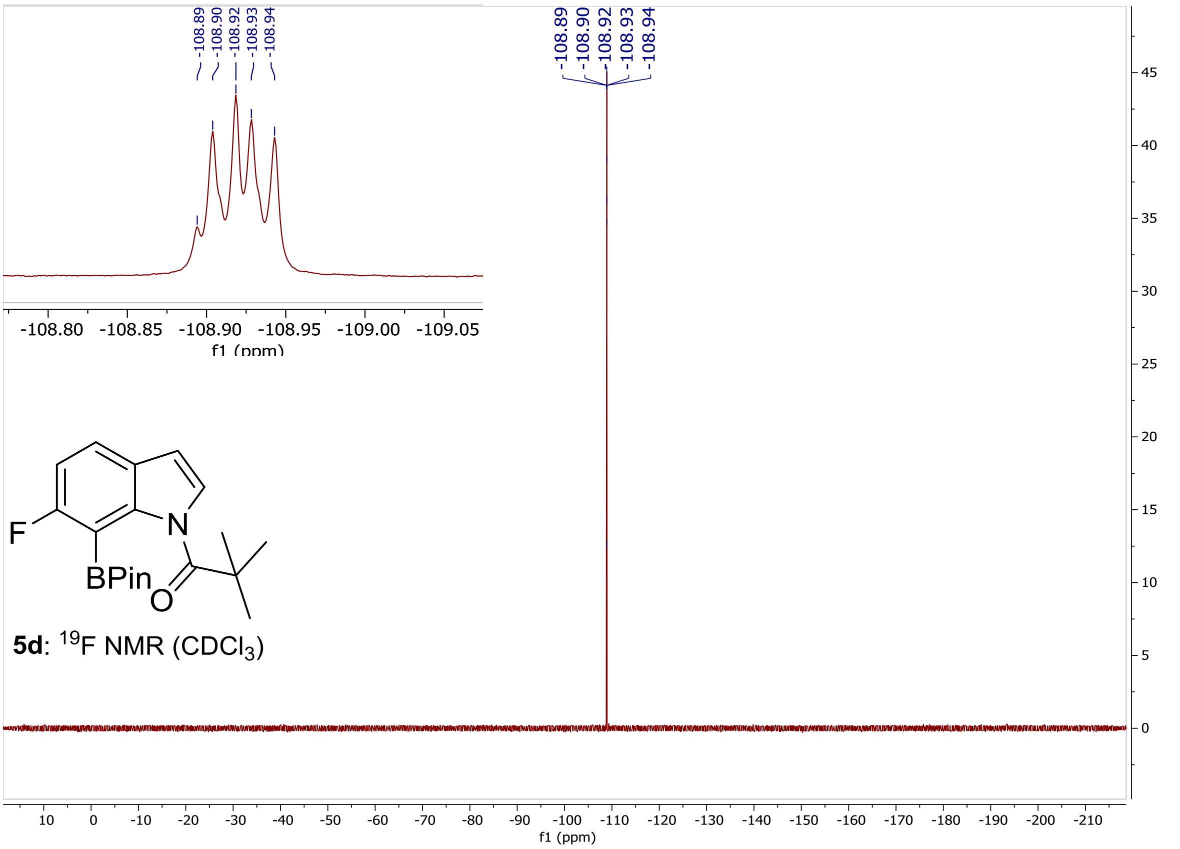

5d: ^{19}F NMR ($CDCl_3$)

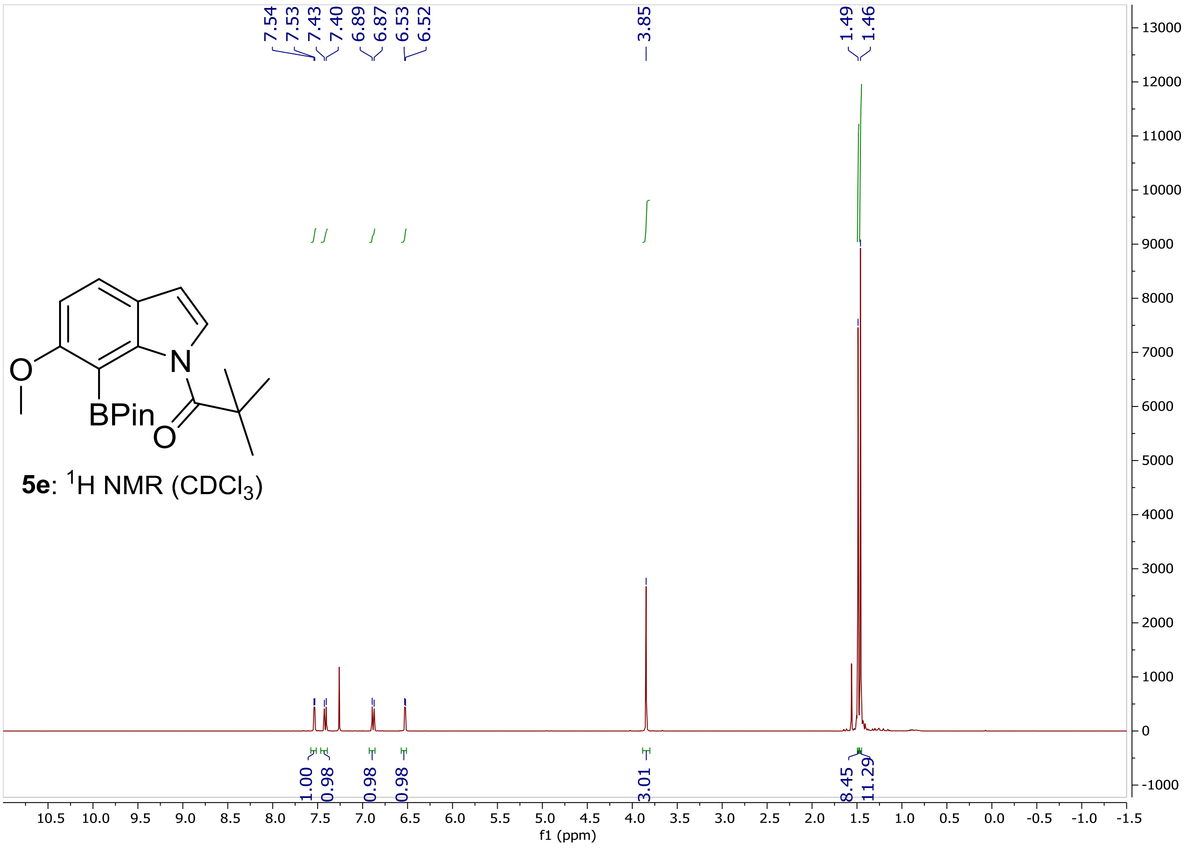

5e: ^{1}H NMR ($CDCl_3$)

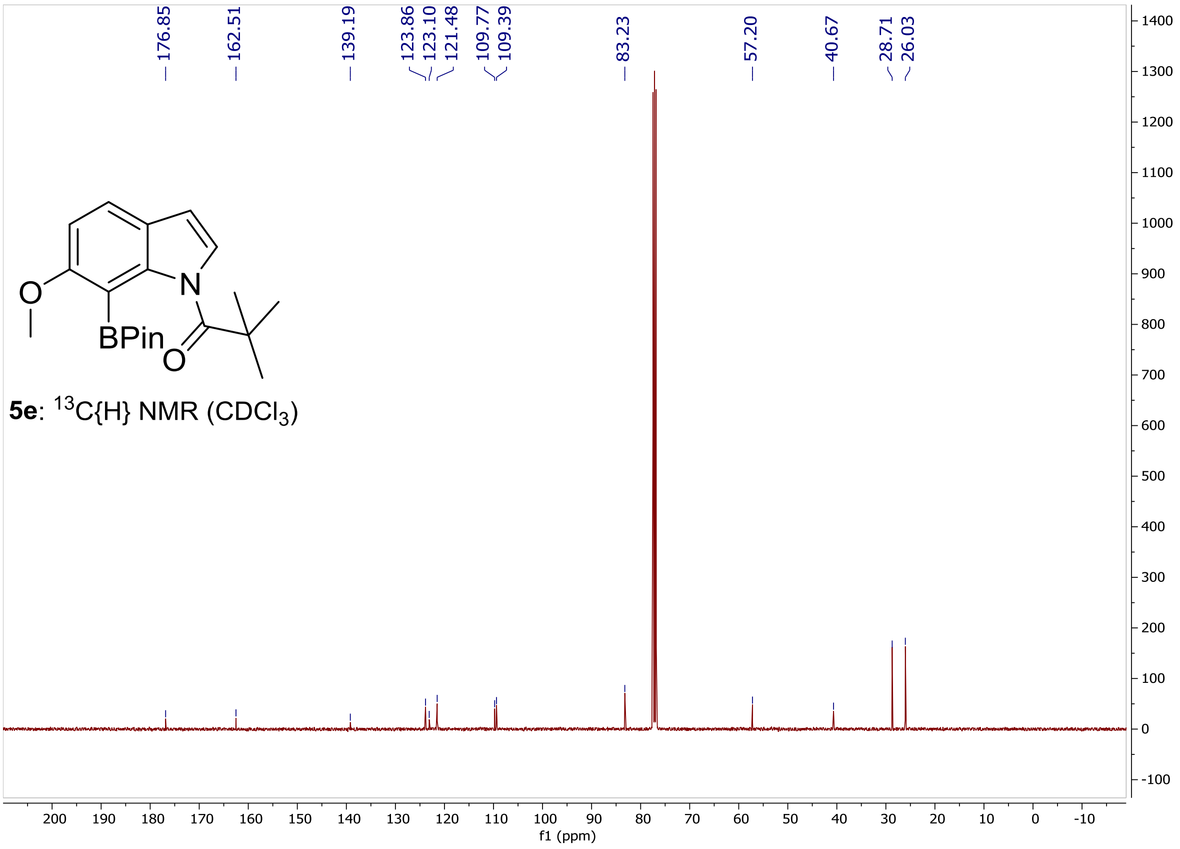

5e: ^{13}C{H} NMR ($CDCl_3$)

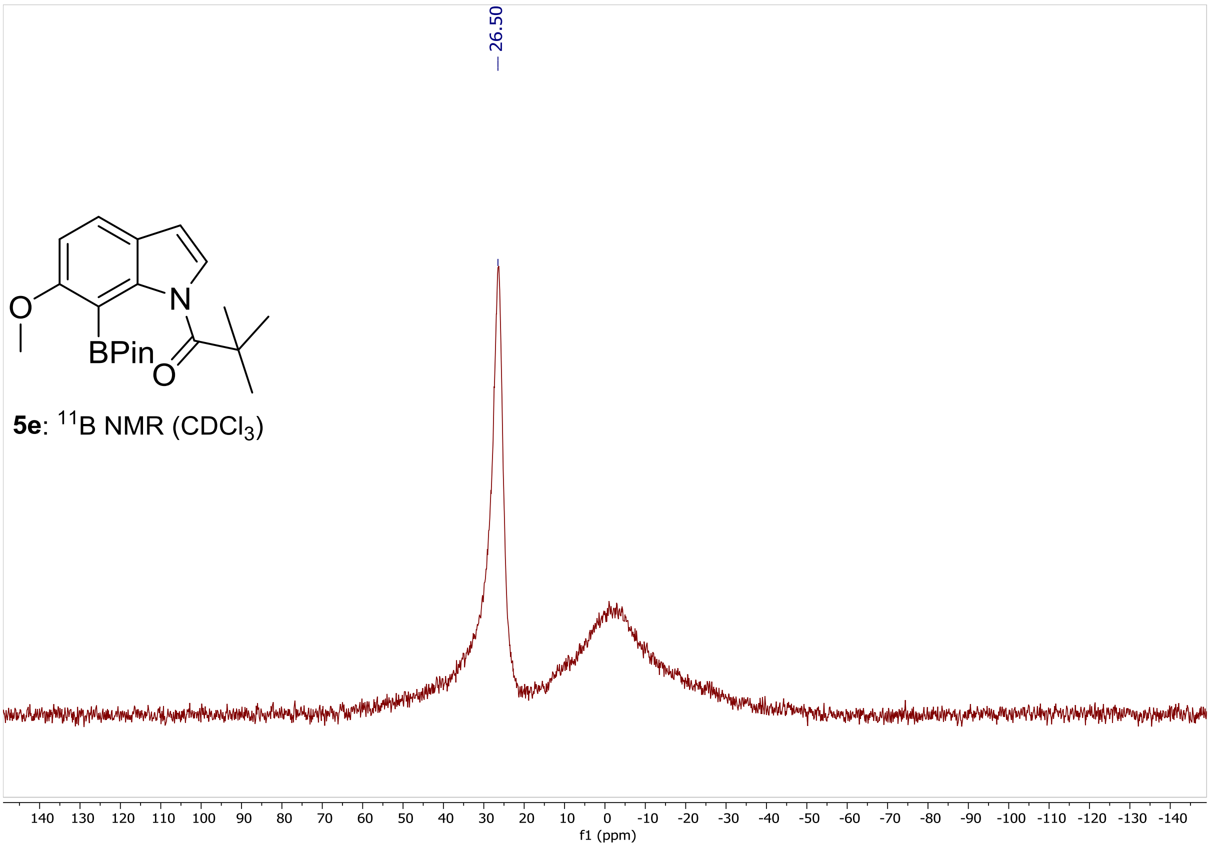

O
N
BPin
O
5e: 11B NMR (CDCl3)
26.50
140 130 120 110 100 90 80 70 60 50 40 30 20 10 0 -10 -20 -30 -40 -50 -60 -70 -80 -90 -100 -110 -120 -130 -140
f1 (ppm)

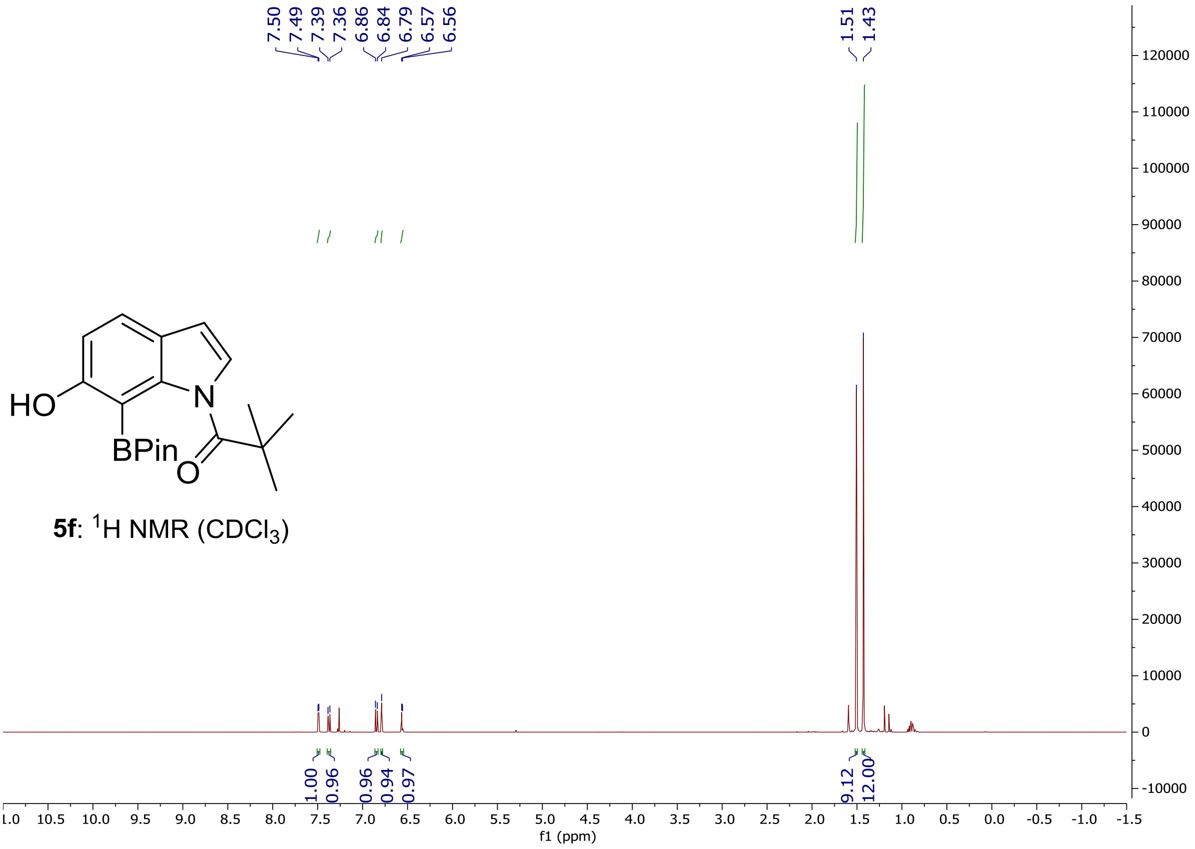

5f: ^{1}H NMR ($CDCl_3$)

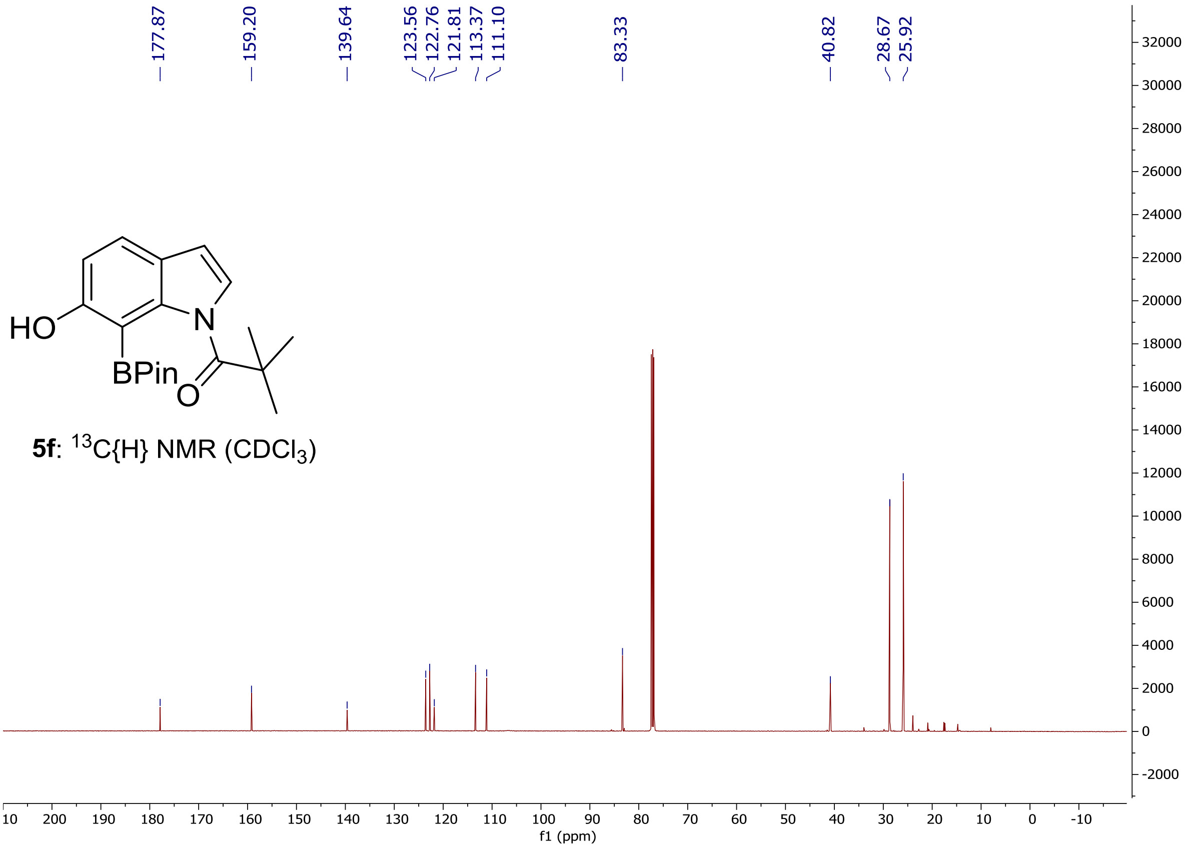

5f: $^{13}C\{H\}$ NMR ($CDCl_3$)

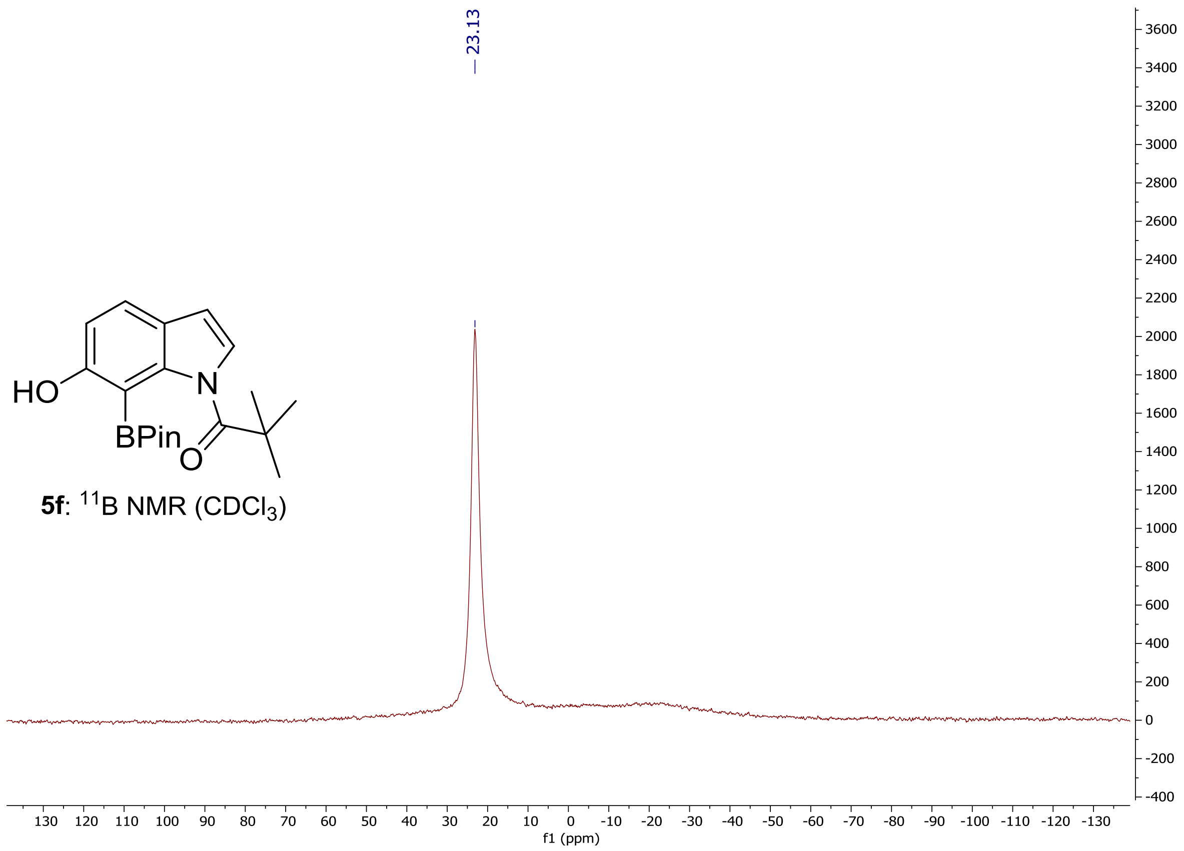

5f: ^{11}B NMR ($CDCl_3$)

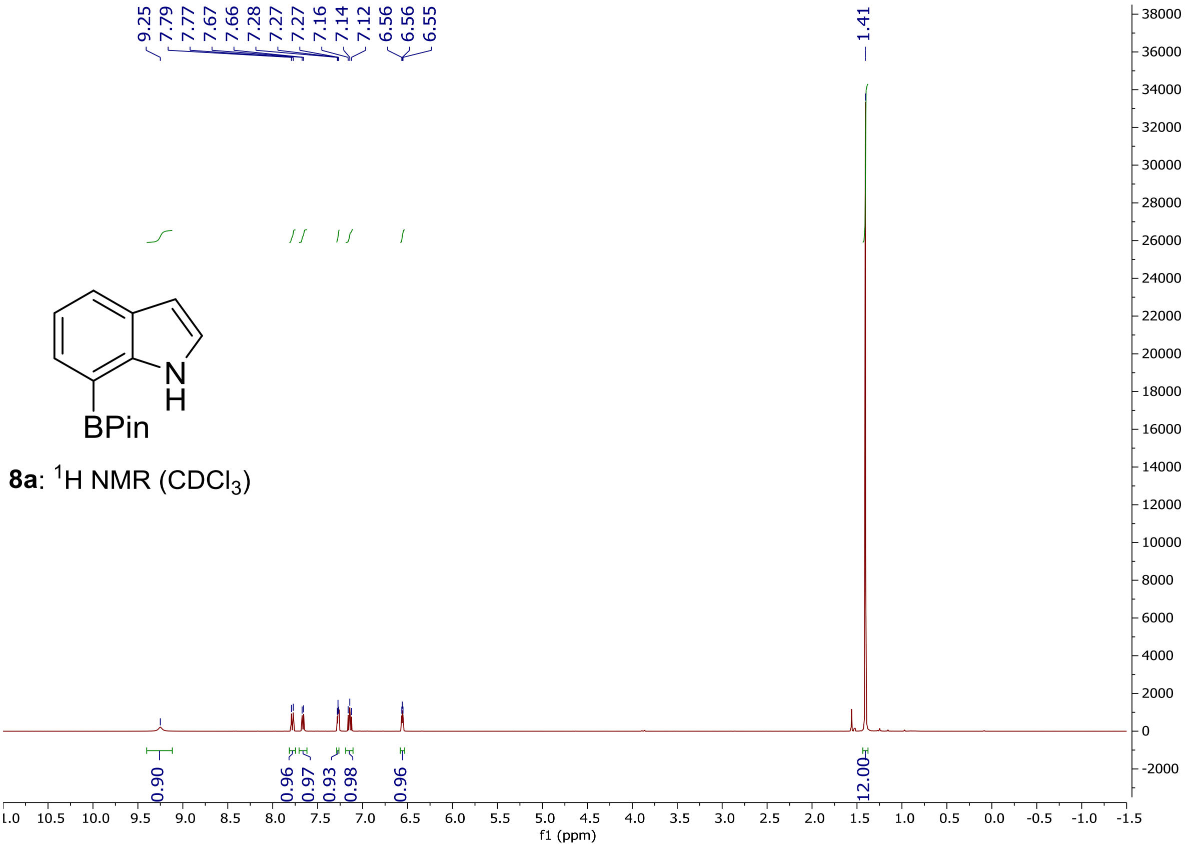

8a: ^{1}H NMR ($CDCl_3$)

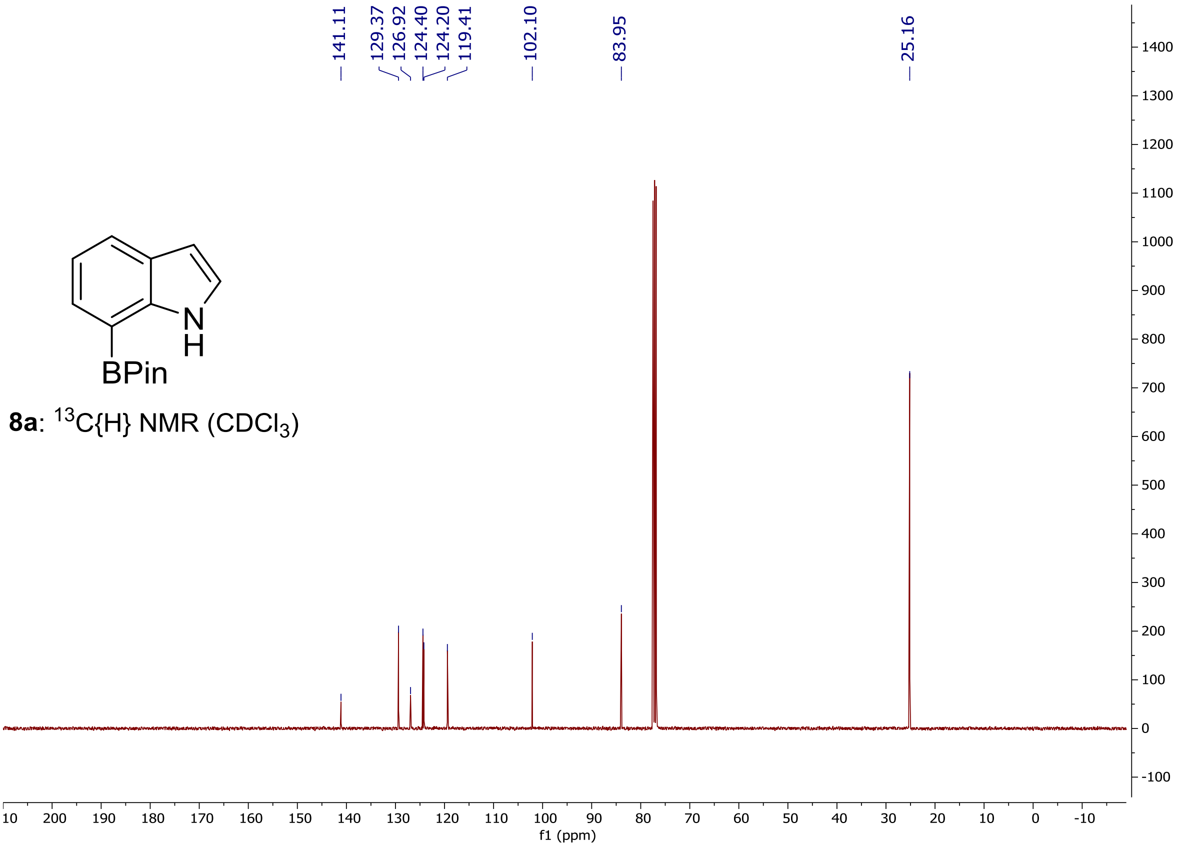

8a: $^{13}C\{H\}$ NMR ($CDCl_3$)

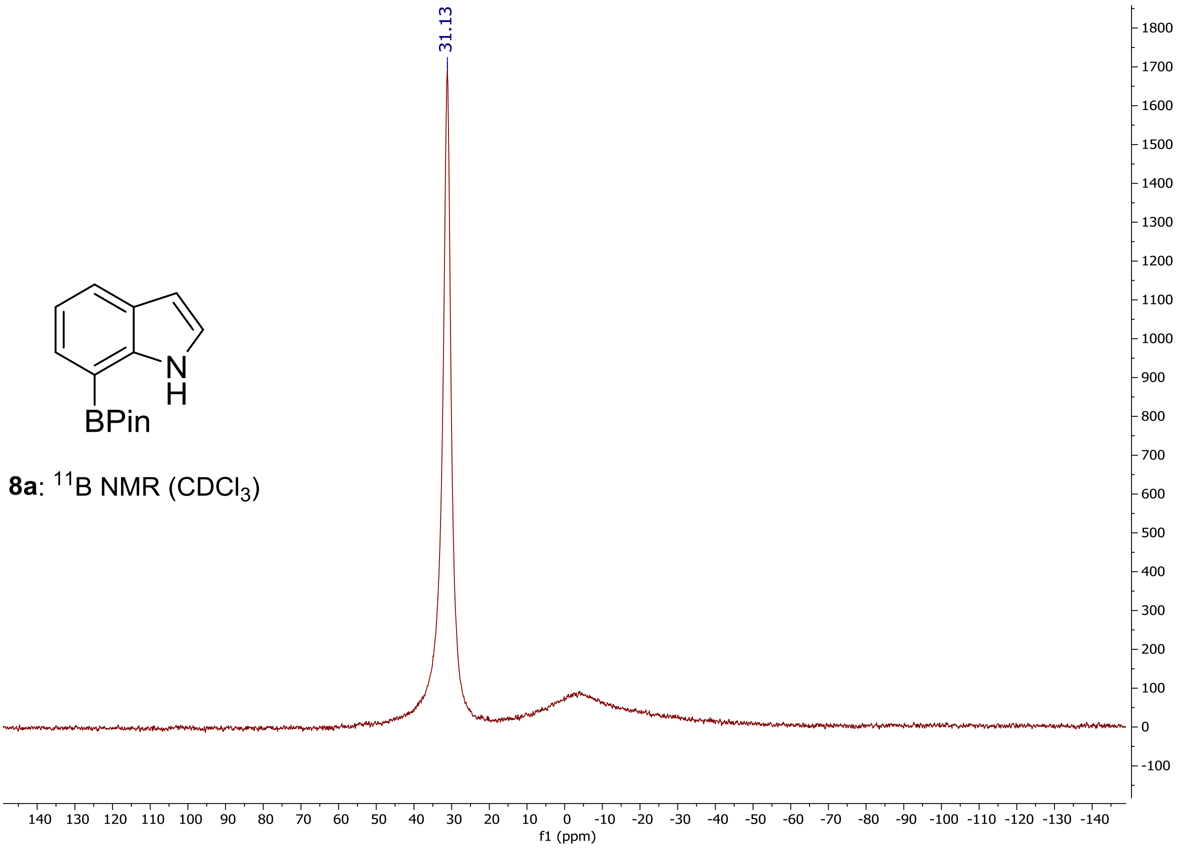

8a: ^{11}B NMR ($CDCl_3$)

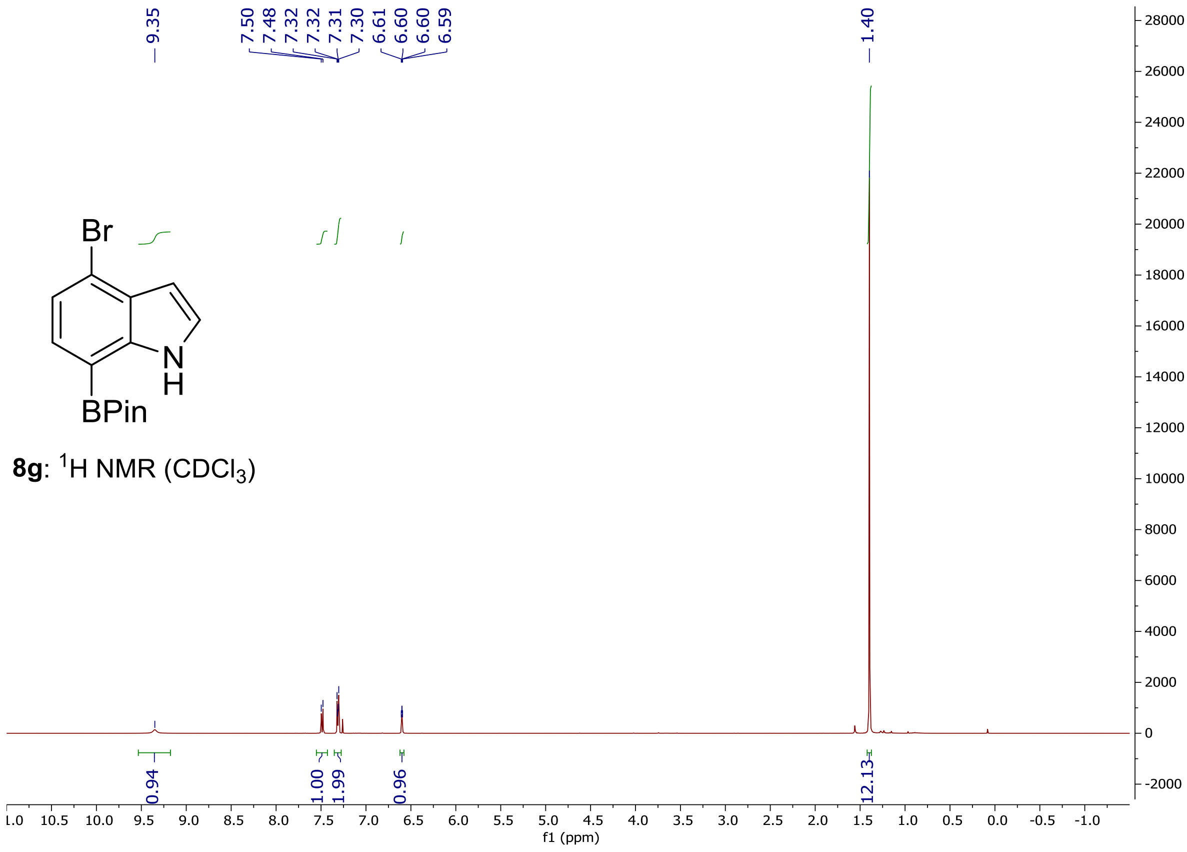
8g: ¹H NMR (CDCl3)
9.35
7.50
7.48
7.32
7.32
7.31
7.30
6.61
6.60
6.60
6.59
1.40
0.94
1.00
1.99
0.96
12.13
f1 (ppm)

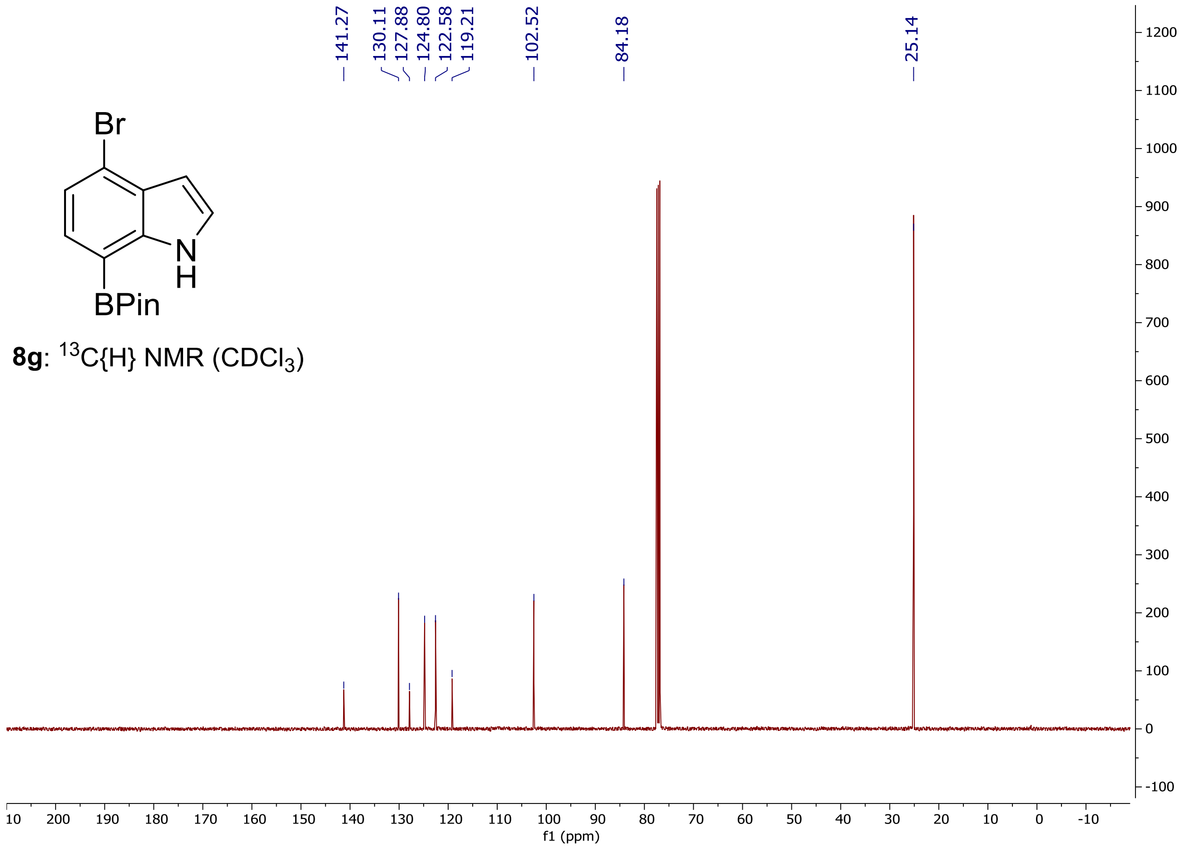
8g: ¹³C{H} NMR (CDCl3)
141.27
130.11
127.88
124.80
122.58
119.21
102.52
84.18
25.14
f1 (ppm)

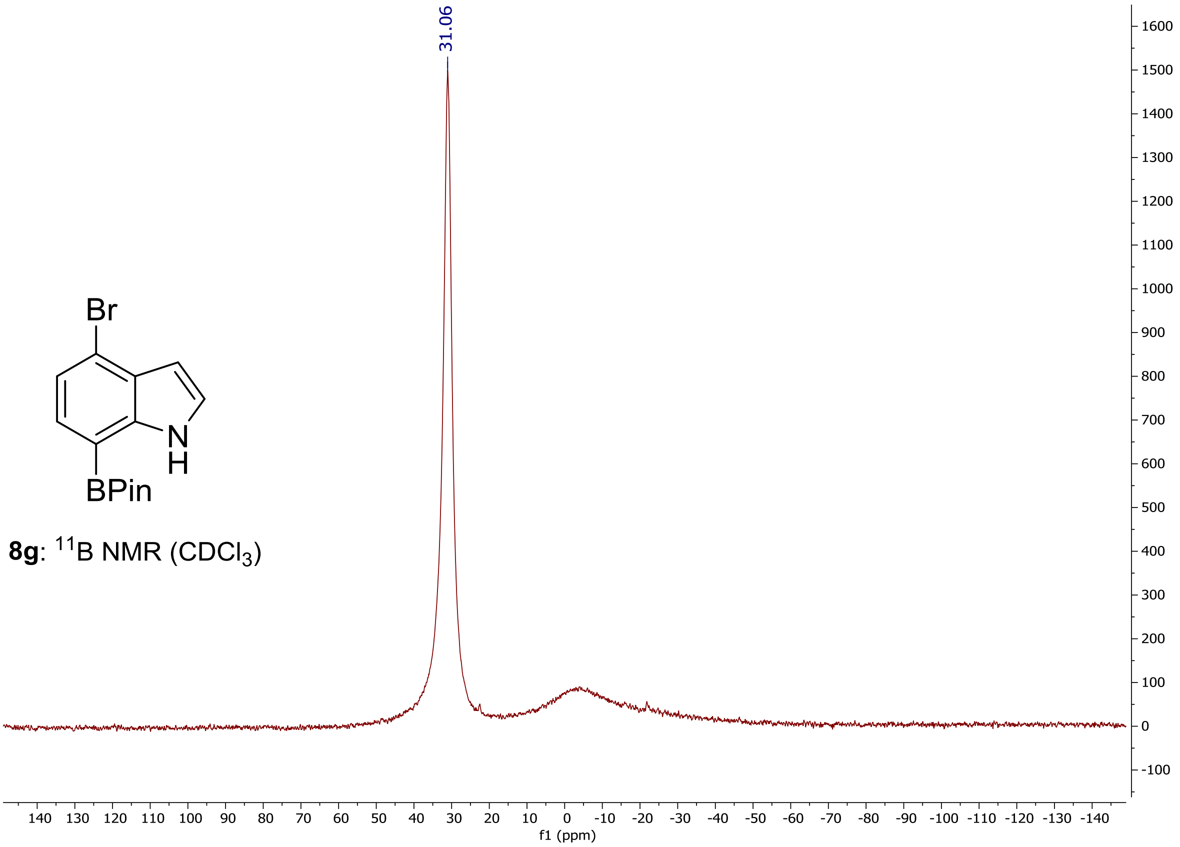

8g: ^{11}B NMR ($CDCl_3$)

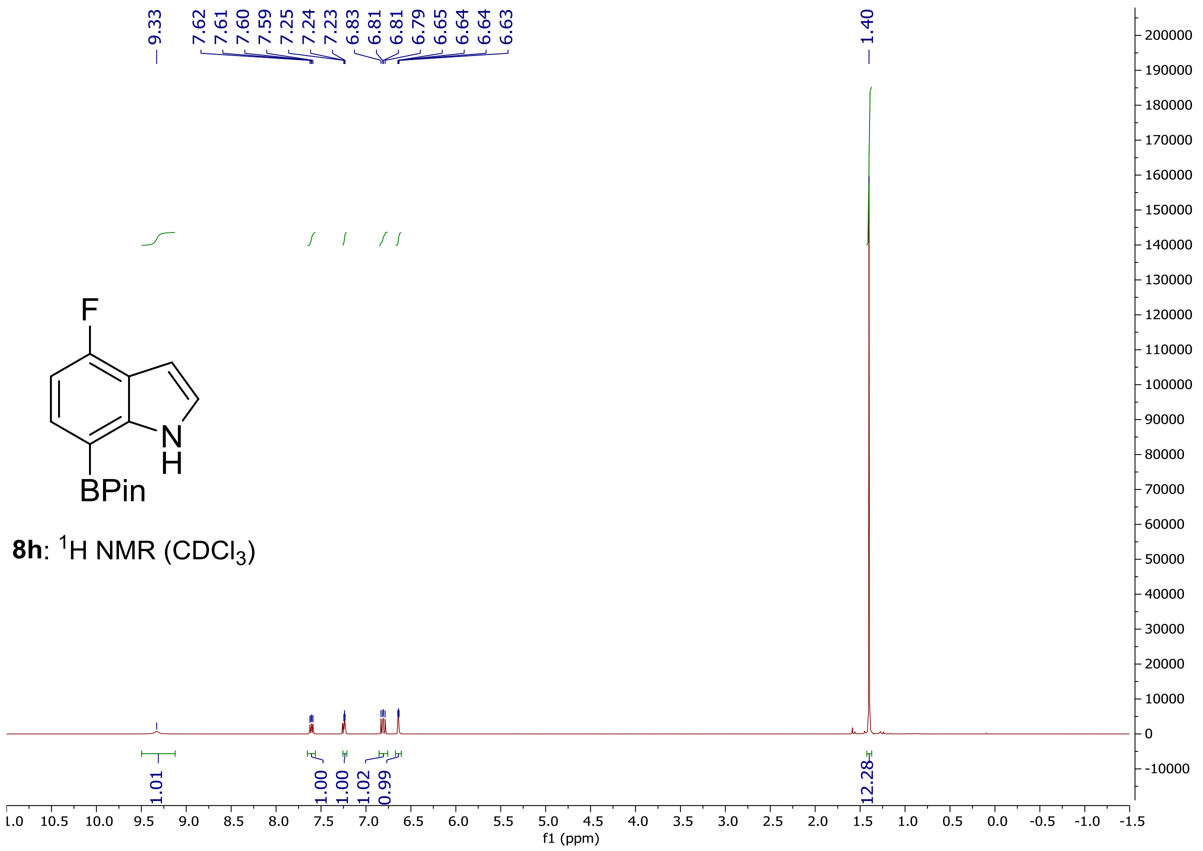

8h: ^{1}H NMR ($CDCl_3$)

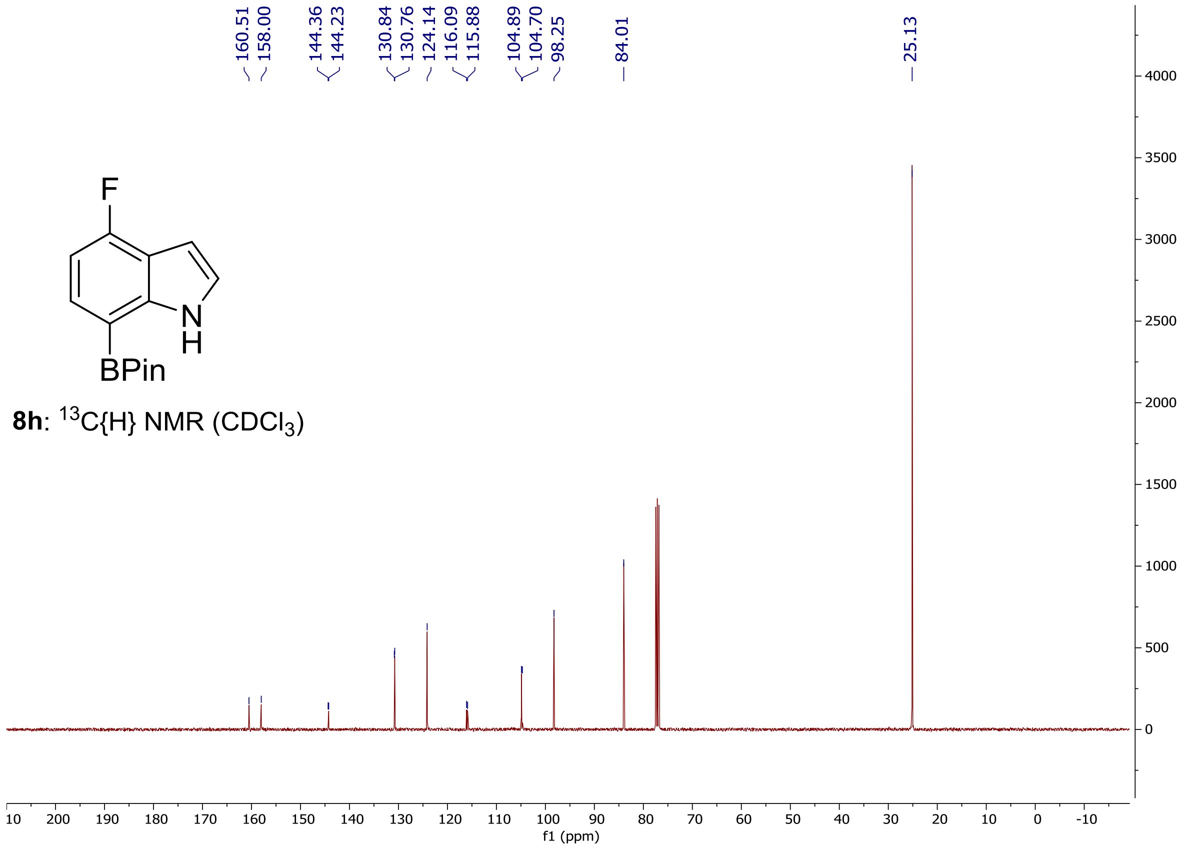

8h: ^{13}C{H} NMR ($CDCl_3$)

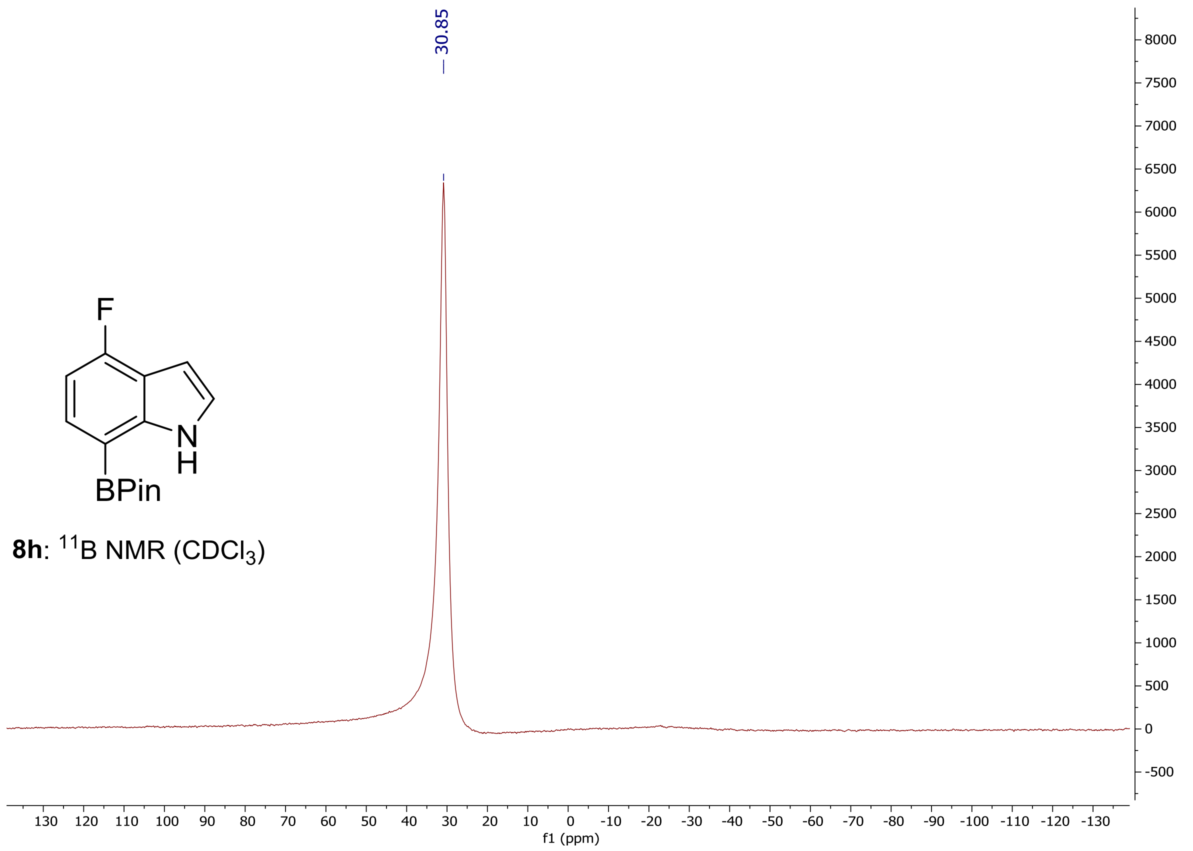
F
N
H
BPin
8h: 11B NMR (CDCl3)
30.85
f1 (ppm)

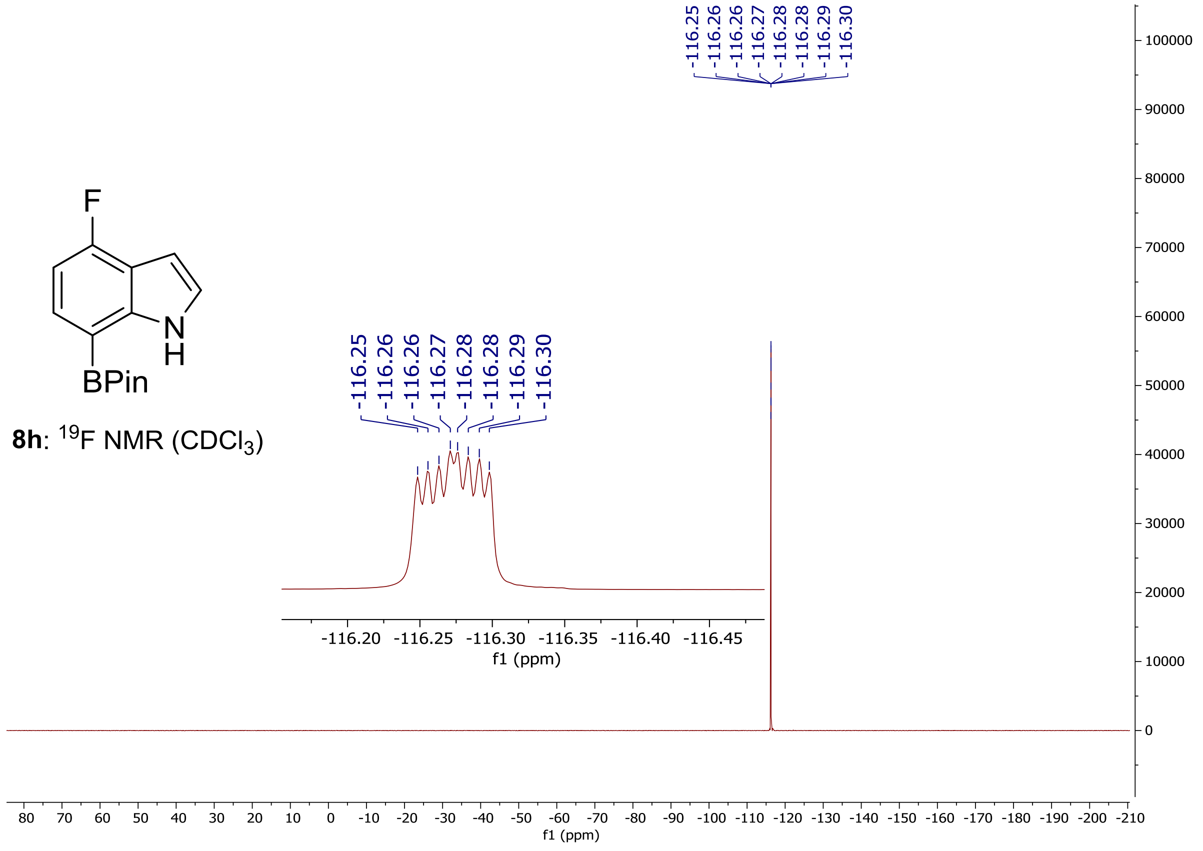
F
N
H
BPin
8h: 19F NMR (CDCl3)
-116.25
-116.26
-116.26
-116.27
-116.28
-116.28
-116.29
-116.30
f1 (ppm)

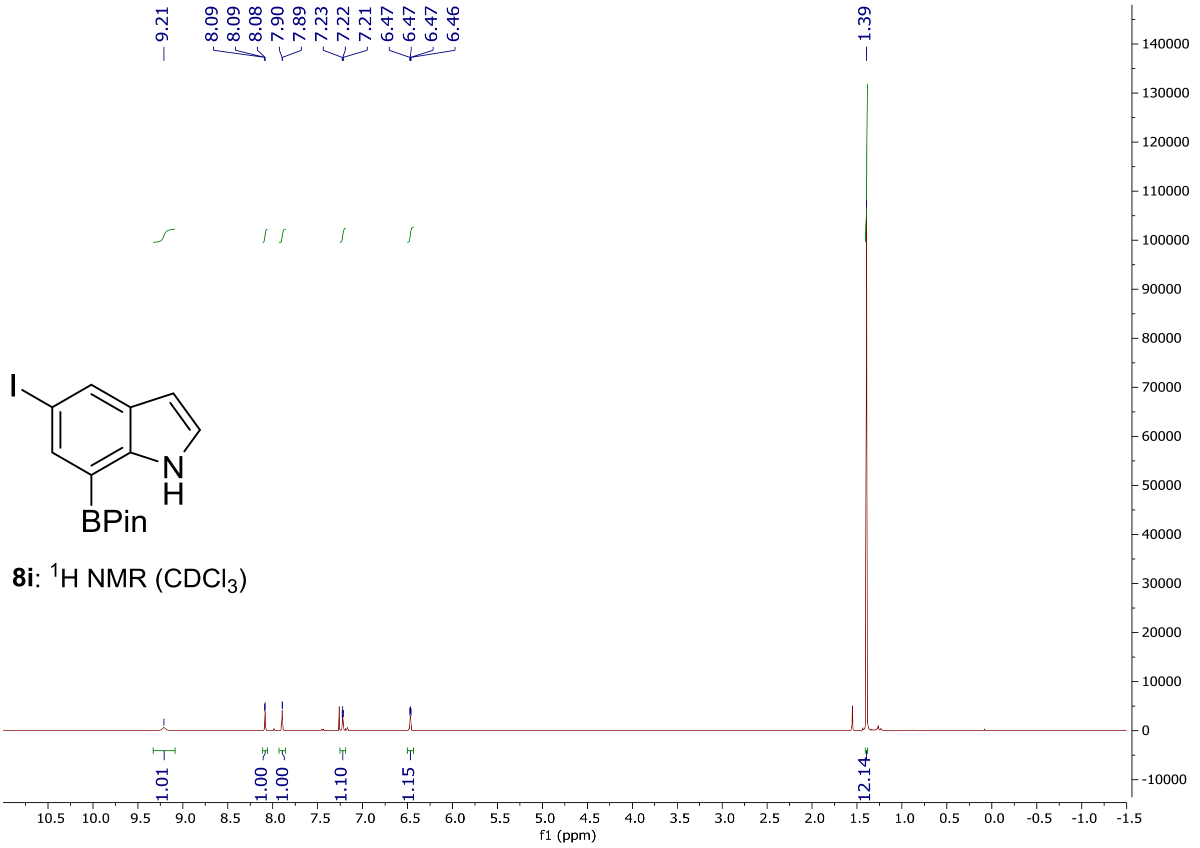
9.21
8.09
8.09
8.08
7.90
7.89
7.23
7.22
7.21
6.47
6.47
6.47
6.46
1.39
I
N
H
BPin
8i: ^{1}H NMR ($CDCl_3$)
1.01
1.00
1.00
1.10
1.15
12.14
f1 (ppm)

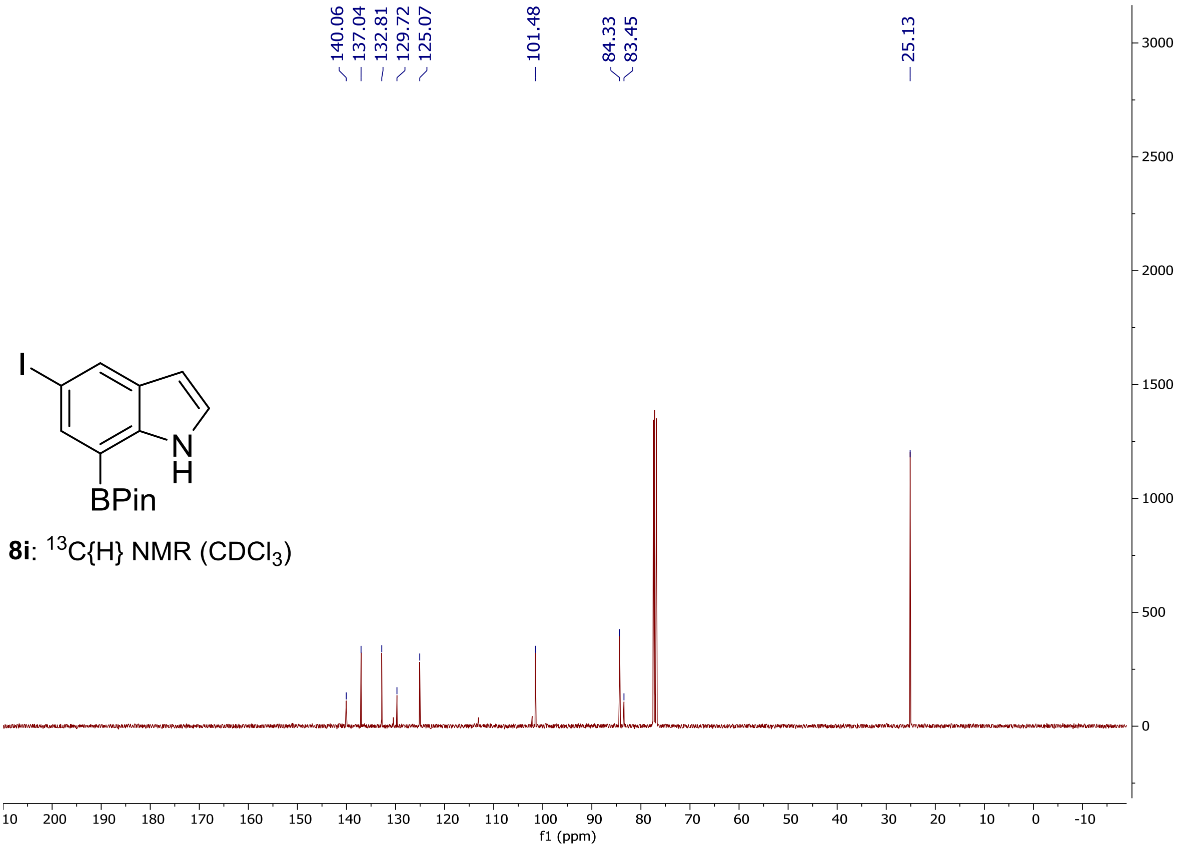
140.06
137.04
132.81
129.72
125.07
101.48
84.33
83.45
25.13
I
N
H
BPin
8i: ^{13}C{H} NMR ($CDCl_3$)
f1 (ppm)

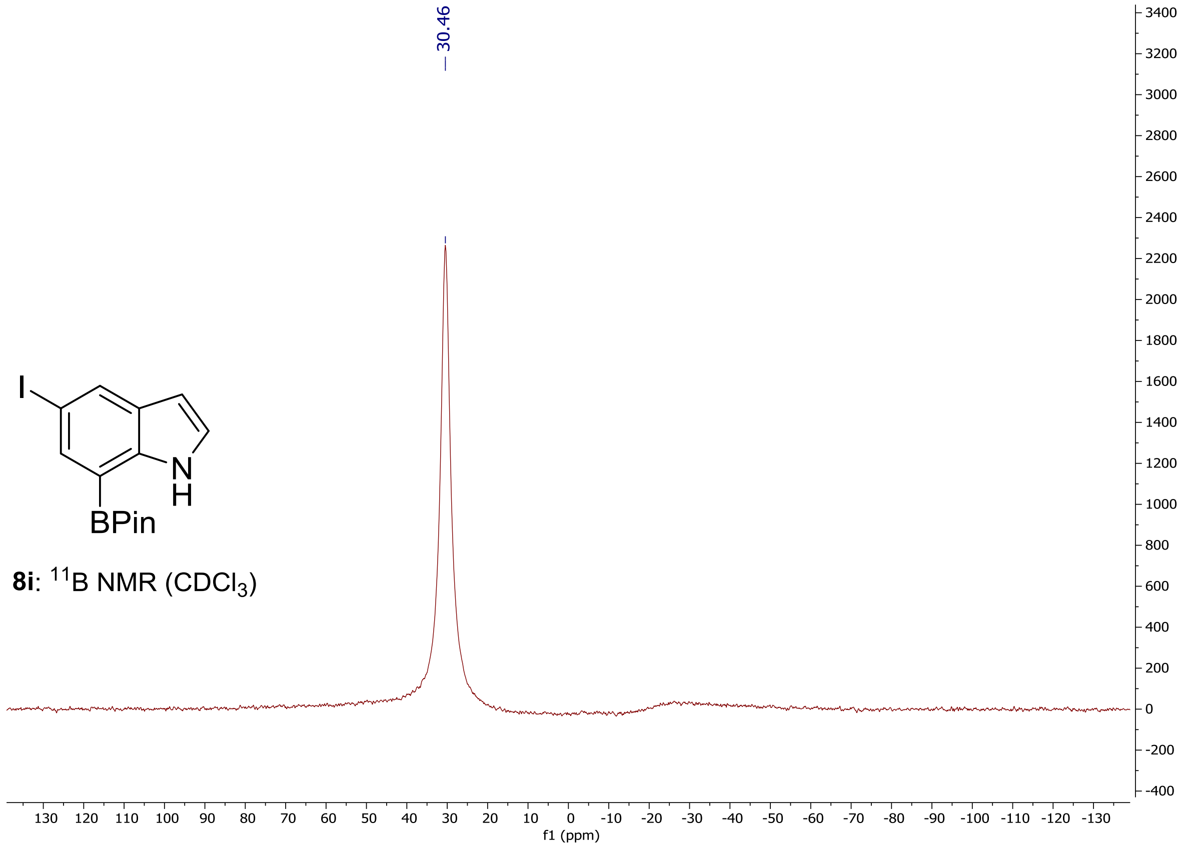

8i: ^{11}B NMR ($CDCl_3$)

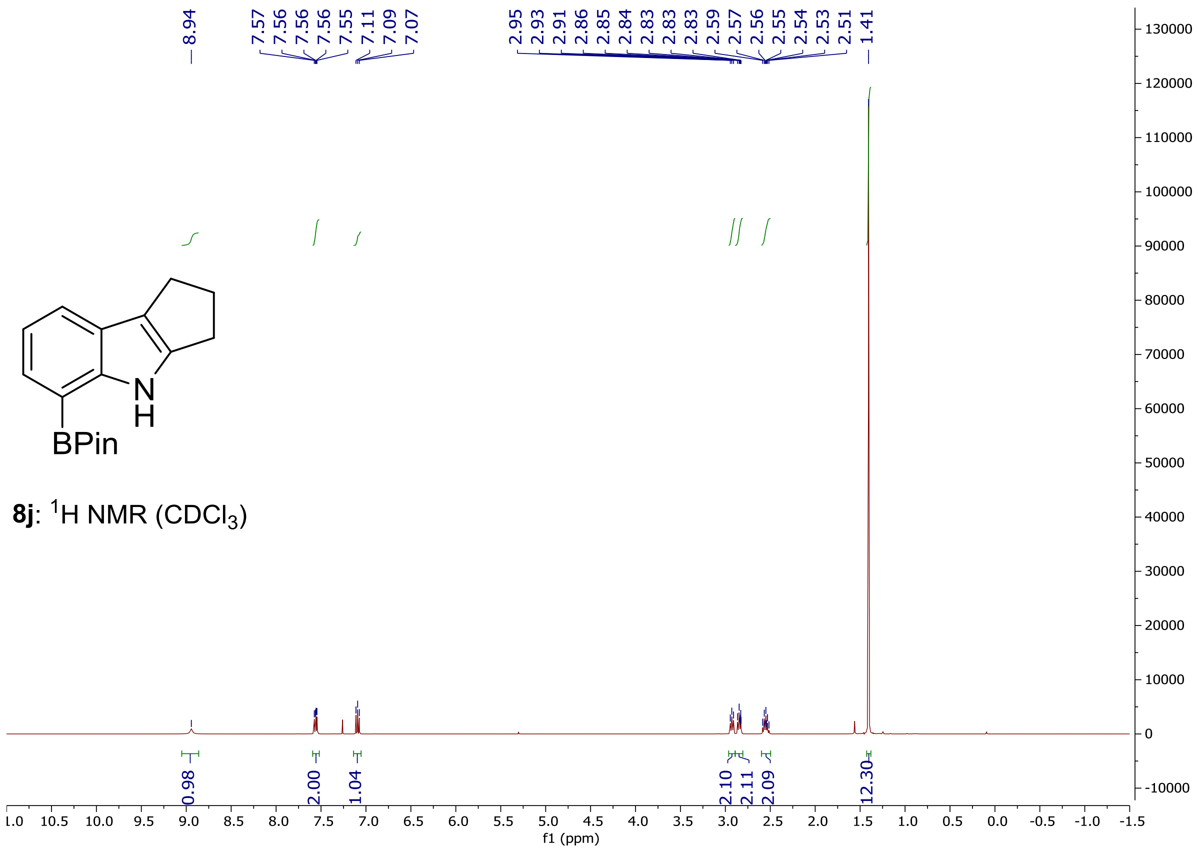
8.94
7.57
7.56
7.56
7.56
7.55
7.11
7.09
7.07
2.95
2.93
2.91
2.86
2.85
2.84
2.83
2.83
2.83
2.59
2.57
2.56
2.55
2.54
2.53
2.51
1.41
BPin
N
H
8j: 1H NMR (CDCl3)
0.98
2.00
1.04
2.10
2.11
2.09
12.30
f1 (ppm)

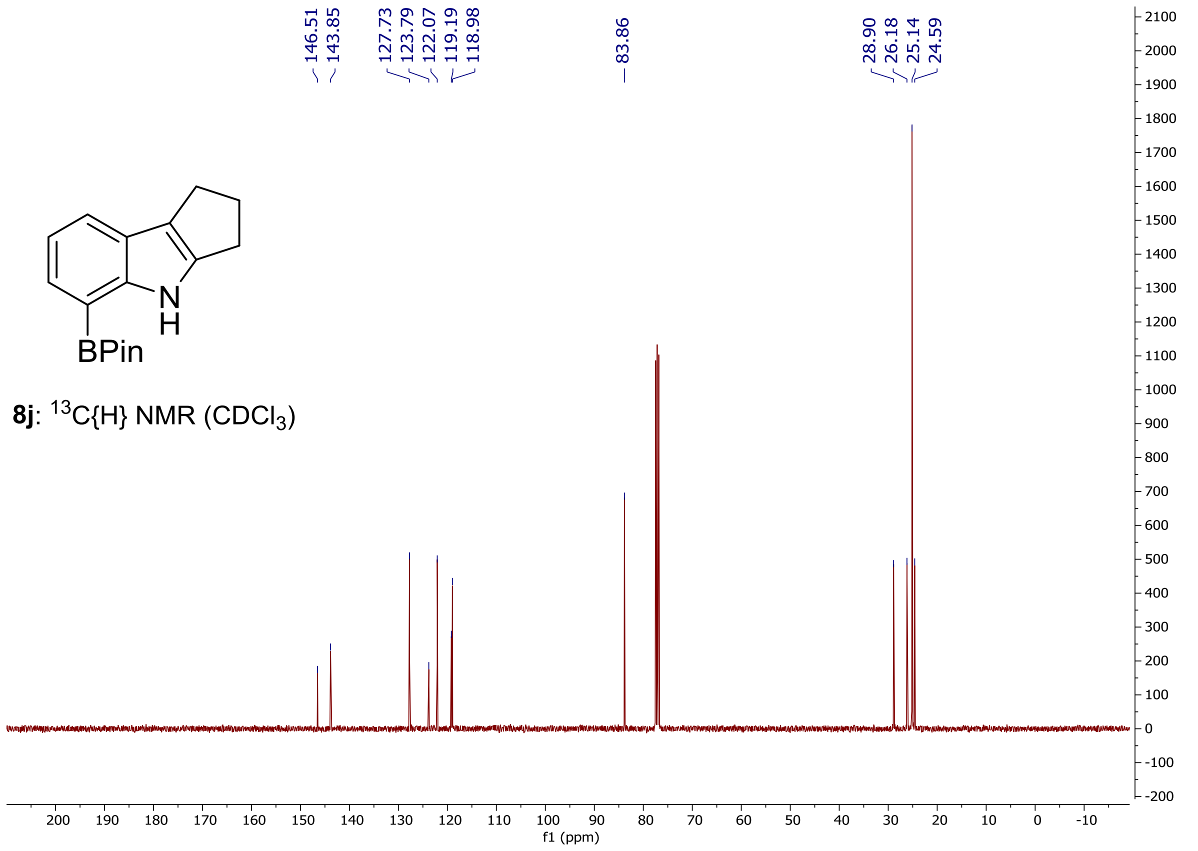
146.51
143.85
127.73
123.79
122.07
119.19
118.98
83.86
28.90
26.18
25.14
24.59
BPin
N
H
8j: 13C{H} NMR (CDCl3)
f1 (ppm)

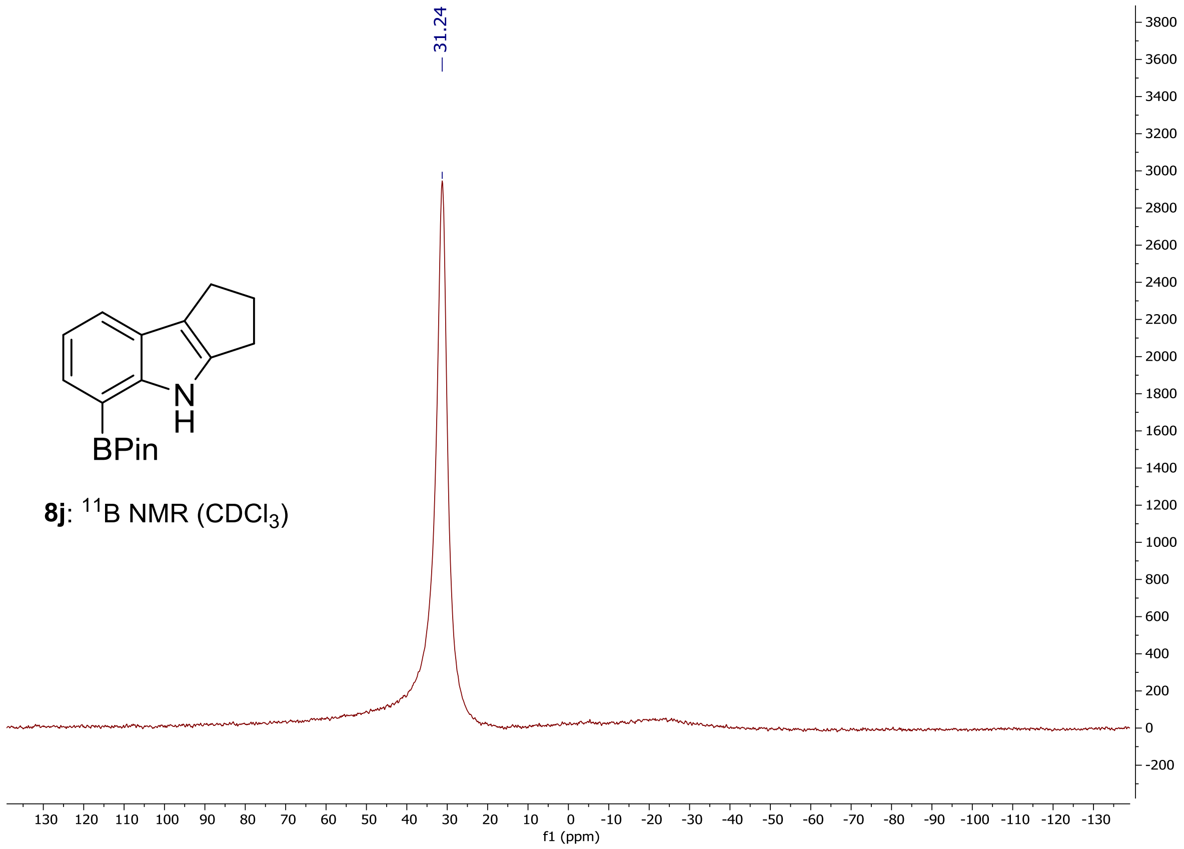

31.24
N
H
BPin
8j: 11B NMR (CDCl3)
f1 (ppm)

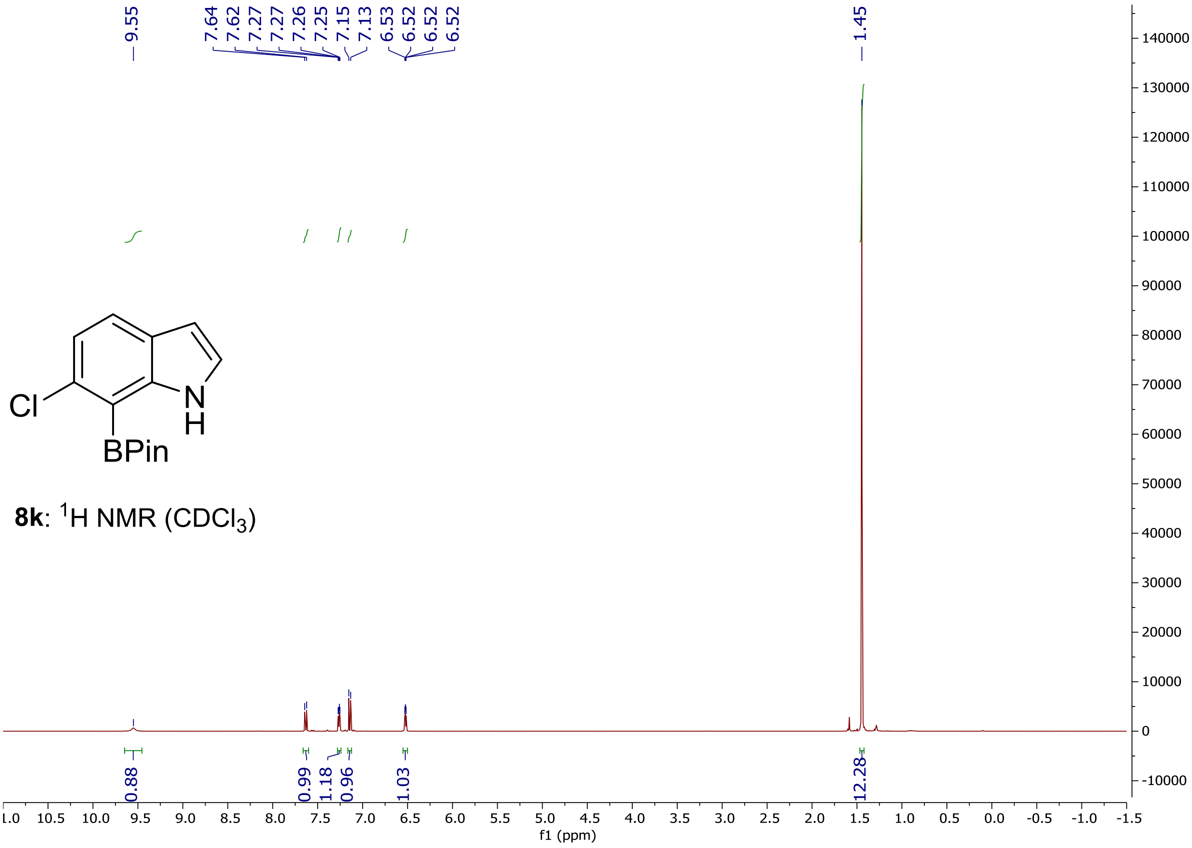
9.55
7.64
7.62
7.27
7.27
7.26
7.25
7.15
7.13
6.53
6.52
6.52
6.52
1.45
Cl
N
H
BPin
8k: 1H NMR (CDCl3)
0.88
0.99
1.18
0.96
1.03
12.28
f1 (ppm)

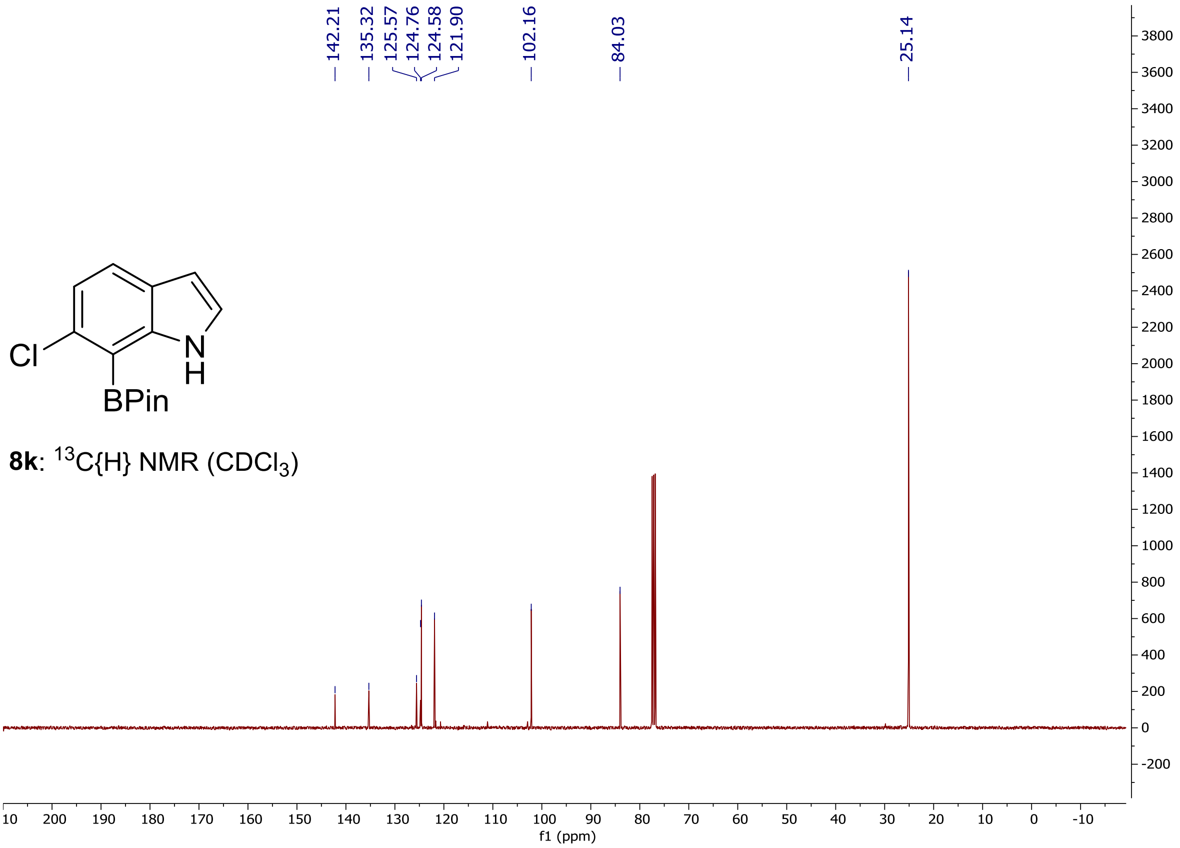
142.21
135.32
125.57
124.76
124.58
121.90
102.16
84.03
25.14
Cl
N
H
BPin
8k: 13C{H} NMR (CDCl3)
f1 (ppm)

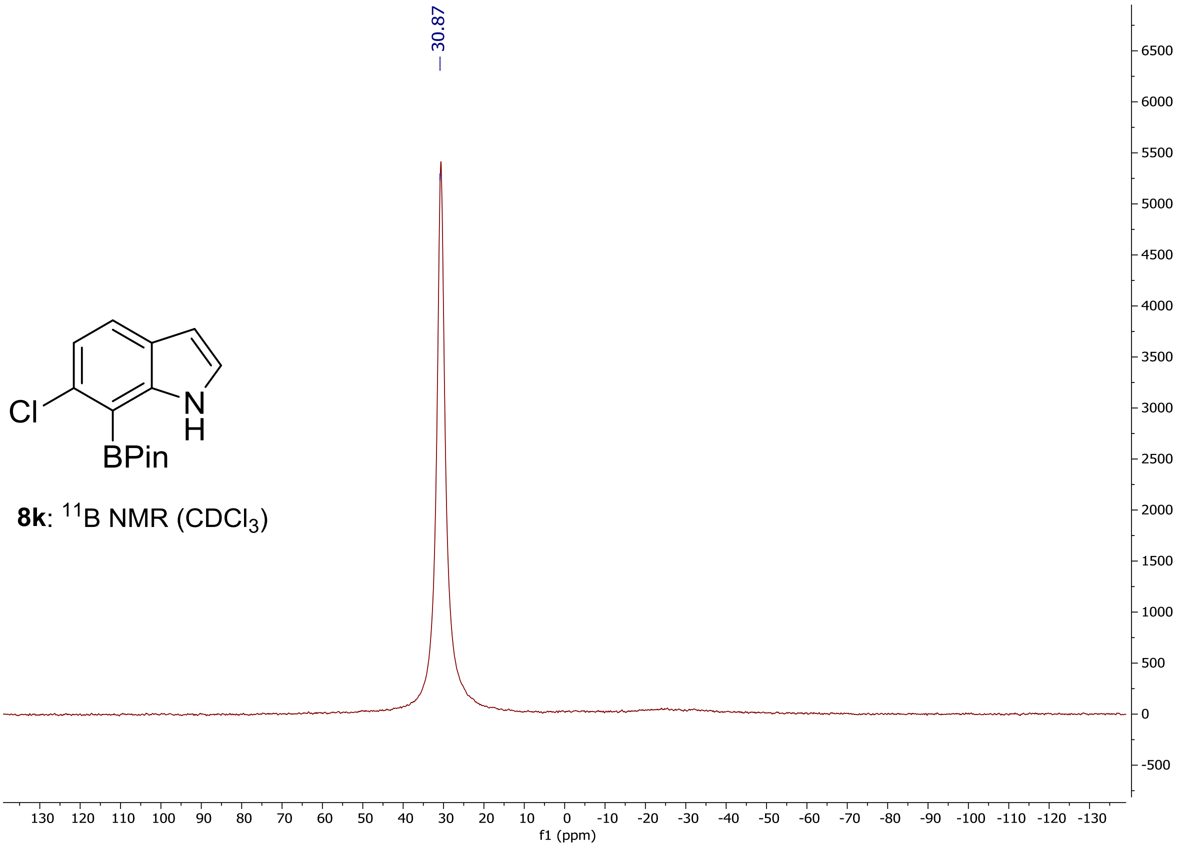

8k: ^{11}B NMR ($CDCl_3$)

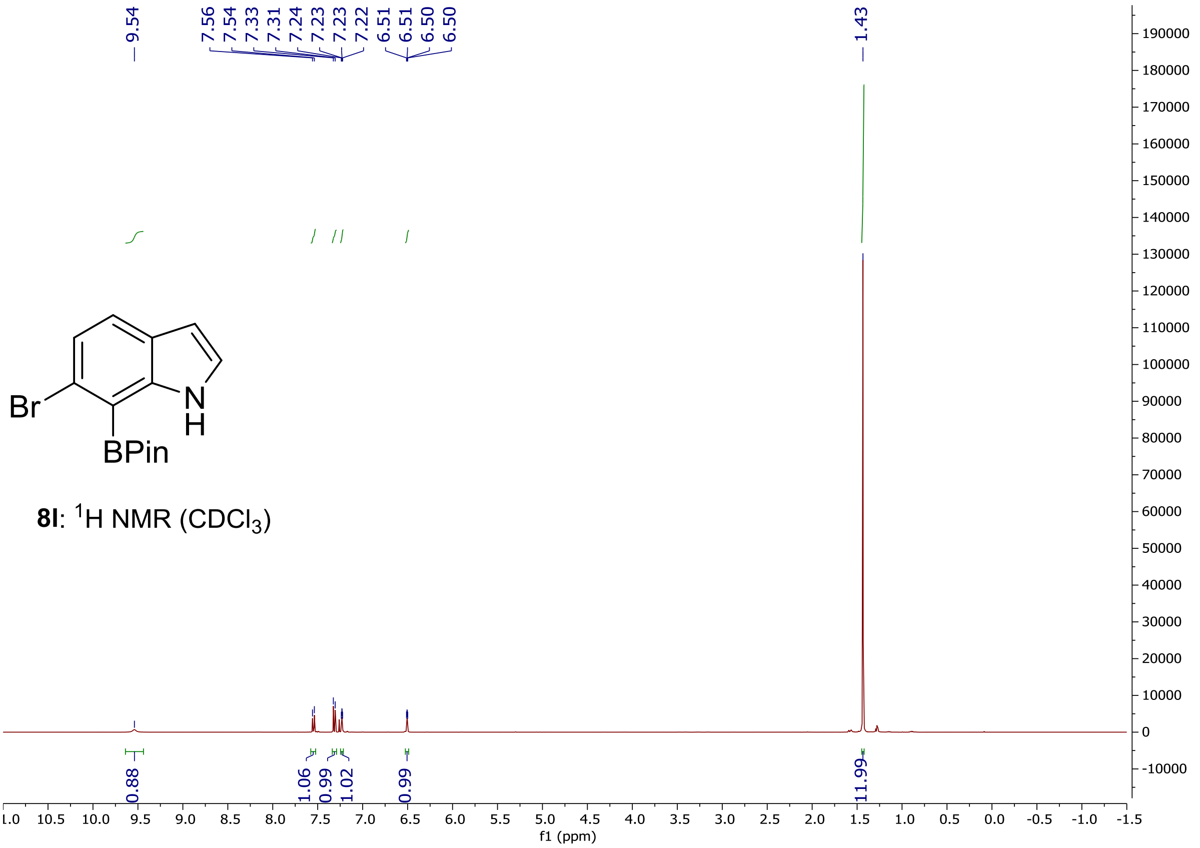

9.54
7.56
7.54
7.33
7.31
7.24
7.23
7.23
7.22
6.51
6.51
6.50
6.50
1.43
Br
N
H
BPin
8l: 1H NMR (CDCl3)
0.88
1.06
0.99
1.02
0.99
11.99
f1 (ppm)

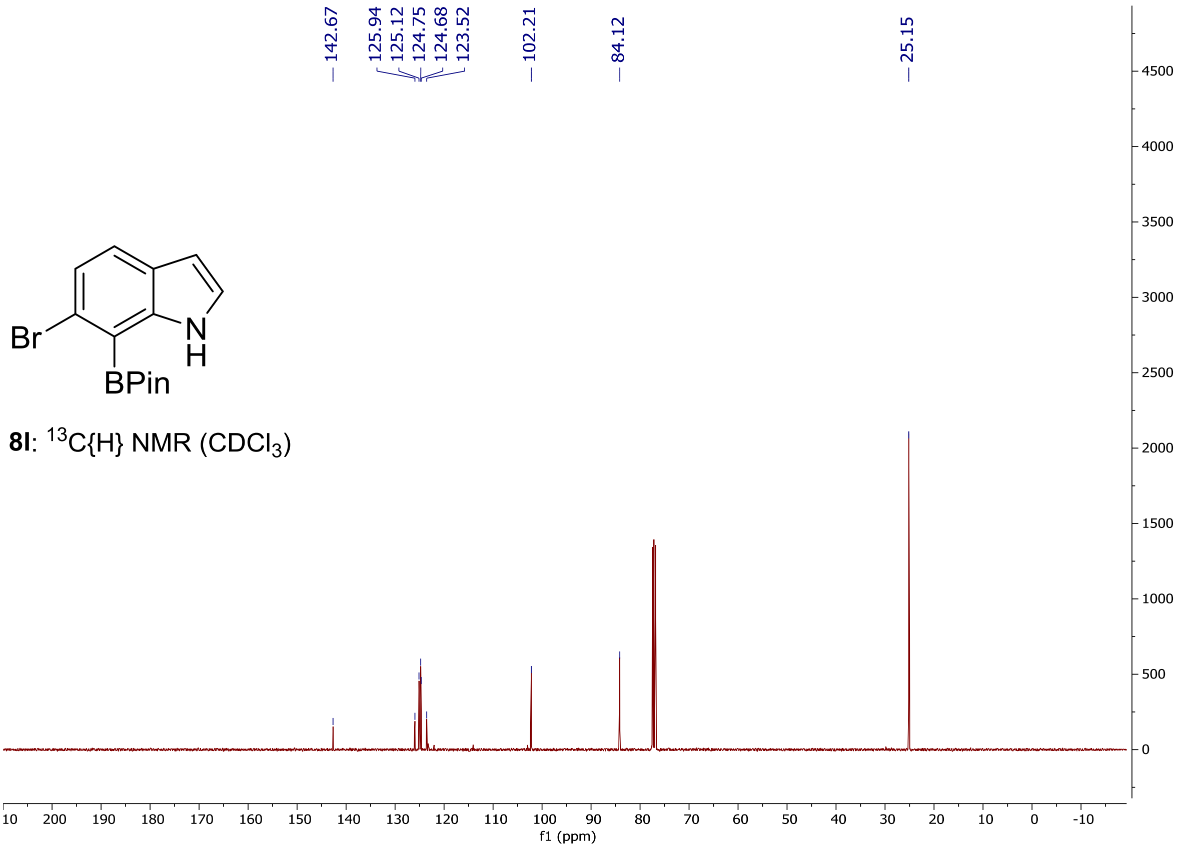

142.67
125.94
125.12
124.75
124.68
123.52
102.21
84.12
25.15
Br
N
H
BPin
8l: 13C{H} NMR (CDCl3)
f1 (ppm)

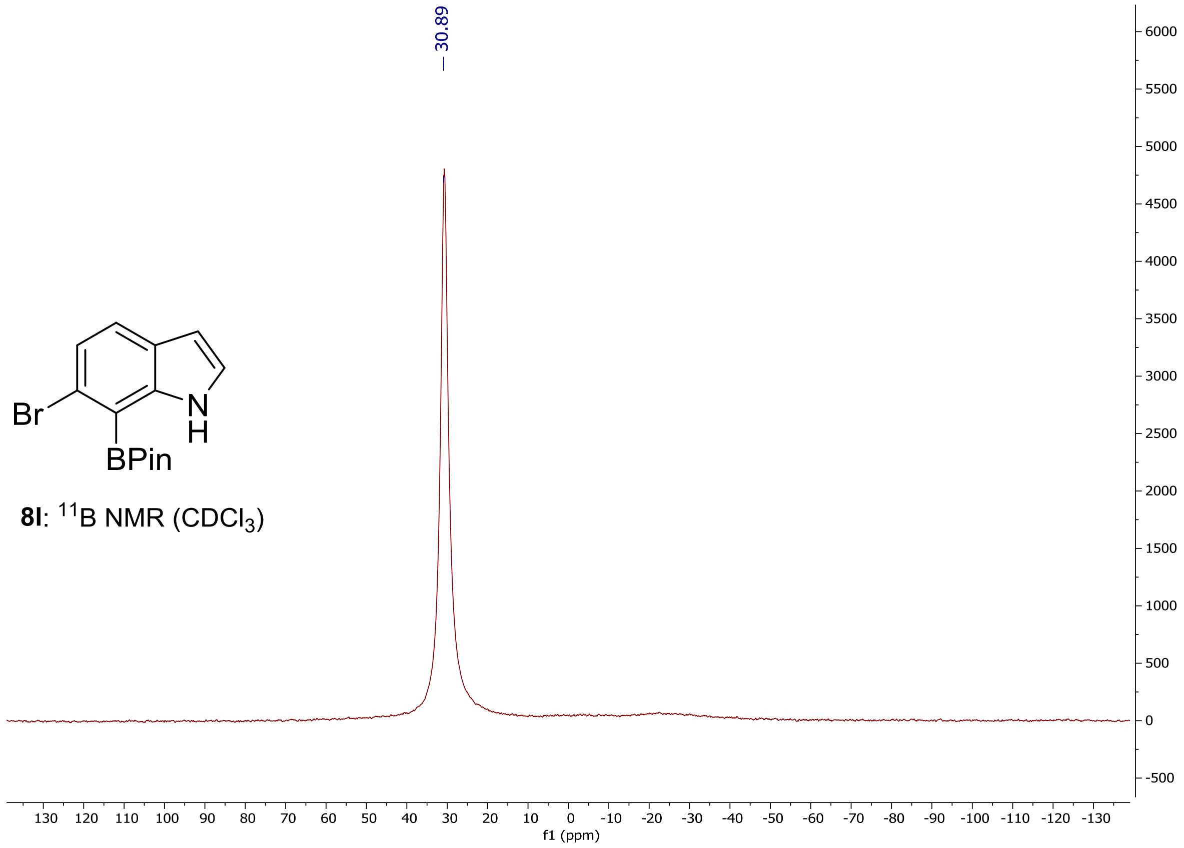
Br
N
H
BPin
8l: ^{11}B NMR ($CDCl_3$)
30.89
f1 (ppm)

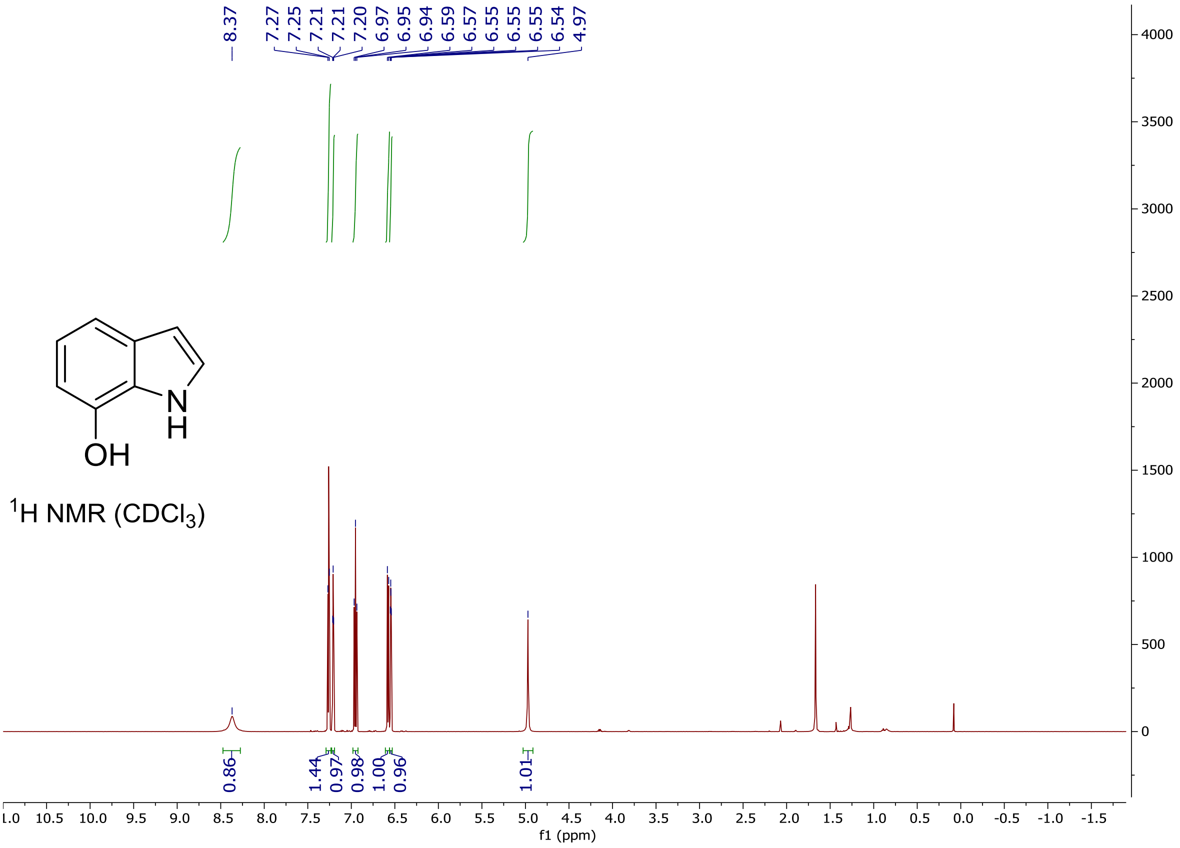

1H NMR (CDCl3)
f1 (ppm)

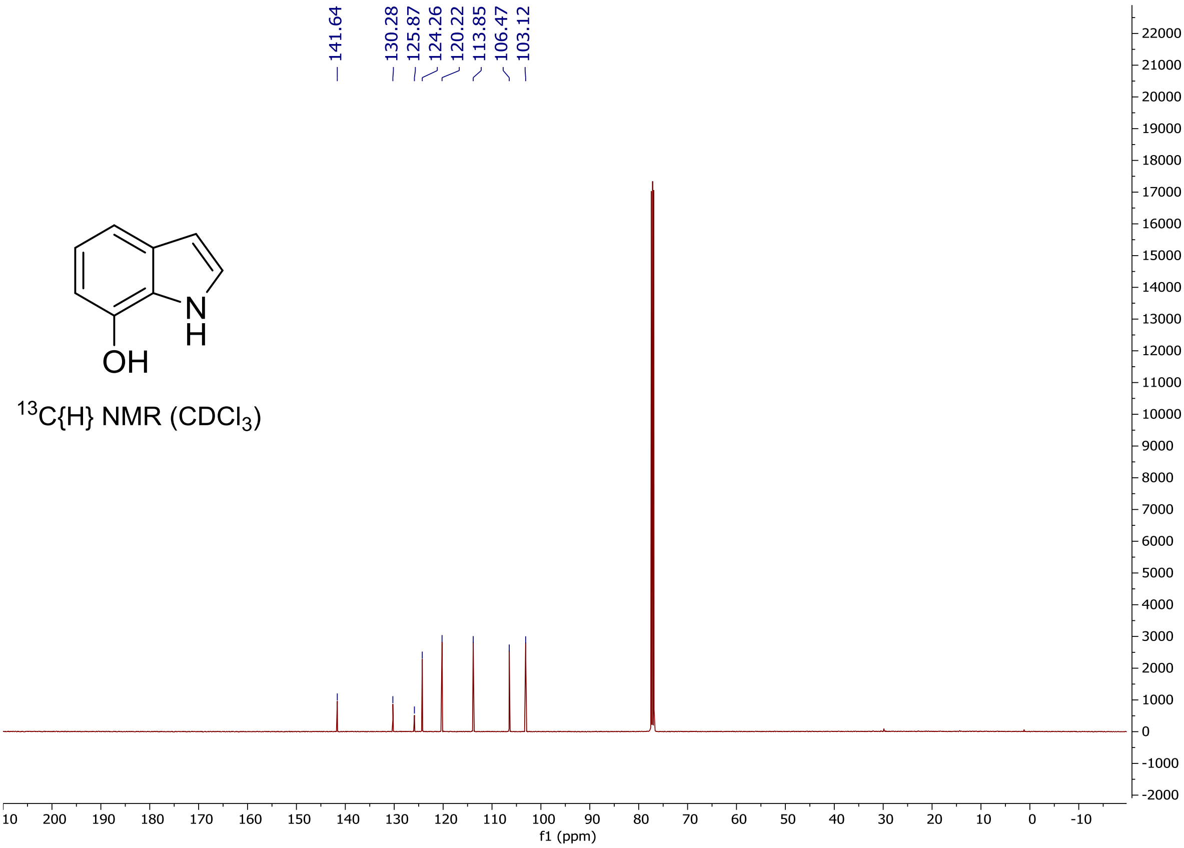

13C{H} NMR (CDCl3)
f1 (ppm)

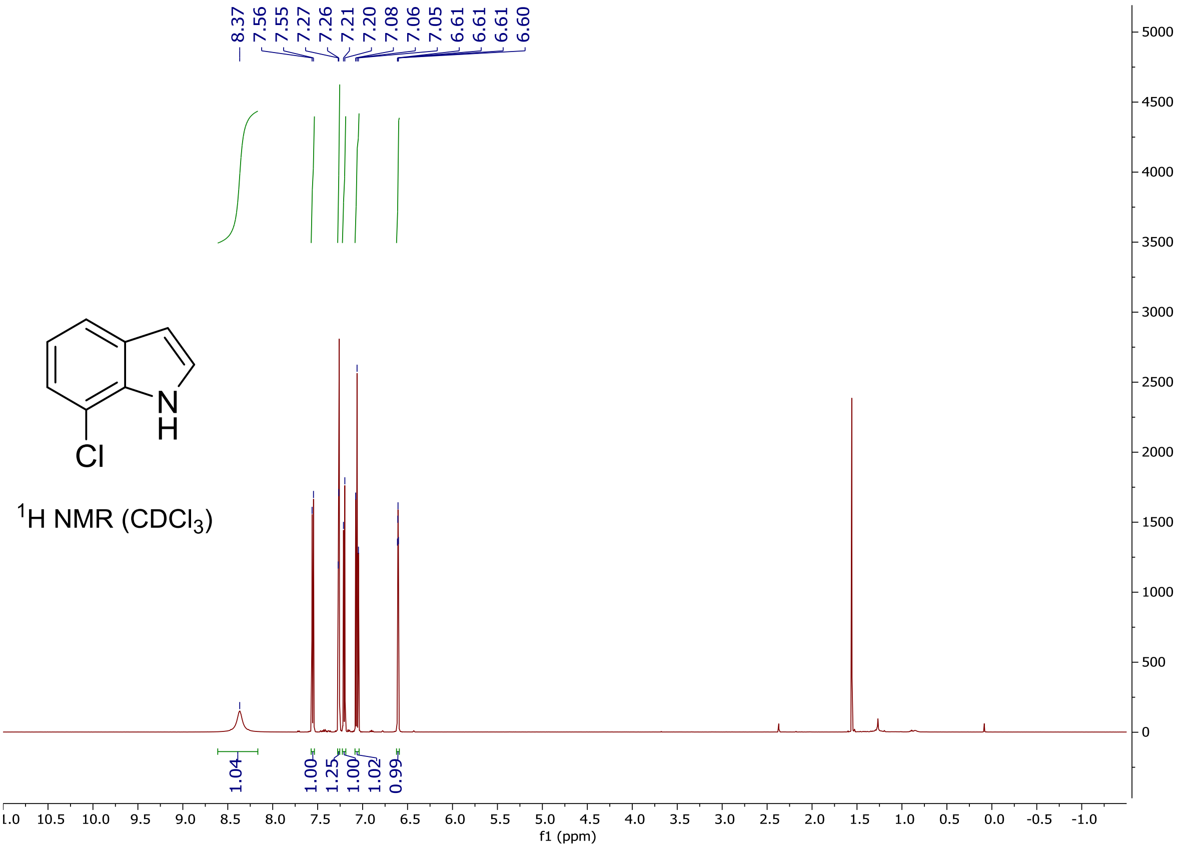
8.37
7.56
7.55
7.27
7.26
7.21
7.20
7.08
7.06
7.05
6.61
6.61
6.61
6.60
Cl
N
H
1H NMR (CDCl3)
1.04
1.00
1.25
1.00
1.02
0.99
f1 (ppm)

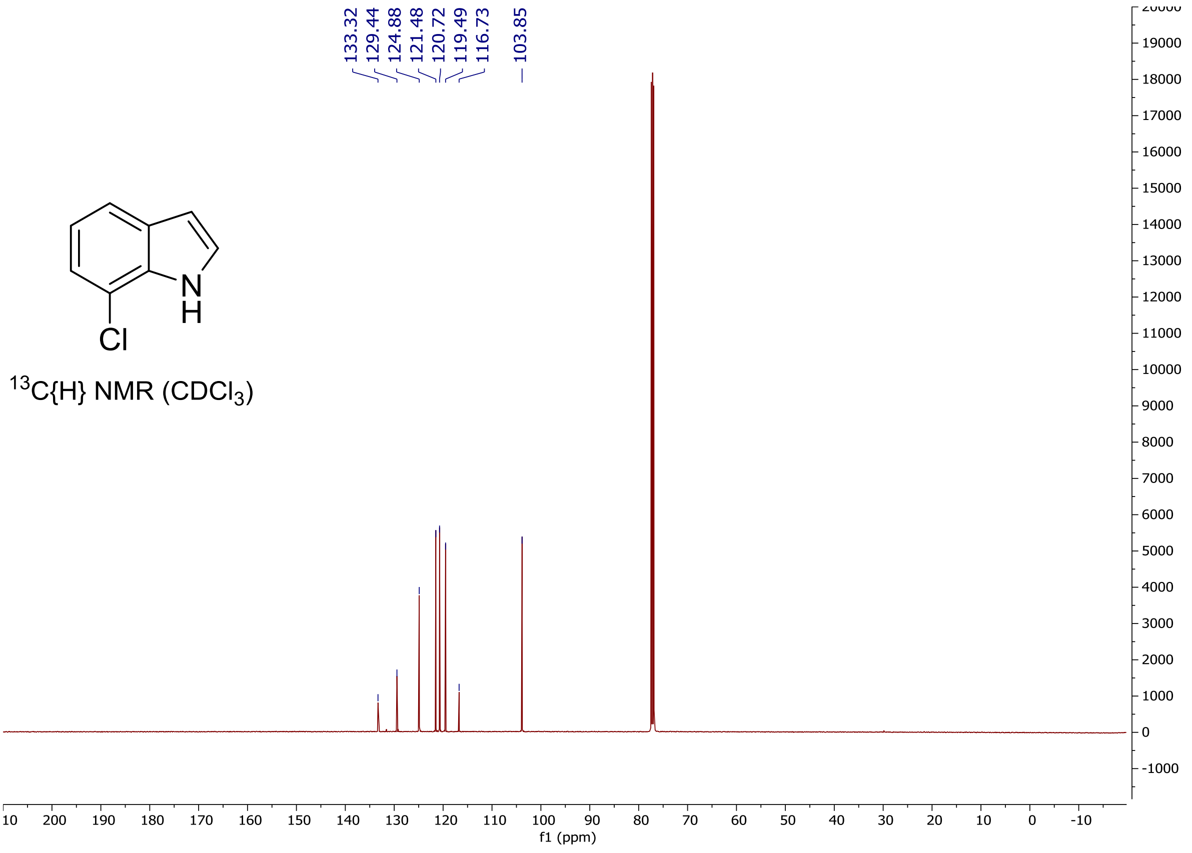
133.32
129.44
124.88
121.48
120.72
119.49
116.73
103.85
N
H
Cl
13C{H} NMR (CDCl3)
f1 (ppm)

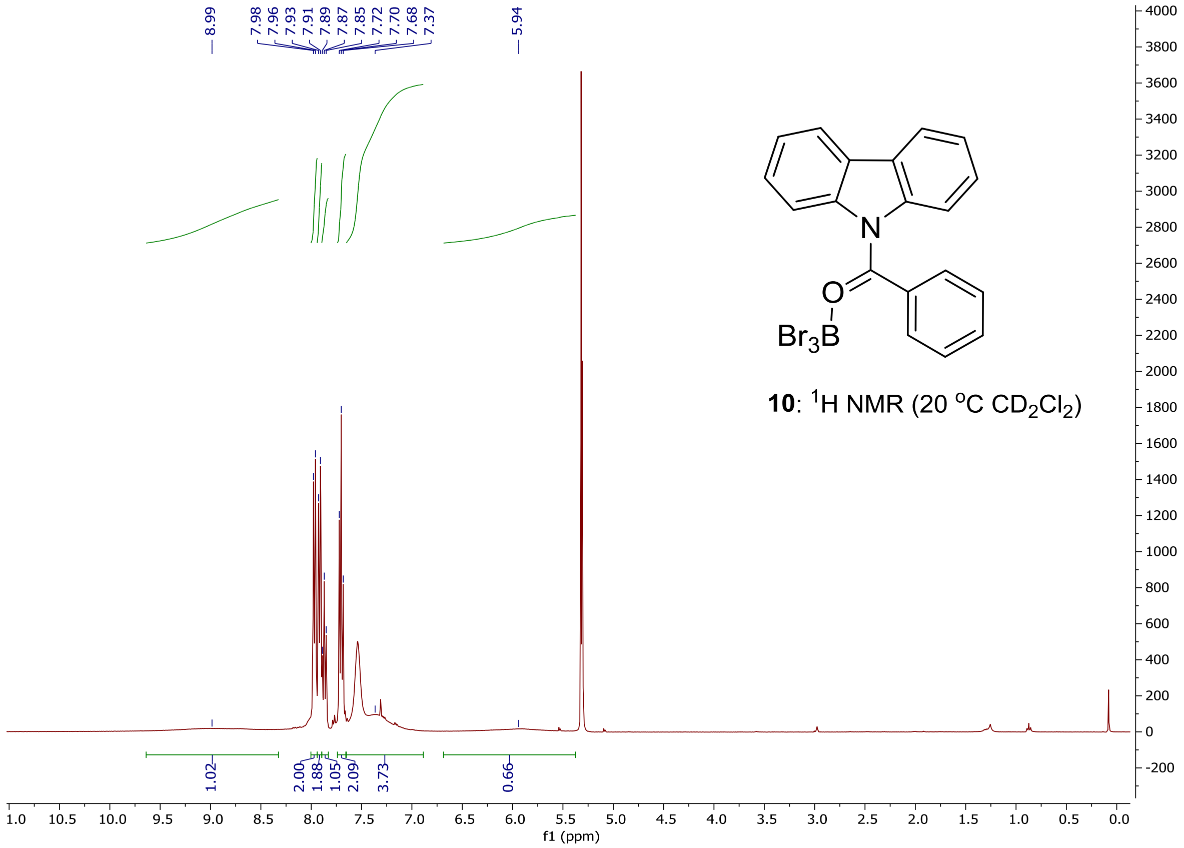
8.99
7.98 7.96 7.93 7.91 7.89 7.87 7.85 7.72 7.70 7.68 7.37
5.94
Br$_3$B
O
N
10: ^{1}H NMR (20 ^{o}C CD_2Cl_2)
1.02 2.00 1.88 1.05 2.09 3.73 0.66
f1 (ppm)

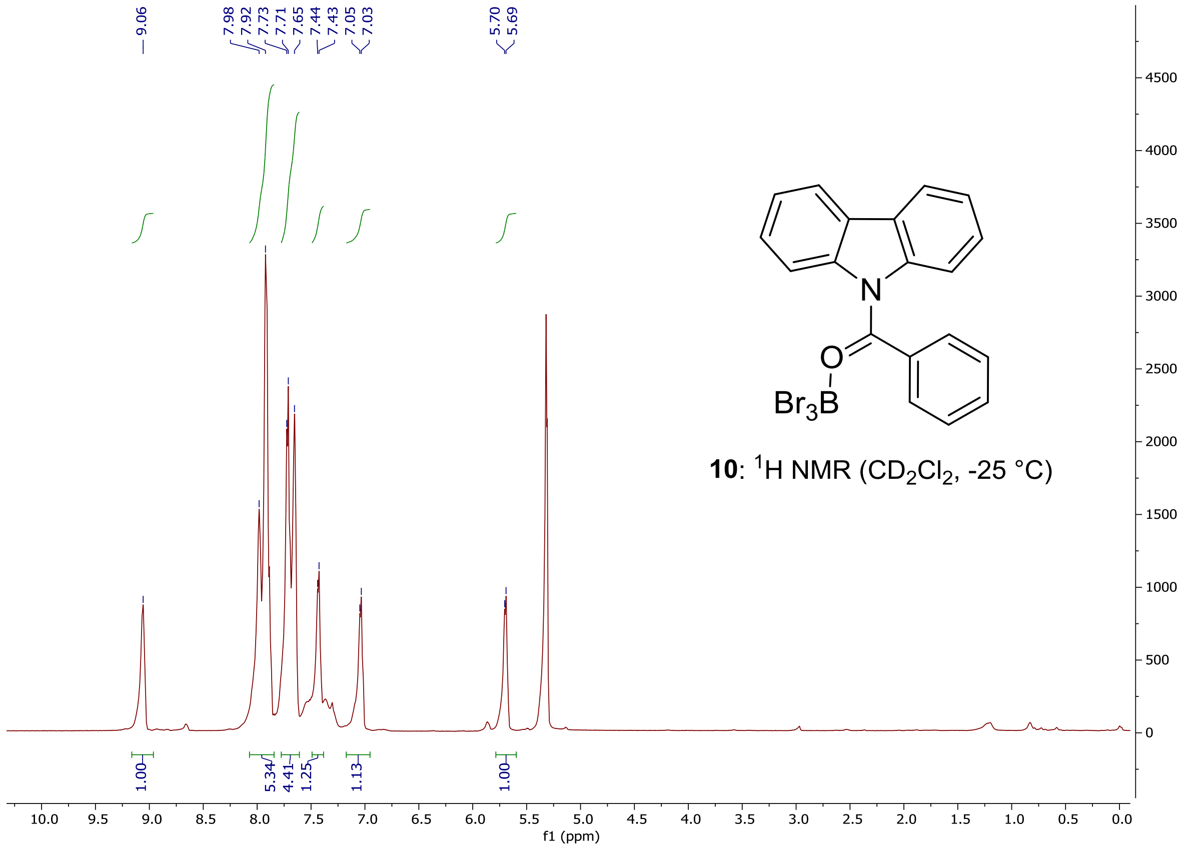
9.06
7.98 7.92 7.73 7.71 7.65 7.44 7.43 7.05 7.03
5.70 5.69
Br$_3$B
O
N
10: ^{1}H NMR (CD_2Cl_2, -25 °C)
1.00 5.34 4.41 1.25 1.13 1.00
f1 (ppm)

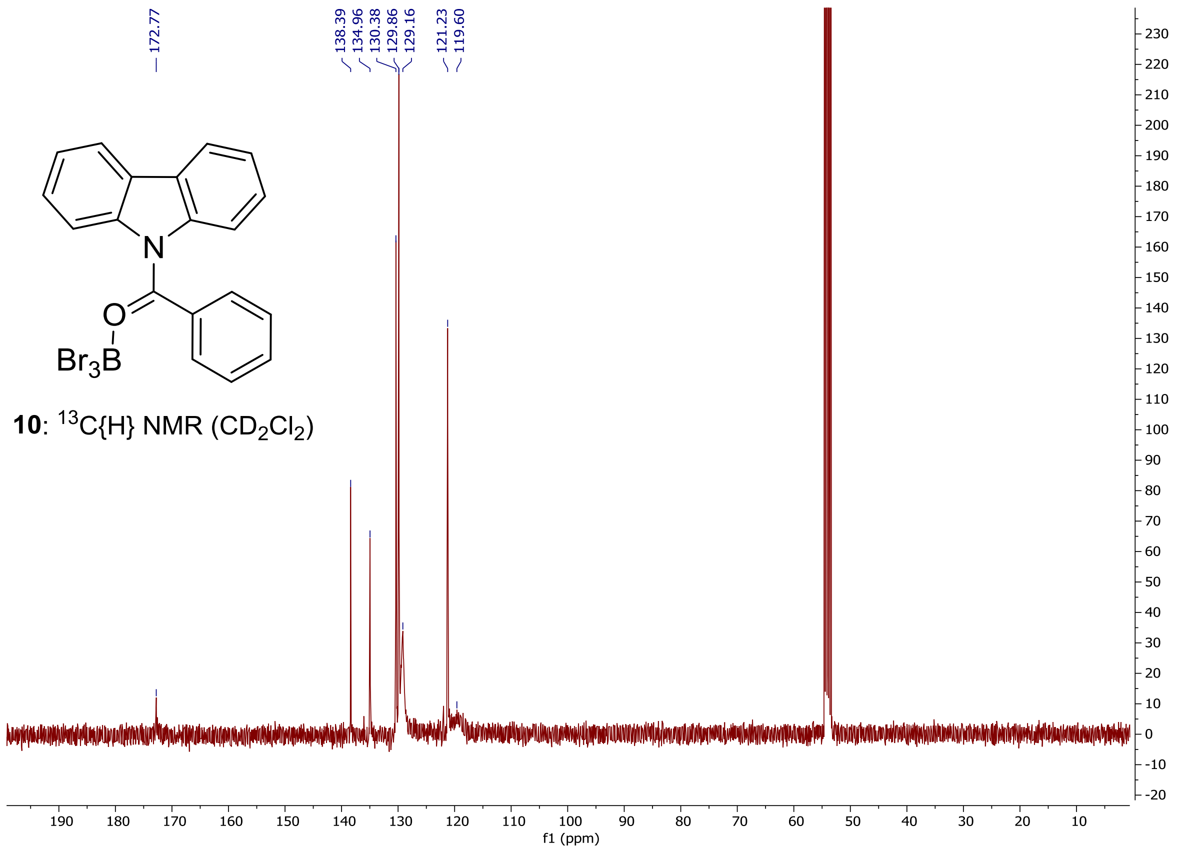

10: $^{13}C\{H\}$ NMR (CD_2Cl_2)

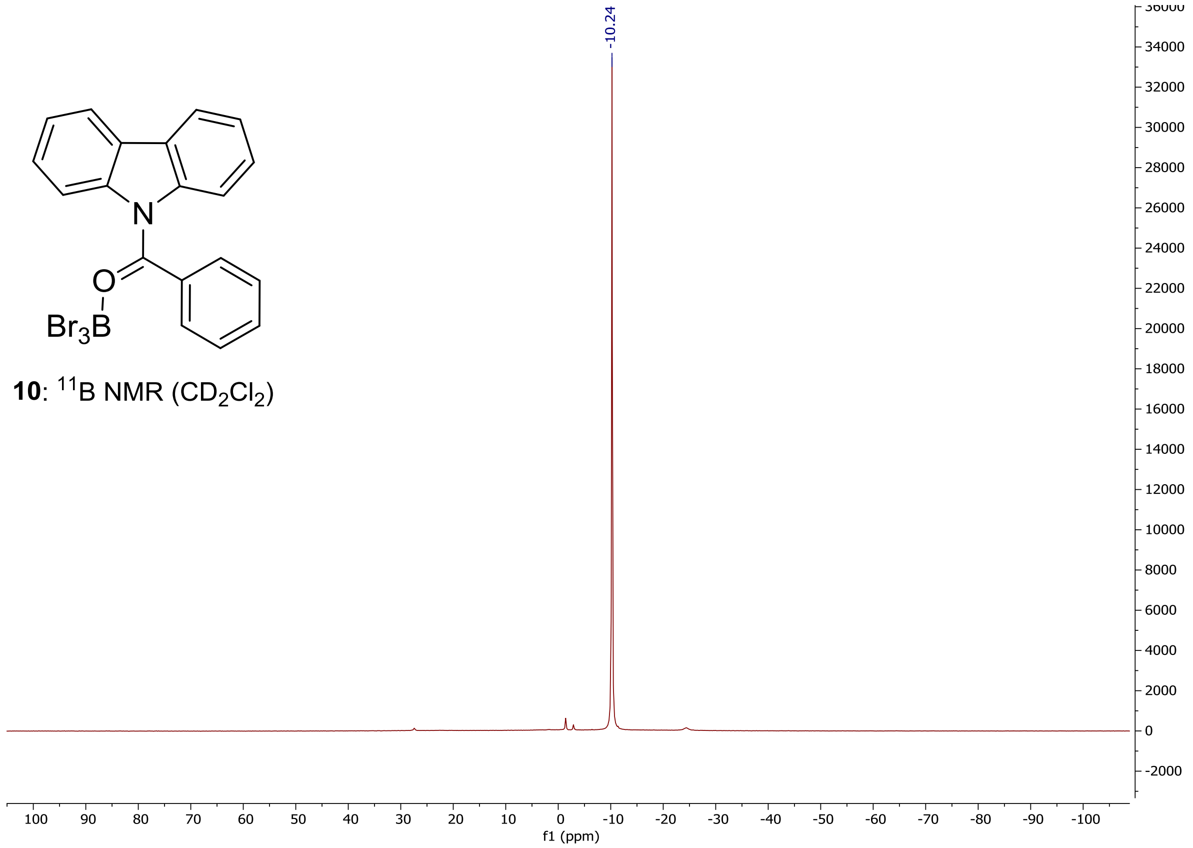

10: ^{11}B NMR (CD_2Cl_2)

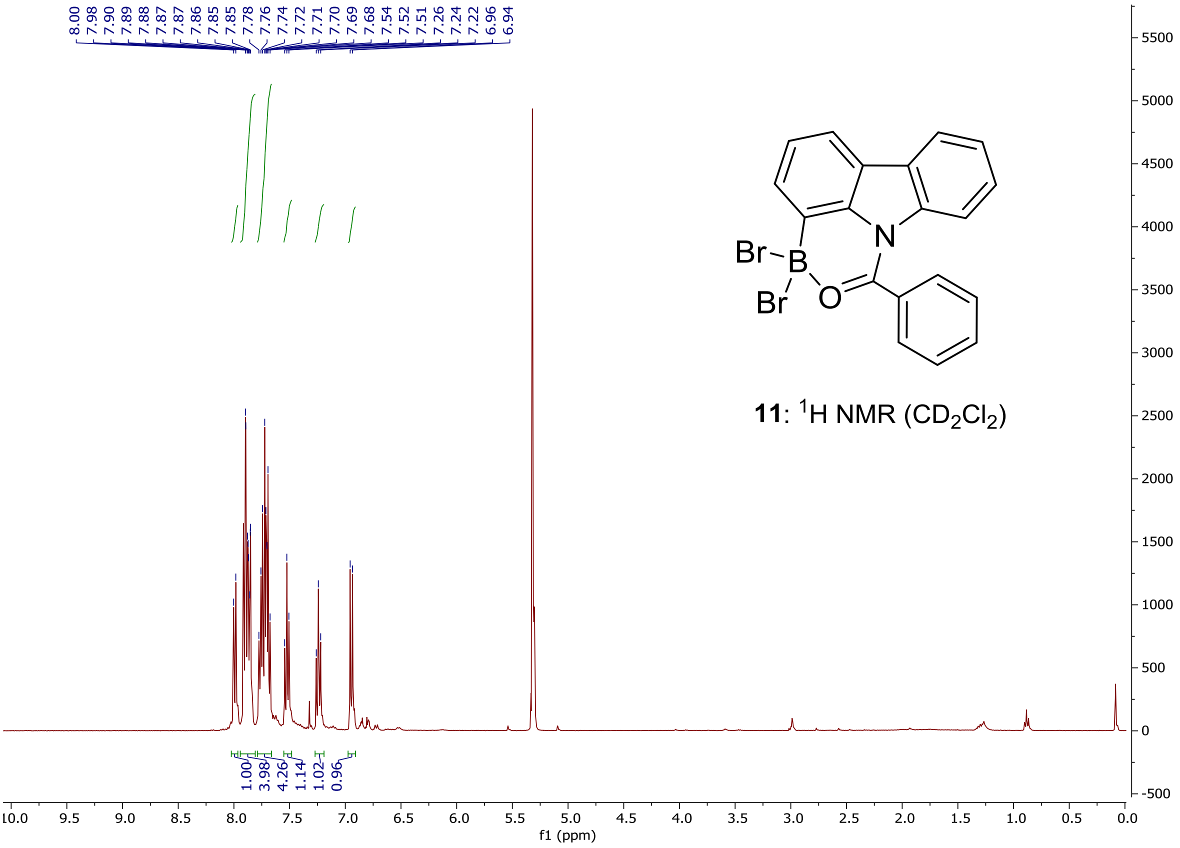

11: ^{1}H NMR (CD_2Cl_2)

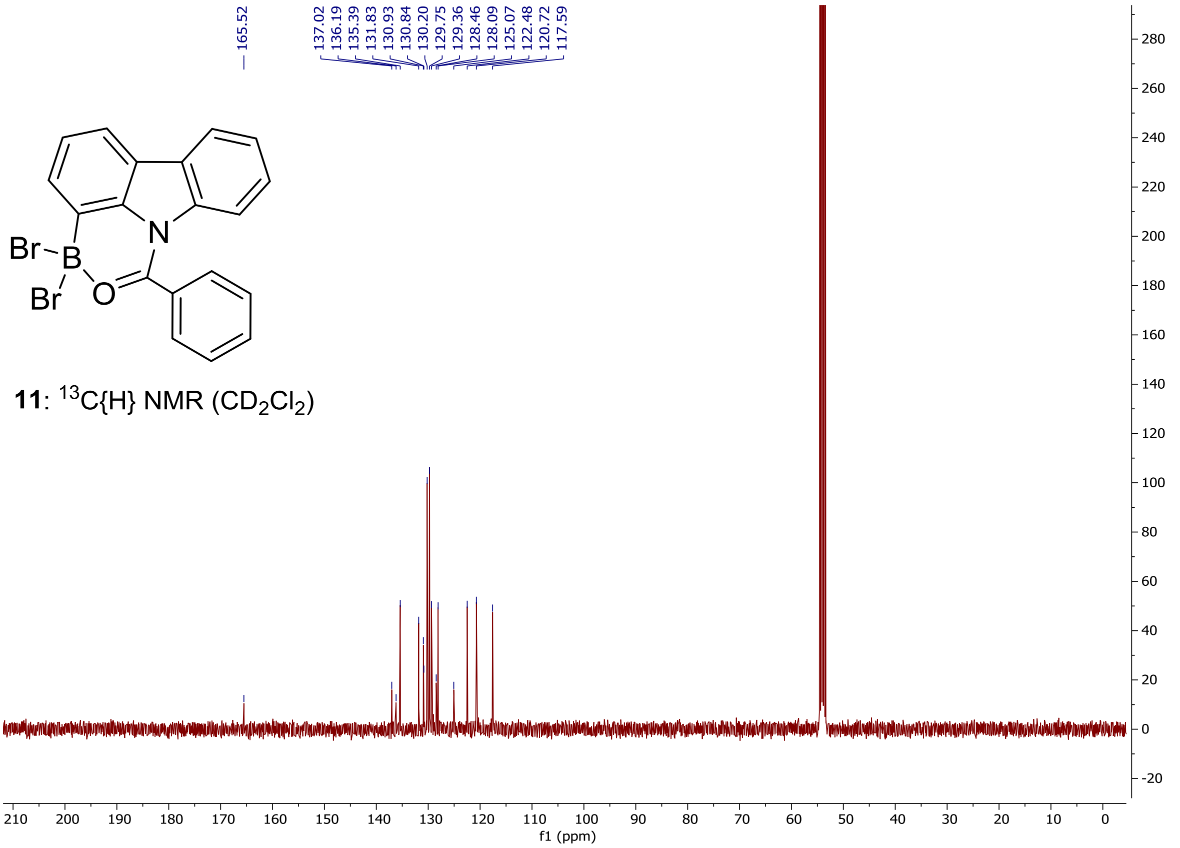

11: $^{13}C\{H\}$ NMR (CD_2Cl_2)

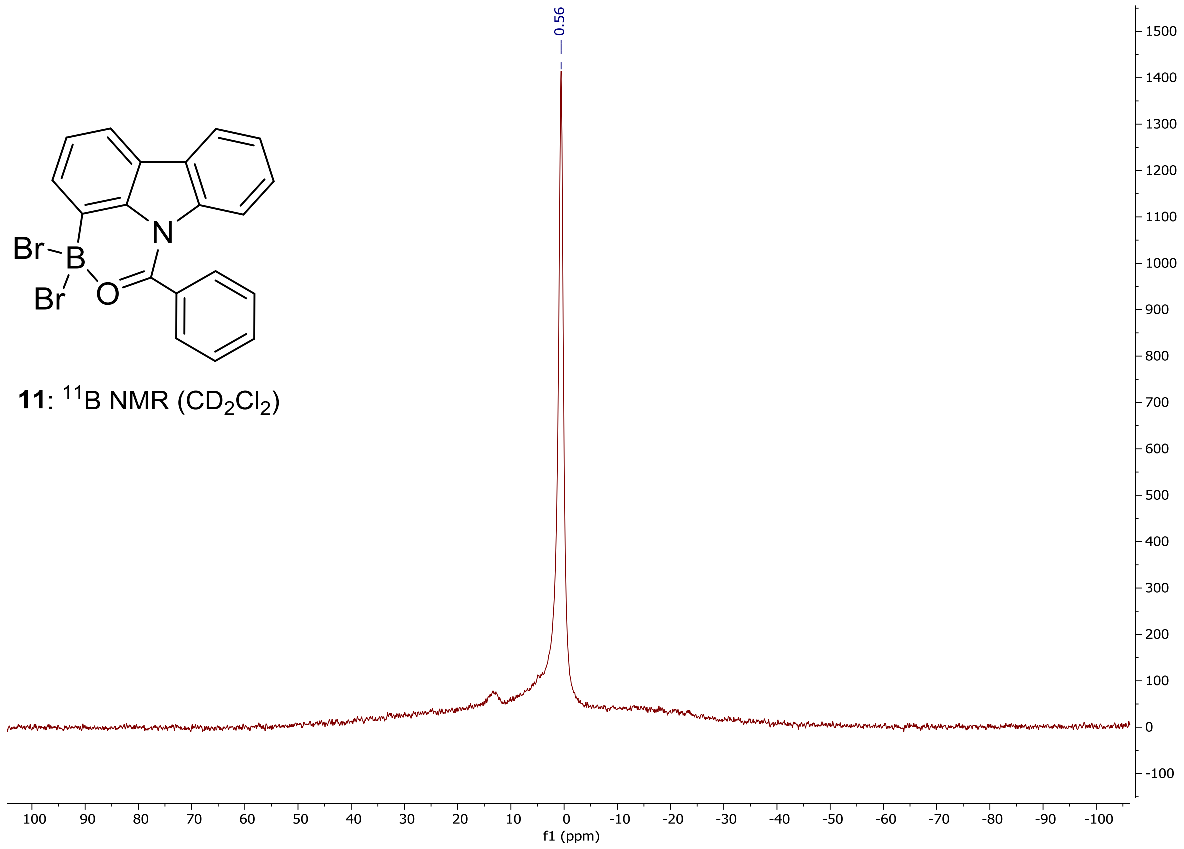

11: ^{11}B NMR (CD_2Cl_2)

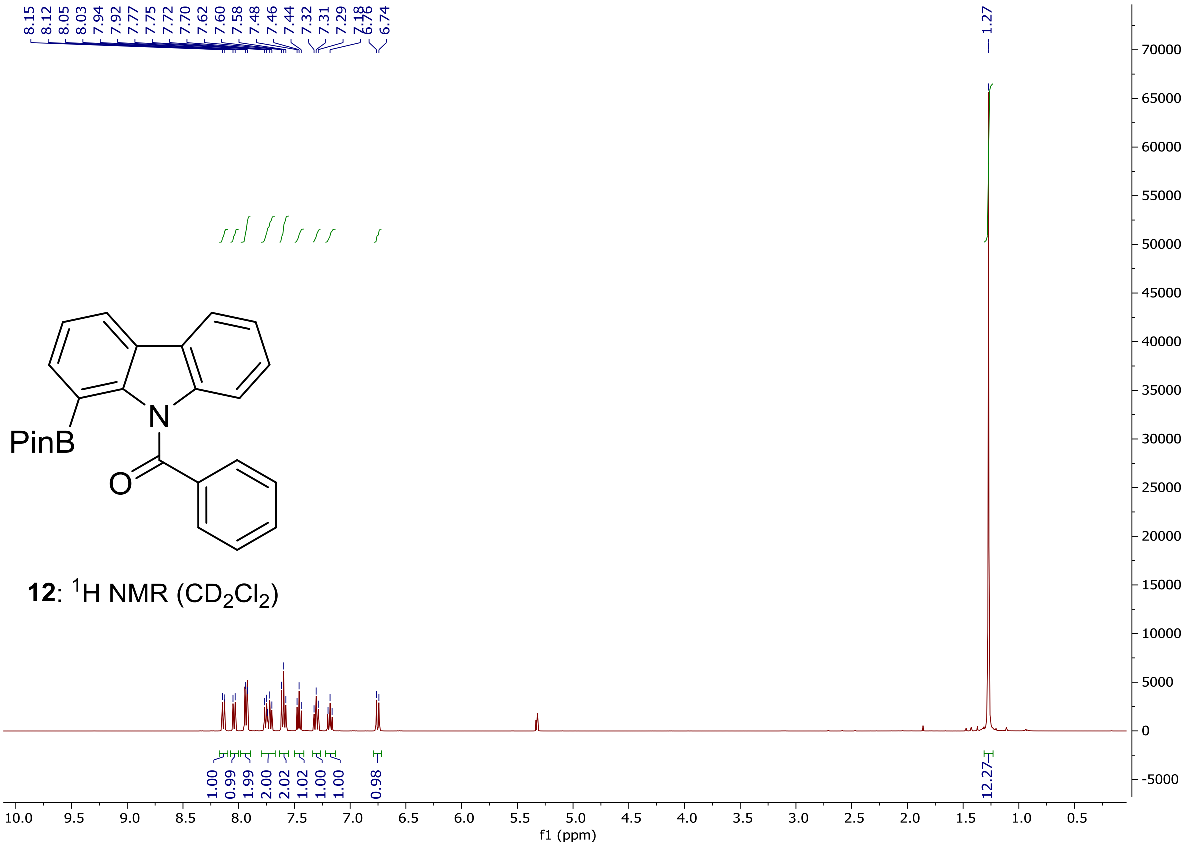

12: ^{1}H NMR (CD_2Cl_2)

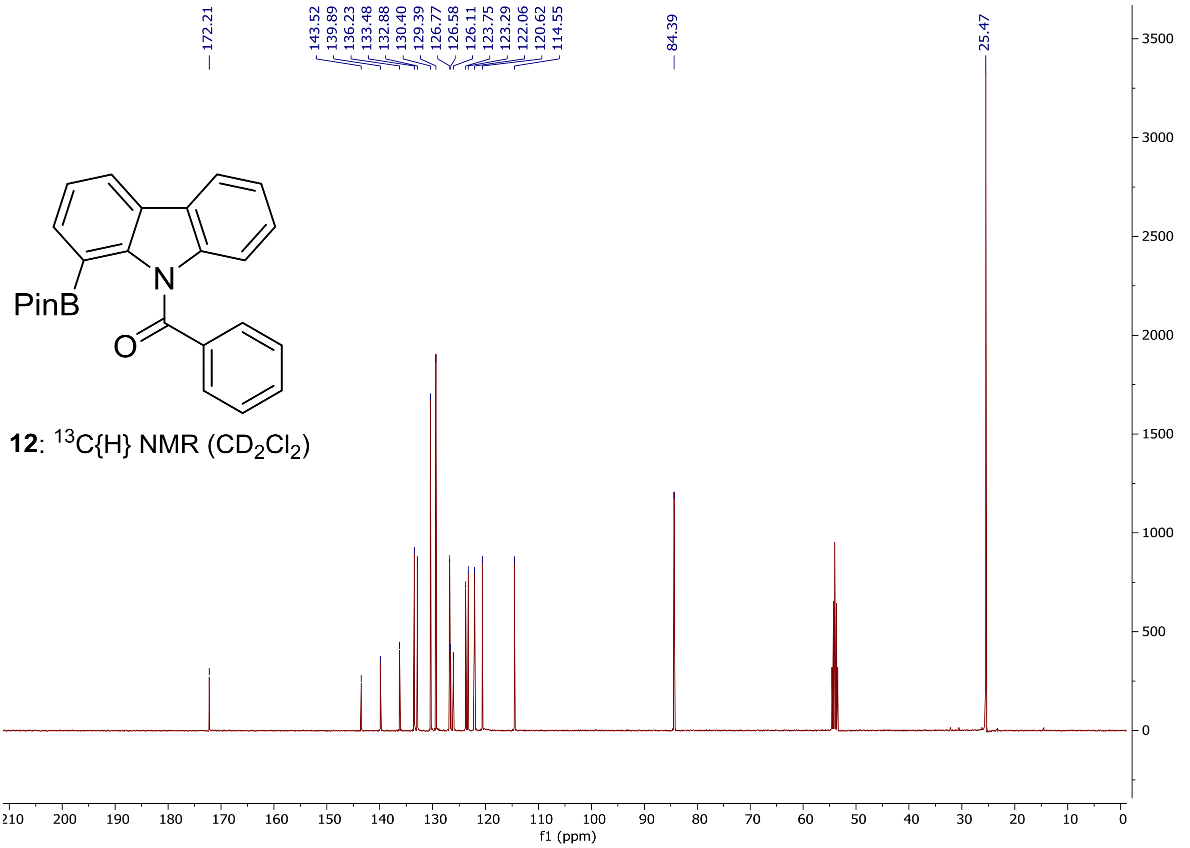

12: $^{13}C\{H\}$ NMR (CD_2Cl_2)

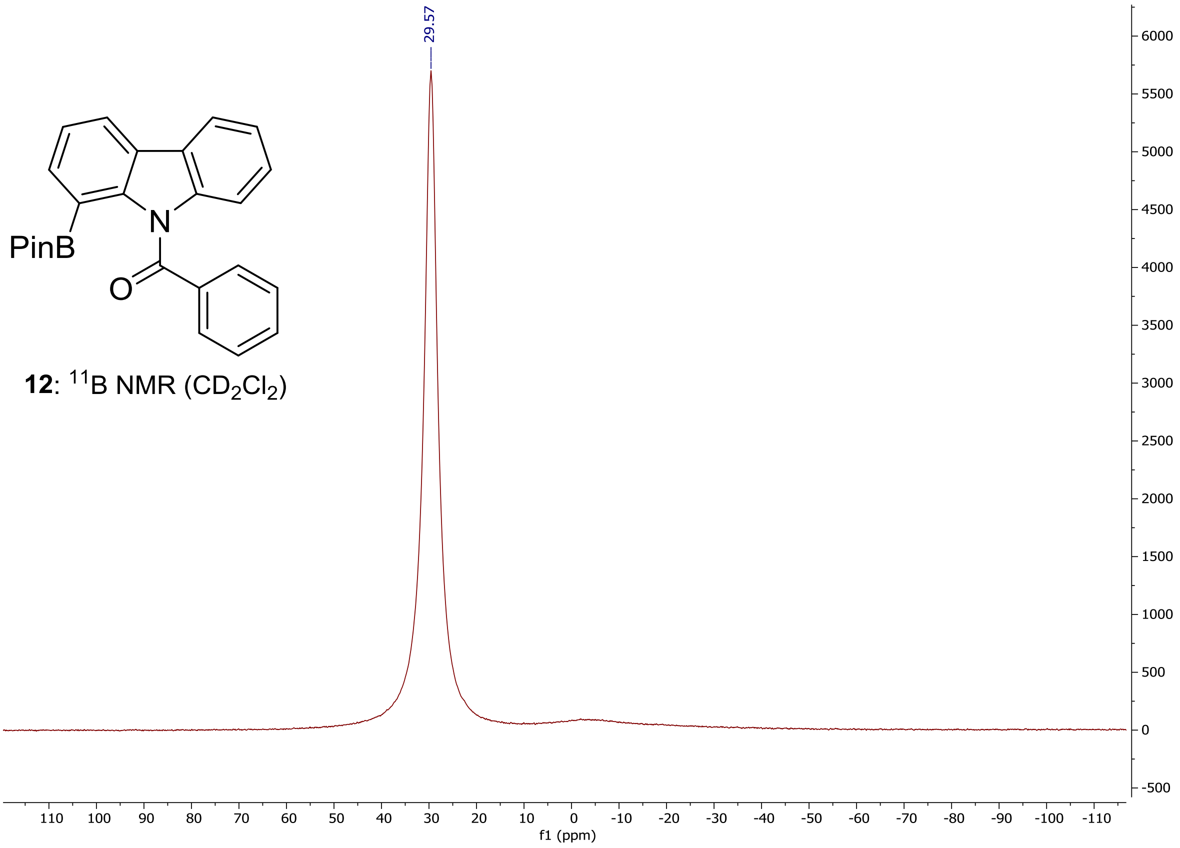

12: ^{11}B NMR (CD_2Cl_2)

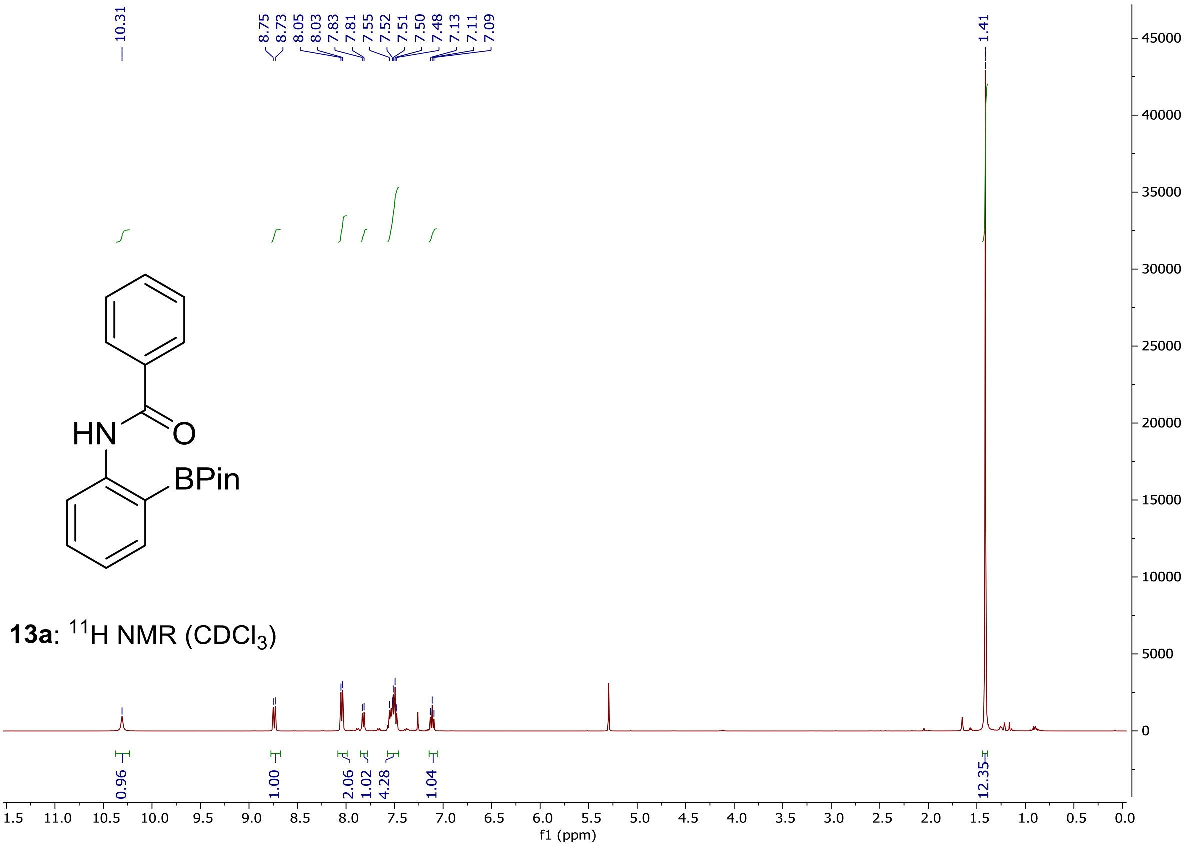

13a: ^{11}H NMR ($CDCl_3$)

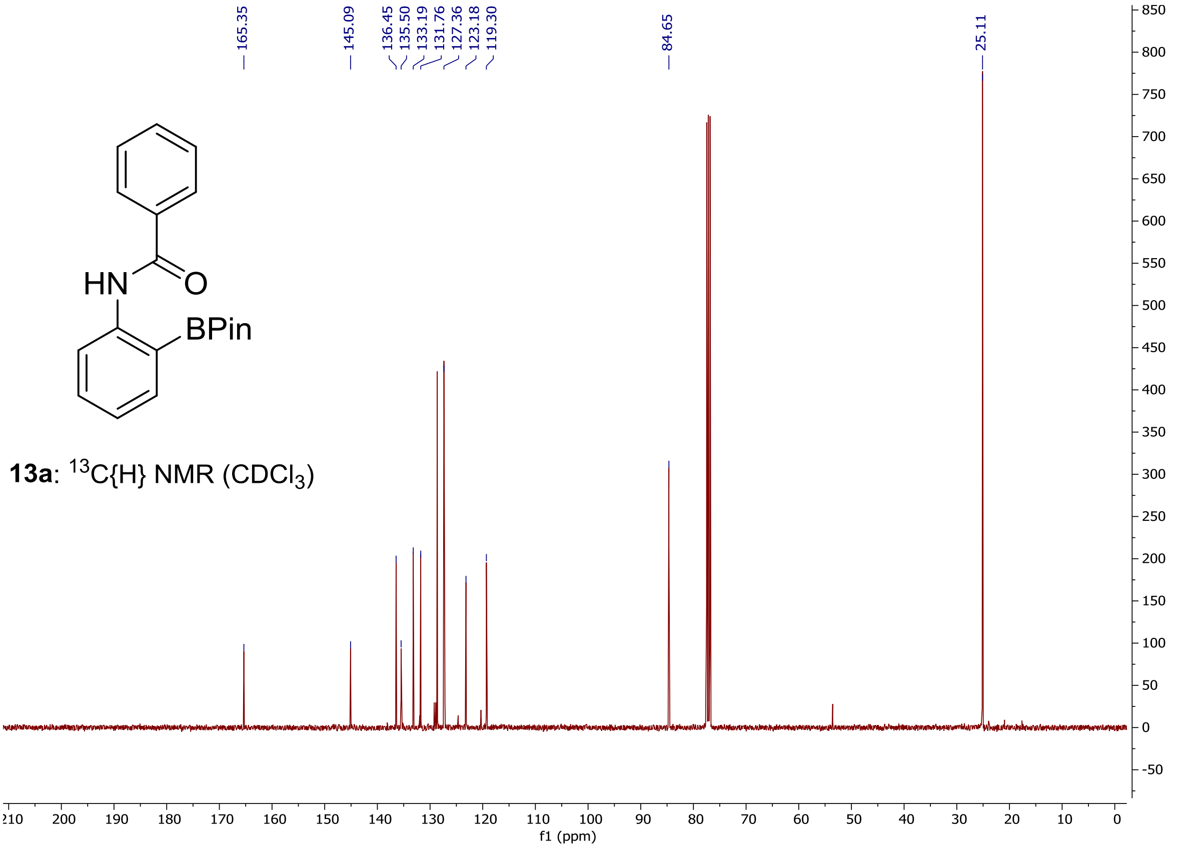

13a: ^{13}C{H} NMR ($CDCl_3$)

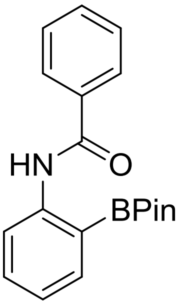

13a: ^{11}B NMR ($CDCl_3$)

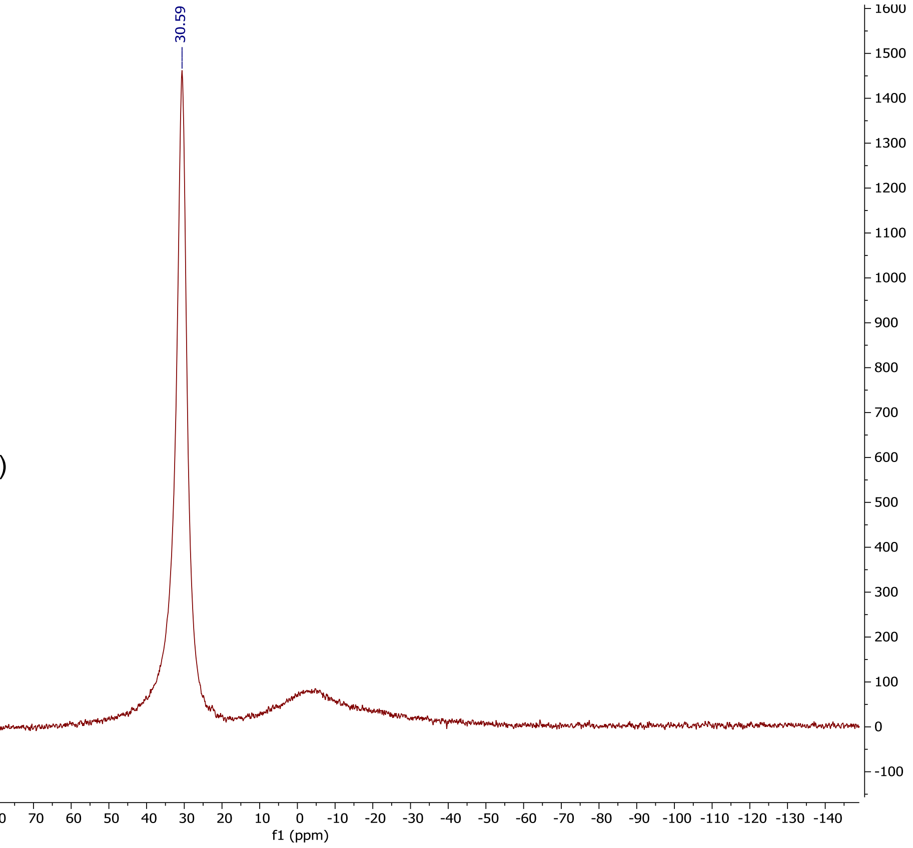

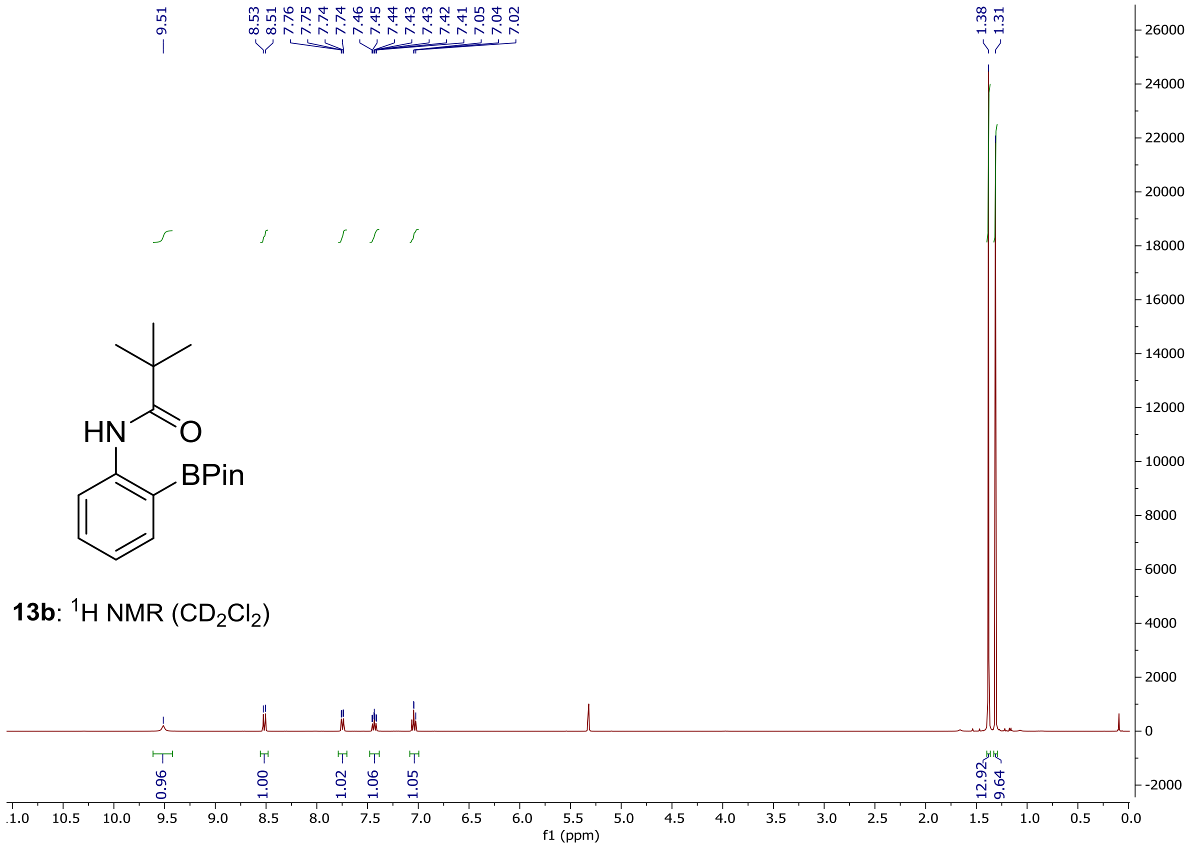
9.51
8.53
8.51
7.76
7.75
7.74
7.74
7.46
7.45
7.44
7.43
7.43
7.42
7.41
7.05
7.04
7.02
1.38
1.31
HN
O
BPin
13b: ^{1}H NMR (CD_2Cl_2)
0.96
1.00
1.02
1.06
1.05
12.92
9.64
f1 (ppm)

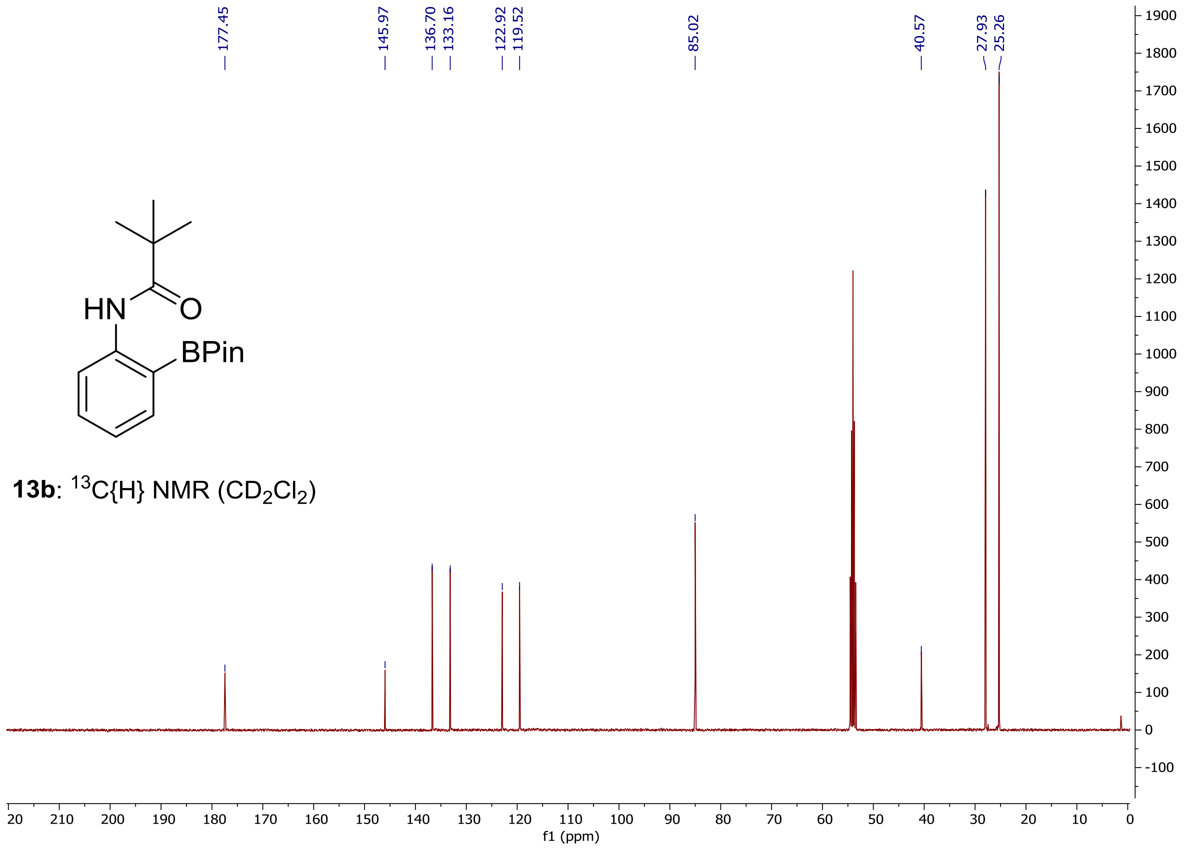
177.45
145.97
136.70
133.16
122.92
119.52
85.02
40.57
27.93
25.26
HN
O
BPin
13b: ^{13}C{H} NMR (CD_2Cl_2)
f1 (ppm)

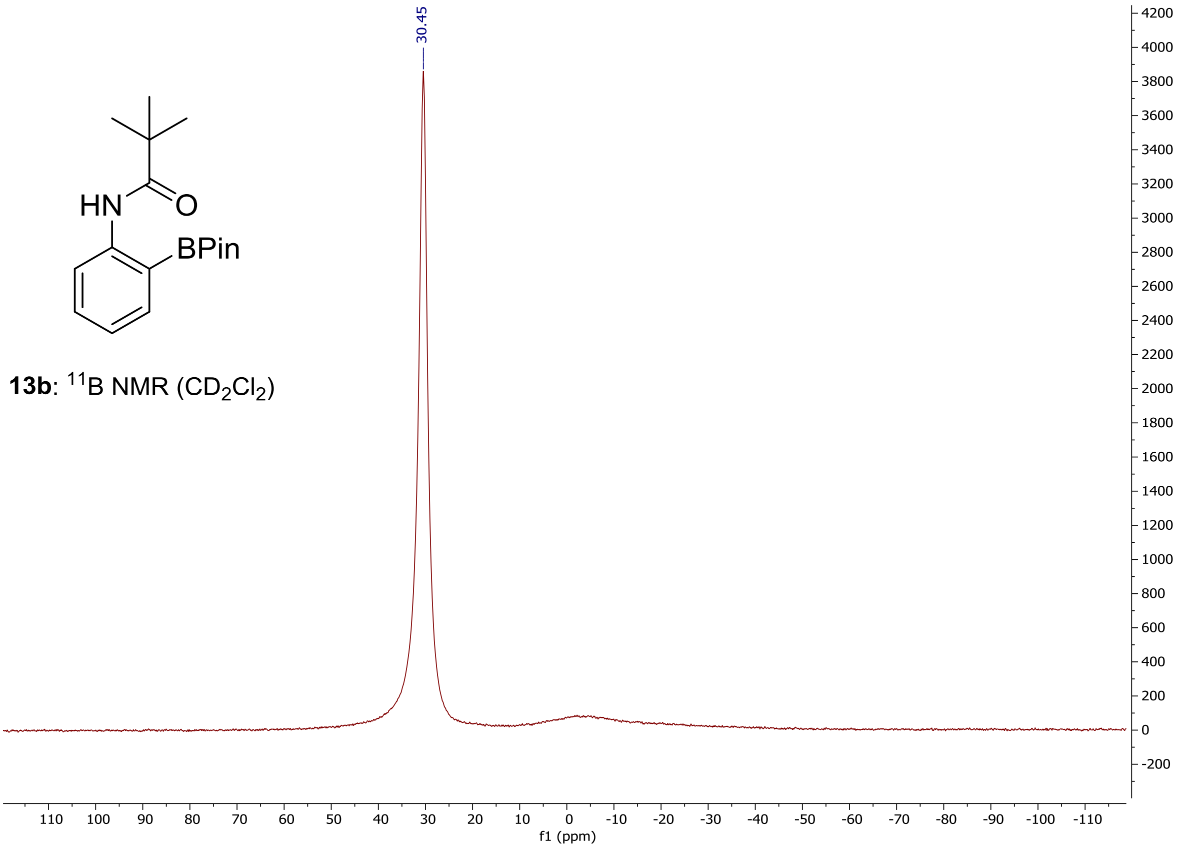

13b: ^{11}B NMR (CD_2Cl_2)

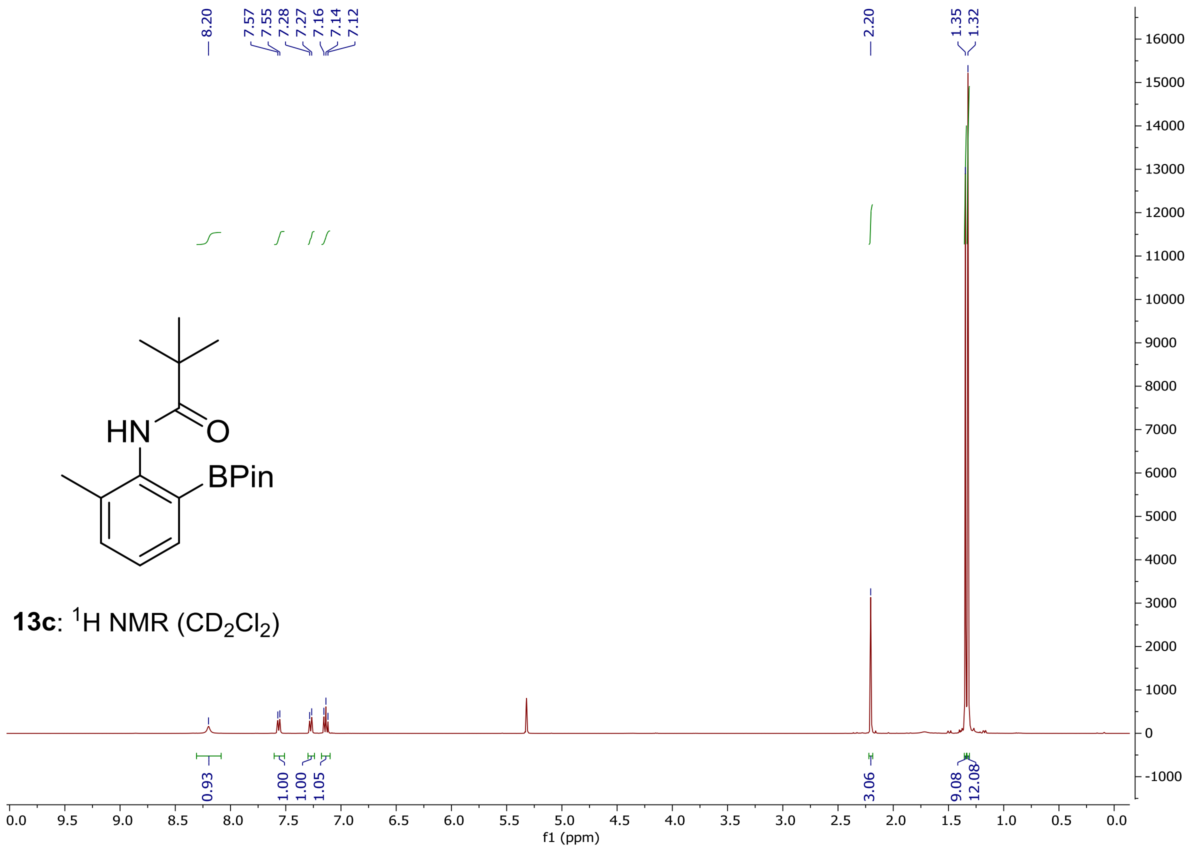

13c: ^{1}H NMR (CD_2Cl_2)

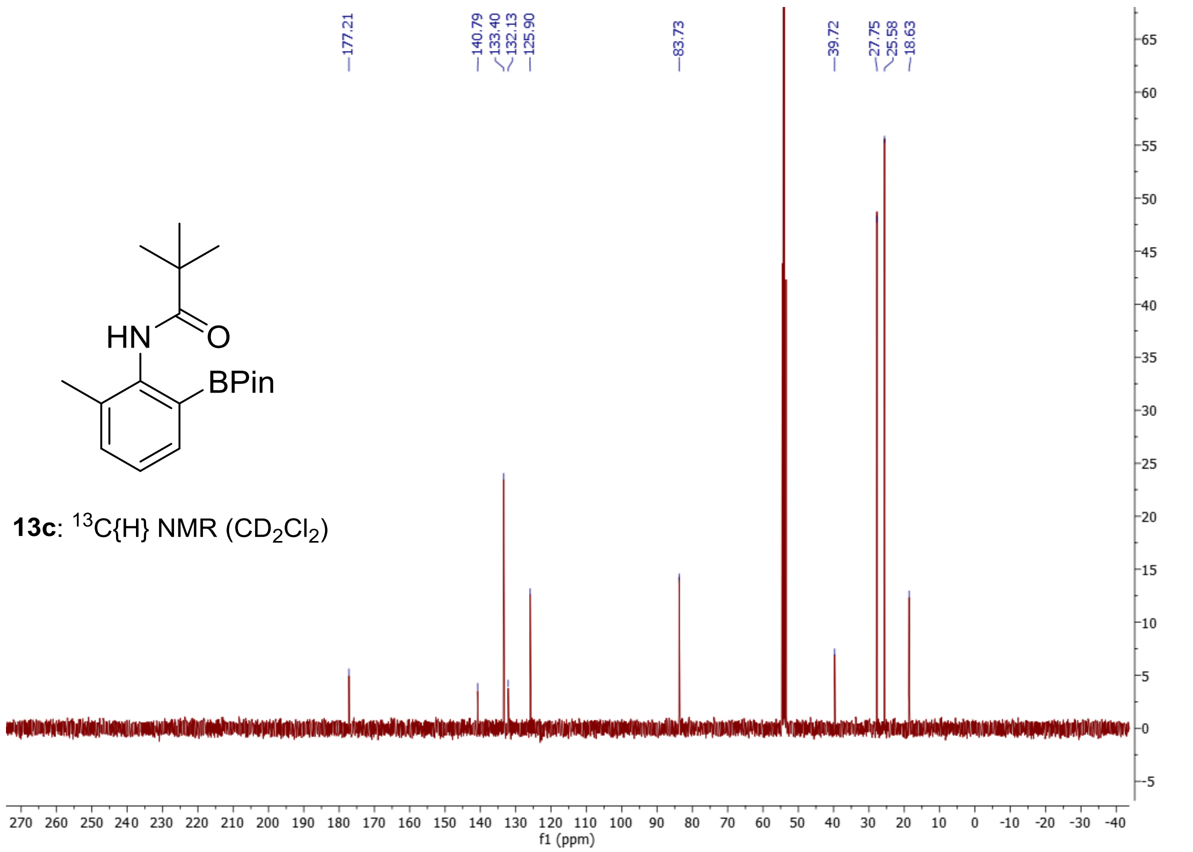

13c: ^{13}C{H} NMR (CD_2Cl_2)

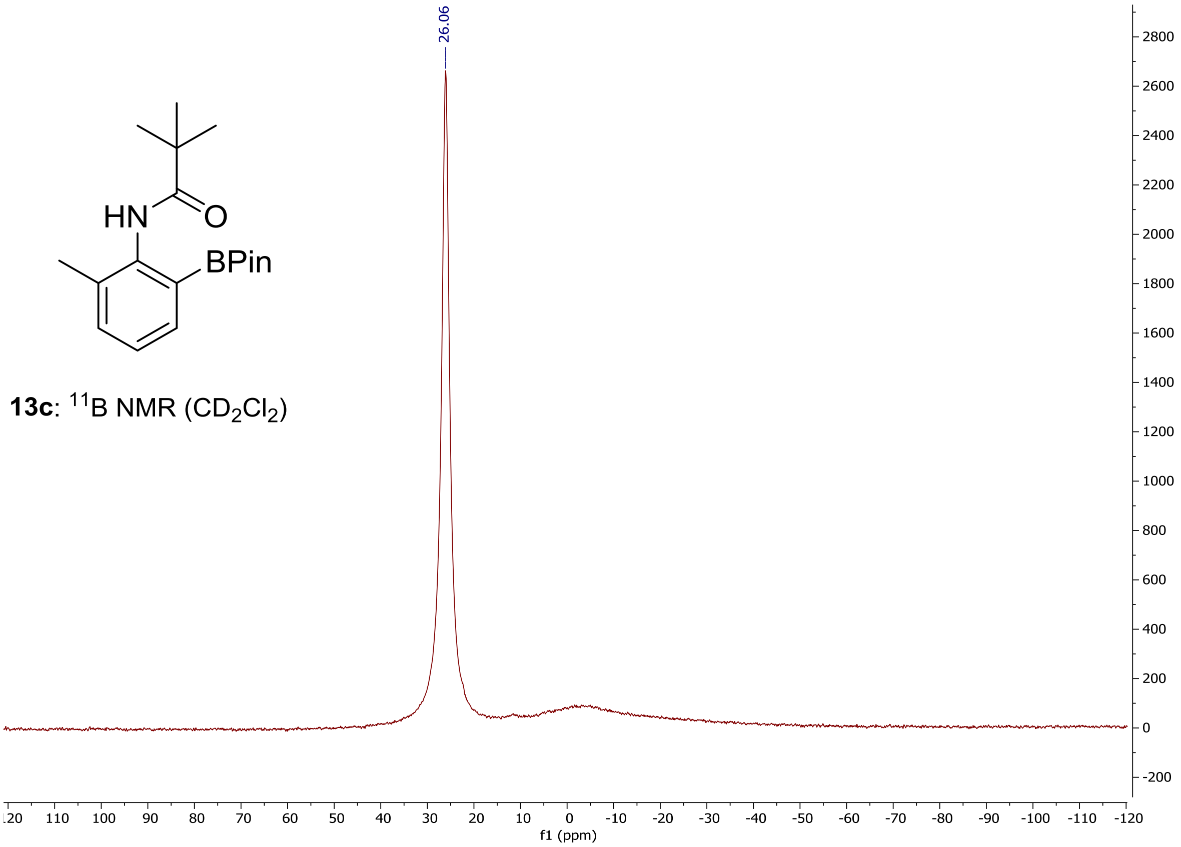

13c: ^{11}B NMR (CD_2Cl_2)

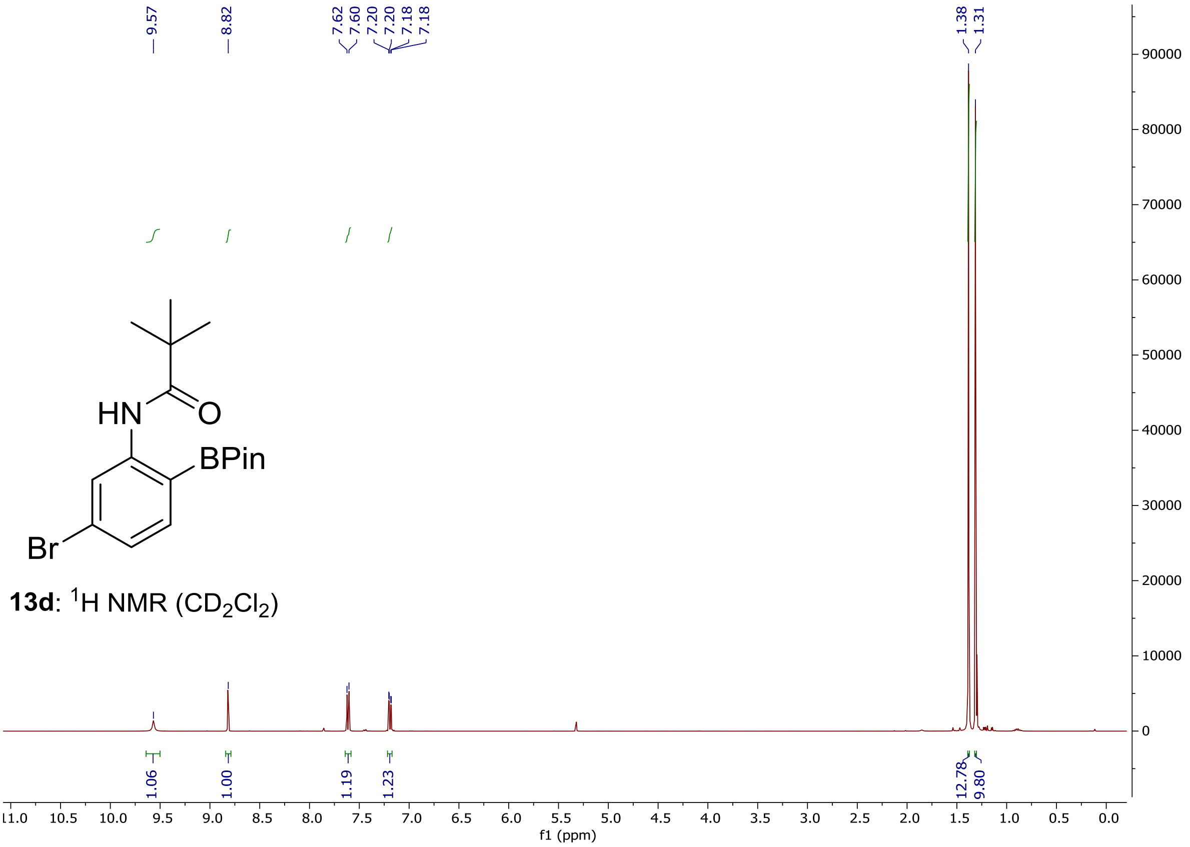

13d: ^{1}H NMR (CD_2Cl_2)

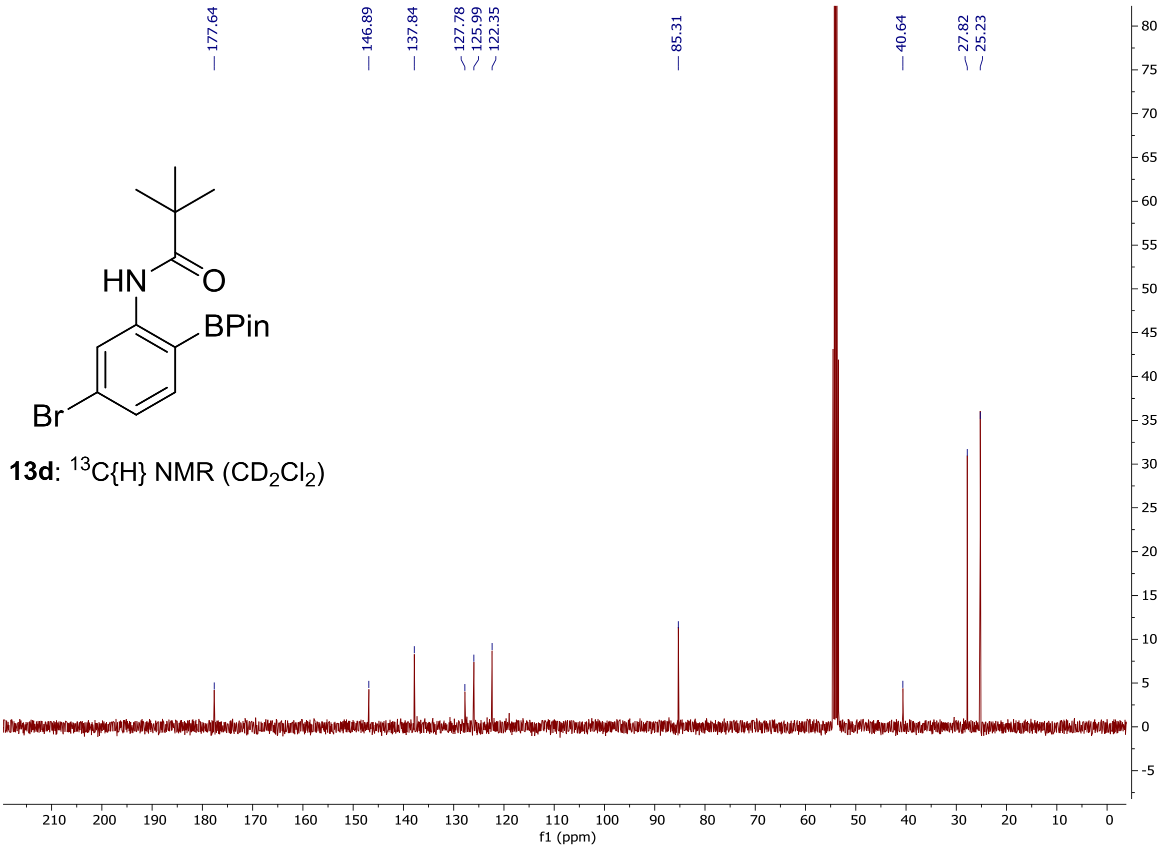

13d: $^{13}C\{H\}$ NMR (CD_2Cl_2)

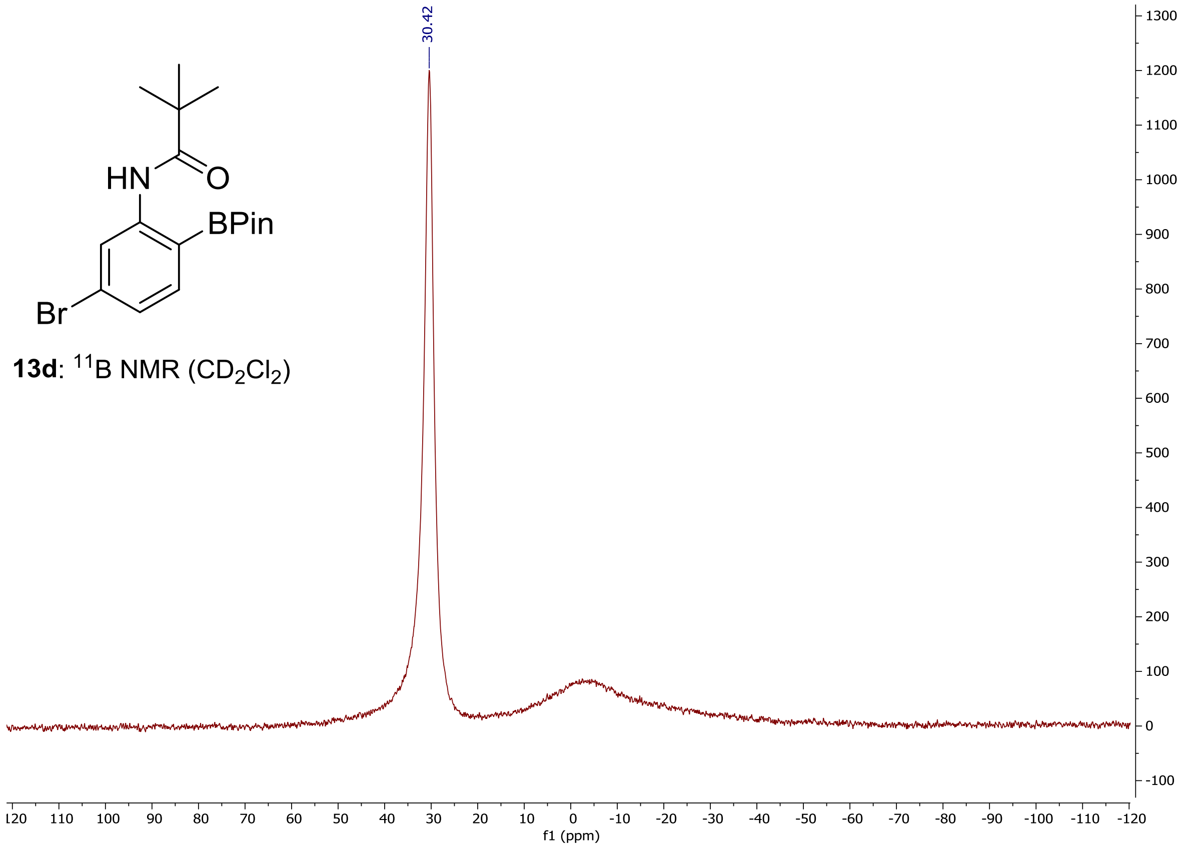

30.42
HN
O
BPin
Br
13d: 11B NMR (CD2Cl2)
1300
1200
1100
1000
900
800
700
600
500
400
300
200
100
0
-100
120 110 100 90 80 70 60 50 40 30 20 10 0 -10 -20 -30 -40 -50 -60 -70 -80 -90 -100 -110 -120
f1 (ppm)

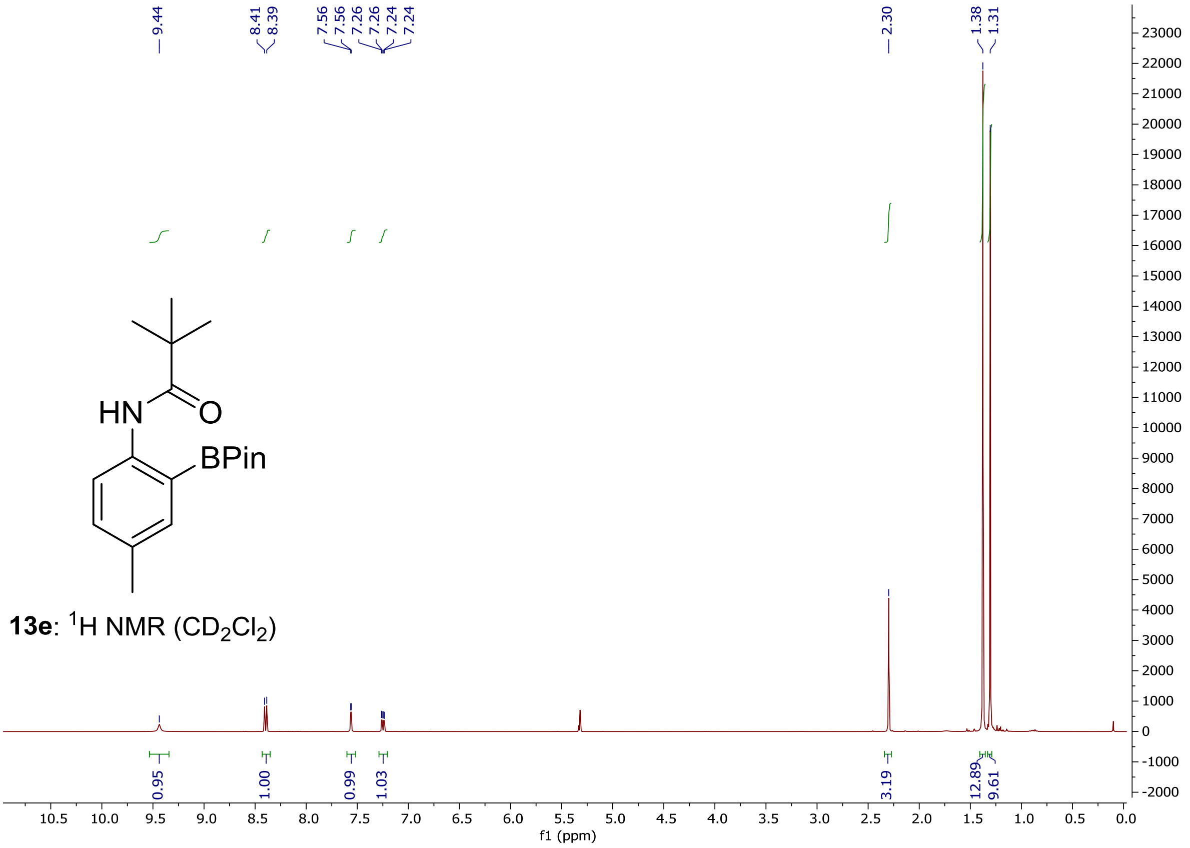

13e: ^{1}H NMR (CD_2Cl_2)

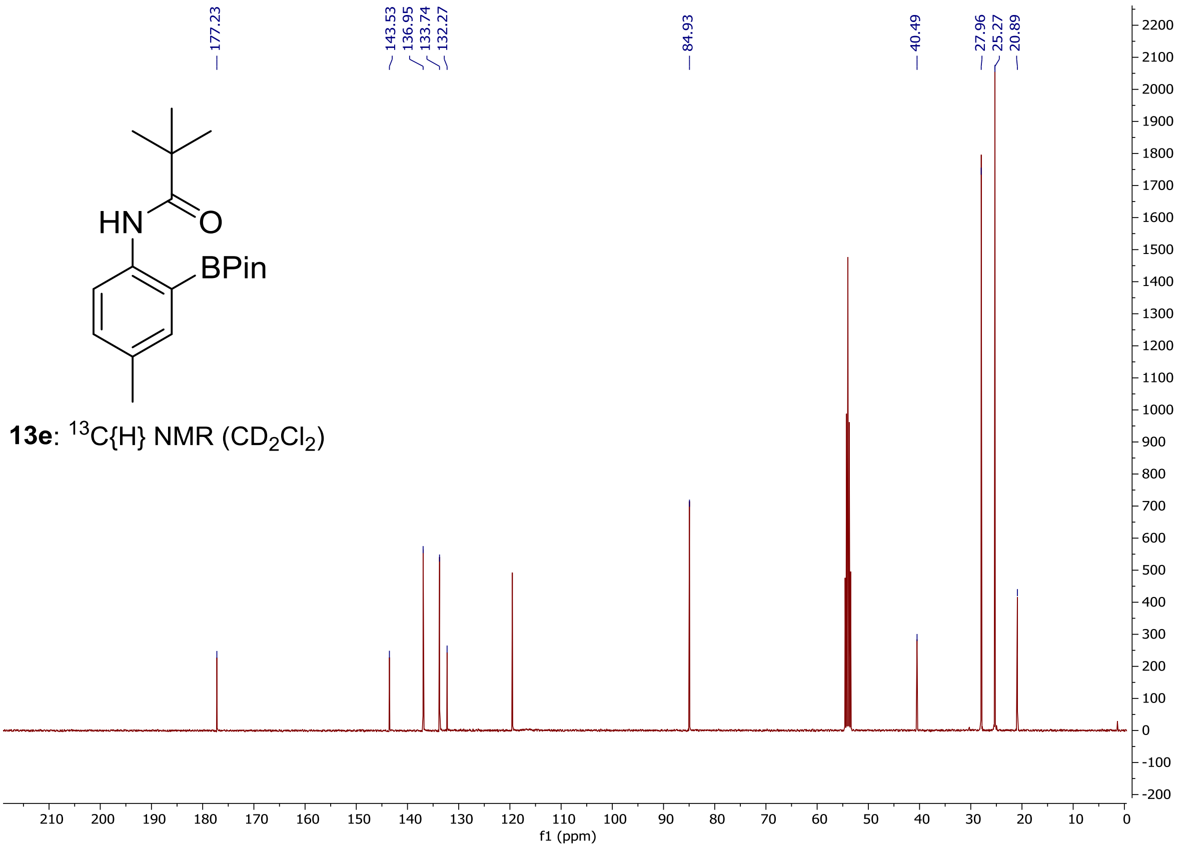

13e: $^{13}C\{H\}$ NMR (CD_2Cl_2)

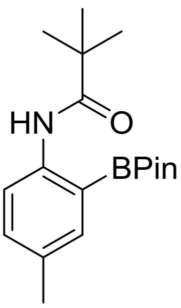

13e: ^{11}B NMR (CD_2Cl_2)

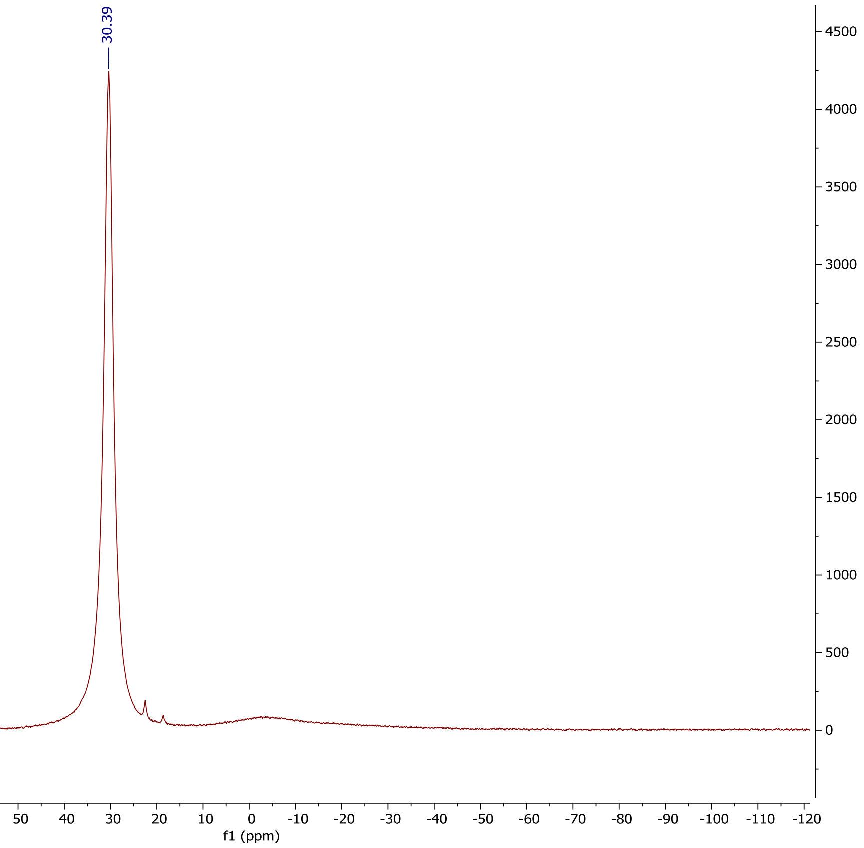

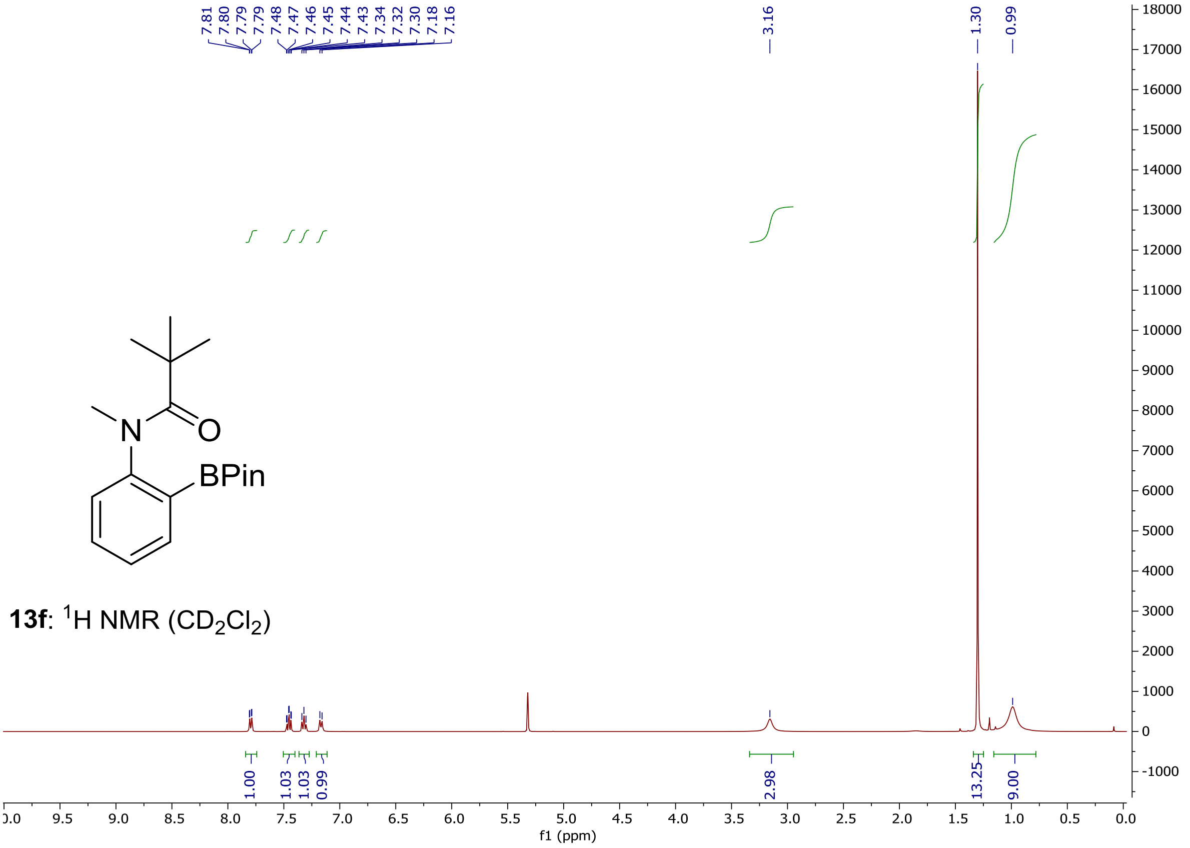

13f: ^{1}H NMR (CD_2Cl_2)

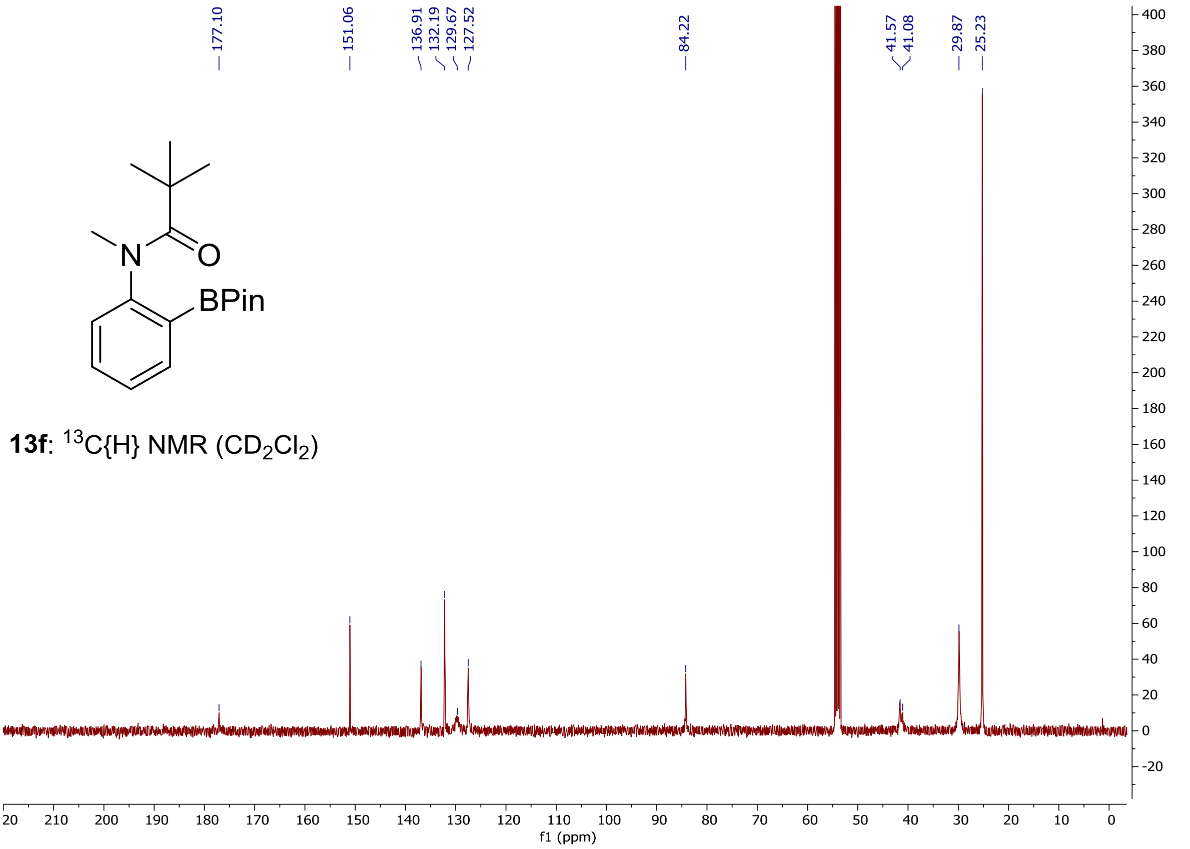

13f: $^{13}C\{H\}$ NMR (CD_2Cl_2)

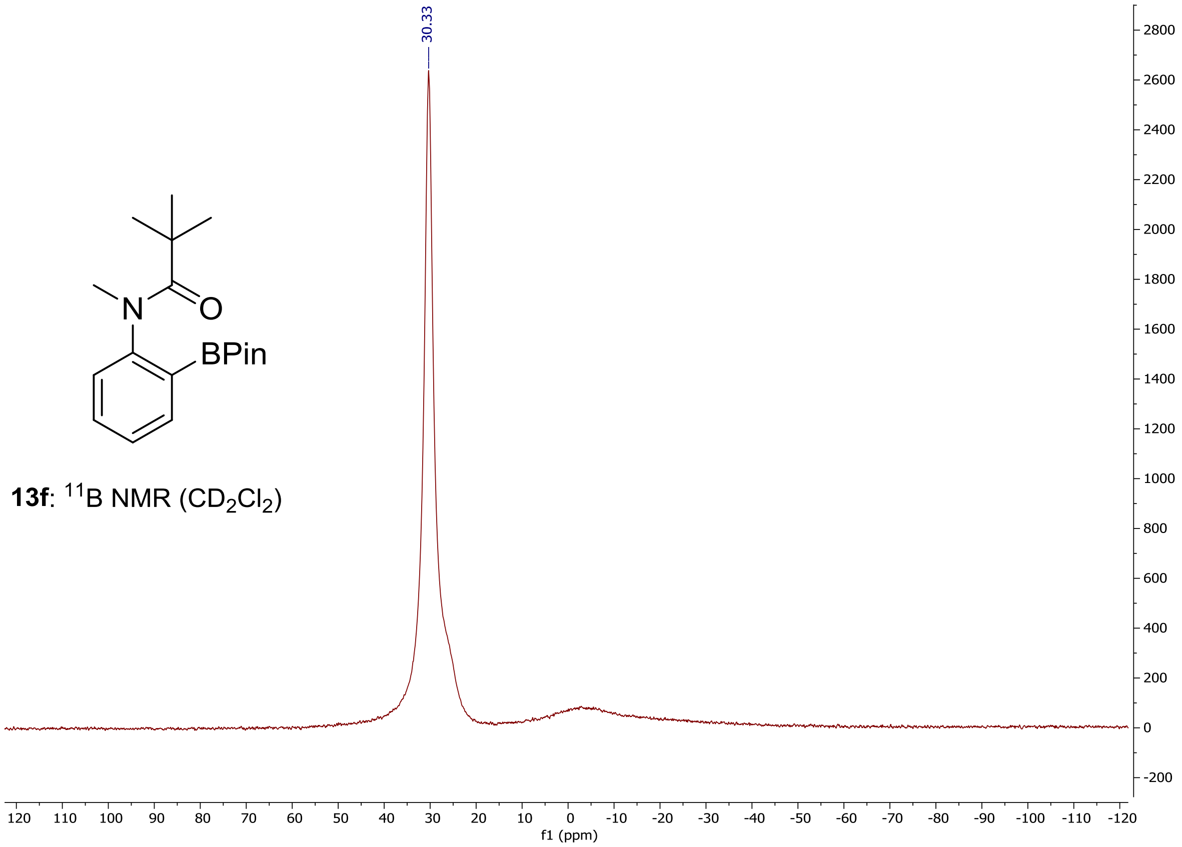

13f: ^{11}B NMR (CD_2Cl_2)

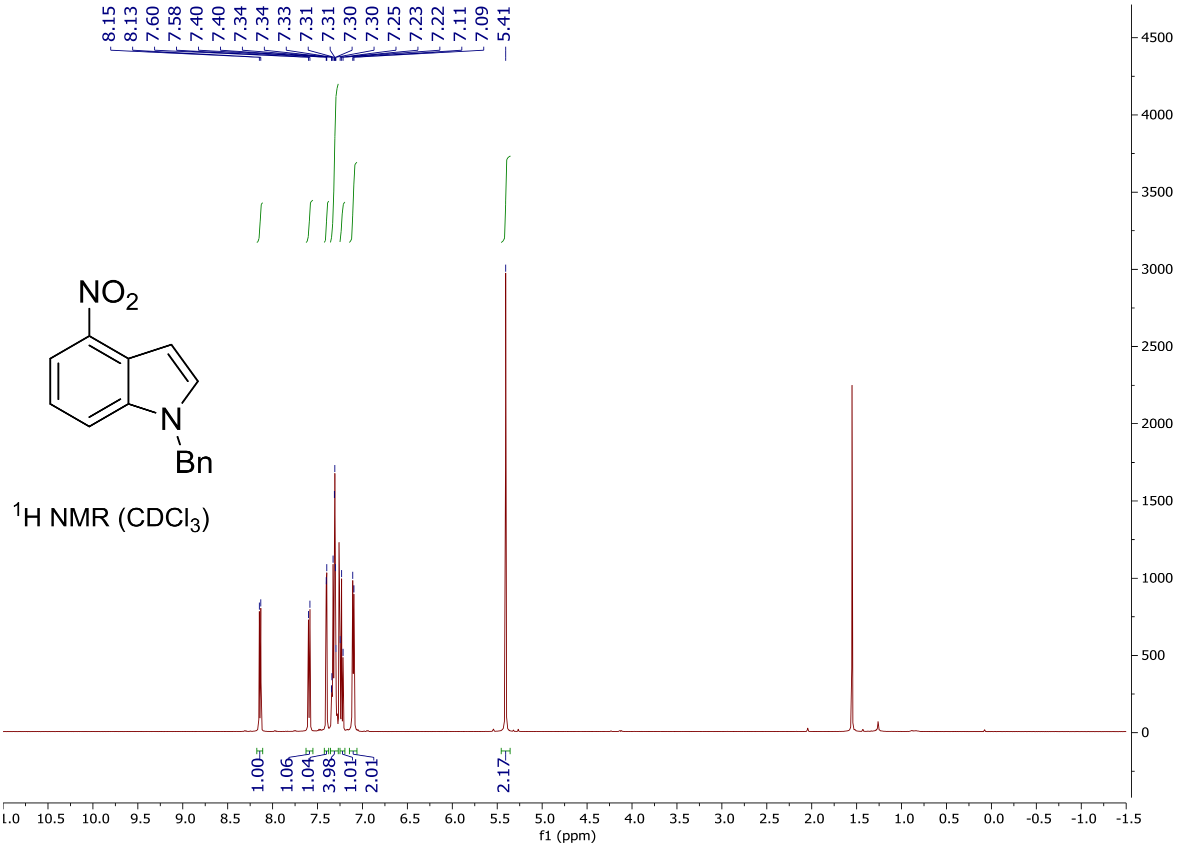
8.15
8.13
7.60
7.58
7.40
7.40
7.34
7.34
7.33
7.31
7.31
7.30
7.30
7.25
7.23
7.22
7.11
7.09
5.41
NO2
N
Bn
1H NMR (CDCl3)
1.00
1.06
1.04
3.98
1.01
2.01
2.17
f1 (ppm)

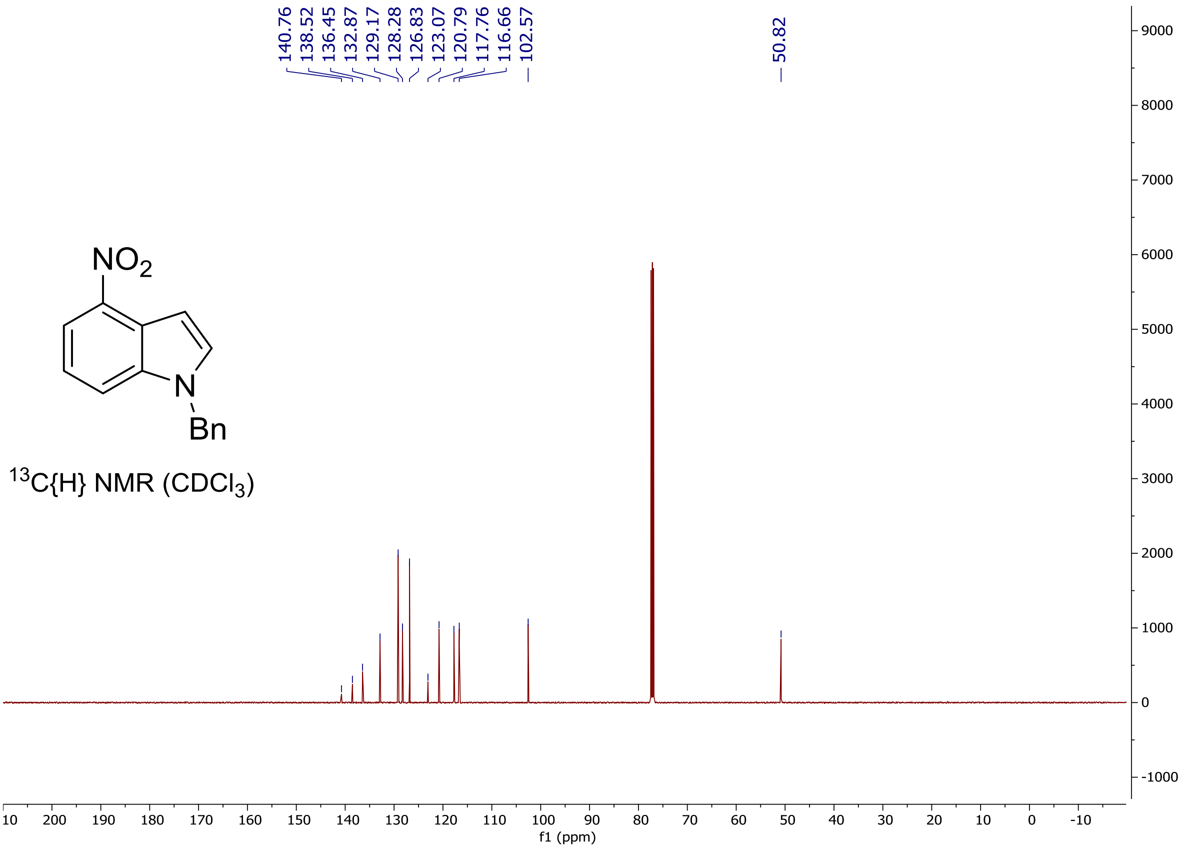
140.76
138.52
136.45
132.87
129.17
128.28
126.83
123.07
120.79
117.76
116.66
102.57
50.82
NO2
N
Bn
13C{H} NMR (CDCl3)
f1 (ppm)

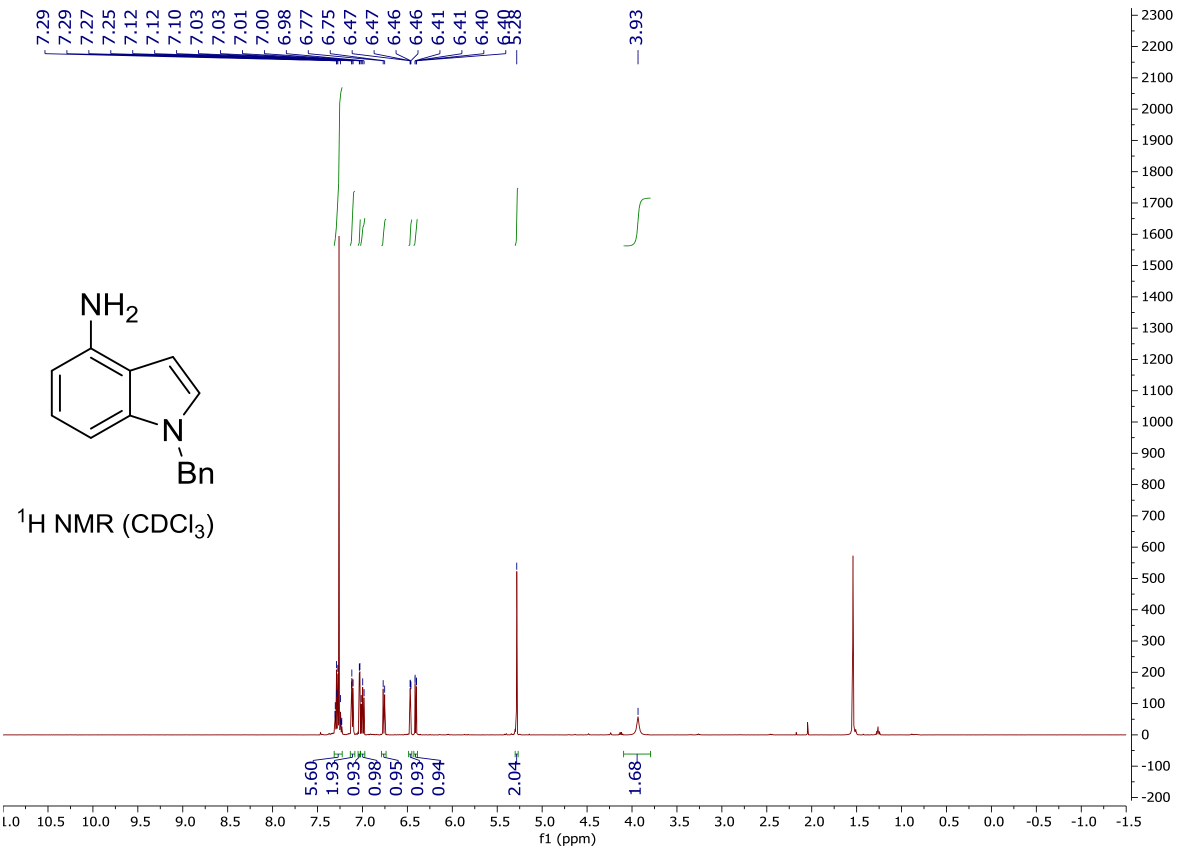
NH2
N
Bn
1H NMR (CDCl3)
7.29
7.29
7.27
7.25
7.12
7.12
7.10
7.03
7.03
7.01
7.00
6.98
6.77
6.75
6.47
6.47
6.46
6.46
6.41
6.41
6.40
6.40
5.28
3.93
5.60
1.93
0.93
0.98
0.95
0.93
0.94
2.04
1.68
f1 (ppm)

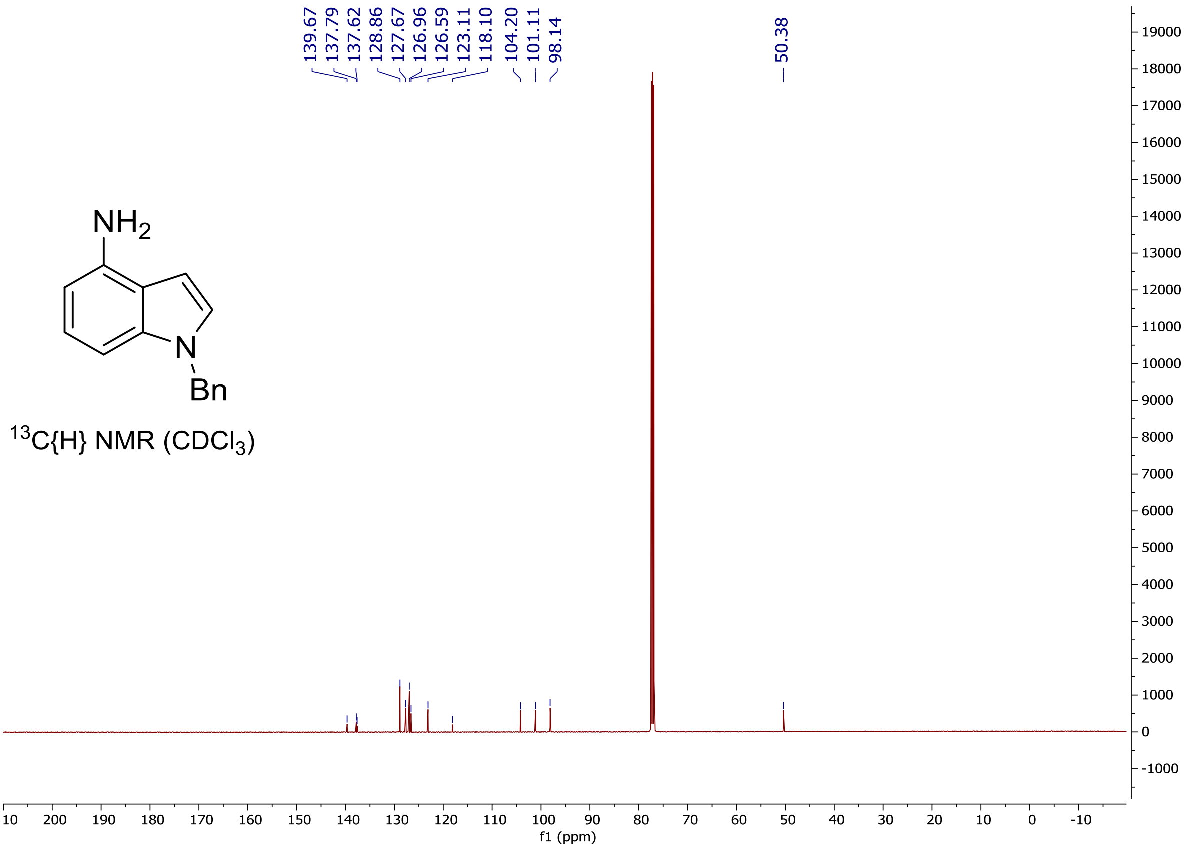
NH2
N
Bn
13C{H} NMR (CDCl3)
139.67
137.79
137.62
128.86
127.67
126.96
126.59
123.11
118.10
104.20
101.11
98.14
50.38
f1 (ppm)

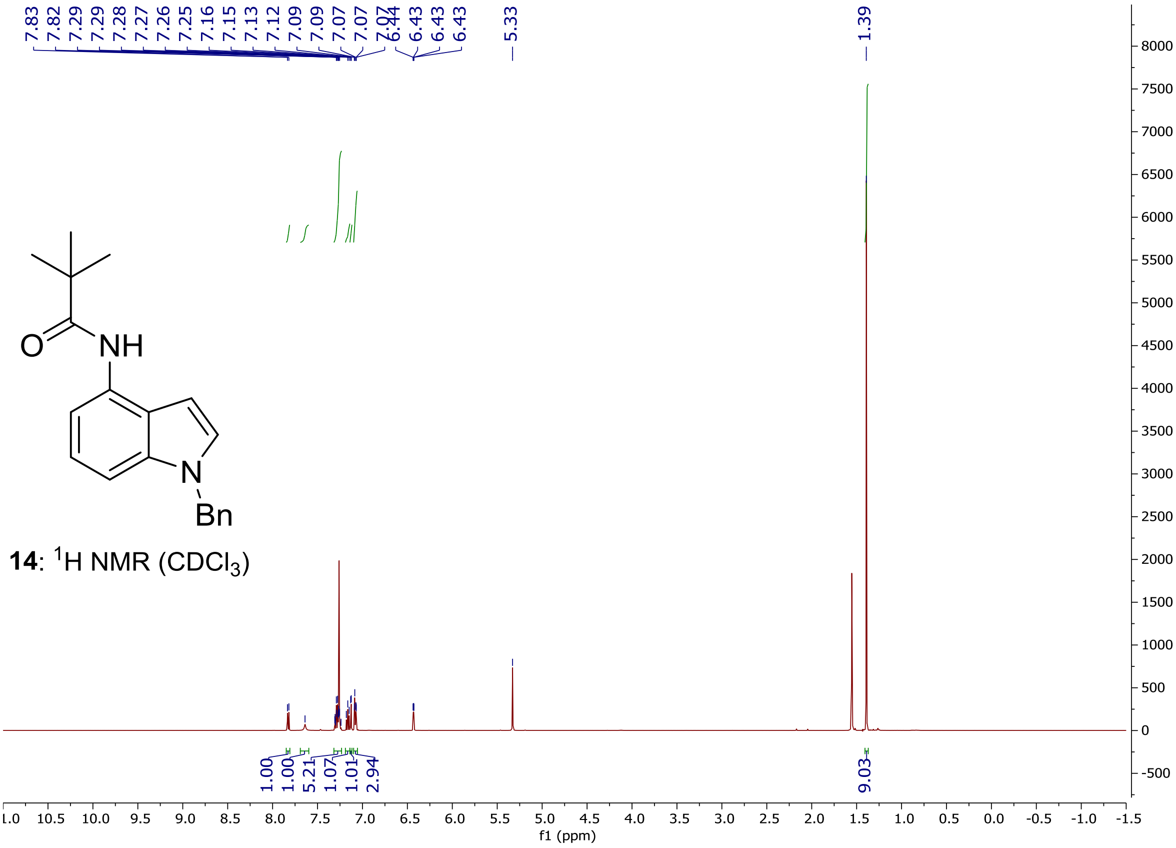

14: ^{1}H NMR ($CDCl_3$)

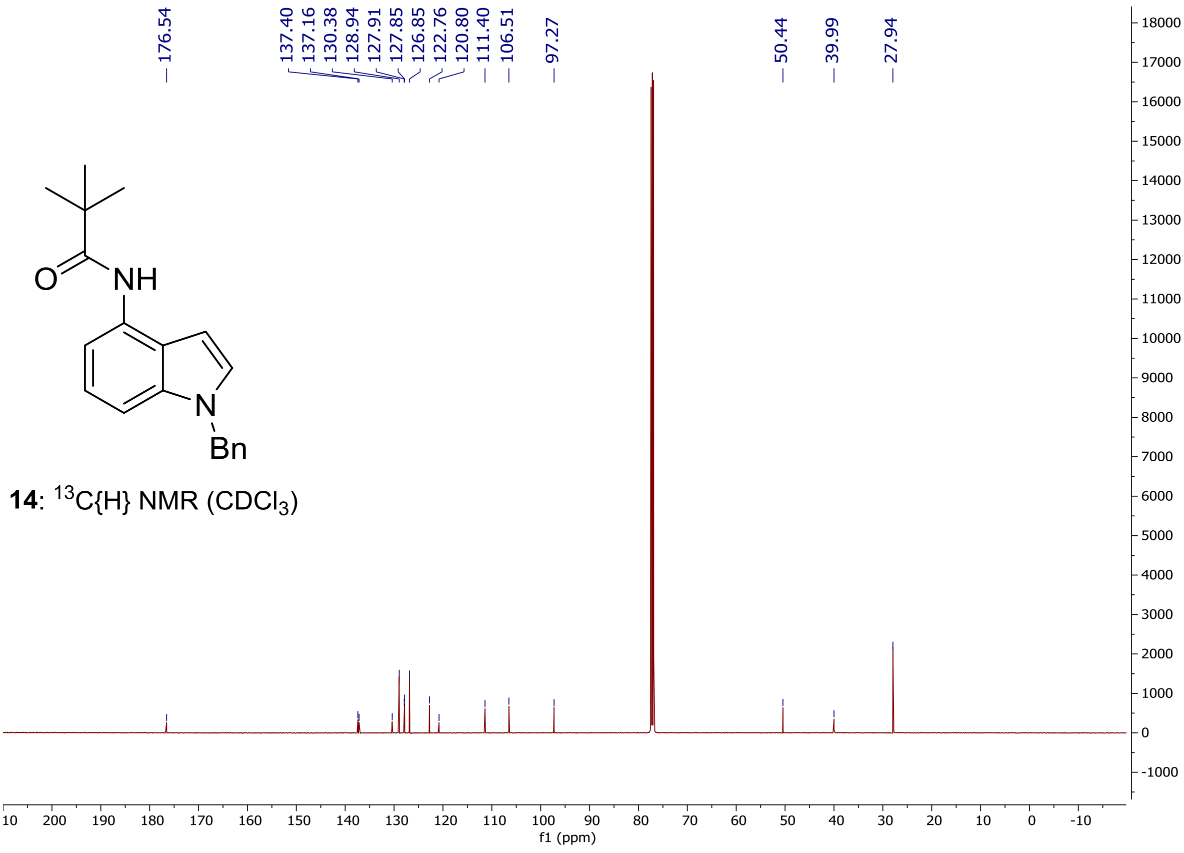

14: $^{13}C\{H\}$ NMR ($CDCl_3$)

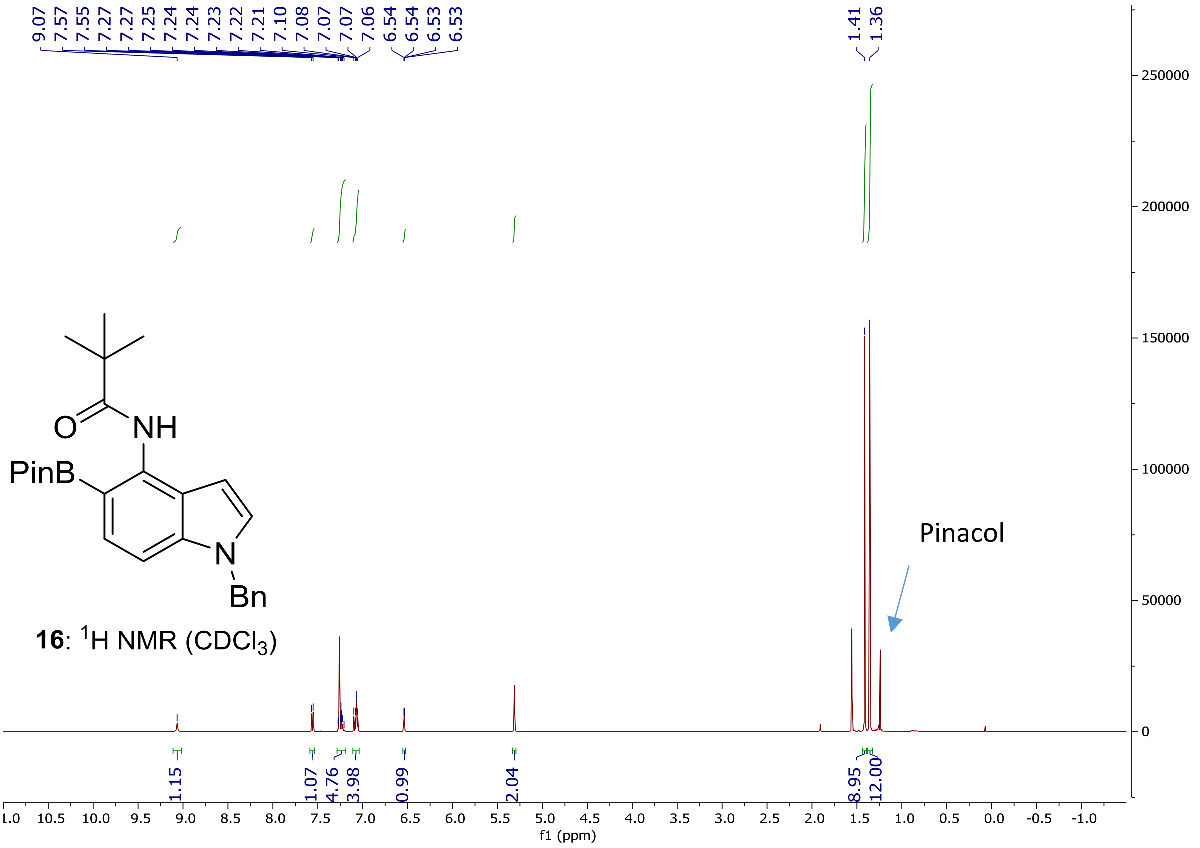
9.07 7.57 7.55 7.27 7.27 7.25 7.24 7.24 7.23 7.22 7.21 7.10 7.08 7.07 7.07 7.06 6.54 6.54 6.53 6.53
1.41 1.36
PinB
O
NH
N
Bn
16: ¹H NMR (CDCl3)
Pinacol
1.15 1.07 4.76 3.98 0.99 2.04 8.95 12.00
f1 (ppm)

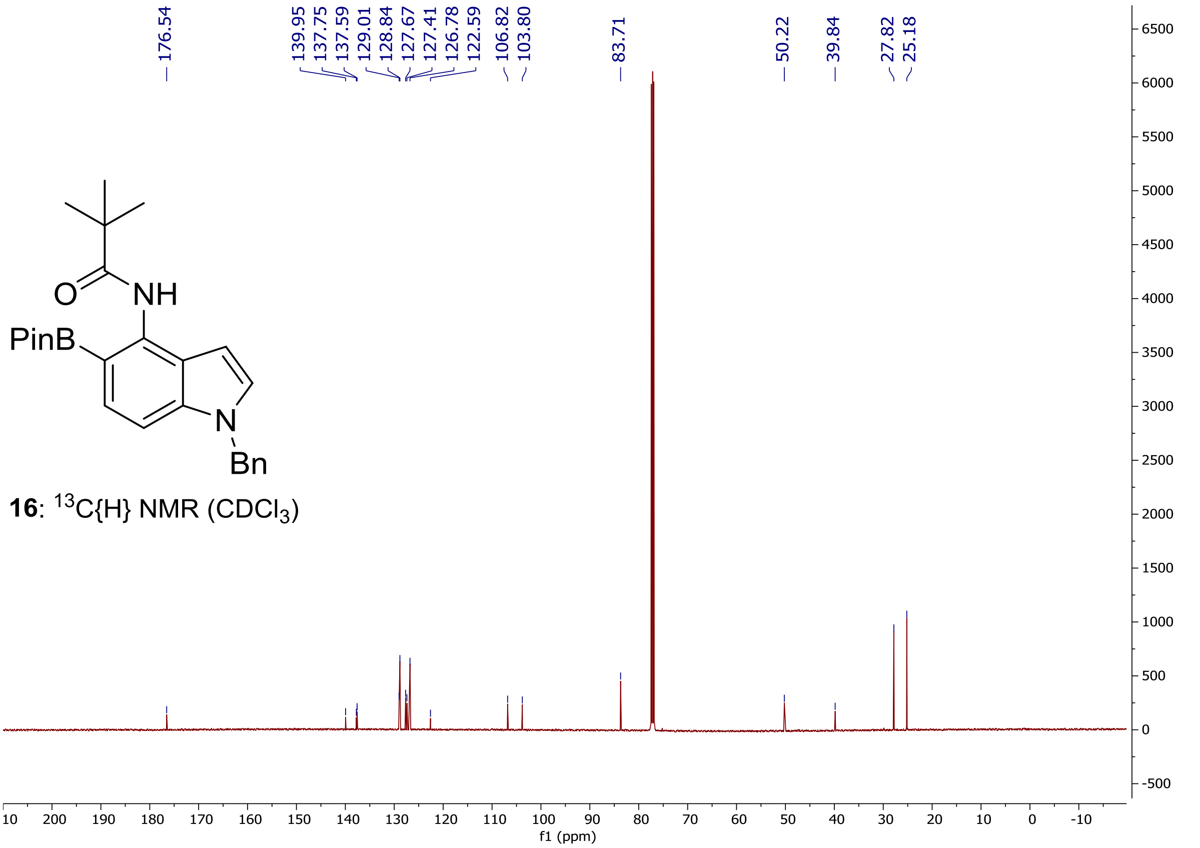
176.54
139.95 137.75 137.59 129.01 128.84 127.67 127.41 126.78 122.59 106.82 103.80
83.71
50.22
39.84
27.82 25.18
PinB
O
NH
N
Bn
16: ¹³C{H} NMR (CDCl3)
f1 (ppm)

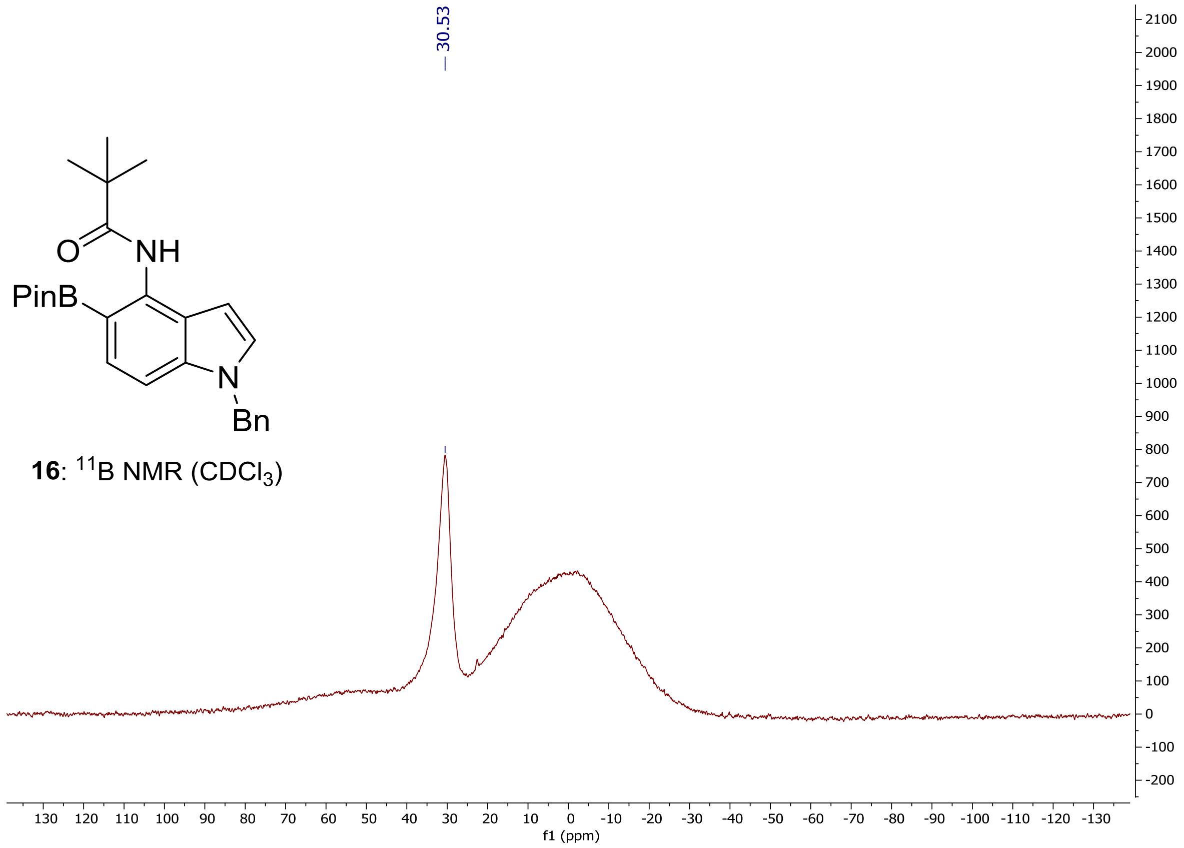
30.53
PinB
NH
O
N
Bn
16: ^{11}B NMR ($CDCl_3$)
f1 (ppm)

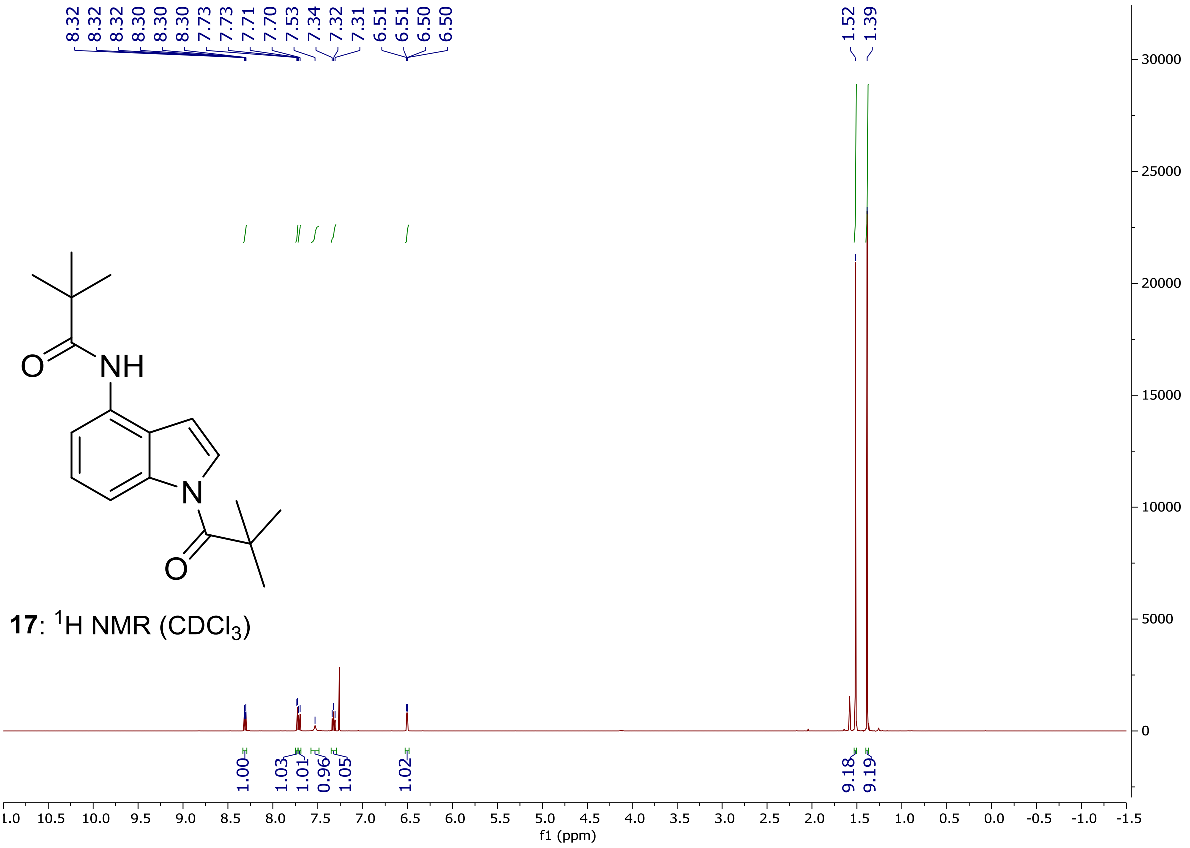

17: ^{1}H NMR ($CDCl_3$)

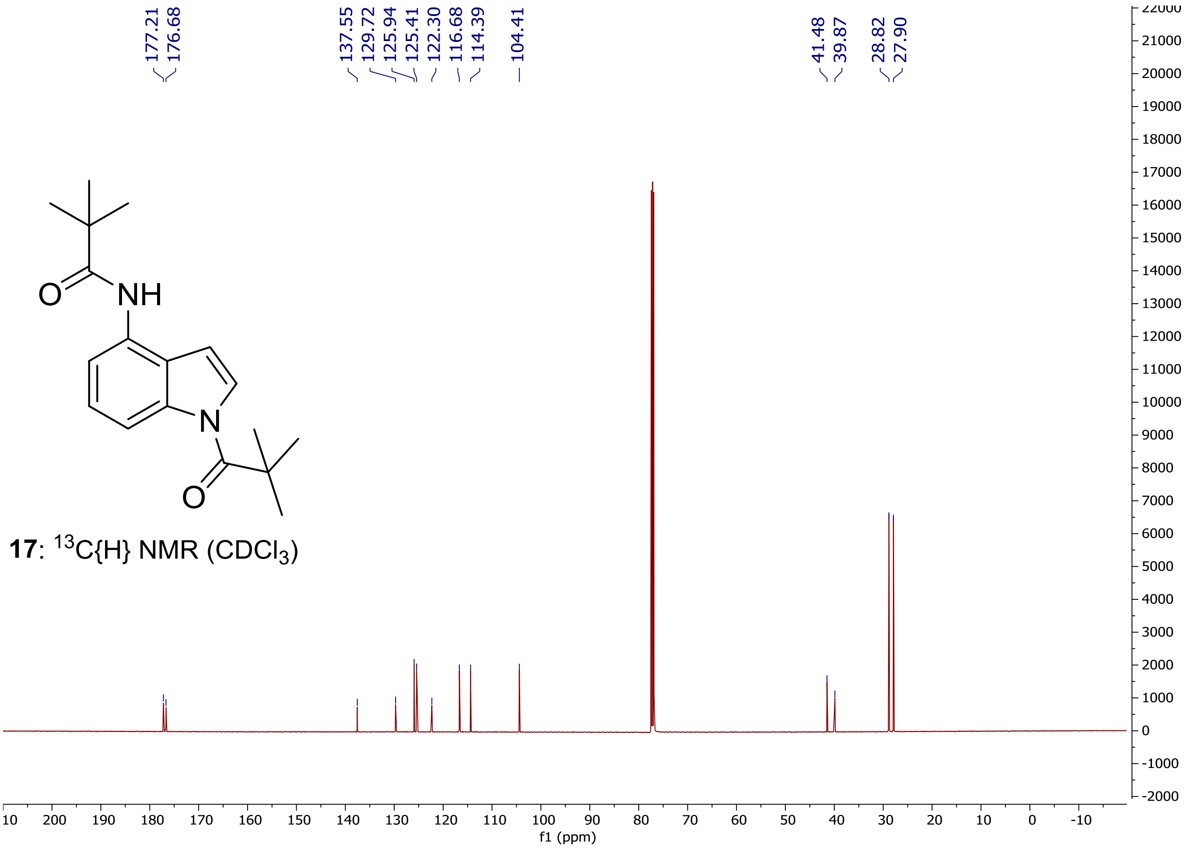

17: $^{13}C\{H\}$ NMR ($CDCl_3$)

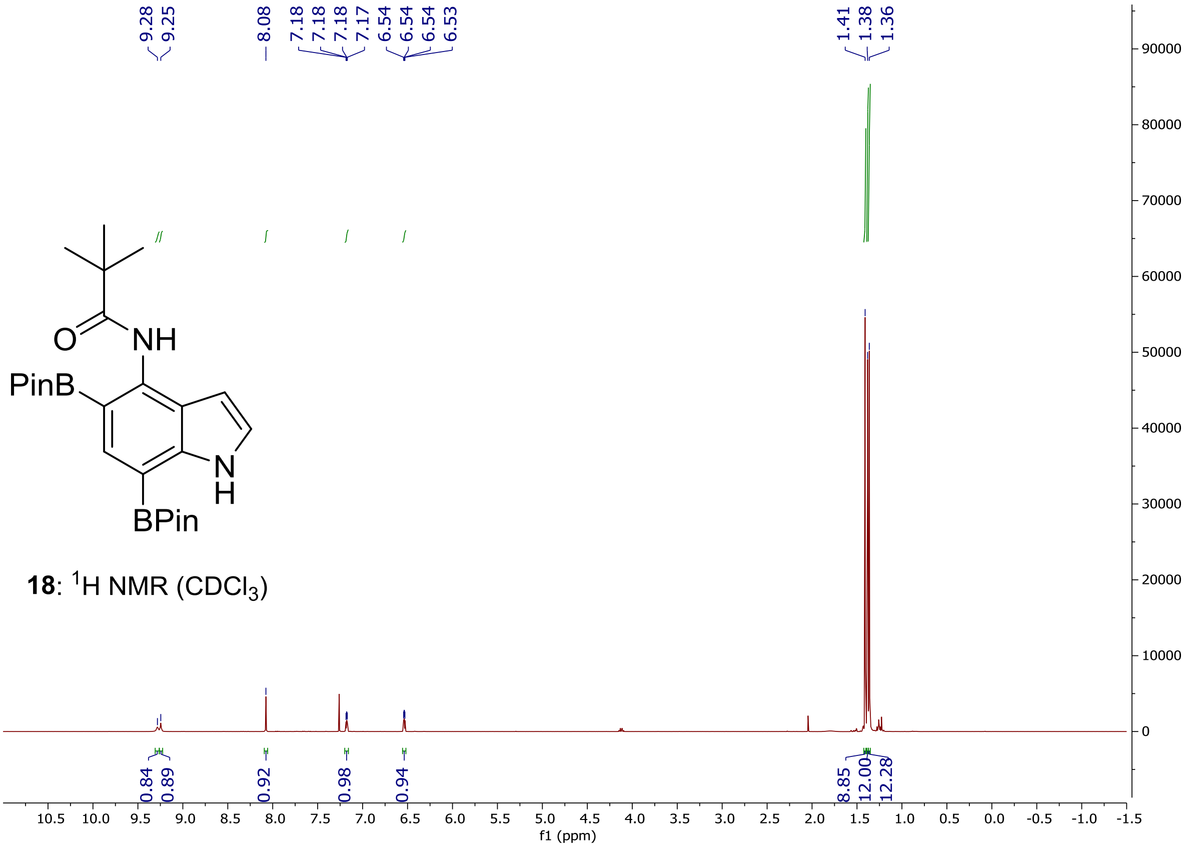

18: ^{1}H NMR ($CDCl_3$)

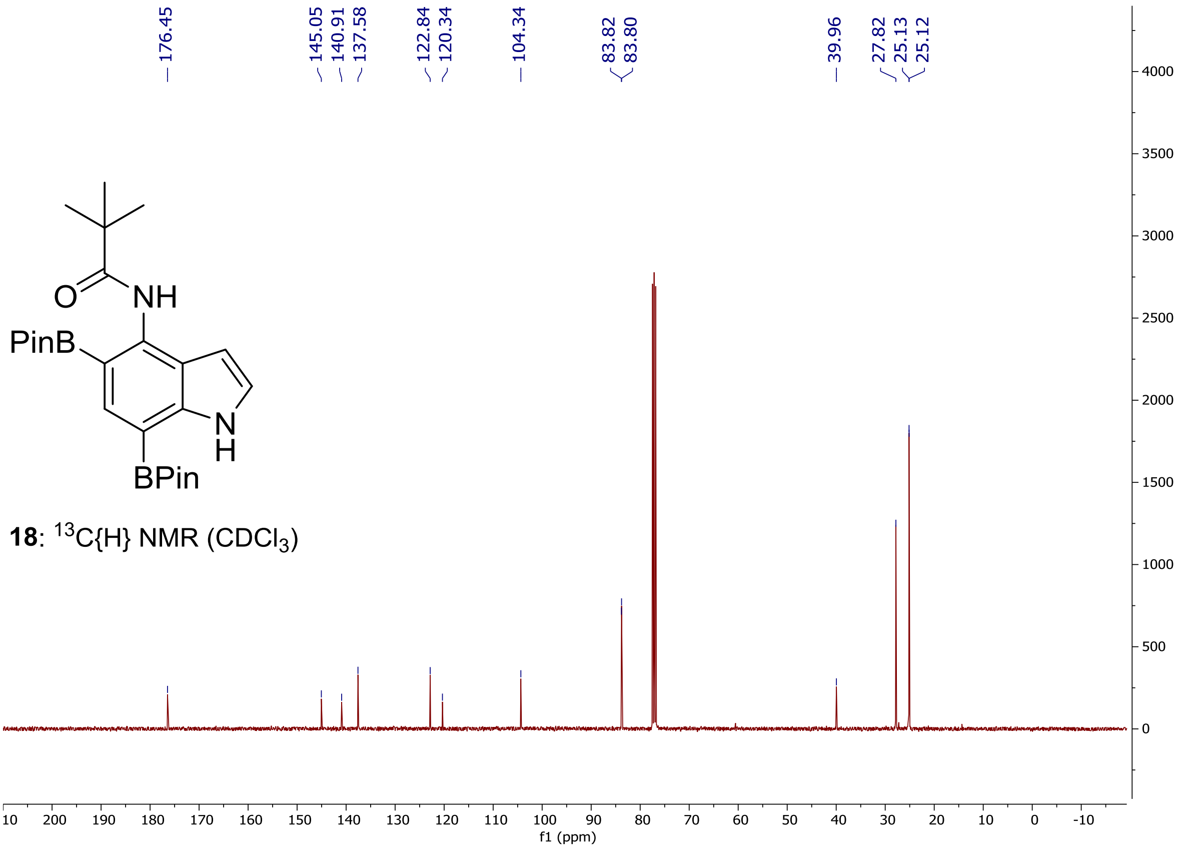

18: ^{13}C{H} NMR ($CDCl_3$)

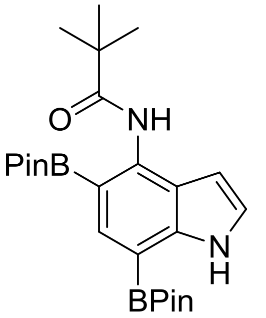

18: ^{11}B NMR ($CDCl_3$)

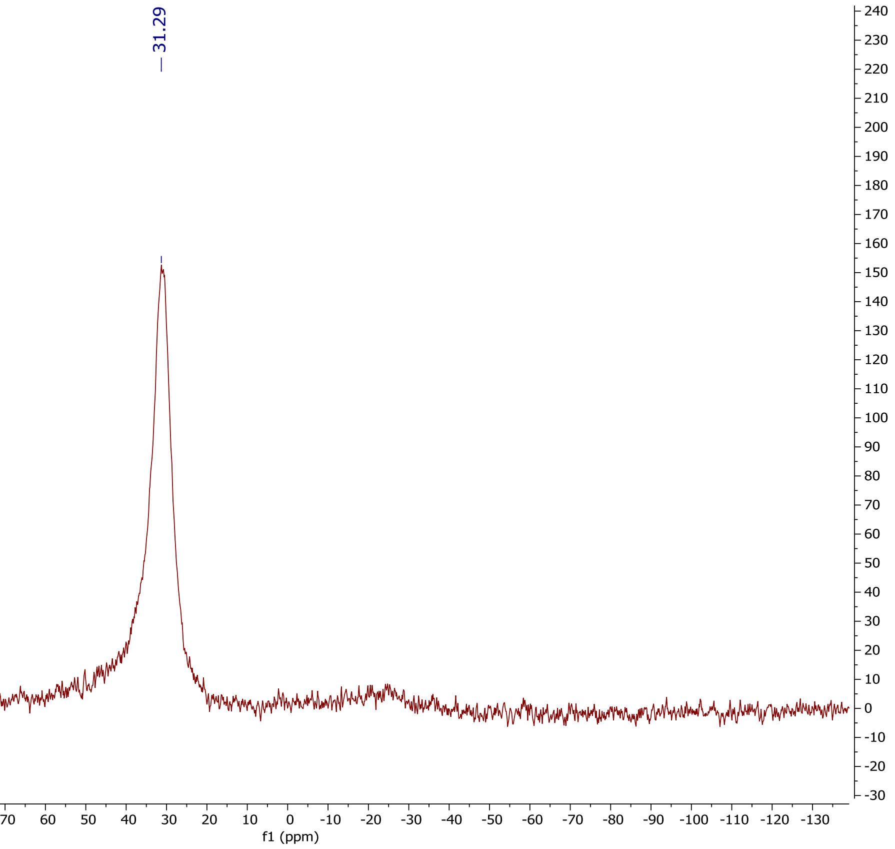

X-Ray crystallographic data

Crystallographic data for compound **7** were recorded on a dual source Rigaku FR-X rotating anode diffractometer equipped with a HyPix 6000 HE detector and an Oxford Cryostream 700 plus, at 100 K with Cu Kα radiation (mirror monochromator, λ = 1.54184). Crystallographic data for compounds **10** and **11** were recorded on an Agilent Supernova diffractometer, at 150 K with Mo Kα radiation (mirror monochromator, λ=0.7107). The CrysAlisPro[1] software package was used for data collection, cell refinement and data reduction. For all data sets the CrysAlisPro software package was used for empirical absorption corrections, which were applied using spherical harmonics, implemented in SCALE3 ABSPACK scaling algorithm. All further data processing was undertaken within the Olex2 software package.[2] The molecular structures of all compounds were solved with the ShelXT[3] structure solution program using Intrinsic Phasing and refined with the ShelXL[4-6] refinement package using Least Squares minimisation. Non-hydrogen atoms were refined anisotropically. Hydrogen atoms were all located in a difference map and repositioned geometrically. There is a solvent void present in structure **10** with poorly defined features in the electron density map, which was modelled using a solvent mask calculated with Olex2. It was calculated to contain 43.2 electrons and has been assigned as CH_2Cl_2 which was used as a solvent during crystallisation. Selected crystallographic data are presented in Table S1 and full details in cif format can be obtained free of charge from the Cambridge Crystallographic Data Centre via www.ccdc.cam.uk/data_request/cif.

Table S1. Selected crystallographic data for compounds **7**, **10** and **11**.

	**7**	**10**	**11**
CCDC No	1922252	1922250	1922251
Empirical formula	$C_{25}H_{24}BNO$	$C_{19}H_{13}BBr_3NO$	$C_{19}H_{12}BBr_3NO$
Formula Weight	365.26	521.84	440.93
Temperature (K)	99.8(5)	150.0(3)	150.0(3)
Radiation	1.54184	0.71073	0.71073
Crystal system	Triclinic	Monoclinic	Orthorhombic
Space group	P -1	C 2/c	P na2_1
a (Å)	9.8698(7)	11.7941(3)	12.6159(4)
b (Å)	10.3661(6)	16.6302(4)	14.5616(5)
c (Å)	10.9558(7)	21.8154(5)	8.9294(3)
α(°)	117.722(6)	90	90
β (°)	93.721(6)	101.175(2)	90
γ (°)	91.508(5)	90	90
Cell volume (Å^3)	988.11(12)	4197.71(18)	1640.40(9)

1. 1. CrysAlisPro, Agilent Technologies, Version 1.171.35.19 (release 27-10-2011 CrysAlis171 .NET) (compiled Oct 27 2011,15:02:11).
2. 2. O. V. Dolomanov, L. J. Bourhis, R. J. Gildea, J. A. K. Howard and H. Puschmann, *J. Appl. Crystallogr.* **2009**, *42*, 339-341.
3. 3. L. Palatinus, G. Chapuis, *J. Appl. Crystallogr.* **2007**, *40*, 786-790.
4. 4. L. Palatinus, A. Van der Lee, *J. Appl. Crystallogr.* **2008**, *41*, 975-984.
5. A. Palatinus, S. J. Prathapa, S. Van Smaalen, *J. Appl. Crystallogr.* **2012**, *45*, 575-580.
6. G. M. Sheldrick, *Acta Cryst.* **2008**, *A64*, 112-122.

Z	2	8	4
ρcalc (g.cm-3)	1.228	1.651	1.785
μ (mm^{-1})	0.563	5.772	4.946
F (000)	388.0	2016.0	864.0
Crystal size/ mm^3	0.2 x 0.2 x 0.1	0.3 x 0.3 x 0.1	0.3 x 0.05 x 0.05
2θ range for data collection/°	8.998 to 152.456	6.172 to 58.462	5.596 to 58.152
Index ranges	$-10 \leq h \leq 11$; $-13 \leq k \leq 12$; $-13 \leq l \leq 13$	$-15 \leq h \leq 15$; $-21 \leq k \leq 22$; $-16 \leq l \leq 29$	$-16 \leq h \leq 16$; $-19 \leq k \leq 19$; $-11 \leq l \leq 11$
Reflections collected	10745	11088	24323
Independent reflections	3873 [R_{int} = 0.0214; R_{sigma} = 0.0252]	4914 [R_{int} = 0.0260; R_{sigma} = 0.0587]	4100 [R_{int} = 0.0767; R_{sigma} = 0.0553]
Data/restraints/parameters	3873/0/256	4914/0/226	4100/1/217
Goodnes-of-fit-on F^2 (*GOF*)	1.068	1.003	1.069
Final *R* indices [$I > 2\sigma(I)$]	R_1 = 0.0424; wR_2 = 0.1080	R_1 = 0.0269; wR_2 = 0.0587	R_1 = 0.0373; wR_2 = 0.0725
R indices (all data)	R_1 = 0.0449; wR_2 = 0.1098	R_1 = 0.0355; wR_2 = 0.0620	R_1 = 0.0545; wR_2 = 0.0824
Largest diff. peak and hole (e $Å^{-3}$)	0.32 / -0.19	0.46 / -0.42	0.57 / -0.67
Flack parameter	n/a	n/a	-0.013 (0.011)

Computational details

All calculations were performed using the Gaussian09 series of programs.[1] Geometries were optimized with the DFT method using M06-2X functional[2] and 6-311G(d,p) as a basis set. All geometry optimizations were full, with no restrictions. All stationary points located in the potential energy hypersurface were characterized as minima (no imaginary frequencies) or as transition states (one and only one imaginary frequency) by vibrational analysis. The transition state was further confirmed by IRC calculations (calcALL, forward, maxpoints=100, stepsize=5. Solvent effects of the dichloromethane were introduced using the self consistent field approach, by means of the integral equation formalism polarizable continuum model (IEFPCM).[3] Full Cartesian coordinates for the optimised geometries are included in the .xyz file.

1. Gaussian 09, Revision C1, Frisch, M. J.; Trucks, G. W.; Schlegel, H. B.; Scuseria, G. E.; Robb, M. A.; Cheeseman, J. R.; Scalmani, G.; Barone, V.; Mennucci, B.; Petersson, G. A.; Nakatsuji, H.; Caricato, M.; Li, X.; Hratchian, H. P.; Izmaylov, A. F.; Bloino, J.; Zheng, G.; Sonnenberg, J. L.; Hada, M.; Ehara, M.; Toyota, K.; Fukuda, R.; Hasegawa, J.; Ishida, M.; Nakajima, T.; Honda, Y.; Kitao, O.; Nakai, H.; Vreven, T.; Montgomery, Jr., J. A.; Peralta, J. E.; Ogliaro, F.; Bearpark, M.; Heyd, J. J.; Brothers, E.; Kudin, K. N.; Staroverov, V. N.; Kobayashi, R.; Normand, J.; Raghavachari, K.; Rendell, A.; Burant, J. C.; Iyengar, S. S.; Tomasi, J.; Cossi, M.; Rega, N.; Millam, J. M.; Klene, M.; Knox, J. E.; Cross, J. B.; Bakken, V.; Adamo, C.; Jaramillo, J.; Gomperts, R.; Stratmann, R. E.; Yazyev, O.; Austin, A. J.; Cammi, R.; Pomelli, C.; Ochterski, J. W.; Martin, R. L.; Morokuma, K.; Zakrzewski, V. G.; Voth, G. A.; Salvador, P.; Dannenberg, J. J.; Dapprich, S.; Daniels, A. D.; Farkas, Ö.; Foresman, J. B.; Ortiz, J. V.; Cioslowski, J.; Fox, D. J. Gaussian, Inc., Wallingford CT, 2009.
2. Zhao, Y.; Truhlar, D. G. *Theor. Chem. Acc.* **2008**, *120*, 215-241.
3. Mennucci B.; Cancès E.; Tomasi J., *J. Phys. Chem. B* **1997**, *101*, 10506-10517.

(a)

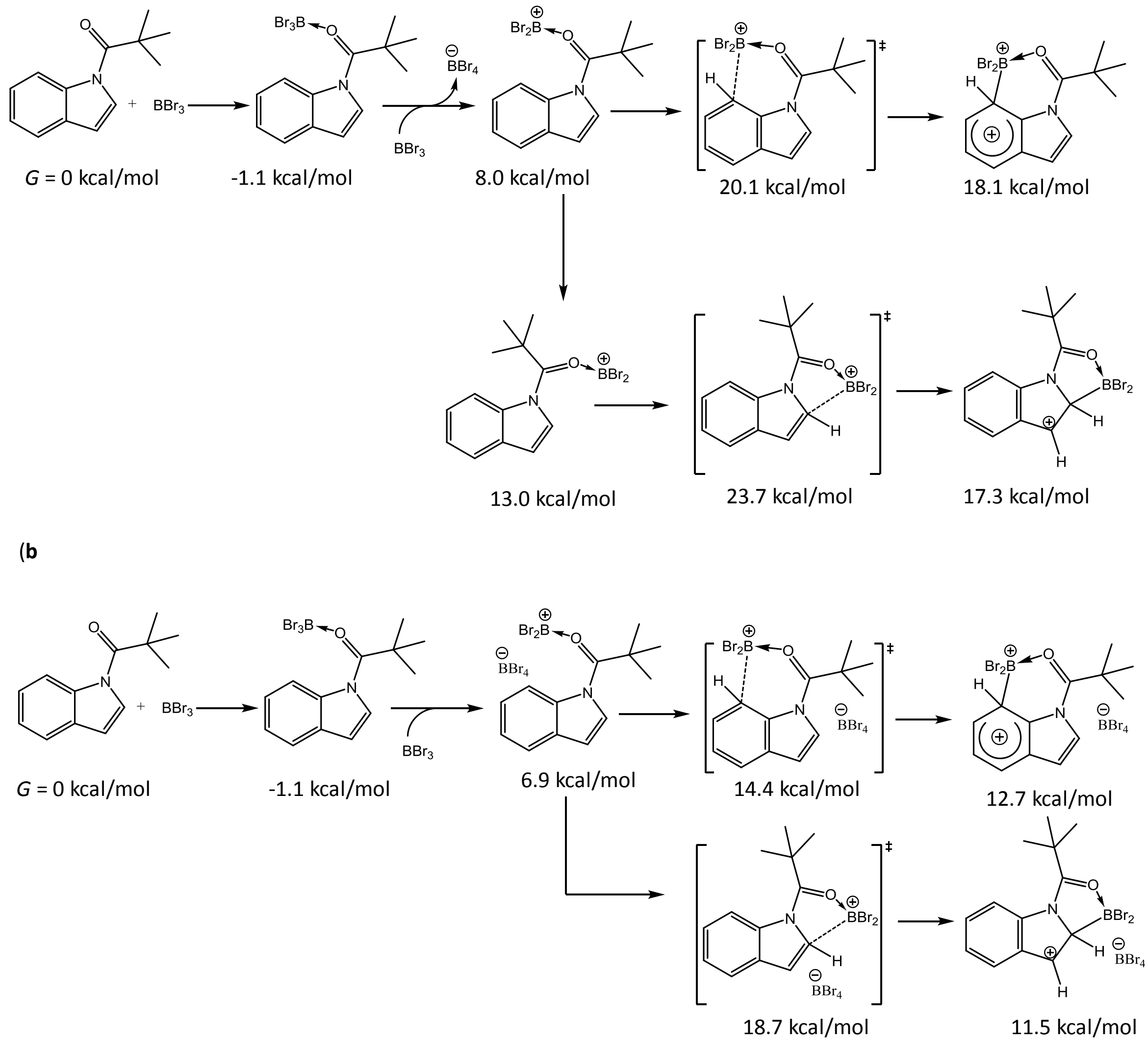

Scheme S1. DFT calculation on C2/C7 borylation of indole at M06-2X/6-311g(d,p) level. (a): without counteranion; (b) with counteranion. In a closely related system studied by M. Uchiyama and co-workers, the Wheland formation step for the directed borylation is the rate determine step.[4] In this study, we found the TS of Wheland formation for C7 position is lower in energy than that of C2 position.

4. Wang, D.-Y.; Minami, H.; Wang, C.; Uchiyama, M. *Chem. Lett.* **2015**, *44*, 1380-1382.
